# The RsmA RNA-Binding Proteins in *Pseudomonas syringae* Exhibit Distinct and Overlapping Roles in Modulating Virulence and Survival Under Different Nutritional Conditions

**DOI:** 10.3389/fpls.2021.637595

**Published:** 2021-02-26

**Authors:** Jun Liu, Menghao Yu, Yixin Ge, Yanli Tian, Baishi Hu, Youfu Zhao

**Affiliations:** ^1^College of Plant Protection and Key Laboratory of Integrated Management of Crop Diseases and Pests, Nanjing Agricultural University, Nanjing, China; ^2^Department of Crop Sciences, University of Illinois at Urbana-Champaign, Urbana, IL, United States

**Keywords:** *Pseudomonas syringae*, RsmA, CsrA, non-coding small RNA, T3SS, syringafactin, ROS

## Abstract

The post-transcriptional regulator RsmA globally controls gene expression in bacteria. Previous studies showed that RsmA2 and RsmA3 played critical roles in regulating type III secretion system (T3SS), motility, syringafactin, and alginate productions in *Pseudomonas syringae* pv. *tomato* strain DC3000 (*Pst*DC3000). In this study, we investigated global gene expression profiles of the wild-type *Pst*DC3000, the *rsmA3* mutant, and the *rsmA2/A3* double mutant in the *hrp*-inducing minimum medium (HMM) and King’s B (KB) medium. By comparing the *rsmA2/A3* and *rsmA3* mutants to *Pst*DC3000, a total of 1358 and 1074 differentially expressed genes (DEGs) in HMM, and 870 and 1463 DEGs in KB were uncovered, respectively. When comparing the *rsmA2/A3* mutant with the *rsmA3* mutant, 277 and 741 DEGs in HMM and KB, respectively, were revealed. Transcriptomic analysis revealed that the *rsmY*, *rsmZ*, and *rsmX1-5* non-coding small RNAs (ncsRNAs) were positively affected by RsmA2 and RsmA3, while RsmA3 positively regulates the expression of the *rsmA2* gene and negatively regulates both *rsmA1* and *rsmA5* gene expression. Comparative transcriptomic analysis showed that RsmA2 and RsmA3 synergistically influenced the expression of genes involved in T3SS and alginate biosynthesis in HMM and chemotaxis in KB. RsmA2 and RsmA3 inversely affected genes involved in syringafactin production in HMM and ribosomal protein biosynthesis in KB. In addition, RsmA2 played a major role in influencing genes involved in sarcosine and thiamine biosynthesis in HMM and in mannitol and phosphate metabolism in KB. On the other hand, genes involved in fatty acid metabolism, cellulose biosynthesis, signal transduction, and stress responses were mainly impacted by RsmA3 in both HMM and KB; whereas RsmA3 played a major role in controlling genes involved in c-di-GMP, phosphate metabolism, chemotaxis, and capsular polysaccharide in HMM. Furthermore, regulation of syringafactin production and oxidative stress by RsmA2 and RsmA3 was experimentally verified. Our results suggested the potential interplay among the RsmA proteins, which exhibit distinct and overlapping roles in modulating virulence and survival in *P. syringae* under different nutritional conditions.

## Introduction

*Pseudomonas syringae* pv. *tomato* strain DC3000 (*Pst*DC3000), a pathogen of tomato, *Brassica* spp. (cabbage and cauliflower), and *Arabidopsis thaliana* (Whalen et al., 1991; Wang et al., 2002; Sreedharan et al., 2006), is a model strain for studies in molecular mechanisms of bacterial pathogenesis and in plant–microbe interactions (Xin and He, 2013). Over 30 effectors in *Pst*DC3000 have been identified to be secreted and translocated into host cells via the type III secretion system (T3SS) to promote disease (Boch et al., 2002; Guttman et al., 2002; Zhao et al., 2003). The expression of the T3SS genes is activated by a HrpL-RpoN sigma factor cascade and bacterial enhancer-binding proteins (EBPs) HrpRS (Alarcón-Chaidez et al., 2003; Tang et al., 2006; Xie et al., 2019). Besides T3SS, phytotoxin coronatine (COR), extracellular protease, and alginate all contribute to the virulence of *Pst*DC3000 (Brooks et al., 2004; Ishiga et al., 2018).

Previous studies have reported that the GacS/GacA two-component system (TCS) affects virulence via regulation of motility, biofilm formation, quorum sensing (QS), stress response, secondary metabolites, and production of extracellular enzymes (Heeb and Haas, 2001; Lapouge et al., 2008; Sonnleitner et al., 2009). GacS, a sensor kinase, senses one or more signals and phosphorylates itself and GacA, a response regulator (Heeb and Haas, 2001). Phosphorylated GacA specifically activates non-coding small RNAs (ncsRNAs), e.g., *csrB* and *csrC*, in *Escherichia coli* (Gudapaty et al., 2001; Suzuki et al., 2002), *rsmY* and *rsmZ* in *Pseudomonas aeruginosa* (Kay et al., 2006; Janssen et al., 2018), and *rsmX1-5*, *rsmY*, and *rsmZ* in *Pst*DC3000 (Moll et al., 2010; Ge et al., 2019). The ncsRNAs contain many GGA motifs which exhibit high affinity with the RNA-binding protein CsrA (carbon storage regulator) or its homologs RsmA and RsmE (repressor of secondary metabolites), thus sequestering and antagonizing their functions (Reimmann et al., 2005; Duss et al., 2014; Vakulskas et al., 2015).

As post-transcriptional regulators, the CsrA/RsmA family proteins bind to specific GGA motifs of the RNA secondary structures in the 5′ untranslated regions (UTRs), thus affecting mRNA stability, riboswitch function, transcript elongation, and repressing or activating translation of target genes (Schubert et al., 2007; Vakulskas et al., 2015; Pourciau et al., 2020). It has been revealed that CsrA/RsmA proteins act as global virulence regulators for many bacterial pathogens in both animals and plants. The *rsmA* mutant of *P. aeruginosa* could not activate T3SS, resulting in reduced depolymerization, cytotoxicity, and anti-internalization during interaction with airway epithelial cells (Mulcahy et al., 2006). In *Pseudomonas amygdali pv. phaseolicola*, RsmA and RsmE redundantly controlled phaseolotoxin biosynthesis and virulence (Ramírez-Zapata et al., 2020). In *Erwinia amylovora*, CsrA positively regulated genes involved in T3SS, amylovoran production, and motility and activated the Rcs phosphorelay system by binding to *rcsB* (Ancona et al., 2016; Lee et al., 2019). In *Xanthomonas citri*, RsmA directly regulated the T3SS master regulator HrpG, and mutation of the *rsmA* gene decreased exopolysaccharide (EPS) production and abolished hypersensitive response (HR) in non-host plants (Andrade et al., 2014).

*Pseudomonas syringae* pv. *tomato* strain DC3000 contains five RsmA protein homologs, i.e., RsmA1 to RsmA5. Previous studies revealed that RsmA2 and RsmA3 were required for T3SS, motility, coronatine toxin, pyoverdine, syringafactin, and alginate productions and *in planta* development of disease symptoms and exhibited strong binding affinities to *rsmX1*, *rsmX5*, *rsmY*, and *rsmZ* ncsRNAs (Ge et al., 2019). In this study, we used RNA-seq to investigate the global gene expression profiles of *Pst*DC3000 and the *rsmA3* and *rsmA2/A3* mutants in both the *hrp*-inducing minimum medium (HMM) and King’s B medium (KB). Comparative transcriptomic analysis revealed distinct and overlapping roles in gene regulation by RsmA2 and RsmA3 in *Pst*DC3000.

## Materials and Methods

### Bacterial Strains and Growth Conditions

All strains used in this study were as reported previously (Ge et al., 2019). The wild-type *Pst*DC3000 and its *rsmA* mutants were routinely cultured in KB medium at 28°C with shaking at 250 rpm. The HMM, supplemented with 10 mM fructose as carbon source, and KB medium were used for RNA isolation (Huynh et al., 1989; Ge et al., 2019). Bacterial growth was monitored by measuring the absorbance of cell suspensions at 600 nm. Antibiotics were supplied at the following final concentrations: 100 μg/ml rifampicin, 50 μg/ml kanamycin, and 100 μg/ml ampicillin.

### RNA Extraction

Overnight cultures of the bacterial strains were collected by centrifugation and washed with HMM or KB for three times, respectively. The suspensions were adjusted to OD_600_ = 0.2 in HMM and KB and incubated at 18 and 28°C for 6 h, respectively. The OD values for samples were similar at collection time. Four ml of RNA protect reagent (Qiagen, Hilden, Germany) was added to 2 ml of bacterial culture mixed by vortex and incubated at room temperature for 5 min. Cells were harvested by centrifugation, and total RNAs were extracted using RNeasy^®^ mini kit (Qiagen, Hilden, Germany) according to the manufacturer’s instructions. DNase I treatment was performed with TURBO DNA-free kit (Ambion, Austin, TX, United States). The quantity and quality of RNA samples were determined using a Nano-drop ND100 spectrophotometer (Nano-Drop Technologies, Wilmington, DE, United States) and/or using Agilent RNA 6000 Nano Chip Bioanalyzer (Agilent, Santa Clara, CA, United States).

### RNA-seq Analysis

Library construction and sequencing of three biological samples each of *Pst*DC3000 and its *rsmA* mutants were performed using the Illumina HiSeq 4000 (Illumina, San Diego, CA, United States) by the Keck Center at the University of Illinois, Urbana-Champaign. Ribosomal RNA was removed with the Ribo-zero Bacteria kit (Illumina), and a total of 18 stranded libraries were constructed using the TruSeq Stranded RNA Sample Prep kit following the manufacturer’s instructions (Illumina, San Diego, CA, United States). The sequence reads were aligned to the genome of *Pst*DC3000 (GenBank accession #: AE016853.1) (Buell et al., 2003) using Bowtie2 version 2.3.2 (Langmead and Salzberg, 2012). Samtools and bedtools were performed for getting the read counts per coding sequence (CDS). Normalized log_2_-based count per million values (log_2_CPM) was calculated after trimmed mean of *M* value (TMM) normalization using the edgeR package (Robinson et al., 2010).

To examine gene expression dynamics among all the samples, a multidimensional scaling (MDS) was drawn using glMDSPlot function in R. Differentially expressed genes (DEGs) were detected using edgeR and defined as genes with a | FC (Fold change)| value ≥ 1.5 and a corrected *p* value < 0.05 from three biological samples. To visualize the overall expression pattern of individual genes, the MA plots (a.k.a., mean-difference plots; log_2_FC versus average log_2_CPM; FC, fold change; CPM, counts per million reads) and heat maps were, respectively generated using plotMD and heatmap.2 functions in R. Protein sequences of all coding genes in *Pst*DC3000 (gene bank accession #: AE016853.1) were downloaded from NCBI website^1^. The FASTA protein file was used as input for protein annotation using eggNOG-mapper^2^. Clusters of orthologous groups (COGs) information for DEGs was extracted from the eggNOG output file. In addition, genes involved in T3SS were manually grouped into an additional orthologous categorization. RNA-seq data files have been submitted to Gene Expression Omnibus (GEO) at the National Center for Biotechnology Information (NCBI) with an accession number GSE162091.

### Identification of Genes Specifically Affected by RsmA2, RsmA3, or Both

Venn diagrams were drawn by analyzing the DEG lists for comparisons of the *rsmA23* mutant versus *Pst*DC3000, the *rsmA23* mutant versus the *rsmA3* mutant, and the *rsmA3* mutant versus *Pst*DC3000 in both HMM and KB. DEGs up-regulated (or down-regulated) simultaneously in all three comparisons were considered to be synergistically regulated by RsmA2 and RsmA3, whereas DEGs inversely expressed in two comparisons (*rsmA23* versus *rsmA3* and *rsmA3* versus *Pst*DC3000) were considered to be inversely regulated by RsmA2 and RsmA3. On the other hand, DEGs found in comparison of *rsmA23* versus *Pst*DC3000 and *rsmA23* versus *rsmA3*, but not in *rsmA3* versus *Pst*DC3000 comparison, were considered to be mainly regulated by RsmA2, whereas DEGs found in comparison of *rsmA23* versus *Pst*DC3000 and Δ*rsmA3* versus *Pst*DC3000, but not in *rsmA23* versus *rsmA3* comparison, were considered to be mainly regulated by RsmA3.

### Quantitative Real-Time PCR (qRT-PCR)

For quantitative Real-Time PCR (qRT-PCR), 1 μg of RNA was reversed to cDNA using the SuperScript^TM^ III Reverse Transcriptase following the manufacturer’s instructions (Invitrogen, Carlsbad, CA, United States). Concentration of cDNA was adjusted to 100 ng/μl and used as template for qRT-PCR. The PowerUp SYBR^®^ Green PCR master mix (Applied Biosystems, Foster, CA, United States) was used to detect the gene expression of selected genes. The qRT-PCR amplifications were performed in the StepOnePlus Real-Time PCR system (Applied Biosystems, Foster, CA, United States) under the following procedure: 50°C for 2 min, and 95°C for 2 min followed by 40 cycles of 95°C for 15 s and 60°C for 1 min. The *rpoD* was used as an endogenous control to calculate relative quantification (ΔΔC_*t*_) (Ge et al., 2019). All primers are listed in Supplementary Table 1. The experiment was repeated, and three biological replicates were used for each gene.

### Detection of Syringafactin Production

Atomized oil assay was used to detect syringafactin as previously described (Burch et al., 2010). *Pst*DC3000 and all *rsmA* mutants were grown on KB agar plates for 48 h, resuspended in PBS, and adjusted to an OD_600_ = 1.0. Ten microliters was pipetted onto the surface of KB plates (1.5% agar) and incubated for 24 h at 20°C. An airbrush (VIVO HOME, Pleasanton, CA, United States) was used to spray a mist of mineral oil over the plates (light paraffin oil; Thermo Fisher Scientific, Waltham, MA, United States). Brighter oil drops formed a visible halo around bacterial colonies. The ring area of the halos was measured to represent syringafactin production. Experiments were performed in triplicate and repeated three times. Statistical comparison among different strains was performed using one-way ANOVA followed by Fisher’s LSD test (*p* < 0.05).

### Oxidative Stress Assay

Spot dilution assay was performed using a previously described procedure to detect oxidative sensitivity (Ge et al., 2018). Briefly, overnight bacterial cells were harvested by centrifugation and washed twice using PBS. After the final wash, the pellet was resuspended in PBS and adjusted to OD_600_ = 1. Tenfold serial dilutions of the bacterial suspension were made in PBS. Each dilution (5 μl) was spotted on the plates with different concentrations of H_2_O_2_ (0, 0.25, or 0.5 mM) and incubated at 28°C for 2 days. The experiment was performed in duplicate and repeated three times.

## Results

### Summary of Gene Expression Profiles

Previous studies showed that RsmA2 and RsmA3 played major roles in the virulence of *Pst*DC3000 and the *rsmA2/A3* double mutant exhibited dramatically reduced disease symptoms and *in planta* bacterial growth. Furthermore, RsmA2 and RsmA3 played distinct roles in regulating virulence factors, including T3SS and swarming motility (Ge et al., 2019). In order to further understand the global effects of RsmA2 and RsmA3, as well as their distinct roles in regulating gene expression, RNA-seq comparing the wild-type *Pst*DC3000, the *rsmA3* mutant (Δ*rsmA3*) and the *rsmA2/A3* double mutant (Δ*rsmA23*) were performed in both HMM and KB media. In total, 11,352,295 to 13,869,462 reads for each biological sample were generated for *Pst*DC3000, Δ*rsmA3*, and Δ*rsmA23* grown in HMM, and the percentage of reads mapped to the *Pst*DC3000 genome ranged from 81.2 to 99.7%, whereas 9,332,318 to 12,837,068 reads for each biological sample were obtained for *Pst*DC3000, Δ*rsmA3*, and Δ*rsmA23* in KB, and the percentage of reads mapped to *Pst*DC3000 genome was from 60.3 to 98.5%.

To explore the similarities and differences between these samples, MDS was conducted. MDS plot clearly showed that the first two dimensions explained about 60 and 21% of the variability in the whole datasets, respectively (Supplementary Figure 1). Dimensions 1 and 2, respectively represented data variations due to different media and different strains (Supplementary Figure 1). The three biological samples of each treatment (strain/medium combination) were clustered together. Furthermore, the heat map also showed that the three biological samples of each strain in different media were very consistent (Supplementary Figure 2), indicating that the datasets were highly reproducible.

**FIGURE 1:**
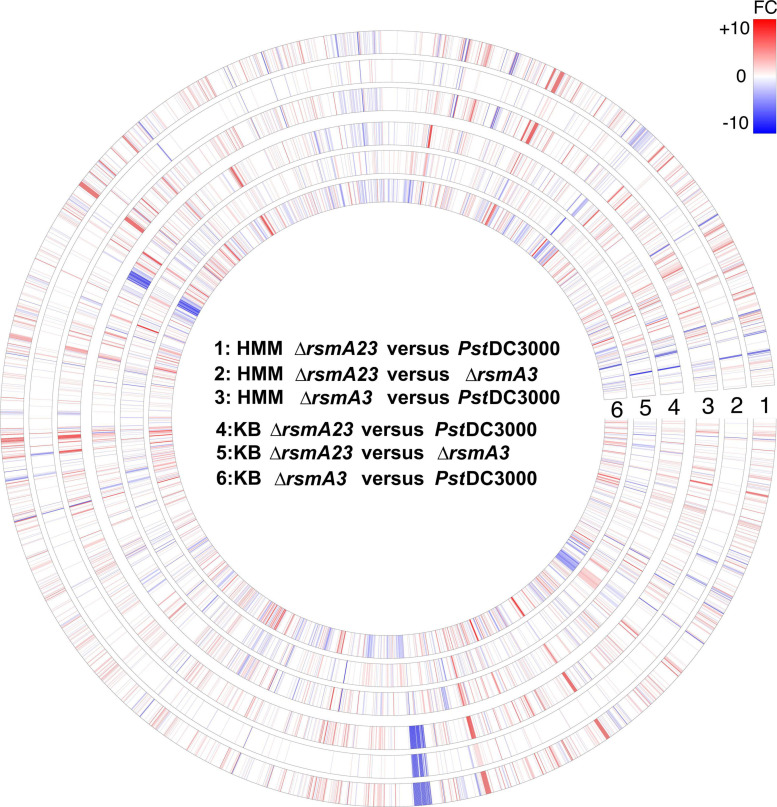
Circular map of RNA-seq data for all 5348 genes. For each gene, red or blue color represents fold change (FC) ≥ 1.5 and FC ≤ –1.5 with a *p* value < 0.05, respectively. The higher the | FC|, the deeper the color. Rings # 1, 2, and 3 represent FCs for comparison of Δ*rsmA23* versus *Pst*DC3000, Δ*rsmA23* versus Δ*rsmA3*, and Δ*rsmA3* versus *Pst*DC3000 in HMM, respectively. Rings # 4, 5, and 6 represent FCs for comparison of Δ*rsmA23* versus *Pst*DC3000, Δ*rsmA23* versus *rsmA3*, and Δ*rsmA3* versus *Pst*DC3000 in KB, respectively. HMM: *hrp*-inducing minimum medium; KB: King’s B medium; *Pst*DC3000: *Pseudomonas syringae* pv. *tomato* DC3000; Δ*rsmA3*: the *rsmA3* mutant; Δ*rsmA23*: the *rsmA2/A3* double mutant.

To show the overall transcriptomic profiles, a circle plot (Figure 1) was constructed where the expression of all 5348 genes was displayed by comparing Δ*rsmA23* versus *Pst*DC3000, Δ*rsmA23* versus Δ*rsmA3*, and Δ*rsmA3* versus *Pst*DC3000 in both HMM and KB. Among the 5348 genes, a total of 2661 genes exhibited a differential expression with a | Fold change (FC)| value ≥ 1.5 and a *p* value < 0.05 between *Pst*DC3000, Δ*rsmA3*, and Δ*rsmA23* grown in both HMM and KB media (Supplementary Figure 2A). These 2661 genes were designated as DEGs, representing about half of all the genes in the *Pst*DC3000 genome. Among the 2661 DEGs, 1560 and 1879 were differentially expressed in HMM and KB, respectively (Supplementary Figures 2B,C and Supplementary Tables 2, 3).

Specifically, by comparing the *rsmA2/A3* and *rsmA3* mutants with the wild-type *Pst*DC3000, a total of 1358 and 1074 DEGs in HMM, and 870 and 1463 DEGs in KB were uncovered, respectively (Supplementary Figures 3A,C,D,F). When comparing the *rsmA2/A3* double mutant with the *rsmA3* mutant, a total of 277 and 741 DEGs were discovered in HMM and KB, respectively (Supplementary Figure 3B,E), suggesting that more genes were influenced by RsmA2 in KB than in HMM. DEGs were then functionally classified based on COGs. A total of 797, 177, and 601 DEGs in HMM (Supplementary Figure 4A,B,C) and 498, 502, and 949 DEGs in KB (Supplementary Figure 4D,E,F) were functionally categorized into 20 known function categories in the three comparisons (Δ*rsmA23* versus *Pst*DC3000, Δ*rsmA23* versus Δ*rsmA3*, and Δ*rsmA3* versus *Pst*DC3000), respectively. By comparing the *rsmA2/A3* double mutant with the *rsmA3* mutant, more DEGs in broad functional categories were found in KB than in HMM (Supplementary Figure 4B,E), further suggesting that RsmA2 might play important roles in KB than in HMM.

To verify the RNA-seq data, seven genes were selected from *Pst*DC3000, including genes encoding catalase (*katE*), adenylate cyclase (*cyaA*), transcriptional regulator FleQ (*fleQ*), phosphate regulon transcriptional regulatory protein (*phoB*), citrate transporter (*citM*), sensor histidine kinase (*ladS*), and pyruvate kinase (*pyk*). The qRT-PCR results showed that expression of these genes showed a similar trend with those of the RNA-seq data (Supplementary Figure 5).

### Expression of *rsmX/Y/Z* ncsRNAs and *rsmAs* in *Pst*DC3000 Wild-Type and *rsmA* Mutant Derivatives

Previous studies revealed that the RsmA family protein positively regulates the transcription of *rsmY* and *rsmZ* ncsRNAs in *Pseudomonas fluorescens* CHA0 (Reimmann et al., 2005). In *Pst*DC3000, RsmA2 and RsmA3 exhibited stronger binding affinities to ncsRNAs (Ge et al., 2019). In this study, *rsmX1-5*, *rsmY*, and *rsmZ* ncsRNAs were down-regulated in the *rsmA3* and the *rsmA2/A3* double mutants as compared with *Pst*DC3000 (Table 1). Except for *rsmY* and *rsmX4*, the FCs of *rsmZ*, *rsmX1*, *X2*, *X3*, and *X5* were much lower in Δ*rsmA23* versus *Pst*DC3000 than those in Δ*rsmA3* versus *Pst*DC3000 in both HMM and KB media (Table 1). The FCs of *rsmY* and *rsmX4* in both comparisons in both media were similar. These results suggested that *rsmY* (and possibly *rsmX4*) were mainly influenced by RsmA3, whereas *rsmZ*, *rsmX1*, *X2*, *X3*, and *X5* were synergistically affected by RsmA2 and RsmA3.

**TABLE 1:** Fold changes of the non-coding small RNAs and the *rsmA* genes in HMM and KB.

** Locus tag**	**Medium comparison gene/small RNA**	**HMM Δ*rsmA23*/ *Pst*DC3000**	**HMM Δ*rsmA23*/Δ *rsmA3***	**HMM Δ*rsmA3*/ *Pst*DC3000**	**KB Δ*rsmA23/Pst*DC3000**	**KB Δ *rsmA23*/Δ *rsmA3***	**KB Δ *rsmA3*/ *Pst*DC3000**
*PSPTO_5647*	*rsmY*	−2.09	/	−1.89	−2.43	/	−1.64
*PSPTO_5652*	*rsmZ*	−22.19	−6.25	−3.55	−42.31	−23.20	−1.8
*PSPTO_5671*	*rsmX1*	−9.83	−3.37	−2.91	−91.63	−8.94	−10.25
*PSPTO_5672*	*rsmX2*	−4.26	−2.7	−1.57	−16.63	/	−9.9
*PSPTO_5673*	*rsmX3*	−1.43	−1.48	/	−1.97	−1.9	/
*PSPTO_5674*	*rsmX4*	−14.47	/	−10.2	−60.09	/	−38.81
*PSPTO_5675*	*rsmX5*	−7.59	−4.52	−1.68	−18.7	−3.74	−4.9
*PSPTO_1629*	*rsmA1*	2.49	/	2.32	1.9	/	1.57
*PSPTO_1844*	*rsmA2*	−20	−15.56	/	−11.28	−6.16	−1.83
*PSPTO_3943*	*rsmA4*	/	/	/	/	/	/
*PSPTO_5621*	*rsmA5*	2.28	/	1.69	1.66	−2.93	4.86

*Differential expression in Δ*rsmA23* versus *Pst*DC3000, Δ*rsmA23* versus ΔrsmA3, and Δ*rsmA3* versus *Pst*DC3000 with fold change (FC) ≥ 1.5 or FC ≤ −1.5 and *p* value < 0.05. /: non-differentially expressed genes (DEGs); HMM: *hrp*-inducing minimum medium; KB: King’s B medium; *Pst*DC3000: *Pseudomonas syringae* pv. *tomato* DC3000; Δ*rsmA3*: the *rsmA3* mutant; Δ*rsmA23*: the *rsmA2/A3* double mutant.*

On the other hand, expression of the *rsmA2* gene was down-regulated in Δ*rsmA3* versus *Pst*DC3000, whereas the expression of the *rsmA1* and *rsmA5* genes was similarly up-regulated in Δ*rsmA3* versus *Pst*DC3000 and Δ*rsmA23* versus *Pst*DC3000 in both HMM and KB media (Table 1). No change was observed for the *rsmA4* gene (Table 1). These results suggested that RsmA3 might positively affect the expression of the *rsmA2* gene (Ge et al., 2019) and negatively influence both *rsmA1* and *rsmA5* gene expression in *Pst*DC3000.

### Transcriptomic Analysis Revealed Distinct and Overlapping Gene Regulation by RsmA2 and RsmA3 in *Pst*DC3000

**FIGURE 2:**
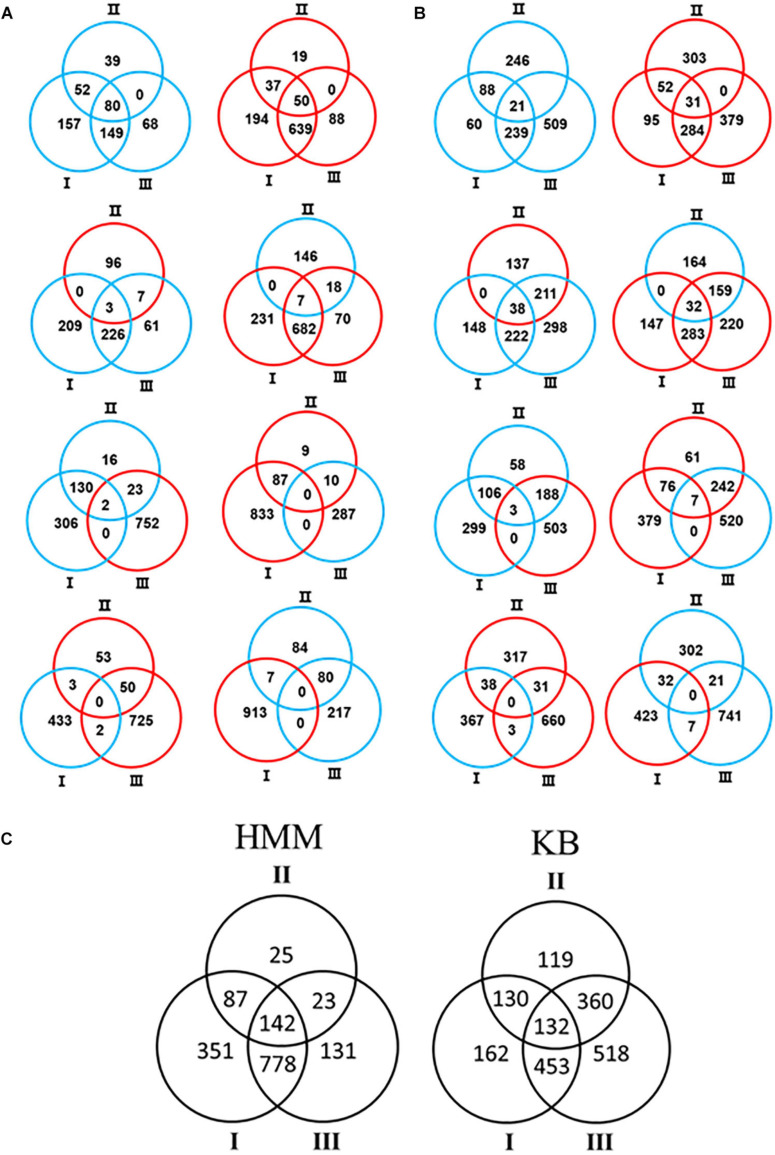
Venn diagram showed number of differentially expressed genes (DEGs) in each of the three comparisons. **(A)** HMM. **(B)** KB. Red and blue circles represent up- and down-regulated genes, respectively. **(C)** Combination of HMM and KB data. Black circles represent differential expression including both up- and down-regulated genes. Comparison I: Δ*rsmA23* versus *Pst*DC3000; II: Δ*rsmA23* versus Δ*rsmA3*; III: Δ*rsmA3* versus *Pst*DC3000; HMM: *hrp*-inducing minimum medium; KB: King’s B medium; *Pst*DC3000: *Pseudomonas syringae* pv. *tomato* DC3000; Δ*rsmA3*: the *rsmA3* mutant; Δ*rsmA23*: the *rsmA2/A3* double mutant.

**FIGURE 3:**
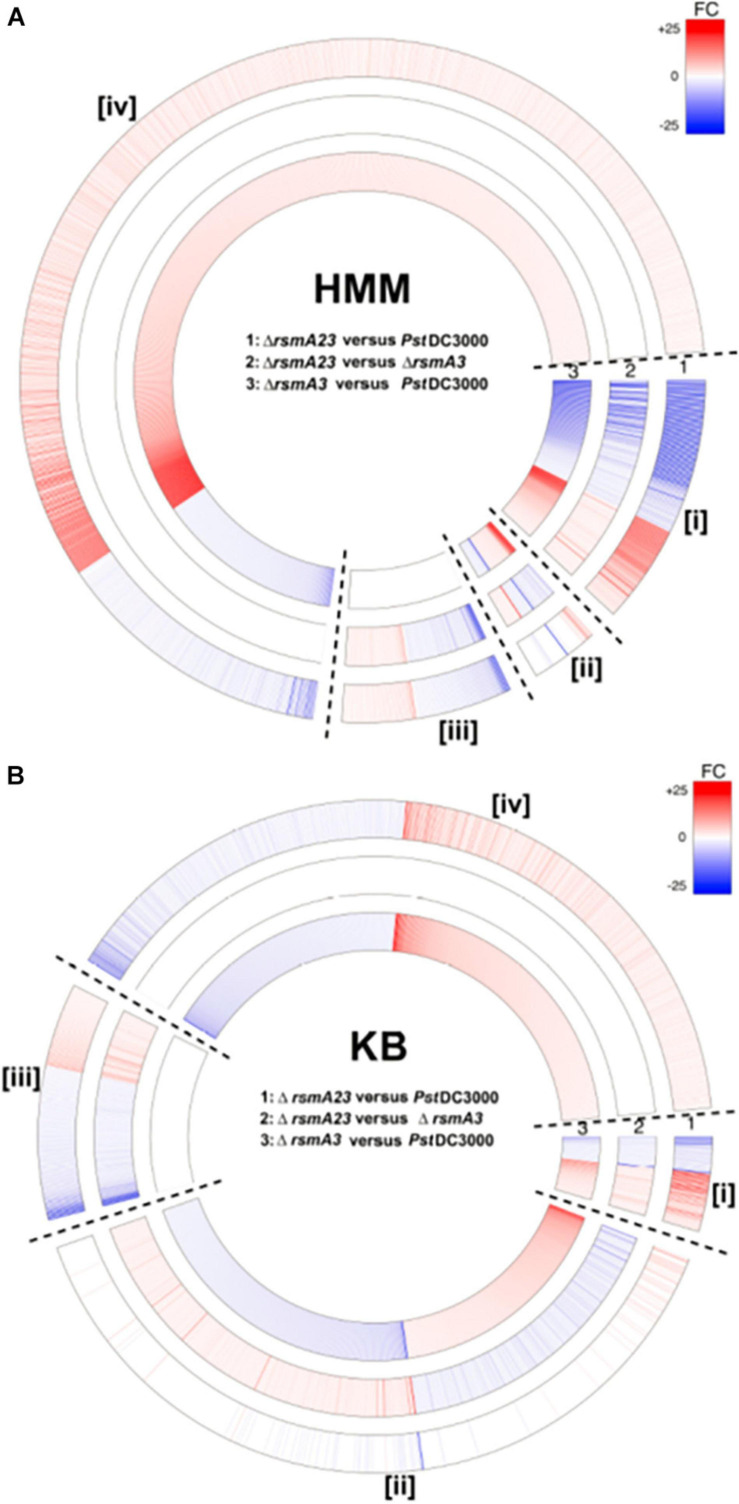
Circular map of differentially expressed genes (DEGs) based on differential regulation by RsmA2 and RsmA3 in *Pst*DC3000. **(A)** Fold changes (FCs) of Δ*rsmA23* versus *Pst*DC3000 (#1), Δ*rsmA23* versus Δ*rsmA3* (#2), and Δ*rsmA3* versus *Pst*DC3000 (#3) in HMM. **(B)** FCs of Δ*rsmA23* versus *Pst*DC3000 (#1), Δ*rsmA23* versus Δ*rsmA3* (#2), and Δ*rsmA3* versus *Pst*DC3000 (#3) in KB. Group [i] represents genes synergistically regulated by RsmA2 and RsmA3; group [ii] represents genes inversely regulated by RsmA2 and RsmA3; group [iii] represents genes regulated mainly by RsmA2; and group [iv] represents genes regulated mainly by RsmA3 (see Table 2). DEGs were defined as genes with a | Fold Change (FC)| value ≥ 1.5 and a *p* value < 0.05. Up- and down-regulated DEGs were indicated by red and blue, respectively. The higher the | FC|, the deeper the color. HMM: *hrp*-inducing minimum medium; KB: King’s B medium; *Pst*DC3000: *Pseudomonas syringae* pv. *tomato* DC3000; Δ*rsmA3*: the *rsmA3* mutant; Δ*rsmA23*: the *rsmA2/A3* double mutant.

In order to comprehensively understand the differential role of RsmA2 and RsmA3 in regulating gene expression, Venn diagrams were generated to group genes differentially regulated by RsmA2 and RsmA3 in both HMM and KB media (Figure 2). The expression patterns in HMM and KB were further visualized using a circle plot and divided into four major groups (Figure 3). Group [i] includes 130 and 52 genes that were synergistically regulated by RsmA2 and RsmA3 in HMM and KB, respectively (Figure 3). Among them, 80 and 21 were positively affected by RsmA2 and RsmA3 in HMM and KB, respectively, whereas 50 and 31 were negatively influenced by RsmA2 and RsmA3 in HMM and KB, respectively (Figures 2A,B and Table 2). Group [ii] includes 35 and 440 genes that were inversely regulated by RsmA2 and RsmA3 in HMM and KB, respectively (Figure 3). Among them, the expression of 10 and 249 genes was inhibited by RsmA2 but activated by RsmA3 in HMM and KB, respectively (Figures 2A,B and Table 2). In contrast, 25 and 191 genes were activated by RsmA2 but suppressed by RsmA3 in HMM and KB, respectively (Figures 2A,B and Table 2). Group [iii] includes 87 and 130 genes that were mainly influenced by RsmA2 in HMM and KB, respectively (Figures 2C, 3). Among them, 50 and 85 were activated by RsmA2 in HMM and KG, respectively, whereas 37 and 45 genes were inhibited by RsmA2 in HMM and KB, respectively (Table 2). Group [iv] includes 778 and 453 genes that were mainly affected by RsmA3 in HMM and KB, respectively (Figures 2C, 3). Among them, 146 and 201 were activated by RsmA3 in HMM and KB, whereas 632 and 252 genes were suppressed by RsmA3 in HMM and KB, respectively (Table 2).

**TABLE 2:** Numbers of differentially expressed genes (DEGs) based on regulation by RsmA2 and RsmA3 in *Pst*DC3000.

	**HMM**			**KB**		
**Groups**	**Down**	**Up**	**Total**	**Down**	**Up**	**Total**
[i] Genes synergistically regulated by RsmA2 and RsmA3	80	50	130	21	31	52
[ii] Genes inversely regulated by RsmA2 and RsmA3	10	25	35	249	191	440
[iii] Genes regulated mainly by RsmA2	50	37	87	85	45	130
[iv] Genes regulated mainly by RsmA3	146	632	778	201	252	453

*The number of genes was generated from Figure 3. Down and up represent expression in comparisons, i.e., Δ*rsmA3* versus *Pst*DC3000, Δ*rsmA23* versus *Pst*DC3000, or Δ*rsmA23* versus Δ*rsmA3*. [i] DEGs up-regulated (or down-regulated) simultaneously in all three comparisons. [ii] DEGs inversely expressed in *ΔrsmA23* versus *ΔrsmA3* and *ΔrsmA3* versus *Pst*DC3000; down or up represents expression in Δ*rsmA3* versus *Pst*DC3000. [iii] DEGs in Δ*rsmA23* versus *Pst*DC3000 and Δ*rsmA23* versus Δ*rsmA3*, but not in Δ*rsmA3* versus *Pst*DC3000. [iv] DEGs in Δ*rsmA23* versus *Pst*DC3000 and Δ*rsmA3* versus *Pst*DC3000, but not in Δ*rsmA23* versus Δ*rsmA3*. HMM: *hrp*-inducing minimum medium; KB: King’s B medium; *Pst*DC3000: *Pseudomonas syringae* pv. *tomato* DC3000; Δ*rsmA3*: the *rsmA3* mutant; Δ*rsmA23*: the *rsmA2/A3* double mutant.*

### Overlapping and Distinct Gene Regulation by RsmA2 and RsmA3 in HMM

**FIGURE 4:**
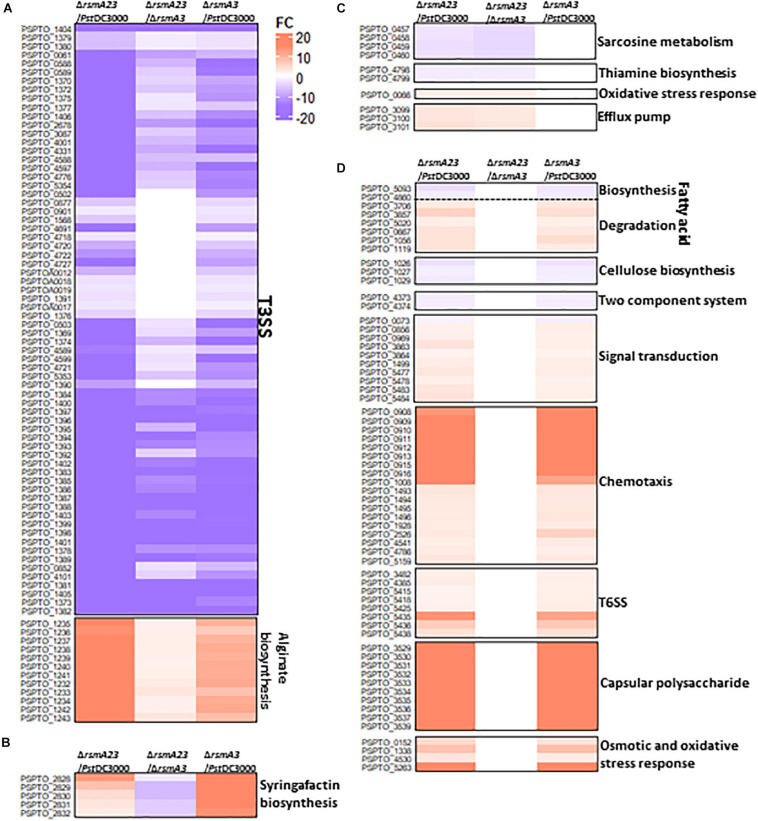
Heat maps of selected differentially expressed genes (DEGs) in HMM. **(A)** T3SS and alginate biosynthesis genes synergistically regulated by RsmA2 and RsmA3. **(B)** Syringafactin biosynthesis genes inversely regulated by RsmA2 and RsmA3. **(C)** Genes regulated mainly by RsmA2. **(D)** Genes regulated mainly by RsmA3. DEGs were defined as genes with a | Fold Change (FC)| value ≥ 1.5 and a *p* value < 0.05. Up- and down-regulated DEGs were indicated by red and blue, respectively. The higher the | FC|, the deeper the color. HMM: *hrp*-inducing minimum medium; *Pst*DC3000: *Pseudomonas syringae* pv. *tomato* DC3000; Δ*rsmA3*: the *rsmA3* mutant; Δ*rsmA23*: the *rsmA2/A3* double mutant; T3SS, type III secretion system; T6SS, type VI secretion system.

In HMM, about 130 genes were synergistically regulated by RsmA2 and RsmA3 (Figure 3A[i] and Supplementary Table 2). Specifically, both RsmA2 and RsmA3 activated 71 T3SS-related genes and inhibited 12 genes involved in alginate biosynthesis in a synergistic way (Figure 4A and Supplementary Table 4). On the other hand, about 35 genes were inversely regulated by RsmA2 and RsmA3 in HMM (Figure 3A[ii] and Supplementary Table 2). Among them, genes related with syringafactin biosynthesis (*syrR*, *syfABCD*) were up-regulated in Δ*rsmA3* versus *Pst*DC3000 but down-regulated in Δ*rsmA23* versus Δ*rsmA3*, suggesting that RsmA3 negatively and RsmA2 positively influenced syringafactin gene expression (Figure 4B and Supplementary Table 5). About 87 genes were regulated mainly by RsmA2 in HMM (Figure 3A[iii] and Supplementary Table 2). Among them, 50 genes were activated mainly by RsmA2, including the *sox* gene cluster involved in sarcosine metabolism and the *thiD* and *thiE* genes involved in thiamine biosynthesis (Figure 4C and Supplementary Table 6); whereas 37 genes were suppressed mainly by RsmA2, including genes involved in efflux pump (*mexE*, *saxF*, and *oprN*) (Figure 4C). Furthermore, about 778 genes were regulated mainly by RsmA3 in HMM, including 146 activated genes and 632 suppressed genes (Figure 3A[iv] and Supplementary Table 2). Genes involved in fatty acid metabolism, cellulose synthases (*wssABD*), and two-component regulatory system (TCRS, ColRS) were activated mainly by RsmA3, whereas genes involved in signal transduction (*spoT*, *pspto_0856*, and *dksA*), QS (*psyR* and *psyI*), c-di-GMP (*wspR*), phosphate metabolism, type VI secretion system (T6SS), and stress responses were inhibited mainly by RsmA3 (Figure 4D and Supplementary Table 7).

### Overlapping and Distinct Gene Regulation by RsmA2 and RsmA3 in KB

**FIGURE 5:**
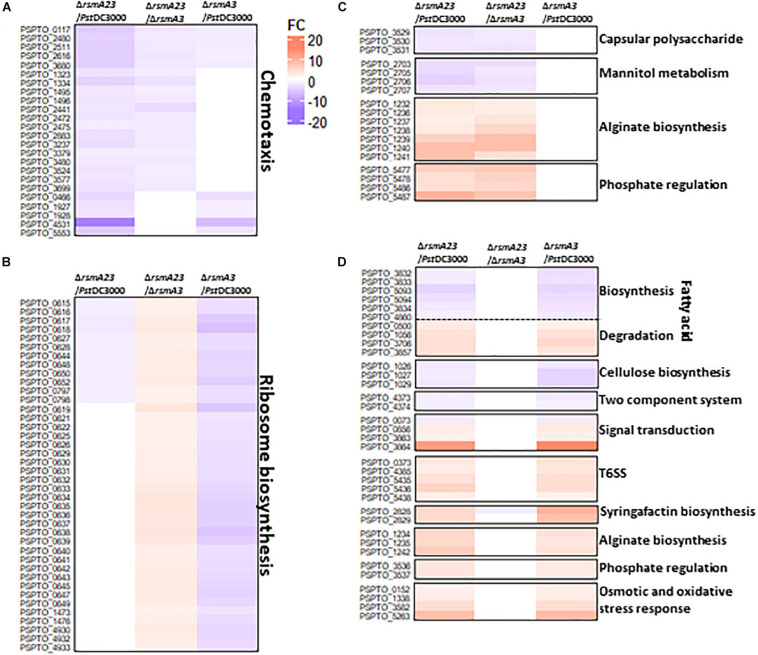
Heat maps of selected differentially expressed genes (DEGs) in KB. **(A)** Chemotaxis genes synergistically regulated by RsmA2 and RsmA3. **(B)** Genes encoding ribosome proteins inversely regulated by RsmA2 and RsmA3. **(C)** Genes regulated mainly by RsmA2. **(D)** Genes regulated mainly by RsmA3. DEGs were defined as genes with a | Fold Change (FC)| value ≥ 1.5 and a *p* value < 0.05. Up- and down-regulated DEGs were indicated by red and blue, respectively. The higher the | FC|, the deeper the color. KB: King’s B medium; *Pst*DC3000: *Pseudomonas syringae* pv. *tomato* DC3000; Δ*rsmA3*: the *rsmA3* mutant; Δ*rsmA23*: the *rsmA2/A3* double mutant; T6SS, type VI secretion system.

Similarly, about 52 genes were synergistically regulated by RsmA2 and RsmA3 in KB (Figure 3B[i] and Supplementary Table 3). Both RsmA3 and RsmA2 synergistically activated 24 chemotaxis-related genes and suppressed the transcription of alginate biosynthesis genes, including *algA*, *algL*, *algX*, *algG*, *algE*, *algK*, *alg44*, *algJ*, *algI*, *alg8*, and *algD* genes (Figures 5A,C,D and Supplementary Tables 8,10, 11), whereas 440 genes were inversely regulated by RsmA2 and RsmA3 in KB (Figure 3B[ii] and Supplementary Table 3). As an example, 40 genes encoding ribosomal proteins were down-regulated in Δ*rsmA3* versus *Pst*DC3000 but up-regulated in Δ*rsmA23* versus Δ*rsmA3*, suggesting that RsmA3 activates and RsmA2 suppresses ribosomal gene expression (Figure 5B and Supplementary Table 9). In addition, about 130 genes were regulated mainly by RsmA2 in KB, including 85 activated and 45 inhibited genes (Figure 3B[iii] and Supplementary Table 3). Among them, the *uxuB* and mannitol ABC transporter genes involved in mannitol metabolism were activated, whereas genes related with phosphate regulation were suppressed (Figure 5C and Supplementary Table 10). On the other hand, 201 and 252 genes were activated and inhibited mainly by RsmA3 in KB, respectively (Figure 3B[iv] and Supplementary Table 3). Consistent with HMM, genes encoding fatty acid metabolism-related proteins, the *wssABD* and *colRS* genes were activated mainly by RsmA3, whereas genes involved in signal transductions [(p)ppGpp and QS], T6SS, and stress responses were suppressed mainly by RsmA3 (Figure 5D and Supplementary Table 11). However, the *pslABD* genes were activated by RsmA2 and the *pslIJ* genes were inhibited by RsmA3 in KB, indicating that the capsular polysaccharide (CPS) was regulated by RsmA2 and RsmA3 differently (Figures 5C,D and Supplementary Tables 10, 11).

### Syringafactin Production and Oxidative Stress Resistance Were Negatively Regulated by RsmA3 in *Pst*DC3000

**FIGURE 6:**
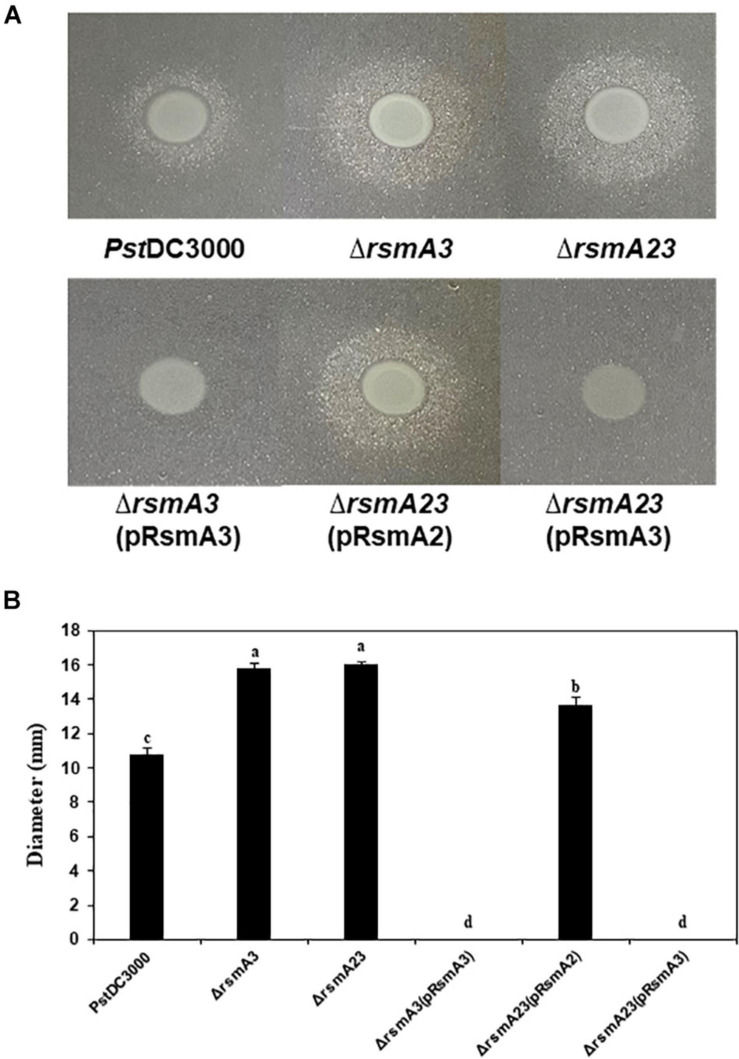
Syringafactin production in *Pst*DC3000 and its mutants and complementation stains. **(A)** Syringafactin halos around *Pst*DC3000 and its derived stains. **(B)** Diameter of syringafactin halos around *Pst*DC3000 and its derived strains. Bacteria were grown on KB plates (1.5% agar) for 24 h at 20°C before a mist of mineral oil was sprayed over. Halo diameters were measured to represent syringafactin production. Different letters in panel **(B)** indicate significant difference from one-way ANOVA followed by Fisher’s LSD test (*p* < 0.05). Error bars represented standard deviations. *Pst*DC3000: *Pseudomonas syringae* pv. *tomato* DC3000; Δ*rsmA2*: the *rsmA2* mutant; Δ*rsmA3*: the *rsmA3* mutant; Δ*rsmA23*: the *rsmA2/A3* double mutant; pRsmA2: pUCP18 containing the *rsmA2* gene with native promoter; pRsmA3: pUCP18 containing the *rsmA3* gene with native promoter.

**FIGURE 7:**
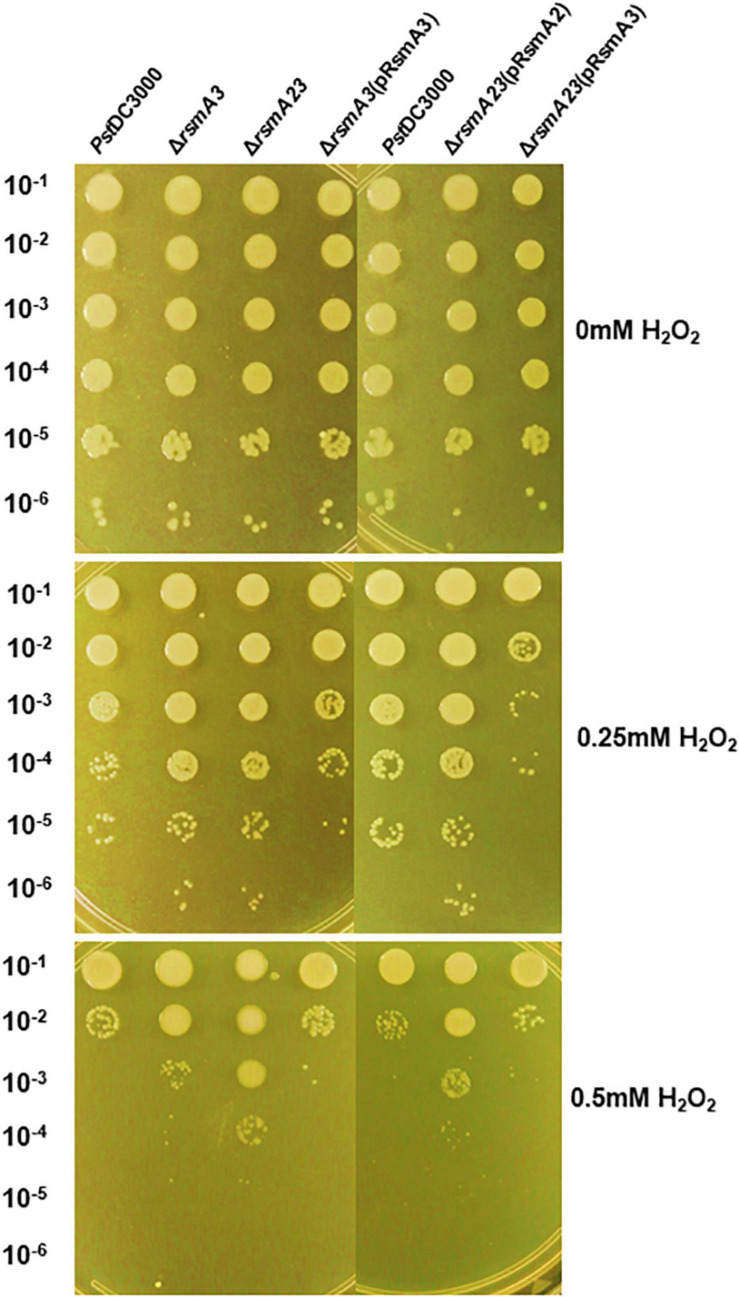
Sensitivity to oxidative stress of *Pst*DC3000 and its derived strains. Serial 10-fold dilutions were made from OD_600_ (optical density at 600 nm) = 0.1 in KB. Five microliters of each dilution were added to KB agar plates amended with 0, 0.25, and 0.5 mM H_2_O_2_. Photographs were taken 2 days post-incubation. *Pst*DC3000: *Pseudomonas syringae* pv. *tomato* DC3000; Δ*rsmA3*: the *rsmA3* mutant; Δ*rsmA23*: the *rsmA2/A3* double mutant; pRsmA2: pUCP18 containing the *rsmA2* gene with native promoter; pRsmA3: pUCP18 containing the *rsmA3* gene with native promoter.

To confirm the effect of RsmA3 on syringafactin biosynthesis in KB, atomized oil assay was used to measure syringafactin production in *Pst*DC300 and its derived *rsmA* mutant strains. Both the *rsmA3* and the *rsmA2/A3* double mutants showed significantly increased production of syringafactin as compared with the wild-type *Pst*DC3000 (Figure 6). However, expression of the *rsmA3* gene in both the *rsmA3* mutant and the *rsmA2/A3* double mutant led to almost no syringafactin production (Figure 6). These results indicated that RsmA3 strongly affected syringafactin production in a negative way in *Pst*DC3000 and RsmA2 might play a minor role. In addition, to confirm the role of RsmA3 in response to oxidative stress, spot dilution assay results showed that both the *rsmA3* and the *rsmA2/A3* double mutants exhibited increased oxidative resistance as compared with *Pst*DC3000 (Figure 7). Complementation of the mutants with the *rsmA3* gene partially restored oxidative resistance of the *rsmA3* mutant to the wild-type level (Figure 7). In addition, complementation of the *rsmA23* mutants, i.e., *rsmA23* (pRsmA2) and *rsmA23* (pRsmA3), restored to the *rsmA3* mutant and wild-type level, respectively (Figure 7). These results suggest that RsmA3 negatively regulates antioxidant stress in *Pst*DC3000 and RsmA2 might play a minor role.

## Discussion

The CsrA/RsmA RNA-binding protein is a global dual post-transcriptional regulator (Romeo et al., 2013; Vakulskas et al., 2015). Bacteria usually possess one or two homologous CsrAs/RsmAs (Pessi et al., 2001; Chatterjee et al., 2003; Ancona et al., 2016). However, five or even seven CsrAs/RsmAs are present in the *P. syringae* genome (Ge et al., 2019; Ramírez-Zapata et al., 2020; Sobrero and Valverde, 2020), and these CsrA/RsmA homologs appear to be functional. It has been further reported that CsrAs/RsmAs in *P. syringae* played redundant or unique roles, especially RsmA2 (RsmA) and RsmA3 (RsmE) in *Pst*DC3000 in regulating T3SS, alginate production, motility, and protease activities (Ferreiro et al., 2018; Ge et al., 2019; Ramírez-Zapata et al., 2020). In this study, comparative transcriptomic analysis established that RsmA proteins exhibited distinct and overlapping roles in modulating virulence and survival in *P. syringae* under different nutritional conditions and shed light on the potential interplay of the RsmA proteins. We further confirmed as how RsmA2 and RsmA3 regulated syringafactin production and oxidative stress resistance in *Pst*DC3000.

The interplay among the RsmA homologs has not been intensively investigated in plant-associated pseudomonads. In *P. fluorescens*, expression of RsmE was inhibited by both RsmA and RsmE, the later negatively regulated itself (Reimmann et al., 2005). In *Pseudomonas putida*, RsmA and RsmE negatively regulated their own expression (Huertas-Rosales et al., 2016). Furthermore, RsmA binds to *rsmA* and *rsmF* mRNA *in vitro* to repress RsmA and RsmF translation in *P. aeruginosa* (Marden et al., 2013). In this study, we found that RsmA3 positively affects the *rsmA2* gene expression and negatively influences both *rsmA1* and *rsmA5* gene expression. This is consistent with previous report that RsmA3 (RsmE) in *Pst*DC3000 promoted the expression of RsmA2 (RsmA) (Ge et al., 2019). In addition, we previously demonstrated that the RsmA2, RsmA3, and RsmA4 protein levels were much lower in *rsmA2* and *rsmA3* double mutants than in the *rsmA3* single mutant, further suggesting that RsmA2 and RsmA3 might synergistically influence the expression of RsmA2, RsmA3, and RsmA4 proteins and RsmA2 may also reciprocally influence RsmA3 expression at the post-transcriptional level (Ge et al., 2019). These results suggest that cross talk between RsmAs in *Pst*DC3000 is very complicated and RsmA3 might be on the top of the regulatory system by controlling other RsmAs at transcriptional, post-transcriptional, and translational levels. On the other hand, GacS and GacA positively control the expression of ncsRNAs (Cui et al., 2001; Chatterjee et al., 2003; Ramírez-Zapata et al., 2020), which sequester and antagonize CsrA/RsmA activities. Earlier studies identified seven ncsRNAs, i.e., *rsmX1-5*, *rsmZ*, and *rsmZ*, in the *Pst*DC3000 genome (Moll et al., 2010), and these ncsRNAs strongly bind to RsmA2 and RsmA3, but less to RsmA1 and RsmA4 (Ge et al., 2019). In this study, we showed that both RsmA2 and RsmA3 positively affect the transcription of *rsmX1*, *X2*, *X3*, *X5*, and *rsmZ* ncsRNAs, whereas the expression of *rsmY* (and possibly *rsmX4*) is positively influenced mainly by RsmA3. Similar results have also been reported in *E. amylovora* (Ancona et al., 2016). These results suggest that RsmA3 together with RsmA2 might influence the *gacS/gacA* mRNAs, thus positively regulating *rsmX/Y/Z* ncsRNAs in *Pst*DC3000 as a negative feedback loop, or directly affect the stability of ncsRNAs (Ge et al., 2019).

It has been previous reported that single *rsmA1/A2/A3/A4* mutation in *Pst*DC3000 did not affect virulence, while the *rsmA2/A3* double mutant exhibited significantly reduced virulence and bacterial growth *in planta* (Ge et al., 2019). It is thus hypothesized that genes regulated by both RsmA2 and RsmA3 might be important for bacterial growth and virulence. The T3SS is a key virulence factor in *Pst*DC3000 by suppressing host defense through injecting effectors into host cells, and the T3SS genes are directly regulated by the RpoN-HrpL sigma factor cascade (Zhao et al., 2003; Schechter et al., 2004). In *E. amylovora*, CsrA promoted the expression of the T3SS gene, which is also directly regulated by the RpoN-HrpL sigma factor cascade (Ancona et al., 2014, 2016; Lee et al., 2019). In this study, RsmA2 and RsmA3 synergistically activated 71 T3SS-related genes in HMM, including *hrpL* and *hrpR/S*, suggesting that RsmA2 and RsmA3 might target similar upstream regulatory genes to influence T3SS genes. In *X. citri*, RsmA protects the *hrpG* transcript, encoding the master regulator of T3SS, from RNase E cleavage and allows T3SS gene expression (Andrade et al., 2014). However, both HrpL and HrpS were not direct targets of CsrA in *E. amylovora*, for which the *relA* mRNA is a direct target (Lee et al., 2019). It is reasonable to speculate that RsmA2/RsmA3 might also directly target upstream regulatory genes such as *relA* mRNA to regulate T3SS in *Pst*DC3000.

Exopolysaccharides in *P. syringae* play a key role in bacterial survival and virulence under stress conditions by avoiding recognition of host plant, resisting to desiccation, and enhancing epiphytic fitness (Lindow, 1991; Kasapis et al., 1994). Alginate is a major EPS in *P. syringae* and *P. aeruginosa* (Penaloza-Vazquez et al., 1997, 2004; Ramsey and Wozniak, 2005). It has previously been reported that the *csrA3*, but not *csrA2*, mutant significantly increased the production of alginate in both LB and minimal medium and reduced the expression of the *algD* gene, the first gene in the alginate biosynthesis operon (Ferreiro et al., 2018). In contrast, we found that both RsmA2 and RsmA3 negatively influenced the expression of the alginate biosynthesis genes as reported previously (Ge et al., 2019). This discrepancy might be due to the fact that RsmA3 positively affects *rsmA2* expression, and the role of RsmA2 in alginate biosynthesis might only be evident when RsmA3 is absent. On the other hand, products of the *psl* gene cluster involved in CPS biosynthesis is essential for biofilm formation in *P. aeruginosa* (Overhage et al., 2005; Campisano et al., 2006), whereas cellulose impacts biofilm formation at the air–liquid interface (Pérez-Mendoza et al., 2014; Prada-Ramírez et al., 2016). In *Pst*DC3000, the *psl* gene cluster was suppressed mainly by RsmA3 in HMM. However, the *psl* genes in KB were activated and suppressed by RsmA2 and RsmA3, respectively. In contrast, cellulose biosynthesis genes were activated mainly by RsmA3 in both HMM and KB.

Many bacteria use flagella-driven motility to respond to changes in their chemical environment, a process known as chemotaxis (Berg and Brown, 1972; Wadhams and Armitage, 2004; Clarke et al., 2016). In *P. aeruginosa*, chemotaxis pathway-related genes play an important role in flagellar motility, T4 pili formation, and biofilm formation (Hickman et al., 2005). In *Pseudomonas savastanoi* and *Pst*DC3000, chemotaxis plays a role in virulence (Matas et al., 2012; Clarke et al., 2016) and the *rsmA2/rsmA3* mutant of *Pst*DC3000 showed significantly reduced motility (Ge et al., 2019). It is well established that CsrA binding of *flhD* inhibits RNase E-mediated cleavage and enhances its translation rate in *E. coli* (Wei et al., 2001; Yakhnin et al., 2013). In *E. amylovora*, CsrA also binds to multiple sites on the *flhD* transcript, resulting in enhanced translation (Lee et al., 2019). Here we showed that expression of the chemotaxis-related genes was positively influenced by both RsmA2 and RsmA3. It is plausible that reduced motility in the *rsmA2/rsmA3* mutant was partly due to reduced expression of chemotaxis genes and FleQ, the regulator for flagellar biosynthesis in *P. syringae* (Nogales et al., 2015), might be a direct target of RsmA2/3, which requires further investigation.

On the other hand, bacterial pathogens produce biosurfactants such as syringafactin to enhance swarming motility and acquire nutrients on leaf surfaces (Burch et al., 2014; Nogales et al., 2015). Syringafactin produced by *P. syringae* promotes bacterial proliferation by increasing the permeability of cuticle and the hygroscopicity of water to improve intake of internal nutrients (Burch et al., 2014). In this study, syringafactin biosynthesis genes were inversely influenced by RsmA3 and RsmA2, suggesting that syringafactin production is tightly regulated to fine-tune bacterial motility on leaf surface. Furthermore, the transcription factor OxyR controls the catalase-related genes (*katB*, *katE*, and *katG*) to cope with plant reactive oxidative stress (ROS) response in *Pst*DC3000 (Ishiga and Ichinose, 2016). Oxidative stress response genes including the *oxyR* gene and the *katB*, *katE*, and *katG* genes in HMM were inhibited by RsmA2 and RsmA3, respectively, whereas osmotic stress response gene *osmC* was also suppressed by RsmA3. These results suggest that RsmA proteins in *Pst*DC3000 play important roles in modulating stress responses for survival.

As a global post-transcriptional regulator in bacteria, CsrA/RsmA controls more than 10% of total genes in *Salmonella typhimurium* and *P. aeruginosa* (Lawhon et al., 2003; Burrowes et al., 2006). It was also revealed that CsrA could directly interact with about 10% of total gene transcripts in *S. typhimurium*, *Legionella pneumophila*, and *Campylobacter jejuni* (Dugar et al., 2016; Holmqvist et al., 2016; Sahr et al., 2017). In *E. coli*, about 25 and 12.5% of total gene transcripts were differentially expressed in the *csrA* mutant and directly bound by CsrA *in vivo*, respectively (Potts et al., 2017). In *E. amylovora*, CsrA affects about 20% of the genes in the genome (Lee et al., 2019). In this study, RsmA3 modulated about 20 and 27% of genes in the *Pst*DC3000 genome in HMM and KB, respectively. Considering both RmsA3 and RsmA2, the percentage of genes affected increased to 29 and 35% in HMM and KB, respectively. When all three comparisons in both conditions were considered, about half (49%) of genes in the genome was differentially expressed. It is reasonable to speculate that RsmAs in *Pst*DC3000 might directly bind to their targets or indirectly affect gene expression through global regulatory systems. Expression of global regulatory systems affected in this study includes secondary messengers (p)ppGpp, c-di-GMP, QS (Las and Rhl QS), and TCSs (GacS/GacA; ColR/ColS) (Stock et al., 2000; Miller and Bassler, 2001; Pessi et al., 2001; Kalia et al., 2013; Liu et al., 2020), suggesting that global regulatory systems might be targets for RsmA proteins.

**FIGURE 8:**
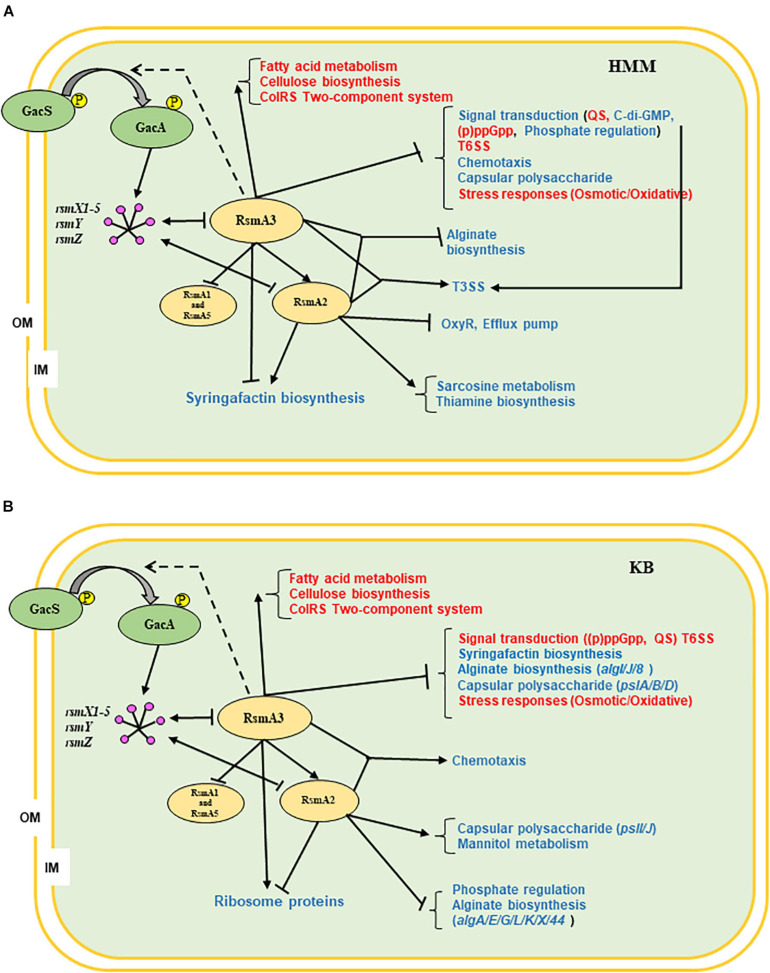
Proposed working models illustrating the global effects of RsmA2 and RsmA3 in *Pst*DC3000 as well as potential interplay among RsmA proteins. **(A)** HMM. **(B)** KB. GacS/GacA: two-component regulatory systems; *rsmX1-X5*, *rsmY*, *rsmZ*: small non-coding regulatory RNAs (sncRNAs); RsmA1, RsmA2, RsmA3, and RsmA5: RNA-binding proteins; OM: outer membrane; IM: inner membrane; ↓: positive effect; ⊥: negative effect; dashed line: unknown. Red and blue fonts represent similarity and difference between KB and HMM, respectively. HMM: *hrp*-inducing minimum medium; KB: King’s B medium; *Pst*DC3000: *Pseudomonas syringae* pv. *tomato* DC3000; (p)ppGpp: guanosine tetra/pentaphosphate; QS: quorum sensing; T3SS: type III secretion system; T6SS: type VI secretion system.

In summary, we propose the following working models for RsmA proteins in modulating gene expression in *Pst*DC3000 based on current data (Figure 8). RsmA3 activates the *rsmA2* gene expression and suppresses both *rsmA1* and *rsmA5* gene expression at transcriptional level. RsmA2 or RsmA3 in turn affected the expression levels of the *rsmY*, *rsmZ*, and *rsmX1-5* ncsRNAs. RsmA2 and RsmA3 synergistically control the expression of genes involved in T3SS and alginate biosynthesis in HMM, and chemotaxis in KB, whereas RsmA2 and RsmA3 inversely modulate transcripts of genes involved in syringafactin production in HMM, and ribosomal protein biosynthesis in KB. In addition, both RsmA2 and RsmA3 play a major role in influencing specific or conserved pathway genes in different nutritional environments as summarized in Figure 8 (red/blue texts), indicating their potential direct targets. In the future, researches should focus more on determining the impacts of the other RsmA homologs or overall impacts of all RsmAs, the precise interplay among all five RsmA homologs, and specifically the molecular targets of RsmAs in *Pst*DC3000.

## Data Availability Statement

RNA-seq data files have been submitted to Gene Expression Omnibus (GEO) at the National Center for Biotechnology Information (NCBI) with an accession number GSE162091.

## Author Contributions

YZ and BH designed the research. JL, MY, YG, and YT performed the research and analyzed the data. JL, MY, and YZ wrote the manuscript. All authors have read and approved the manuscript.

## Conflict of Interest

The authors declare that the research was conducted in the absence of any commercial or financial relationships that could be construed as a potential conflict of interest.

## Footnotes

1 https://www.ncbi.nlm.nih.gov/

2 http://eggnogdb.embl.de/#/app/emapper

## Supplementary Material

Supplementary Figure 1 Multidimensional clustering plot. Dimension 1 and Dimension 2 explained data variability of 60% (media) and 21% (strains), respectively, for strains of *Pst*DC3000, Δ*rsmA3*, and Δ*rsmA23* cultured in HMM and KB media. HMM: *hrp*-inducing minimum medium; KB: King’s B medium; *Pst*DC3000: *Pseudomonas syringae* pv. *tomato* DC3000; Δ*rsmA3*: the *rsmA3* mutant; Δ*rsmA23*: the *rsmA2/A3* double mutant.

Supplementary Figure 2 Heatmap. **(A)** *Pst*DC3000, Δ*rsmA3*, and Δ*rsmA23* in both HMM and KB, with a total of 2661 differentially expressed genes (DEGs). **(B)** *Pst*DC3000, Δ*rsmA3*, and Δ*rsmA23* in HMM with a total of 1560 DEGs. **(C)** *Pst*DC3000, *rsmA3*, and *rsmA23* in KB with a total of 1879 DEGs. Up- and down-regulated genes were indicated by red and blue colors, respectively. HMM: *hrp*-inducing minimum medium; KB: King’s B medium; *Pst*DC3000: *Pseudomonas syringae* pv. *tomato* DC3000; Δ*rsmA3*: the *rsmA3* mutant; Δ*rsmA23*: the *rsmA2/A3* double mutant.

Supplementary Figure 3 MA plots (a.k.a., mean-difference plots; M: log_2_FC; A: average log_2_CPM; CPM: counts per million reads). **(A)** Δ*rsmA23* versus *Pst*DC3000 in HMM. **(B)** Δ*rsmA23* versus Δ*rsmA3* in HMM. **(C)** Δ*rsmA3* versus *Pst*DC3000 in HMM. **(D)** Δ*rsmA23* versus *Pst*DC3000 in KB. **(E)** Δ*rsmA23* versus Δ*rsmA3* in KB. **(F)** Δ*rsmA3* versus *Pst*DC3000 in KB. M: log_2_FC, A: Average Log_2_CPM, counts per million reads. Dots between two purple lines represent | log_2_FC| value ≤ 0.58496250072 (i.e., | FC| value ≤ 1.5), and outside dots represent | log_2_FC| value ≥ 0.58496250072 (i.e., | FC| value ≥ 1.5). Up- and down-regulated genes were indicated by red and blue dot with *p* value < 0.05, black dot represents no significant difference with *p* value > 0.05. HMM: *hrp*-inducing minimum medium; KB: King’s B medium; *Pst*DC3000: *Pseudomonas syringae* pv. *tomato* DC3000; Δ*rsmA3*: the *rsmA3* mutant; Δ*rsmA23*: the *rsmA2/A3* double mutant.

Supplementary Figure 4 Functional classification of differentially expressed genes (DEGs) based on the clusters of orthologous groups (COGs). **(A)** Δ*rsmA23* versus *Pst*DC3000 in HMM. **(B)** Δ*rsmA23* versus Δ*rsmA3* in HMM. **(C)** Δ*rsmA3* versus *Pst*DC3000 in HMM. **(D)** Δ*rsmA23* versus *Pst*DC3000 in KB. **(E)** Δ*rsmA23* versus Δ*rsmA3* in KB. **(F)** Δ*rsmA3* versus *Pst*DC3000 in KB. DEGs were defined as genes with a | Fold Change (FC)| value ≥ 1.5 and a *p* value < 0.05. Up- and down-regulated DEGs were indicated by red and blue bars, respectively. HMM: *hrp*-inducing minimum medium; KB: King’s B medium; *Pst*DC3000: *Pseudomonas syringae* pv. *tomato* DC3000; Δ*rsmA3*: the *rsmA3* mutant; Δ*rsmA23*: the *rsmA2/A3* double mutant.

Supplementary Figure 5 Verification of selected differentially expressed genes (DEGs) using quantitative real-time PCR. **(A)** Expression of the *katE*, *cyaA*, *fleQ*, *phoB*, *citM*, *ladS*, and *pyk* genes in the *rsmA3* mutant and the *rsmA2/A3* double mutant as compared with *Pst*DC3000 grown in HMM. **(B)** Expression of the *katE*, *cyaA*, *fleQ*, *phoB*, *citM*, *ladS*, and *pyk* genes in the *rsmA3* mutant and the *rsmA2/A3* double mutant as compared with *Pst*DC3000 in KB. The values on top of bars represent fold changes of the gene in Δ*rsmA23* or Δ*rsmA3* versus *Pst*DC3000, while the absence of a value represents no significant difference in the RNA-seq data. HMM: *hrp*-inducing minimum medium; KB: King’s B medium; *Pst*DC3000: *Pseudomonas syringae* pv. *tomato* DC3000; Δ*rsmA3*: the *rsmA3* mutant; Δ*rsmA23*: the *rsmA2/A3* double mutant.

Supplementary Table 1 Primers for qRT-PCR used in this study.

Supplementary Table 2 List of differentially expressed genes (DEGs) in *Pst*DC3000, the *rsmA3* mutant, and the *rsmA2/A3* double mutant in HMM.

Supplementary Table 3 List of differentially expressed genes (DEGs) in *Pst*DC3000, the *rsmA3* mutant, and the *rsmA2/A3* double mutant in KB.

Supplementary Table 4 Fold changes of the type III secretion system (T3SS) and alginate biosynthesis genes in HMM.

Supplementary Table 5 Fold changes of the syringafactin biosynthesis genes in HMM.

Supplementary Table 6 Selected genes regulated mainly by RsmA2 in HMM.

Supplementary Table 7 Selected genes regulated mainly by RsmA3 in HMM.

Supplementary Table 8 Fold changes of the chemotaxis genes in KB.

Supplementary Table 9 Fold changes of the ribosome genes in KB.

Supplementary Table 10 Selected genes regulated mainly by RsmA2 in KB.

Supplementary Table 11 Selected genes regulated mainly by RsmA3 in KB.
