# Supplementary figures and images for: The RsmA RNA-Binding Proteins in Pseudomonas syringae Exhibit Distinct and Overlapping Roles in Modulating Virulence and Survival Under Different Nutritional Conditions
Source: Front Plant Sci. 2021 Feb 26;12:637595. doi: 10.3389/fpls.2021.637595 (PMC7952654; doi:10.3389/fpls.2021.637595)

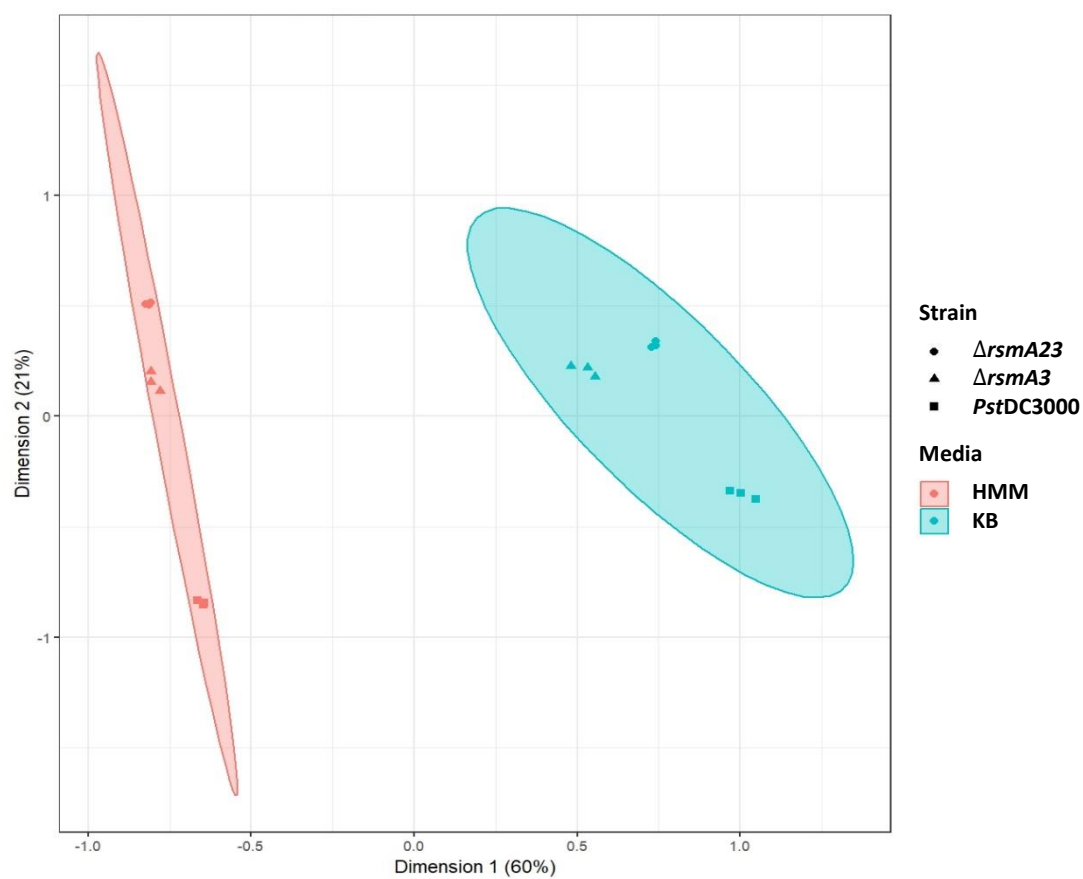

Fig. S1

A

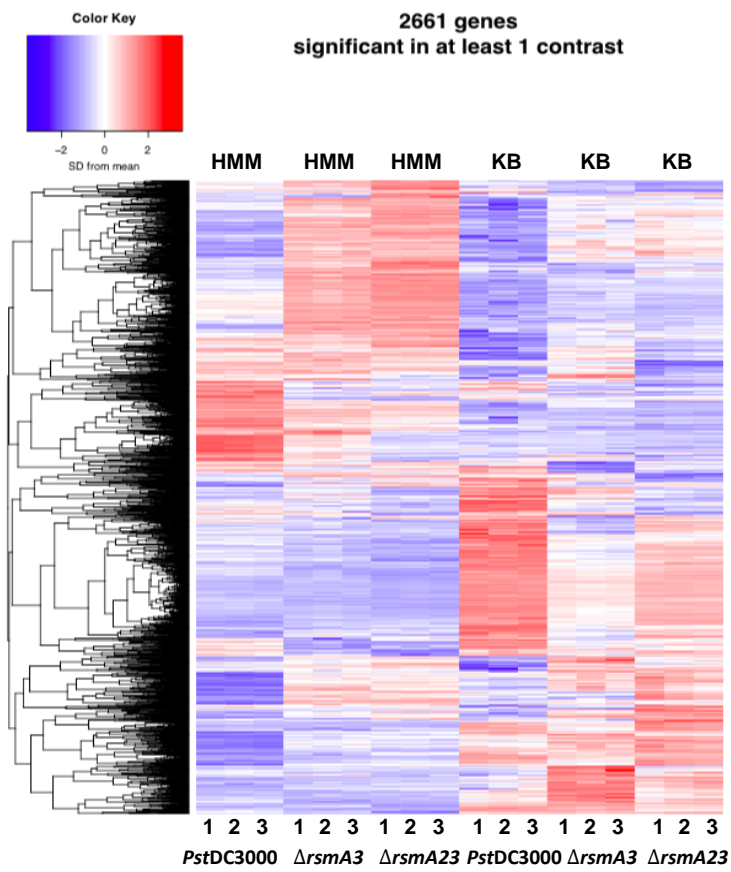

Total=2661

B

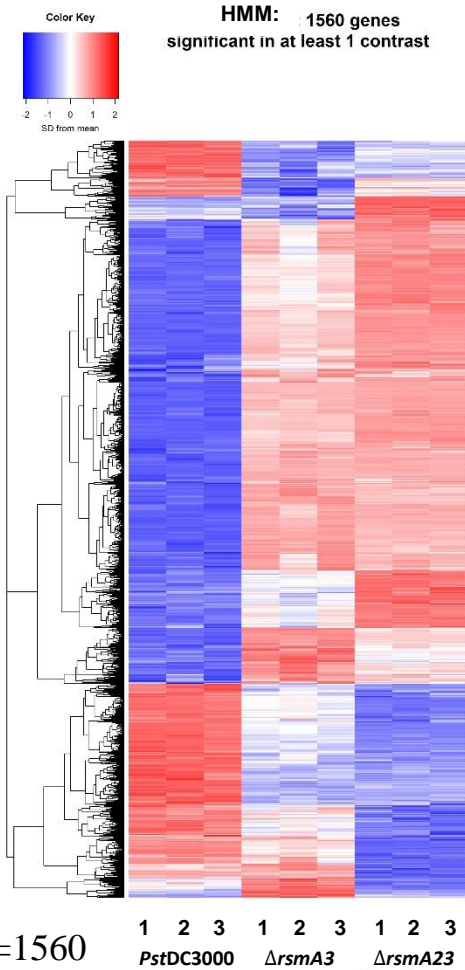

Total=1560

C

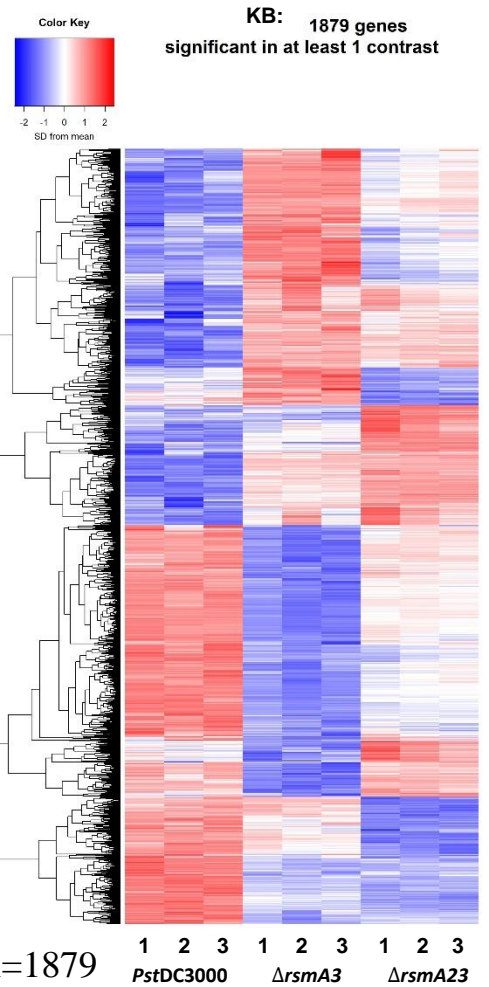

Total=1879

Fig. S2

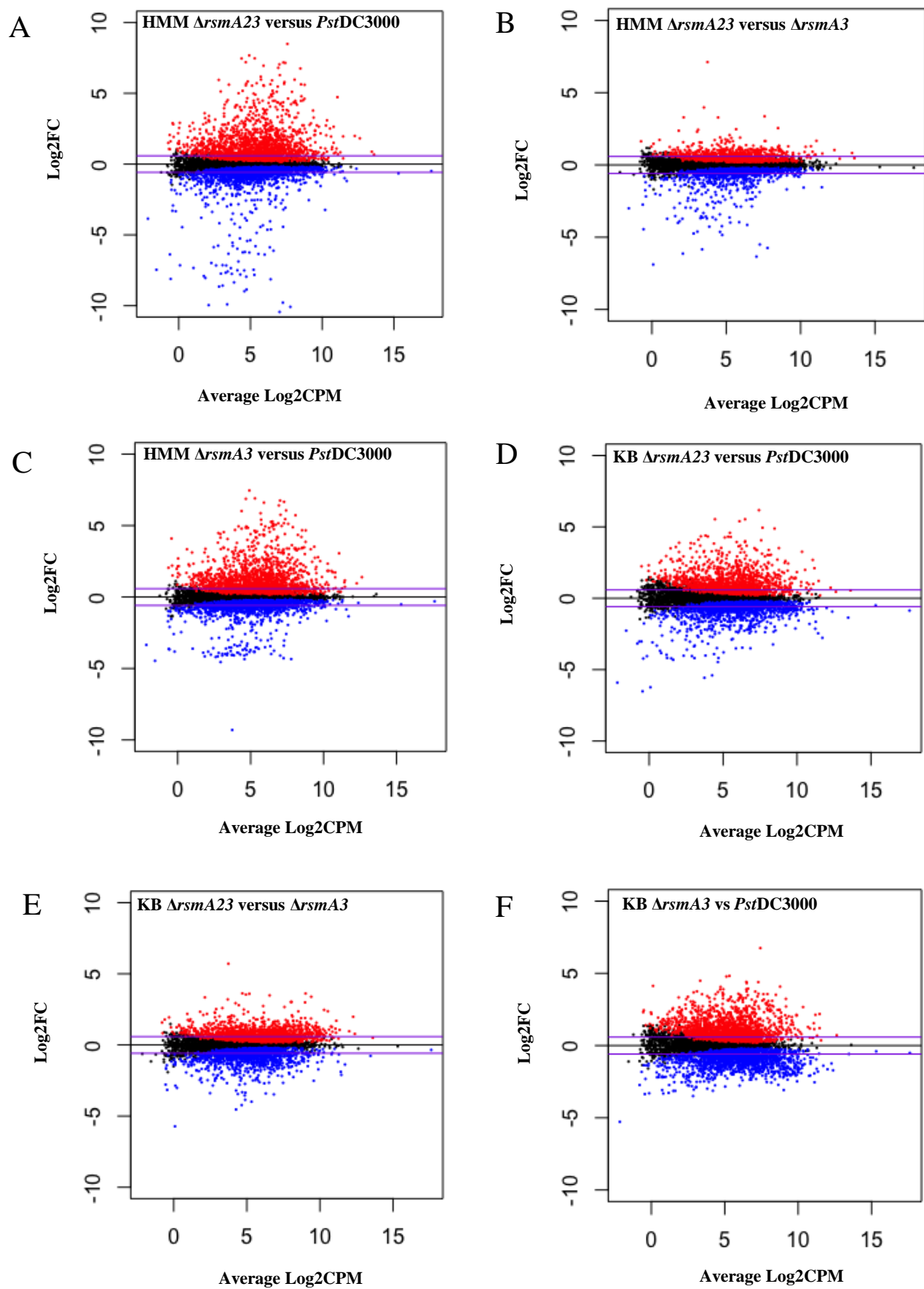

Fig. S3

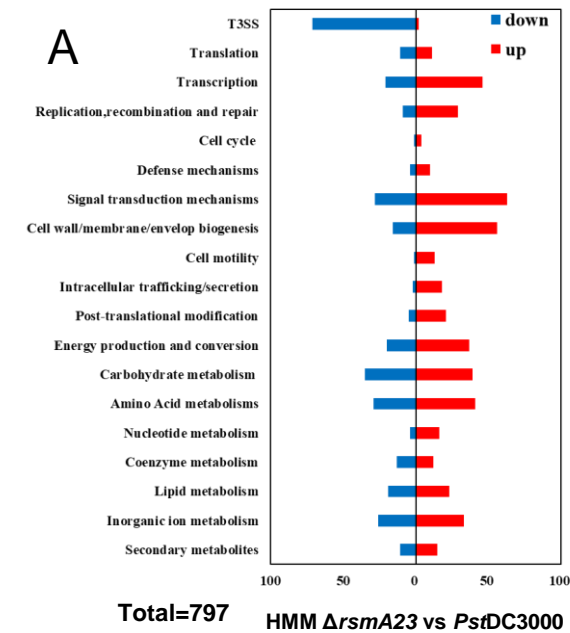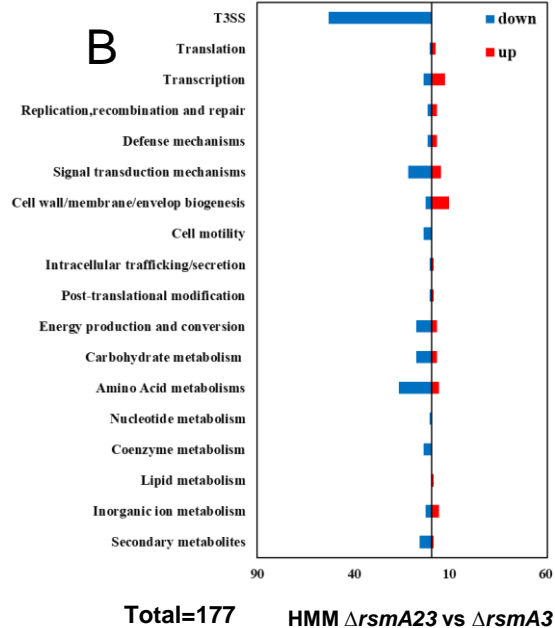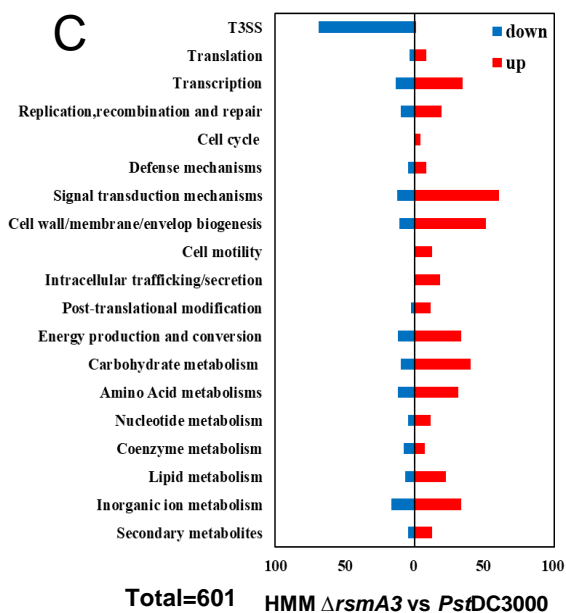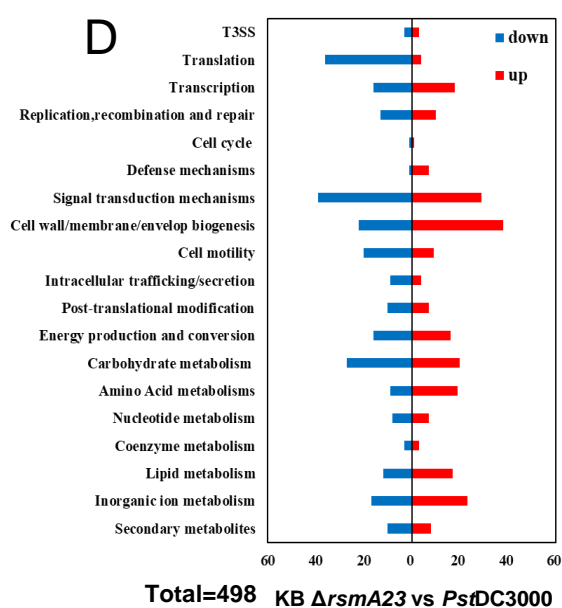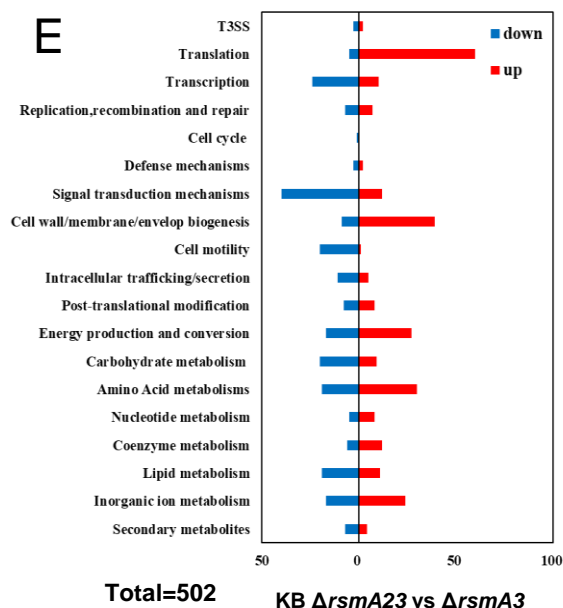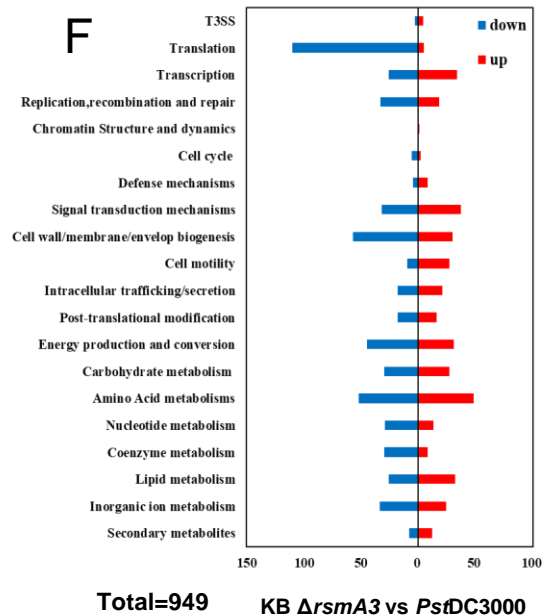

Fig. S4

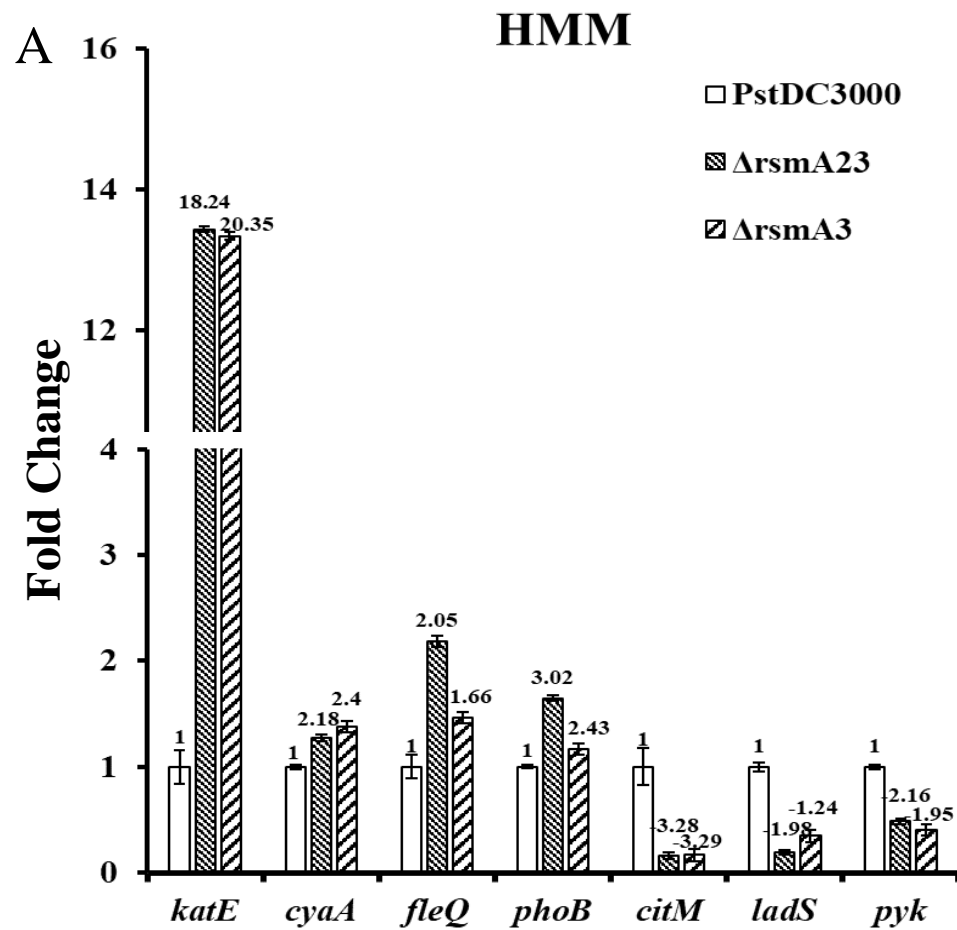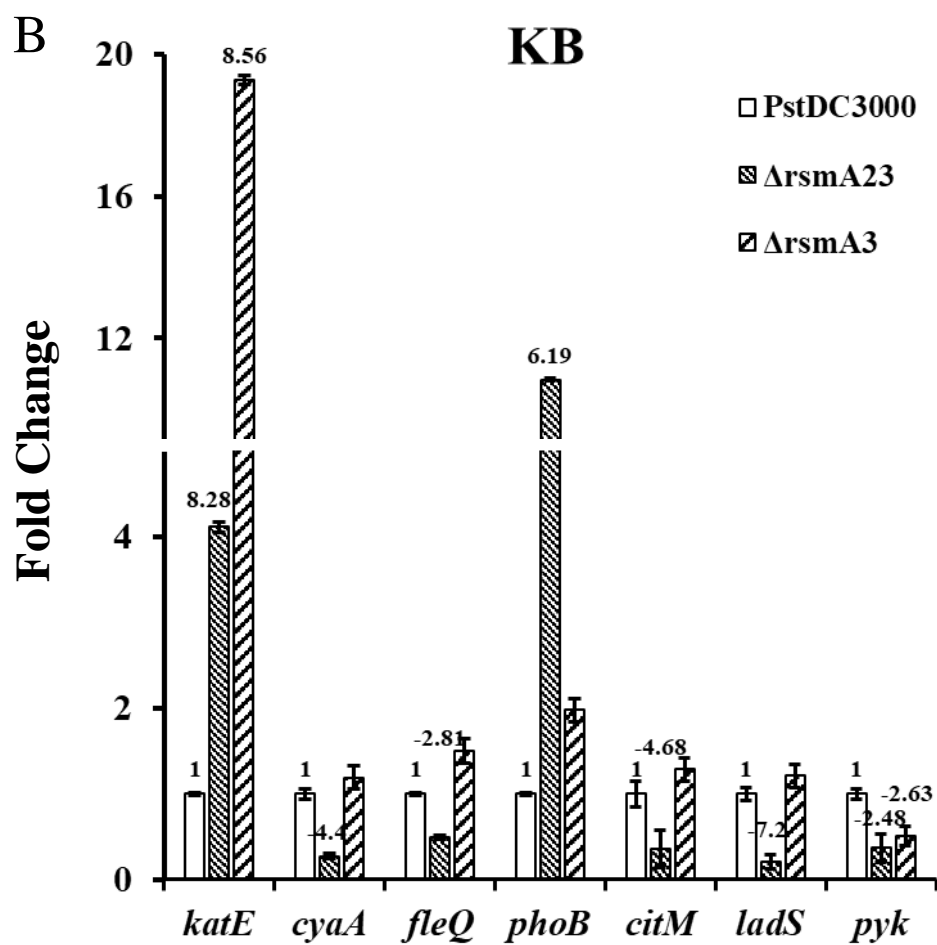

Fig. S5

Supplement: Supplementary Figure 1 — Multidimensional clustering plot. Dimension 1 and Dimension 2 explained data variability of 60% (media) and 21% (strains), respectively, for strains of PstDC3000, ΔrsmA3, and ΔrsmA23 cultured in HMM and KB media. HMM: hrp-inducing minimum medium; KB: King’s B medium; PstDC3000: Pseudomonas syringae pv. tomato DC3000; ΔrsmA3: the rsmA3 mutant; ΔrsmA23: the rsmA2/A3 double mutant. [file Data_Sheet_1.PDF]
