# Supplementary material for: The RsmA RNA-Binding Proteins in Pseudomonas syringae Exhibit Distinct and Overlapping Roles in Modulating Virulence and Survival Under Different Nutritional Conditions
Source: Front Plant Sci. 2021 Feb 26;12:637595. doi: 10.3389/fpls.2021.637595 (PMC7952654; doi:10.3389/fpls.2021.637595)
Supplement: Supplementary Table 1 — Primers for qRT-PCR used in this study. [file Data_Sheet_2.PDF]

Table S1. Primers for qRT-PCR used in this study

Primer	Sequences (5' to 3')
rpoD-rt1	GAAGGCATCCGTGAAGTGAT
rpoD-rt2	GAGAACGTCGGAGAGACGAC
katE-rt1	GTTTCGCGCAAAGTCGCCAT
katE-rt2	GTCGAACGCTACCGAAGGCA
cyaA-rt1	ACGCTGCTGCCACTGTTCTT
cyaA-rt2	GTACGAGAACGAGCGCGTCA
fleQ-rt1	GCACCGTGCCCAGGTCTATC
fleQ-rt2	TGTCGTACGTGCTGGATCGC
phoB-rt1	TCGATGGCAAACCGGCAGAA
phoB-rt2	TGTAAGCGCGCTCCTGATGG
phoR-rt1	GCATGCAGACGCTGCTCAAC
phoR-rt2	GCAGTGACCACGACCGGATT
cytM-rt1	TTCGCGGCGGGTATCTTCAC
cytM-rt2	TCCGACAGCACCGGCAATAC
ladS-rt1	GGCGCTACTTTGTGCTTGCC
ladS-rt2	TGTGCGAGGCGTACATGGTC
pyk-rt1	TCCGCTGTCCGACCACAAAG
pyk-rt2	ACGCAGCTGGCGAGCATATT

Table S2. List of differentially expressed genes (DEGs) in *Pst*DC3000, the *rsmA3* mutant, and the *rsmA23* double mutant after incubation in the *hrp*-inducing minimal medium (HMM) with |FC| value ≥1.5 and an adjusted p-value <0.05.

Locus tag	Description	Δ*rsmA23* /*Pst*DC3000	Δ*rsmA23* /Δ*rsmA3*	Δ*rsmA3* /*Pst*DC3000
**Type III secretion system**				
PSPTO_0061	type III effector HopY1	**-80.53**	**-10.03**	**-8.03**
PSPTO_0474	type III effector HopAS1, partial	**-3.52**	/	/
PSPTO_0501	type III effector HopU1	/	/	**-3.42**
PSPTO_0502	type III effector HopF2	**-15.08**	/	**-11.61**
PSPTO_0503	type III chaperone protein ShcF	**-54.35**	**-3.06**	**-17.77**
PSPTO_0588	type III effector HopH1	**-117.78**	**-7.83**	**-15.05**
PSPTO_0589	type III effector HopC1	**-75.79**	**-5.28**	**-14.35**
PSPTO_0852	type III helper protein HopAJ1	**-24.47**	**-3.26**	**-7.51**
PSPTO_0877	type III effector HopQ1-1	**-5.86**	/	**-4.24**
PSPTO_0883	type III effector HopR1	**-1.90**	**-2.99**	/
PSPTO_0901	type III effector HopAG1	**-2.37**	/	**-2.74**
PSPTO_1369	type III chaperone protein ShcN	**-38.18**	**-3.67**	**-10.39**
PSPTO_1370	type III effector HopN1	**-43.08**	**-4.51**	**-9.56**
PSPTO_1372	type III effector HopAA1-1	**-62.90**	**-5.83**	**-10.79**
PSPTO_1373	type III helper protein HrpW1	**-248.19**	**-18.86**	**-13.16**
PSPTO_1374	type III chaperone ShcM	**-142.67**	**-7.78**	**-18.33**
PSPTO_1375	type III effector HopM1	**-29.03**	**-2.51**	**-11.54**
PSPTO_1376	type III chaperone ShcE	**-4.67**	/	**-4.36**
PSPTO_1377	type III effector protein AvrE1	**-16.45**	**-2.79**	**-5.89**
PSPTO_1378	membrane-bound lytic murein transglycosylase D	**-142.88**	**-11.74**	**-12.17**
PSPTO_1379	type III transcriptional regulator HrpR	**-6.82**	**-2.11**	**-3.24**
PSPTO_1380	type III transcriptional regulator HrpS	**-7.05**	**-2.22**	**-3.18**
PSPTO_1381	type III helper protein HrpA1	**-1084.47**	**-53.60**	**-20.23**
PSPTO_1382	type III restriction system endonuclease	**-883.37**	**-45.44**	**-19.44**
PSPTO_1383	type III secretion protein HrpB	**-464.83**	**-28.76**	**-16.16**
PSPTO_1384	type III secretion protein HrcJ	**-176.38**	**-12.42**	**-14.20**
PSPTO_1385	type III secretion protein HrpD	**-181.24**	**-12.29**	**-14.75**
PSPTO_1386	type III secretion protein HrpE	**-215.99**	**-13.02**	**-16.59**
PSPTO_1387	type III secretion protein HrpF	**-402.02**	**-26.78**	**-15.01**
PSPTO_1388	type III secretion protein HrpG	**-959.23**	**-57.35**	**-16.73**
PSPTO_1389	outer-membrane type III secretion protein HrcC	**-226.35**	**-15.82**	**-14.31**
PSPTO_1390	type III secretion protein	**-10.40**	/	**-7.46**
PSPTO_1391	negative regulator of hrp expression HrpV	**-3.25**	/	**-3.37**
PSPTO_1392	type III secretion protein HrcU	**-58.37**	**-4.89**	**-11.94**
PSPTO_1393	type III secretion protein HrcT	**-161.66**	**-13.21**	**-12.24**
PSPTO_1394	type III secretion protein HrcS	**-276.60**	**-21.81**	**-12.68**
PSPTO_1395	type III secretion protein HrcR	**-126.58**	**-7.75**	**-16.34**
PSPTO_1396	type III secretion protein HrcQb	**-263.65**	**-14.84**	**-17.76**
PSPTO_1397	type III secretion protein HrcQa	**-997.41**	**-70.41**	**-14.17**
PSPTO_1398	type III secretion protein HrpP	**-384.41**	**-24.48**	**-15.70**
PSPTO_1399	type III secretion protein HrpO	**-239.83**	**-16.13**	**-14.87**
PSPTO_1400	type III secretion cytoplasmic ATPase HrcN	**-192.15**	**-12.53**	**-15.33**
PSPTO_1401	type III secretion protein HrpQ	**-327.00**	**-19.28**	**-16.96**

PSPTO_1402	type III secretion protein HrcV	**-222.59**	**-13.68**	**-16.27**
PSPTO_1403	type III secretion protein HrpJ	**-204.17**	**-12.87**	**-15.86**
PSPTO_1404	RNA polymerase sigma factor HrpL	**-504.90**	**-24.79**	**-20.37**
PSPTO_1405	type III helper protein HrpK1	**-281.56**	**-17.75**	**-15.86**
PSPTO_1406	type III effector HopB1	**-81.28**	**-6.58**	**-12.36**
PSPTO_1568	type III effector HopAF1	**-5.79**	**/**	**-3.33**
PSPTO_2678	type III helper protein HopP1	**-182.20**	**-10.69**	**-17.04**
PSPTO_3087	type III effector HopAB2	**-58.72**	**-5.41**	**-10.86**
PSPTO_4001	type III effector protein AvrPto1	**-84.14**	**-7.38**	**-11.40**
PSPTO_4101	type III helper protein HopAK1	**-55.64**	**-4.78**	**-11.65**
PSPTO_4331	type III effector HopE1	**-213.12**	**-9.08**	**-23.46**
PSPTO_4588	type III effector HopS2	**-47.00**	**-6.88**	**-6.83**
PSPTO_4589	type III chaperone ShcS2	**-14.13**	**-2.35**	**-6.02**
PSPTO_4590	type III effector HopT2	**2.24**	**/**	**/**
PSPTO_4597	type III effector HopS1	**-140.87**	**-8.24**	**-17.09**
PSPTO_4599	type III chaperone ShcS1	**-54.99**	**-3.33**	**-16.52**
PSPTO_4691	type III effector HopAD1	**-15.83**	**/**	**-10.72**
PSPTO_4703	type III effector HopAQ1	**-2.46**	**/**	**/**
PSPTO_4718	type III effector HopAA1-2	**-2.98**	**/**	**-1.83**
PSPTO_4720	type III effector HopV1	**-8.73**	**/**	**-5.70**
PSPTO_4721	type III chaperone ShcV	**-32.76**	**-2.85**	**-11.51**
PSPTO_4722	type III effector HopAO1	**-11.73**	**/**	**-8.71**
PSPTO_4724	type III effector HopD	**7.53**	**/**	**4.48**
PSPTO_4727	type III effector HopG1	**-18.60**	**/**	**-10.18**
PSPTO_4776	type III effector HopI1	**-75.24**	**-5.80**	**-12.98**
PSPTO_5353	type III chaperone protein ShcA	**-73.98**	**-6.06**	**-12.20**
PSPTO_5354	type III effector HopA1	**-69.62**	**-5.15**	**-13.53**
PSPTOA0012	type III effector HopX1	**-8.45**	**/**	**-5.99**
PSPTOA0017	type III chaperone ShcO1	**-2.70**	**/**	**-1.95**
PSPTOA0018	type III effector HopO1-1	**-3.14**	**/**	**-3.04**
PSPTOA0019	type III effector HopT1-1	**-2.73**	**/**	**-2.45**
**Translation, ribosomal structure and biogenesis**				
PSPTO_0178	methionyl-tRNA formyltransferase	**-2.00**	**/**	**/**
PSPTO_0179	sun protein	**-2.18**	**/**	**/**
PSPTO_0551	dimethyladenosine transferase	**-2.04**	**/**	**/**
PSPTO_0836	hypothetical protein PSPTO_0836	**-2.33**	**/**	**/**
PSPTO_1110	hemK protein	**/**	**/**	**1.81**
PSPTO_1268	valyl-tRNA synthetase	**2.25**	**/**	**/**
PSPTO_1413	queuine tRNA-ribosyltransferase	**-1.80**	**/**	**-1.80**
PSPTO_1842	alanyl-tRNA synthetase	**-1.84**	**/**	**/**
PSPTO_2362	methionine aminopeptidase	**2.03**	**/**	**/**
PSPTO_2569	amidase family protein	**2.11**	**/**	**/**
PSPTO_3006	deaminase AmnE	**2.01**	**/**	**/**
PSPTO_3570	GNAT family acetyltransferase	**-1.76**	**2.20**	**-3.87**
PSPTO_3840	ribosomal large subunit pseudouridine synthase C	**-1.90**	**/**	**-2.05**
PSPTO_4134	hypothetical protein PSPTO_4134	**3.23**	**/**	**4.19**
PSPTO_4158	ribonuclease T	**-1.78**	**/**	**/**
PSPTO_4183	50S ribosomal protein L31 type B	**/**	**3.06**	**/**

PSPTO_4468	ribonuclease G	**1.83**	/	/
PSPTO_4487	30S ribosomal protein S15	/	**-2.20**	**2.32**
PSPTO_4499	hypothetical protein PSPTO_4499	/	/	**-1.80**
PSPTO_4526	6-aminohexanoate-cyclic-dimer hydrolase	**-2.58**	/	/
PSPTO_4579	peptide chain release factor 3	**-2.46**	/	/
PSPTO_4654	tRNA (uracil-5-)-methyltransferase	**3.54**	/	**3.71**
PSPTO_4661	GNAT family acetyltransferase	**4.96**	/	**5.98**
PSPTO_4943	tRNA delta(2)-isopentenylpyrophosphate transferase	**1.86**	/	**1.80**
PSPTO_5559	hypothetical protein PSPTO_5559	**20.92**	/	**29.55**
PSPTO_5387	stability cassette protein	**2.93**	/	**2.33**
**Transcription**				
PSPTO_0066	oxidative stress regulatory protein OxyR	**2.20**	**2.16**	/
PSPTO_0100	leucine-responsive regulatory protein	/	**2.02**	/
PSPTO_0281	hypothetical protein PSPTO_0281	**4.30**	/	**4.14**
PSPTO_0365	AraC family transcriptional regulator	**-1.94**	/	/
PSPTO_0430	RNA polymerase sigma-32 factor	**2.13**	/	**2.04**
PSPTO_0440	regulatory protein BetI	**2.26**	**2.48**	/
PSPTO_0465	AraC family transcriptional regulator	**-7.96**	**-4.09**	**-1.95**
PSPTO_0482	hypothetical protein PSPTO_0482	**2.04**	/	/
PSPTO_0665	ROK family protein	/	/	**2.25**
PSPTO_0756	transcriptional regulator	**2.44**	/	/
PSPTO_0771	TetR family transcriptional regulator	**-2.11**	/	**-2.06**
PSPTO_0773	LysR family transcriptional regulator	**2.71**	/	**2.24**
PSPTO_0781	LysR family transcriptional regulator	**-1.86**	/	/
PSPTO_1083	peptidase, S24 family	**8.42**	/	**5.58**
PSPTO_1120	hypothetical protein PSPTO_1120	**3.87**	/	**2.70**
PSPTO_1286	RNA polymerase sigma-70 family protein	**-3.08**	/	/
PSPTO_1565	RNA polymerase sigma-38 factor	**8.52**	**2.39**	**3.57**
PSPTO_1645	MarR family transcriptional regulator	**-1.87**	/	**-2.25**
PSPTO_1666	Crp/Fnr family transcriptional regulator	**4.11**	/	**3.49**
PSPTO_1704	NAD-dependent deacetylase	**-2.13**	/	**-2.07**
PSPTO_1758	TetR family transcriptional regulator	**-5.39**	/	**-5.03**
PSPTO_1813	hypothetical protein PSPTO_1813	**-1.96**	/	/
PSPTO_1873	GntR family transcriptional regulator	**1.97**	/	/
PSPTO_1954	transcriptional regulator FleQ	**2.06**	/	/
PSPTO_1991	transcriptional activator Anr	**2.97**	/	**1.85**
PSPTO_2172	LysR family transcriptional regulator	**2.24**	/	/
PSPTO_2190	GntR family transcriptional regulator	**-2.09**	/	/
PSPTO_2279	Cys regulon transcriptional activator	**1.93**	/	/
PSPTO_2286	GntR family transcriptional regulator	**3.86**	**2.36**	/
PSPTO_2395	transcriptional regulator	**2.04**	/	/
PSPTO_2447	LysR family transcriptional regulator	**2.51**	/	**2.21**
PSPTO_2505	AraC family transcriptional regulator	**2.47**	/	**2.05**
PSPTO_2558	transcriptional regulator PhnF	/	/	**2.44**
PSPTO_2622	Sir2 family transcriptional regulator	**2.47**	/	**2.75**
PSPTO_2708	transcriptional activator MltR	**-1.83**	/	**-1.79**
PSPTO_2780	IclR family transcriptional regulator	**-2.37**	/	/
PSPTO_2804	LysR family transcriptional regulator	/	/	**-2.14**

PSPTO_2828	transcriptional regulator SyrR	**12.93**	**-2.38**	**30.74**
PSPTO_2833	LuxR family transcriptional regulator	**3.40**	/	**1.98**
PSPTO_2945	MarR family transcriptional regulator	**3.27**	/	**3.27**
PSPTO_2951	sigma-54 dependent transcriptional regulator	**-1.99**	/	**-3.08**
PSPTO_3076	TetR family transcriptional regulator	**2.05**	/	/
PSPTO_3160	transcriptional regulator	**2.11**	/	/
PSPTO_3181	transcriptional regulator	**2.32**	/	/
PSPTO_3191	DeoR family transcriptional regulator	**-2.05**	/	/
PSPTO_3207	LysR family transcriptional regulator	**3.05**	/	**2.59**
PSPTO_3244	AraC family transcriptional regulator	/	/	**2.64**
PSPTO_3355	cold shock domain family protein	**4.50**	/	**3.68**
PSPTO_3421	repressor protein c2	/	/	**-1.86**
PSPTO_3444	transcriptional activator RfaH	**2.47**	/	**2.55**
PSPTO_3510	LexA repressor	/	/	**-1.76**
PSPTO_3676	hypothetical protein PSPTO_3676	**2.10**	**2.14**	/
PSPTO_3695	TetR family transcriptional regulator	**2.96**	/	**2.56**
PSPTO_3741	sigma-54 dependent transcriptional regulator	**-2.21**	**-2.34**	/
PSPTO_3863	transcriptional regulator PsyR	**3.45**	/	**2.10**
PSPTO_4020	GNAT family acetyltransferase	**2.35**	/	**2.31**
PSPTO_4154	hypothetical protein PSPTO_4154	**3.41**	/	/
PSPTO_4192	hypothetical protein PSPTO_4192	**3.77**	/	**2.12**
PSPTO_4267	TetR family transcriptional regulator	**-2.46**	/	**-2.38**
PSPTO_4274	TetR family transcriptional regulator	/	/	**-1.97**
PSPTO_4302	TetR family transcriptional regulator	/	**3.11**	/
PSPTO_4427	AraC family transcriptional regulator	**-1.99**	/	/
PSPTO_4523	AsnC family transcriptional regulator	**-2.21**	/	**-2.25**
PSPTO_4528	AraC family transcriptional regulator	**-5.86**	**-3.47**	/
PSPTO_4601	transcription elongation factor GreB	**14.27**	/	**9.33**
PSPTO_4622	MerR family transcriptional regulator	**2.74**	/	**2.52**
PSPTO_4630	PbsX family transcriptional regulator	**10.26**	/	**6.54**
PSPTO_4644	LuxR family transcriptional regulator	**3.55**	/	/
PSPTO_4903	DNA-binding transcriptional activator OsmE	/	/	**2.68**
PSPTO_5078	malonate utilization transcriptional regulator	**-2.09**	/	**-2.02**
PSPTO_5116	RpiR family transcriptional regulator	**-2.42**	/	/
PSPTO_5146	transcriptional regulator PhaD	**22.94**	/	**16.66**
PSPTO_5176	RNA polymerase sigma-70 family protein	**16.92**	/	**10.05**
PSPTO_5177	hypothetical protein PSPTO_5177	**11.05**	/	**6.16**
PSPTO_5217	sigma-54-binding protein	**4.84**	/	**3.29**
PSPTO_5424	sigma-54 dependent transcriptional regulator	/	/	**2.33**
PSPTO_5454	GntR family transcriptional regulator	**1.99**	/	/
PSPTO_5638	hypothetical protein PSPTO_5638	**4.36**	/	**4.14**
**Replication, recombination and repair**				
PSPTO_0046	hypothetical protein PSPTO_0046	**4.14**	/	**6.79**
PSPTO_0047	UvrD/REP helicase family protein	**3.66**	/	**6.34**
PSPTO_0065	ATP-dependent DNA helicase RecG	**2.05**	/	/
PSPTO_0079	exodeoxyribonuclease III	/	/	**-1.72**
PSPTO_0086	DNA repair protein RadC	**23.82**	/	**13.59**
PSPTO_0175	DNA processing protein DprA	**4.37**	/	**3.03**

PSPTO_0183	DNA-3-methyladenine glycosidase I	**-1.96**	**-1.93**	/
PSPTO_0284	hypothetical protein PSPTO_0284	**6.39**	/	**4.89**
PSPTO_0382	NAD-dependent DNA ligase LigB	**8.91**	/	**5.94**
PSPTO_0470	exonuclease	**2.10**	/	/
PSPTO_0591	impB/mucB/samB family protein	**2.48**	/	/
PSPTO_0654	excinuclease ABC subunit A	/	/	**2.11**
PSPTO_0656	single-stranded DNA-binding protein	**1.87**	/	/
PSPTO_1121	deoxyribodipyrimidine photolyase	**2.44**	/	/
PSPTO_1253	ATP-dependent RNA helicase RhlB	**-3.10**	/	**-2.16**
PSPTO_1455	hypothetical protein PSPTO_1455	**9.15**	/	**6.36**
PSPTO_1548	ribonuclease HII	**-2.67**	**-2.26**	/
PSPTO_1644	ATP-dependent DNA helicase RecQ	**-1.88**	/	**-1.96**
PSPTO_1660	helicase/SNF2 family domain protein	**2.27**	/	/
PSPTO_1757	competence protein	**9.48**	**1.89**	**5.03**
PSPTO_2023	exonuclease	**3.74**	/	**3.54**
PSPTO_2164	excinuclease ABC subunit B	**2.62**	/	**3.46**
PSPTO_2364	endonuclease I	/	/	**-2.00**
PSPTO_2857	site-specific recombinase, phage integrase family	**-2.01**	/	/
PSPTO_2860	helicase domain-containing protein	**9.79**	/	**9.52**
PSPTO_2976	DNA topoisomerase, type I	**3.93**	/	**4.11**
PSPTO_3023	excinuclease ABC subunit C	/	/	**-1.83**
PSPTO_3464	ATP-dependent DNA ligase	**5.06**	/	**7.80**
PSPTO_3465	KU domain protein	**8.94**	/	**11.19**
PSPTO_3930	retron reverse transcriptase	/	/	**2.64**
PSPTO_4033	recA protein	**1.75**	/	/
PSPTO_4057	site-specific recombinase, phage integrase family	**2.01**	/	/
PSPTO_4058	DNA mismatch repair protein MutS	/	/	**-1.77**
PSPTO_4135	ATP-dependent DNA ligase	**2.56**	/	**3.06**
PSPTO_4141	helicase domain-containing protein	**2.21**	/	/
PSPTO_4316	exodeoxyribonuclease I	**-2.37**	/	**-2.54**
PSPTO_4382	MGMT family protein	**-2.05**	/	**-2.21**
PSPTO_4604	site-specific recombinase, phage integrase family	**2.92**	**2.03**	/
PSPTO_4744	site-specific recombinase, phage integrase family	/	/	**-1.90**
PSPTO_4745	ATP-dependent helicase HrpB	**2.17**	**3.25**	/
PSPTO_4748	site-specific recombinase, phage integrase family	**1.95**	/	/
PSPTO_5007	ATP-independent RNA helicase DbpA	**-1.85**	/	/
PSPTO_5254	radical SAM domain-containing protein	**3.33**	/	**2.54**
PSPTO_5388	bifunctional antitoxin/transcriptional repressor RelB	**2.79**	/	**2.03**
PSPTO_5472	HU family DNA-binding protein	**-2.49**	/	**-2.15**
**Cell cycle control, cell division, chromosome partitioning**				
PSPTO_0429	putative protein insertion permease FtsX	**2.59**	/	**2.38**
PSPTO_0855	ParA family protein	**2.16**	/	**2.86**
PSPTO_1026	cell morphology protein	**-3.27**	/	**-3.05**
PSPTO_1684	hypothetical protein PSPTO_1684	**2.61**	/	**2.02**
PSPTO_5387	stability cassette protein	**2.93**	/	**2.33**
**Defense mechanisms**				

PSPTO_0115	multidrug efflux protein NorA	**2.40**	/	/
PSPTO_0370	MATE efflux family protein	**-5.08**	/	**-3.10**
PSPTO_1076	O-antigen ABC transporter, permease protein	**-1.96**	/	/
PSPTO_1637	ABC transporter permease	**3.26**	/	**2.00**
PSPTO_1638	ABC transporter ATP-binding protein	**4.46**	/	**2.83**
PSPTO_2593	multidrug resistance protein, AcrA/AcrE family	**3.89**	/	/
PSPTO_2753	HlyD family secretion protein	/	/	**1.82**
PSPTO_2831	syringafactin efflux protein SyfC	**3.30**	**-5.39**	**17.77**
PSPTO_2832	syringafactin efflux protein SyfD	**2.60**	**-5.36**	**13.97**
PSPTO_2875	ABC transporter ATP-binding protein	**6.81**	/	**11.16**
PSPTO_2876	ABC transporter permease	**5.68**	/	**10.23**
PSPTO_3100	aliphatic isothiocyanate resistance protein SaxF; AcrB/AcrD/AcrF family	**3.28**	**3.00**	/
PSPTO_3703	HlyD family secretion protein	/	/	**2.09**
PSPTO_3913	hypothetical protein PSPTO_3913	**2.12**	/	/
PSPTO_4303	RND family efflux transporter MFP subunit	/	**2.17**	**-1.97**
PSPTO_4304	isothiocyanate resistance protein SaxB; isochorismatase family	/	**1.87**	**-1.94**
PSPTO_4352	hydrolase	**-2.16**	/	**-2.82**
PSPTO_4984	lipid A ABC transporter, ATP-binding/permease protein	**-1.77**	/	**-2.11**
**Signal transduction mechanisms**				
PSPTO_0064	hypothetical protein PSPTO_0064	**2.79**	/	/
PSPTO_0126	alginate biosynthesis protein AlgZ/FimS	**3.85**	/	**2.96**
PSPTO_0127	alginate biosynthesis regulatory protein AlgR	**3.05**	/	**2.84**
PSPTO_0205	hypothetical protein PSPTO_0205	**6.14**	/	**3.73**
PSPTO_0236	EAL domain-containing protein	**1.82**	/	/
PSPTO_0304	sensory box/GGDEF domain protein	**5.26**	/	**4.89**
PSPTO_0352	nitrogen regulation protein NR(I)	**-5.74**	/	**-2.39**
PSPTO_0353	nitrogen regulation protein NtrB	**-5.75**	**-2.40**	**-2.39**
PSPTO_0379	heavy metal sensor histidine kinase	/	/	**-1.76**
PSPTO_0406	sensory box/GGDEF domain/EAL domain-containing protein	**-2.36**	/	/
PSPTO_0505	diguanylate cyclase	/	**-2.41**	/
PSPTO_0536	sensory box/GGDEF domain/EAL domain-containing protein	**11.64**	/	**10.54**
PSPTO_0547	hypothetical protein PSPTO_0547	**4.50**	/	**2.64**
PSPTO_0908	protein-glutamate methylesterase CheB	**13.42**	/	**17.54**
PSPTO_0913	chemotaxis sensor histidine kinase CheA	**132.04**	/	**107.78**
PSPTO_0915	chemotaxis protein CheY	**176.81**	/	**119.59**
PSPTO_0940	tellurium resistance protein TerZ	**2.22**	/	/
PSPTO_0941	tellurium resistance protein TerA	**1.92**	/	/
PSPTO_0946	tellurium resistance protein	**2.77**	/	**2.32**
PSPTO_0969	dnaK suppressor protein	**2.13**	/	**2.11**
PSPTO_1246	PhoH-like protein	**1.98**	**2.44**	/
PSPTO_1290	DNA-binding response regulator	**-1.82**	/	/
PSPTO_1291	sensor histidine kinase	**-2.00**	/	/
PSPTO_1482	sensory box histidine kinase/response regulator	**2.58**	**2.03**	/
PSPTO_1490	sensory box histidine kinase/response regulator	**2.67**	/	**2.76**
PSPTO_1497	sensor histidine kinase/response regulator	**2.23**	/	**2.55**
PSPTO_1498	protein-glutamate methylesterase CheB	**1.99**	/	**2.12**

PSPTO_1499	response regulator/GGDEF domain-containing protein	**2.42**	/	**2.32**
PSPTO_1605	sensory box histidine kinase	**-2.08**	/	/
PSPTO_1629	carbon storage regulator	**2.49**	/	**2.32**
PSPTO_1648	aerotaxis receptor	**8.73**	/	**9.47**
PSPTO_1737	sensory box/GGDEF domain/EAL domain-containing protein	**1.94**	/	**1.98**
PSPTO_1808	hypothetical protein PSPTO_1808	**-2.16**	/	**-1.81**
PSPTO_1844	carbon storage regulator	**-20.03**	**-15.56**	/
PSPTO_1870	sensory box histidine kinase/response regulator	**2.79**	/	**3.56**
PSPTO_1911	response regulator/TPR domain protein	/	/	**2.26**
PSPTO_1963	STAS domain-containing protein	**6.06**	/	**4.06**
PSPTO_1964	response regulator	**3.93**	/	**3.16**
PSPTO_1965	Hpt domain-containing protein	**2.08**	/	/
PSPTO_2014	aerotaxis receptor	/	**-2.42**	**2.63**
PSPTO_2117	response regulator	/	**-1.80**	/
PSPTO_2128	response regulator	**-3.28**	/	**-2.95**
PSPTO_2129	sensory box histidine kinase/response regulator	**-2.90**	/	**-2.32**
PSPTO_2130	LuxR family DNA-binding response regulator	**-4.34**	/	**-2.89**
PSPTO_2171	sensory box/GGDEF domain/EAL domain-containing protein	**3.53**	/	**2.89**
PSPTO_2212	sensor histidine kinase/response regulator	**2.57**	/	**2.85**
PSPTO_2245	sensor protein KdpD	**5.62**	/	**3.87**
PSPTO_2246	KDP operon transcriptional regulatory protein kdpE	**2.21**	/	/
PSPTO_2259	sigma-54 dependent transcriptional regulator	**3.59**	/	**3.22**
PSPTO_2278	universal stress protein family	**5.51**	/	**3.98**
PSPTO_2326	extracellular solute-binding protein/sensory box protein, partial	**1.75**	/	/
PSPTO_2479	hypothetical protein PSPTO_2479	**11.80**	/	**8.11**
PSPTO_2591	diguanylate cyclase	**-1.95**	**-2.87**	/
PSPTO_2717	sensory box histidine kinase/response regulator	/	/	**2.58**
PSPTO_2718	hypothetical protein PSPTO_2718	**2.66**	/	**2.66**
PSPTO_2757	GGDEF domain/EAL domain protein	**-2.06**	/	/
PSPTO_2867	TspO/MBR family protein	**2.81**	/	**2.42**
PSPTO_2896	sensory box histidine kinase/response regulator	**1.94**	/	**2.26**
PSPTO_2907	GGDEF domain/EAL domain protein	**2.11**	/	**2.80**
PSPTO_3111	sensory box histidine kinase	**10.60**	**1.90**	**5.58**
PSPTO_3246	hypothetical protein PSPTO_3246	**2.20**	/	/
PSPTO_3566	carbon storage regulator	**-4.55**	**138.93**	**-631.64**
PSPTO_3696	sensory box histidine kinase/response regulator	**1.95**	/	**2.29**
PSPTO_3699	methyl-accepting chemotaxis protein	**-1.97**	/	/
PSPTO_4079	sensor histidine kinase/response regulator	**-2.85**	/	**-2.21**
PSPTO_4080	LuxR family DNA-binding response regulator	**-2.98**	/	**-2.42**
PSPTO_4106	GGDEF domain/EAL domain protein	**11.12**	/	**11.52**
PSPTO_4151	LuxR family DNA-binding response regulator	**5.65**	/	**4.56**
PSPTO_4208	HAMP domain/GGDEF domain/EAL domain protein	/	/	**2.13**
PSPTO_4292	sigma-54 dependent transcriptional regulator/response regulator	**-2.13**	/	/
PSPTO_4293	sensory box histidine kinase/response regulator	**-1.99**	/	/
PSPTO_4371	inaA protein	**-7.36**	**-1.93**	**-3.80**
PSPTO_4373	sensor histidine kinase ColS	**-2.00**	/	/

PSPTO_4374	DNA-binding response regulator ColR	**-1.91**	/	**-1.86**
PSPTO_4531	methyl-accepting chemotaxis protein	**-2.53**	/	/
PSPTO_4543	GAF domain/GGDEF domain/EAL domain protein	**-2.02**	**-5.16**	**2.55**
PSPTO_4631	sensory box/GGDEF domain/EAL domain-containing protein	**3.74**	/	**3.04**
PSPTO_4784	diguanylate cyclase	**5.32**	**2.35**	**2.26**
PSPTO_4796	sensor histidine kinase	**-1.98**	/	/
PSPTO_4833	sensory box histidine kinase	**30.46**	/	**30.60**
PSPTO_4836	DNA binding response regulator, LuxR family	**34.55**	/	**18.61**
PSPTO_4837	response regulator	**-3.03**	**-3.87**	/
PSPTO_4868	sensor histidine kinase/response regulator RetS	**3.80**	/	**3.34**
PSPTO_5014	response regulator/sensory box/GGDEF domain/EAL domain protein	**18.71**	/	**12.39**
PSPTO_5284	phosphoenolpyruvate-protein phosphotransferase PtsP	**-2.24**	/	/
PSPTO_5417	serine/threonine phosphoprotein phosphatase	/	/	**2.50**
PSPTO_5422	FHA domain-containing protein	/	/	**1.91**
PSPTO_5477	phosphate regulon transcriptional regulatory protein PhoB	**3.02**	/	**2.43**
PSPTO_5478	phosphate regulon sensor protein phoR	**2.05**	/	**1.81**
PSPTO_5482	response regulator	/	**-2.90**	/
PSPTO_5569	methyl-accepting chemotaxis protein	**1.96**	/	/
PSPTO_5573	sensor histidine kinase	**5.49**	/	**3.56**
PSPTO_5583	Ais protein	/	/	**2.45**
PSPTO_0466	methyl-accepting chemotaxis protein	/	/	**-2.30**
PSPTO_0909	chemoreceptor glutamine deamidase CheD	**32.07**	/	**30.81**
PSPTO_0910	chemotaxis protein methyltransferase CheR	**123.62**	/	**97.38**
PSPTO_0911	chemotaxis protein CheW	**204.42**	/	**175.37**
PSPTO_0912	methyl-accepting chemotaxis protein	**144.44**	/	**101.24**
PSPTO_1494	chemotaxis protein CheW	**2.93**	/	**2.90**
PSPTO_1495	chemotaxis protein methyltransferase CheR	**2.76**	/	**2.94**
PSPTO_1496	chemotaxis protein CheW	**2.84**	/	**3.25**
PSPTO_1928	chemotaxis protein methyltransferase CheR	**2.59**	/	**2.81**
PSPTO_3237	methyl-accepting chemotaxis protein	/	**-2.44**	/
PSPTO_3524	chemotaxis protein CheV	**-1.95**	**-2.63**	/
**Cell wall/membrane/envelope biogenesis**				
PSPTO_0373	Rhs family protein	/	/	**2.87**
PSPTO_0377	metal ion efflux outer membrane protein	**2.80**	/	/
PSPTO_0674	tonB domain protein	**7.56**	/	**10.38**
PSPTO_1005	GDP-mannose 4,6-dehydratase	**8.34**	/	**6.45**
PSPTO_1027	cellulose synthase, catalytic subunit	/	/	**-1.96**
PSPTO_1053	hypothetical protein PSPTO_1053	**2.09**	/	**2.12**
PSPTO_1116	cyclopropane-fatty-acyl-phospholipid synthase	**3.10**	/	**2.34**
PSPTO_1231	insecticidal toxin protein	**2.71**	/	**2.22**
PSPTO_1232	alginate biosynthesis protein AlgA	**27.60**	**2.51**	**11.00**
PSPTO_1233	alginate biosynthesis protein AlgF	**24.71**	**3.12**	**7.91**
PSPTO_1234	alginate biosynthesis protein AlgJ	**22.26**	**2.13**	**10.47**
PSPTO_1235	alginate biosynthesis protein AlgI	**20.27**	/	**10.00**
PSPTO_1237	alginate biosynthesis protein AlgX	**19.92**	/	**9.62**
PSPTO_1238	alginate biosynthesis protein AlgG	**22.10**	/	**10.62**
PSPTO_1239	alginate biosynthesis protein AlgE	**20.37**	/	**10.04**

PSPTO_1240	alginate biosynthesis protein AlgK	**20.41**	/	**10.69**
PSPTO_1241	alginate biosynthesis protein Alg44	**22.14**	/	**10.47**
PSPTO_1242	alginate biosynthesis protein Alg8	**22.67**	**2.04**	**11.12**
PSPTO_1243	GDP-mannose 6-dehydrogenase AlgD	**26.46**	**3.18**	**8.33**
PSPTO_1296	porin B	**-2.69**	/	**-1.97**
PSPTO_1445	peptidase, M23/M37 family	**-2.27**	/	/
PSPTO_1506	ompA family protein	**6.22**	/	**3.30**
PSPTO_1547	lipid A disaccharide synthase	**-2.52**	**-2.27**	/
PSPTO_1564	lipoprotein NlpD	**2.07**	/	/
PSPTO_1632	GNAT family acetyltransferase	**2.44**	/	**2.51**
PSPTO_1705	NLP/P60 family protein	/	/	**-2.66**
PSPTO_2297	membrane protein	**3.10**	/	**1.97**
PSPTO_2343	outer membrane porin, OprD family	**-3.00**	/	**-2.39**
PSPTO_2354	glycosyl transferase family protein	**4.14**	/	**3.72**
PSPTO_2756	outer membrane efflux protein	**2.07**	/	**2.53**
PSPTO_2767	lipopolysaccharide core biosynthesis domain protein	/	**2.12**	/
PSPTO_2899	hypothetical protein PSPTO_2899	**4.86**	/	**5.60**
PSPTO_2946	outer membrane porin, OprD family	**-3.31**	/	**-3.78**
PSPTO_3099	multidrug efflux RND membrane fusion protein MexE	**3.21**	**2.95**	/
PSPTO_3101	outer membrane efflux protein	**3.89**	**3.30**	/
PSPTO_3134	glycosyl transferase family protein	**4.48**	/	**4.85**
PSPTO_3188	choline/ethanolamine kinase	**-2.18**	/	/
PSPTO_3247	mandelate racemase/muconate lactonizing protein	/	**1.98**	**-1.99**
PSPTO_3285	glucarate dehydratase	**-2.10**	/	**-2.17**
PSPTO_3290	outer membrane porin, OprD family	**5.28**	/	**3.31**
PSPTO_3447	glycoside hydrolase family protein	**4.45**	/	**3.01**
PSPTO_3450	capsular polysaccharide biosynthesis protein	**4.37**	/	**3.72**
PSPTO_3482	Rhs element Vgr protein	**2.10**	/	**1.92**
PSPTO_3529	capsular polysaccharide biosynthesis protein PslA	**44.49**	/	**35.66**
PSPTO_3530	mannose-1-phosphate guanylyltransferase/mannose-6-phosphate isomerase PslB	**34.38**	/	**29.57**
PSPTO_3531	lipoprotein PslD	**49.77**	/	**38.29**
PSPTO_3532	exopolysaccharide biosynthesis protein PslE	**55.35**	/	**43.74**
PSPTO_3533	glycosyl transferase, group 1 family protein PslF	**36.21**	/	**34.33**
PSPTO_3535	glycosyl transferase, group 1 family protein PslH	**25.99**	/	**23.63**
PSPTO_3536	glycosyl transferase, group 1 family protein PslI	**31.85**	/	**26.82**
PSPTO_3537	membrane protein PslJ	**22.25**	/	**20.75**
PSPTO_3648	acid phosphatase	**4.43**	/	**3.96**
PSPTO_3715	membrane-bound lytic murein transglycosylase D	**-1.84**	/	/
PSPTO_3849	Rhs element Vgr protein	**2.09**	/	/
PSPTO_3987	porin D	**-9.42**	**-2.96**	**-3.18**
PSPTO_4062	mandelate racemase/muconate lactonizing protein	**3.05**	/	**3.05**
PSPTO_4122	nucleoside-specific channel-forming protein	**-2.34**	/	/
PSPTO_4191	hypothetical protein PSPTO_4191	**10.75**	/	**6.42**
PSPTO_4305	outer membrane efflux protein	/	/	**-2.37**

PSPTO_4340	insecticidal toxin protein	/	/	**1.94**
PSPTO_4385	Rhs element Vgr protein	**2.42**	/	**2.17**
PSPTO_4471	rod shape-determining protein MreC	**-2.00**	/	/
PSPTO_4641	large conductance mechanosensitive channel protein	**5.96**	/	**3.70**
PSPTO_4817	HopAJ2 protein	/	/	**-1.80**
PSPTO_4907	hypothetical protein PSPTO_4907	**2.25**	/	/
PSPTO_4991	glycoside hydrolase family protein	**2.27**	/	**2.48**
PSPTO_5036	tonB domain protein	**2.22**	/	/
PSPTO_5097	glycosyl transferase family protein	**-4.13**	/	/
PSPTO_5101	hypothetical protein PSPTO_5101	**-2.32**	/	/
PSPTO_5170	lipoprotein Blc	**1.93**	/	**2.12**
PSPTO_5283	prolipoprotein diacylglyceryl transferase	**-2.72**	**-2.53**	/
PSPTO_5391	outer membrane porin, OprD family	**9.88**	/	**6.03**
PSPTO_5415	Rhs element Vgr protein	**1.75**	/	**1.99**
PSPTO_5418	hypothetical protein PSPTO_5418	**1.79**	/	**2.19**
PSPTO_5436	Rhs element Vgr protein	**6.38**	/	**7.00**
PSPTO_5438	Rhs family protein	**3.75**	/	**4.11**
PSPTO_0732	hypothetical protein PSPTO_0732	**4.01**	/	**2.74**
PSPTO_3079	hypothetical protein PSPTO_3079	**4.83**	/	**3.13**
PSPTO_4360	dehydrogenase	**-1.85**	/	/
PSPTO_4549	5-dehydro-4-deoxyglucarate dehydratase	**-2.29**	/	**-1.91**
**Cell motility**				
PSPTO_1939	flagellar basal body rod protein FlgF	**2.33**	/	/
PSPTO_1940	flagellar basal-body rod protein FlgG	**2.23**	/	/
PSPTO_1945	flagellar hook-associated protein FlgL	**1.84**	/	/
PSPTO_1949	flagellin	/	**-2.91**	**2.04**
PSPTO_2526	methyl-accepting chemotaxis protein	**2.88**	/	**5.74**
PSPTO_4156	sodium-type flagellar protein MotY	/	**-2.36**	/
PSPTO_0466	methyl-accepting chemotaxis protein	/	/	**-2.30**
PSPTO_0909	chemoreceptor glutamine deamidase CheD	**32.07**	/	**30.81**
PSPTO_0910	chemotaxis protein methyltransferase CheR	**123.62**	/	**97.38**
PSPTO_0911	chemotaxis protein CheW	**204.42**	/	**175.37**
PSPTO_0912	methyl-accepting chemotaxis protein	**144.44**	/	**101.24**
PSPTO_1494	chemotaxis protein CheW	**2.93**	/	**2.90**
PSPTO_1495	chemotaxis protein methyltransferase CheR	**2.76**	/	**2.94**
PSPTO_1496	chemotaxis protein CheW	**2.84**	/	**3.25**
PSPTO_1928	chemotaxis protein methyltransferase CheR	**2.59**	/	**2.81**
PSPTO_3237	methyl-accepting chemotaxis protein	/	**-2.44**	/
PSPTO_3524	chemotaxis protein CheV	**-1.95**	**-2.63**	/
PSPTO_1313	pili assembly chaperone	/	/	**2.47**
PSPTO_3033	twitching motility protein	**4.11**	/	**2.94**
**Intracellular trafficking, secretion, and vesicular transport**				
PSPTO_0051	hypothetical protein PSPTO_0051	**2.21**	/	/
PSPTO_0319	type IV pilus biogenesis protein	**2.82**	/	**2.82**
PSPTO_0531	type IV secretion system protein	**4.20**	/	**3.12**
PSPTO_0672	TonB system transport protein ExbD	/	/	**4.40**
PSPTO_0673	TonB system transport protein ExbB	/	/	**3.91**
PSPTO_0810	type IV pilus biogenesis protein	**2.60**	/	/

PSPTO_0878	hypothetical protein PSPTO_0878	**2.38**	/	**2.00**
PSPTO_0927	type IV pilus biogenesis protein	**2.83**	/	**2.61**
PSPTO_2482	TonB system transport protein ExbB	**2.59**	/	/
PSPTO_2483	TonB system transport protein ExbD	**3.18**	/	**3.01**
PSPTO_3230	hemolysin activator protein, HlyB family	**-5.96**	**-3.27**	/
PSPTO_4834	hypothetical protein PSPTO_4834	**25.24**	/	**16.01**
PSPTO_4850	hypothetical protein PSPTO_4850	**45.98**	**2.25**	**20.40**
PSPTO_4851	type II/III secretion system protein	**62.62**	/	**33.89**
PSPTO_4852	hypothetical protein PSPTO_4852	**71.96**	/	**42.50**
PSPTO_4853	type II/IV secretion system protein	**60.15**	/	**35.14**
PSPTO_4854	hypothetical protein PSPTO_4854	**51.49**	/	**30.59**
PSPTO_4855	membrane protein	**47.10**	/	**28.75**
PSPTO_4856	TPR domain-containing protein	**47.41**	/	**25.92**
PSPTO_5157	sec-independent protein translocase TatC	**-1.81**	/	/
PSPTO_1313	pili assembly chaperone	/	/	**2.47**
PSPTO_3033	twitching motility protein	**4.11**	/	**2.94**
PSPTO_4835	hypothetical protein PSPTO_4835	**34.97**	/	**21.19**
**Posttranslational modification, protein turnover, chaperones**				
PSPTO_0119	peptidase, M16 family	**2.05**	/	**2.37**
PSPTO_0152	osmotically inducible protein	**4.81**	/	**3.77**
PSPTO_0341	thiol:disulfide interchange protein DsbA	**2.08**	/	/
PSPTO_0540	O-sialoglycoprotein endopeptidase	**1.99**	/	/
PSPTO_0597	OsmC/Ohr family protein	/	/	**-1.73**
PSPTO_1324	disulfide oxidoreductase	**6.67**	/	**3.45**
PSPTO_1649	autotransporter	**7.08**	/	**10.00**
PSPTO_1767	organic hydroperoxide resistance protein	**-3.28**	/	/
PSPTO_1778	heat shock protein HtpX	/	**2.04**	/
PSPTO_2408	urease accessory protein UreD	**3.06**	/	**3.03**
PSPTO_2548	clpB protein	**2.17**	/	/
PSPTO_2657	xanthine dehydrogenase accessory factor XdhC	**2.43**	/	**4.94**
PSPTO_3107	alkyl hydroperoxide reductase subunit F	**-2.43**	/	/
PSPTO_3108	alkyl hydroperoxide reductase	**-2.00**	/	**-1.83**
PSPTO_3112	glutathione reductase	**1.70**	/	/
PSPTO_3343	glutathione S-transferase domain protein	**2.54**	/	**2.86**
PSPTO_3351	hypothetical protein PSPTO_3351	**2.33**	/	/
PSPTO_3353	ATP-dependent clp protease, ATP-binding subunit ClpA	**2.14**	/	/
PSPTO_3629	thiol:disulfide interchange protein DsbE	**-2.25**	/	/
PSPTO_3630	cytochrome c-type biogenesis protein CcmF	**-1.96**	/	/
PSPTO_3744	peptidyl-prolyl cis-trans isomerase B	/	/	**-2.18**
PSPTO_3856	glutathione S-transferase family protein	**1.86**	/	/
PSPTO_4640	DNA repair protein RadA	**2.25**	/	/
PSPTO_4858	thiol:disulfide interchange protein DsbD	**2.36**	/	/
PSPTO_5258	cytochrome c oxidase assembly protein	**6.92**	/	**4.96**
PSPTO_5317	antioxidant, AhpC/Tsa family	**5.38**	/	**6.39**
PSPTO_5363	DnaJ domain-containing protein	/	**-2.52**	/
PSPTO_5425	clpB protein	**1.95**	/	**2.36**
PSPTO_5535	hypothetical protein PSPTO_5535	**1.78**	/	/
PSPTO_4835	hypothetical protein PSPTO_4835	**34.97**	/	**21.19**

**Energy production and conversion**				
PSPTO_0062	citrate transporter	**-3.28**	/	**-3.29**
PSPTO_0092	aldehyde dehydrogenase family protein	**-1.86**	/	/
PSPTO_0107	hypothetical protein PSPTO_0107	**3.43**	/	**3.05**
PSPTO_0173	quinone oxidoreductase	**3.55**	/	**3.16**
PSPTO_0181	molybdopterin oxidoreductase subunit alpha	**4.13**	/	**6.07**
PSPTO_0396	NADH:flavin oxidoreductase/NADH oxidase family protein	**-4.31**	**-6.59**	/
PSPTO_0397	iron-sulfur cluster-binding protein	**-2.25**	**-2.39**	/
PSPTO_0398	electron transfer flavoprotein subunit alpha	**-2.97**	**-2.78**	/
PSPTO_0402	iron-sulfur cluster-binding protein	/	/	**2.13**
PSPTO_0441	betaine aldehyde dehydrogenase BADH	**2.34**	/	**2.08**
PSPTO_0455	glutathione-independent formaldehyde dehydrogenase	**5.74**	/	**5.65**
PSPTO_0480	malate synthase G	**-1.92**	/	**-1.97**
PSPTO_0775	methylmalonate-semialdehyde dehydrogenase	**3.01**	/	**2.62**
PSPTO_0782	methylmalonate-semialdehyde dehydrogenase	**3.80**	/	**2.42**
PSPTO_0834	alcohol dehydrogenase	**-3.55**	/	**-2.68**
PSPTO_0917	NADH dehydrogenase	**1.84**	/	**1.90**
PSPTO_0986	membrane protein	**2.45**	/	/
PSPTO_1215	D-isomer specific 2-hydroxyacid dehydrogenase family protein	/	/	**-2.57**
PSPTO_1489	xenobiotic reductase	**2.66**	/	**2.79**
PSPTO_1558	alcohol dehydrogenase, class III	**2.70**	/	**2.62**
PSPTO_1731	class II fumarate hydratase	**3.12**	/	**3.25**
PSPTO_1795	alkanesulfonate monooxygenase	/	**-2.81**	**2.20**
PSPTO_1875	glutathione-dependent formaldehyde dehydrogenase	**17.69**	/	**24.93**
PSPTO_2016	aconitate hydratase 1	**2.06**	/	**2.00**
PSPTO_2047	HAD-superfamily hydrolase	**-2.01**	/	/
PSPTO_2106	soluble pyridine nucleotide transhydrogenase	**-2.27**	/	**-2.00**
PSPTO_2405	xenobiotic reductase A	/	**1.86**	/
PSPTO_2491	oxidoreductase, aldo/keto reductase family	**-2.19**	/	/
PSPTO_2510	pyruvate dehydrogenase	**3.59**	/	**4.49**
PSPTO_2655	oxidoreductase, molybdopterin-binding subunit	**3.43**	/	**6.22**
PSPTO_2656	aldehyde oxidase and xanthine dehydrogenase family protein	**2.71**	/	**4.61**
PSPTO_2676	glutathione-independent formaldehyde dehydrogenase	**27.62**	/	**39.04**
PSPTO_2680	succinate-semialdehyde dehydrogenase	**2.49**	/	/
PSPTO_2697	oxidoreductase zinc-binding protein	/	**-4.62**	**3.52**
PSPTO_2805	oxidoreductase, FAD-binding	**-3.15**	/	**-3.00**
PSPTO_2861	4Fe-4S binding protein, partial	/	/	**3.22**
PSPTO_2905	vanillate O-demethylase oxidoreductase	**-2.70**	/	**-4.56**
PSPTO_2932	oxidoreductase, aldo/keto reductase family	/	**2.59**	/
PSPTO_2943	vanillin dehydrogenase	**-2.43**	/	**-2.21**
PSPTO_3039	alcohol dehydrogenase	**2.19**	/	/
PSPTO_3080	NADH:flavin oxidoreductase / NADH oxidase family	/	**-2.11**	/
PSPTO_3106	lactoylglutathione lyase	**2.37**	/	/
PSPTO_3184	putative oxidoreductase	**4.03**	/	**2.34**
PSPTO_3262	nitrite reductase	**-1.87**	/	/
PSPTO_3356	isocitrate dehydrogenase	/	/	**-1.80**

PSPTO_3471	putative monovalent cation/H+ antiporter subunit A	**1.85**	**2.07**	/
PSPTO_3497	methylmalonate-semialdehyde dehydrogenase	**-1.82**	/	/
PSPTO_3505	cytochrome b561	**4.88**	/	**3.65**
PSPTO_3601	hypothetical protein PSPTO_3601	**19.53**	/	**11.99**
PSPTO_3860	pyruvate dehydrogenase, E1 component	**2.12**	/	**1.86**
PSPTO_3924	malate dehydrogenase	**2.20**	/	**1.91**
PSPTO_4162	oxidoreductase, molybdopterin-binding protein	**5.60**	/	**4.05**
PSPTO_4170	glycerol-3-phosphate dehydrogenase	**2.72**	/	/
PSPTO_4338	hypothetical protein PSPTO_4338	**-1.83**	/	/
PSPTO_4358	oxidoreductase FAD/FMN-binding protein	/	/	**-2.12**
PSPTO_4367	lipoprotein	**-2.03**	/	**-1.93**
PSPTO_4527	hypothetical protein PSPTO_4527	**-5.58**	**-3.56**	/
PSPTO_4647	cyanide-insensitive terminal oxidase CioB	**4.74**	/	**4.38**
PSPTO_4648	cyanide-insensitive terminal oxidase CioA	**5.68**	/	**4.99**
PSPTO_4652	iron-sulfur cluster-binding protein	**7.52**	/	**9.52**
PSPTO_5072	cytochrome b561	**3.79**	/	**2.33**
PSPTO_5104	hypothetical protein PSPTO_5104	**-2.44**	**-2.00**	/
PSPTO_5600	F0F1 ATP synthase subunit gamma	**1.86**	/	**1.81**
PSPTO_5602	F0F1 ATP synthase subunit delta	**1.86**	/	/
PSPTO_5603	F0F1 ATP synthase subunit B	**1.85**	/	/
PSPTO_5606	F0F1 ATP synthase subunit I	**1.72**	/	**1.81**
PSPTO_5240	CDP-6-deoxy-delta-3,4-glucoseen reductase	**-3.43**	/	**-2.94**
**Carbohydrate transport and metabolism**				
PSPTO_0202	membrane protein	**16.35**	/	**16.05**
PSPTO_0364	sugar ABC transporter periplasmic sugar-binding protein	**-2.30**	/	**-2.61**
PSPTO_0400	chitinase	**5.48**	**1.93**	**2.84**
PSPTO_0563	polyamine ABC transporter periplasmic polyamine-binding protein	**2.94**	/	**2.29**
PSPTO_0889	sugar ABC transporter periplasmic sugar-binding protein	/	/	**-2.09**
PSPTO_0999	major facilitator family transporter	**2.95**	/	/
PSPTO_1047	hypothetical protein PSPTO_1047	**2.68**	/	**2.89**
PSPTO_1049	TRAP dicarboxylate transporter subunit DctM	**3.99**	/	**4.69**
PSPTO_1050	TRAP dicarboxylate transporter, DctQ subunit	**7.54**	/	**8.67**
PSPTO_1051	TRAP dicarboxylate transporter subunit DctP	**8.77**	/	**7.99**
PSPTO_1052	senescence marker protein-30 family protein	**4.08**	/	**3.71**
PSPTO_1236	alginate lyase	**14.18**	/	**6.65**
PSPTO_1287	glyceraldehyde 3-phosphate dehydrogenase, type I	**-2.93**	/	/
PSPTO_1288	6-phosphogluconate dehydratase	**-2.15**	/	/
PSPTO_1289	glucokinase	**-2.09**	/	/
PSPTO_1292	glucose ABC transporter, periplasmic glucose-binding protein	**-2.08**	/	/
PSPTO_1293	glucose ABC transporter permease	**-2.19**	/	/
PSPTO_1300	glucose-6-phosphate 1-dehydrogenase	**-1.93**	/	/
PSPTO_1301	6-phosphogluconolactonase	**-2.25**	/	/
PSPTO_1302	keto-hydroxyglutarate-aldolase/keto-deoxy-phosphogluconate aldolase	**-2.42**	/	/
PSPTO_1345	gluconolactonase	**2.50**	/	**1.81**
PSPTO_1469	major facilitator family transporter	**-2.10**	/	/
PSPTO_1524	glycosyl transferase family protein	**5.05**	**2.15**	**2.34**

PSPTO_1608	hypothetical protein PSPTO_1608	**2.20**	/	/
PSPTO_1612	major facilitator family transporter	**2.91**	/	**2.56**
PSPTO_1707	L-sorbosone dehydrogenase	**5.27**	/	**5.21**
PSPTO_1906	major facilitator family transporter	**-1.84**	/	/
PSPTO_2102	glyceraldehyde 3-phosphate dehydrogenase, type I	/	**1.91**	/
PSPTO_2287	methylisocitrate lyase	**3.26**	/	/
PSPTO_2305	levansucrase	**-1.84**	/	/
PSPTO_2367	ribose ABC transporter periplasmic ribose-binding protein	**-2.12**	/	/
PSPTO_2399	ribose ABC transporter periplasmic ribose-binding protein	**-2.48**	/	**-1.93**
PSPTO_2400	ribose ABC transporter permease	**-2.47**	/	**-2.00**
PSPTO_2467	major facilitator family transporter	/	/	**2.01**
PSPTO_2470	senescence marker protein-30 family protein	/	/	**1.93**
PSPTO_2473	periplasmic substrate-binding protein	/	**-2.57**	/
PSPTO_2493	glycosyl hydrolase family protein	**3.30**	/	**4.57**
PSPTO_2638	L-arabinose ABC transporter periplasmic L-arabinose-binding protein	**-2.28**	/	/
PSPTO_2731	periplasmic sugar-binding domain protein	**5.75**	/	**3.88**
PSPTO_2760	alpha-amylase family protein	**2.99**	/	**3.53**
PSPTO_2761	alpha-amylase family protein	**2.60**	/	**3.59**
PSPTO_2762	1,4-alpha-glucan-branching protein	**2.47**	/	**3.28**
PSPTO_2826	hypothetical protein PSPTO_2826	**3.71**	**-2.73**	**10.14**
PSPTO_3018	hypothetical protein PSPTO_3018	**-2.38**	/	/
PSPTO_3035	phosphoglucomutase, alpha-D-glucose phosphate-specific	**1.89**	/	**1.90**
PSPTO_3122	6-phosphogluconate dehydrogenase	**2.05**	/	**1.90**
PSPTO_3125	glycogen synthase	**7.36**	/	**5.53**
PSPTO_3126	alpha-amylase family protein	**5.38**	/	**4.02**
PSPTO_3127	4-alpha-glucanotransferase	**5.05**	/	**4.94**
PSPTO_3128	glycosyl hydrolase family protein	**5.47**	/	**5.03**
PSPTO_3130	glycogen operon protein GlgX	**6.25**	/	**5.70**
PSPTO_3278	hypothetical protein PSPTO_3278	**-2.77**	/	/
PSPTO_3488	sugar ABC transporter permease	**-2.72**	**-2.20**	/
PSPTO_3490	sugar ABC transporter periplasmic sugar-binding protein	**-4.47**	**-2.24**	**-1.99**
PSPTO_3498	iolB protein	**-2.21**	/	/
PSPTO_3499	iolE protein	**-2.45**	/	/
PSPTO_3500	iolC protein	**-2.23**	/	/
PSPTO_3534	glycosyl hydrolase, family 5 PslG	**20.75**	/	**17.78**
PSPTO_3564	gluconokinase	/	/	**1.85**
PSPTO_3666	polysaccharide deacetylase family protein	/	/	**-2.65**
PSPTO_3687	L-sorbosone dehydrogenase	**11.00**	/	**5.22**
PSPTO_3694	glycosyl transferase family protein	**-1.99**	/	**-1.93**
PSPTO_3737	ABC transporter permease	**2.82**	/	**3.22**
PSPTO_3739	ABC transporter ATP-binding protein	**-8.04**	**-6.29**	/
PSPTO_3740	ABC transporter ATP-binding protein	**-13.85**	**-11.62**	/
PSPTO_3771	major facilitator family transporter	/	/	**1.96**
PSPTO_4203	polysaccharide deacetylase family protein	**-2.83**	/	/
PSPTO_4290	beta-glucosidase	**-2.21**	/	/
PSPTO_4306	dicarboxylic acid transport protein	**-2.58**	/	**-3.06**

PSPTO_4337	pyruvate kinase	**-2.16**	/	**-1.95**
PSPTO_4383	AmpG protein	**-1.86**	/	/
PSPTO_4494	triosephosphate isomerase	/	**-1.84**	/
PSPTO_4522	membrane protein	**2.31**	/	**1.83**
PSPTO_4524	hypothetical protein PSPTO_4524	**-2.00**	/	/
PSPTO_5165	glycogen phosphorylase	**4.57**	/	**4.67**
PSPTO_5340	MFS permease-like protein	**-4.14**	**-1.93**	**-2.15**
PSPTO_5490	aldose 1-epimerase family protein	**-1.83**	/	/
PSPTO_0732	hypothetical protein PSPTO_0732	**4.01**	/	**2.74**
PSPTO_3079	hypothetical protein PSPTO_3079	**4.83**	/	**3.13**
PSPTO_4360	dehydrogenase	**-1.85**	/	/
PSPTO_1729	membrane protein	**2.29**	/	**2.35**
PSPTO_3470	membrane protein	**4.94**	/	**2.96**
PSPTO_4083	membrane protein	**5.54**	/	**4.03**
**Amino acid transport and metabolism**				
PSPTO_0125	argininosuccinate lyase	/	/	**-1.85**
PSPTO_0203	cysteine synthase	**62.33**	/	**52.72**
PSPTO_0272	cysteine desulfurase	**-2.50**	/	**-2.39**
PSPTO_0275	DNA-binding protein	**1.74**	/	/
PSPTO_0280	methionine aminopeptidase	**2.63**	/	**2.36**
PSPTO_0394	renal dipeptidase family protein	**-4.04**	**-5.59**	/
PSPTO_0457	sarcosine oxidase, gamma subunit	**-2.90**	**-3.91**	/
PSPTO_0458	sarcosine oxidase, alpha subunit	**-2.94**	**-4.21**	/
PSPTO_0459	sarcosine oxidase subunit delta	**-3.52**	**-4.21**	/
PSPTO_0460	sarcosine oxidase, beta subunit	**-3.13**	**-3.86**	/
PSPTO_0461	serine hydroxymethyltransferase	**-7.37**	**-13.18**	/
PSPTO_0481	ACT domain-containing protein	**3.16**	/	**2.21**
PSPTO_0489	ABC transporter ATP-binding protein	/	/	**2.15**
PSPTO_0508	hypothetical protein PSPTO_0508	**2.51**	/	**2.54**
PSPTO_0518	tryptophan 2-monooxygenase	**2.41**	/	/
PSPTO_0562	polyamine ABC transporter ATP-binding protein	**3.95**	/	**3.00**
PSPTO_0774	beta-alanine--pyruvate aminotransferase	**6.07**	/	**4.18**
PSPTO_0800	glutamate 5-kinase	**2.47**	/	**2.44**
PSPTO_0873	amidinotransferase family protein	**-4.20**	/	**-2.29**
PSPTO_1134	amino acid ABC transporter substrate-binding protein	/	**2.06**	/
PSPTO_1221	LysE family transporter	**5.32**	/	**2.83**
PSPTO_1255	amino acid ABC transporter substrate-binding protein	**-5.84**	**-3.23**	/
PSPTO_1256	amino acid ABC transporter permease	**-6.22**	**-3.82**	/
PSPTO_1257	amino acid ABC transporter permease	**-5.82**	**-3.41**	/
PSPTO_1258	amino acid ABC transporter ATP-binding protein	**-4.74**	**-2.74**	/
PSPTO_1343	hypothetical protein PSPTO_1343	**4.92**	**2.08**	**2.37**
PSPTO_1528	tetrahydrodipicolinate succinylase	**1.80**	/	/
PSPTO_1585	hypothetical protein PSPTO_1585	**3.94**	/	**3.72**
PSPTO_1600	ABC transporter periplasmic substrate-binding protein	**3.19**	/	**3.09**
PSPTO_1631	hypothetical protein PSPTO_1631	**2.50**	/	**2.34**
PSPTO_1826	arginine/ornithine ABC transporter, periplasmic arginine/ornithine-binding protein	**1.79**	/	/

PSPTO_2045	methylthioribulose-1-phosphate dehydratase	**-1.98**	/	/
PSPTO_2061	transglutaminase-like domain protein	**3.27**	/	**2.46**
PSPTO_2120	peptidase, M20/M25/M40 family	**2.03**	/	/
PSPTO_2261	racemase	**2.77**	/	**2.94**
PSPTO_2451	sarcosine oxidase subunit delta	/	/	**-4.27**
PSPTO_2775	amino acid ABC transporter substrate-binding protein	**-8.26**	**-3.74**	**-2.21**
PSPTO_2776	amino acid ABC transporter permease	**-7.27**	**-3.75**	**-1.94**
PSPTO_2777	amino acid ABC transporter permease	**-5.43**	**-3.33**	/
PSPTO_2781	hypothetical protein PSPTO_2781	**-2.39**	/	/
PSPTO_2902	glutamine amidotransferase	**3.01**	/	/
PSPTO_3059	glycine betaine/L-proline ABC transporter permease	**-2.58**	/	/
PSPTO_3060	glycine betaine/L-proline ABC transporter ATP-binding protein	**-2.69**	**-2.56**	/
PSPTO_3176	thermolysin metallopeptidase	**12.15**	/	**10.39**
PSPTO_3287	D-isomer specific 2-hydroxyacid dehydrogenase family protein	**-2.25**	/	**-1.87**
PSPTO_3647	hypothetical protein PSPTO_3647	**8.46**	/	**6.77**
PSPTO_3717	ABC transporter substrate-binding protein	/	/	**-2.10**
PSPTO_3780	glutathionylspermidine synthase	**2.07**	/	**2.30**
PSPTO_3819	aspartate-semialdehyde dehydrogenase	**3.43**	/	**3.27**
PSPTO_3867	dihydrodipicolinate synthetase family protein	**2.47**	/	**2.16**
PSPTO_3876	aspartyl aminopeptidase	/	/	**-1.83**
PSPTO_3881	polyamine ABC transporter permease	**2.31**	/	**2.32**
PSPTO_3882	polyamine ABC transporter, ATP-binding protein	**2.26**	/	**2.10**
PSPTO_3883	HAD-superfamily hydrolase	/	/	**2.04**
PSPTO_3893	glyoxalase	**1.97**	/	/
PSPTO_4108	leucine ABC transporter subunit substrate-binding protein LivK	**-2.47**	/	/
PSPTO_4110	leucine/isoleucine/valine transporter permease subunit	**-1.90**	/	/
PSPTO_4136	amino acid ABC transporter substrate-binding protein	**-5.84**	**-3.67**	/
PSPTO_4138	histidine/lysine/arginine/ornithine ABC transporter permease HisM	**-3.45**	/	/
PSPTO_4173	amino acid ABC transporter permease	**3.56**	/	**2.94**
PSPTO_4540	proline iminopeptidase	**6.90**	/	**4.68**
PSPTO_4558	dipeptide ABC transporter, periplasmic dipeptide-binding protein	/	**-2.30**	/
PSPTO_4919	high affinity branched-chain amino acid ABC transporter periplasmic amino acid-binding protein	**1.78**	/	/
PSPTO_4924	transglutaminase-like domain protein	**-2.15**	**-2.79**	/
PSPTO_5010	amino acid ABC transporter, periplasmic amino acid-binding protein	**3.67**	/	**3.43**
PSPTO_5016	proline permease	**2.74**	**3.65**	/
PSPTO_5099	histidine ammonia-lyase	**-2.77**	/	/
PSPTO_5270	urocanate hydratase	**-2.57**	/	**-2.62**
PSPTO_5309	glutamine synthetase	**2.07**	/	/
PSPTO_5356	gamma-aminobutyrate permease	**-4.50**	/	**-2.70**
PSPTO_5393	hypothetical protein PSPTO_5393	**2.25**	/	**1.82**
PSPTO_5394	carbon-nitrogen hydrolase family protein	**2.39**	/	/
PSPTO_5499	aspartate ammonia-lyase	**4.70**	**5.55**	/
PSPTO_1729	membrane protein	**2.29**	/	**2.35**
PSPTO_3470	membrane protein	**4.94**	/	**2.96**

PSPTO_4083	membrane protein	**5.54**	/	**4.03**
PSPTO_4549	5-dehydro-4-deoxyglucarate dehydratase	**-2.29**	/	**-1.91**
PSPTO_4538	peptide ABC transporter permease	**1.96**	/	**2.64**
**Nucleotide transport and metabolism**				
PSPTO_0043	cytidine/deoxycytidylate deaminase family protein	**4.79**	/	**4.49**
PSPTO_0073	guanosine-3\',5\'-bis(diphosphate) 3\'-pyrophosphohydrolase	/	/	**-1.75**
PSPTO_0230	adenylate cyclase	**2.18**	/	**2.40**
PSPTO_0456	formyltetrahydrofolate deformylase	**6.44**	/	**6.26**
PSPTO_0549	bis(5\'-nucleosyl)-tetraphosphatase, symmetrical	**1.86**	/	**1.98**
PSPTO_0757	adenosine deaminase	**2.14**	/	/
PSPTO_0772	xanthine/uracil permease family protein	**-2.49**	/	**-2.18**
PSPTO_1130	uracil phosphoribosyltransferase	**1.87**	/	/
PSPTO_1153	NAD(P)H-flavin oxidoreductase	**15.38**	/	**10.77**
PSPTO_1602	cytosine deaminase	**1.84**	/	/
PSPTO_2309	dihydroorotate dehydrogenase	**-1.87**	/	/
PSPTO_2373	inosine/uridine-preferring nucleoside hydrolase	**1.85**	/	/
PSPTO_2474	DNA/RNA non-specific endonuclease	**7.67**	/	**4.39**
PSPTO_2654	4Fe-4S binding protein	**4.26**	/	**7.21**
PSPTO_2670	mutT/nudix family protein	**1.97**	/	/
PSPTO_3668	allantoicase	/	/	**-2.19**
PSPTO_4314	formyltetrahydrofolate deformylase	/	/	**-1.88**
PSPTO_4650	aldehyde oxidase and xanthine dehydrogenase family protein	**5.00**	/	**6.50**
PSPTO_4651	oxidoreductase, molybdopterin-binding subunit	**7.37**	/	**10.43**
PSPTO_4795	AMP nucleosidase	/	/	**-1.76**
PSPTO_4867	phosphoribosylamine--glycine ligase	**1.83**	/	**1.90**
PSPTO_5282	thymidylate synthase	**-2.57**	**-2.41**	/
PSPTO_5493	phosphoribosylaminoimidazole carboxylase ATPase subunit	**-1.86**	/	/
PSPTO_1871	cytosine/purines uracil thiamine allantoin permease	**2.69**	/	**2.23**
**Coenzyme transport and metabolism**				
PSPTO_0495	8-amino-7-oxononanoate synthase	**-1.87**	/	/
PSPTO_0510	coenzyme PQQ synthesis protein D	**4.54**	/	**2.76**
PSPTO_0511	coenzyme PQQ synthesis protein C	**2.44**	/	/
PSPTO_0512	coenzyme PQQ synthesis protein B	**1.89**	/	/
PSPTO_0552	pyridoxal phosphate biosynthetic protein PdxA	**-1.89**	/	/
PSPTO_1128	ferrochelatase	**2.08**	/	/
PSPTO_1181	dihydroneopterin aldolase	**-2.48**	/	**-2.26**
PSPTO_1182	GTP cyclohydrolase I	**-3.42**	/	**-3.76**
PSPTO_1183	dihydromonapterin reductase	**-2.43**	/	**-3.34**
PSPTO_1248	molybdenum cofactor biosynthesis protein D	**1.97**	/	/
PSPTO_1525	ThiF family protein	**2.72**	/	**2.55**
PSPTO_2105	thiamine biosynthesis lipoprotein	**-41.61**	**-3.19**	**-13.04**
PSPTO_2346	4-hydroxyphenylpyruvate dioxygenase	**2.67**	/	**1.95**
PSPTO_2668	bifunctional 3,4-dihydroxy-2-butanone 4-phosphate synthase/GTP cyclohydrolase II-like protein	**3.27**	/	**3.03**
PSPTO_3154	precorrin-4 C(11)-methyltransferase	**-2.04**	/	/
PSPTO_3553	4-hydroxyphenylpyruvate dioxygenase	**-1.88**	/	/

PSPTO_4034	cinA domain protein	**7.79**	/	**4.27**
PSPTO_4116	pyridoxamine 5\'-phosphate oxidase	**2.45**	/	**2.16**
PSPTO_4225	L-aspartate oxidase	**2.08**	/	/
PSPTO_4798	bifunctional hydroxy-methylpyrimidine kinase/ hydroxy-phosphomethylpyrimidine kinase	**-2.05**	**-2.06**	/
PSPTO_4799	thiamine-phosphate pyrophosphorylase	**-2.10**	**-2.01**	/
PSPTO_5052	HemN family oxidoreductase	**-10.85**	**-2.21**	**-4.91**
PSPTO_5086	2-(5\'\'-triphosphoribosyl)-3\'-dephosphocoenzyme-A synthase	**-5.13**	/	/
PSPTO_5475	chorismate-pyruvate lyase	/	/	**-2.05**
PSPTO_5476	4-hydroxybenzoate octaprenyltransferase	/	/	**-1.90**
PSPTO_5240	CDP-6-deoxy-delta-3,4-glucoseen reductase	**-3.43**	/	**-2.94**
PSPTO_1871	cytosine/purines uracil thiamine allantoin permease	**2.69**	/	**2.23**
**Lipid transport and metabolism**				
PSPTO_0200	hypothetical protein PSPTO_0200	**5.06**	/	**5.09**
PSPTO_0500	acyl-CoA dehydrogenase family protein	**2.43**	/	/
PSPTO_0734	MaoC-like domain protein	**29.06**	**1.93**	**15.08**
PSPTO_0742	MaoC-like domain protein	**3.37**	/	**2.93**
PSPTO_0743	3-ketoacyl-(acyl-carrier-protein) reductase	**2.18**	/	**2.42**
PSPTO_0744	acetyl-CoA acetyltransferase	**2.68**	/	**3.24**
PSPTO_0783	3-hydroxyisobutyrate dehydrogenase	**6.48**	/	**4.74**
PSPTO_0997	acyltransferase family protein	**2.44**	/	**1.94**
PSPTO_2335	exopolysaccharide production protein ExoZ	**3.91**	/	**3.56**
PSPTO_2734	acetyltransferase family protein	**1.93**	/	/
PSPTO_2944	p-hydroxycinnamoyl CoA hydratase/lyase	**-3.13**	/	**-3.60**
PSPTO_3163	enoyl-CoA hydratase/isomerase family protein	**4.80**	/	**4.90**
PSPTO_3164	acetyl-CoA acetyltransferase	**4.68**	/	**4.56**
PSPTO_3282	lipoprotein	**-1.92**	/	/
PSPTO_3455	3-oxoacid CoA-transferase, subunit A family	**-1.96**	/	/
PSPTO_3457	short-chain fatty acid transporter	**-2.39**	/	/
PSPTO_3463	CDP-diacylglycerol--serine O-phosphatidyltransferase	/	/	**-2.00**
PSPTO_3503	cardiolipin synthetase 2	/	/	**2.29**
PSPTO_3705	enoly-CoA hydratase/isomerase family protein	**2.53**	/	**2.36**
PSPTO_3706	acyl-CoA dehydrogenase	**2.74**	/	**2.95**
PSPTO_3857	acyl-CoA dehydrogenase	**5.63**	/	**4.27**
PSPTO_4307	3-oxoadipyl-CoA thiolase	**-2.21**	/	**-2.25**
PSPTO_4517	fatty acid desaturase	**2.36**	/	/
PSPTO_4860	acetyl-CoA carboxylase, biotin carboxyl carrier protein	**-1.96**	/	**-2.11**
PSPTO_4861	acetyl-CoA carboxylase, biotin carboxylase	**-2.08**	/	/
PSPTO_4955	bifunctional thiosulfate sulfurtransferase/phosphatidylserine decarboxylase	**-3.07**	/	**-2.25**
PSPTO_5020	acyl-CoA dehydrogenase family protein	**2.15**	/	**2.09**
PSPTO_5081	malonyl CoA-acyl carrier protein transacylase	**-3.62**	/	/
PSPTO_5084	malonate decarboxylase subunit beta	**-4.99**	/	/
PSPTO_5092	acyltransferase family protein	**-2.10**	/	/
PSPTO_5107	3-oxoacyl-(acyl carrier protein) synthase I	**-2.22**	/	/
PSPTO_5108	hypothetical protein PSPTO_5108	**-2.48**	/	/
PSPTO_5109	3-ketoacyl-(acyl-carrier-protein) reductase	**-2.23**	/	/
PSPTO_5110	3-oxoacyl-(acyl carrier protein) synthase II	**-2.40**	/	/

PSPTO_5145	poly(3-hydroxyalkanoate) polymerase	**29.88**	/	**23.07**
PSPTO_5299	hypothetical protein PSPTO_5299	**2.93**	/	**2.29**
PSPTO_5382	ACP phosphodiesterase	**2.10**	/	**2.16**
PSPTO_5489	cytosolic long-chain acyl-CoA thioester hydrolase family protein	**-4.11**	/	**-2.43**
PSPTO_5520	cytosolic long-chain acyl-CoA thioester hydrolase family protein	**-1.83**	/	/
PSPTO_5541	phosphatidate cytidylyltransferase	/	/	**1.93**
PSPTO_0667	short-chain dehydrogenase/reductase family oxidoreductase	**3.54**	/	**2.97**
PSPTO_1056	short chain dehydrogenase/reductase family oxidoreductase	**3.76**	/	**4.38**
PSPTO_1119	short chain dehydrogenase/reductase family oxidoreductase	**3.41**	/	**2.41**
PSPTO_5094	acyl carrier protein	**-3.89**	/	/
PSPTO_5093	acyl carrier protein	**-3.51**	/	**-2.51**
**Inorganic ion transport and metabolism**				
PSPTO_0110	metallo-beta-lactamase superfamily protein	**-2.32**	/	/
PSPTO_0141	lead uptake protein	**-2.00**	/	/
PSPTO_0148	oligopeptide transporter	**2.42**	/	**2.50**
PSPTO_0168	sulfate transporter family protein	**-3.74**	/	**-3.84**
PSPTO_0218	ammonium transporter	**2.92**	/	**2.10**
PSPTO_0314	iron ABC transporter periplasmic iron-binding protein	**3.83**	/	**2.38**
PSPTO_0401	iron-sulfur cluster-binding protein, Rieske family	/	**-2.58**	/
PSPTO_0550	apaG protein	**3.50**	/	**3.83**
PSPTO_0564	polyamine ABC transporter permease	**2.20**	/	**2.10**
PSPTO_0653	bacterioferritin	**1.87**	/	/
PSPTO_0671	TonB-dependent receptor	**3.66**	/	**3.04**
PSPTO_0753	Bcr/CflA family multidrug resistance transporter	/	/	**2.36**
PSPTO_0764	calcium/proton antiporter	/	/	**3.54**
PSPTO_0994	carbonic anhydrase	**-1.81**	/	/
PSPTO_1338	superoxide dismutase, Cu-Zn	**7.65**	/	**8.15**
PSPTO_1340	carbonic anhydrase	**-9.25**	/	**-8.26**
PSPTO_1529	arsC family protein	**1.86**	/	/
PSPTO_1665	hypothetical protein PSPTO_1665	/	/	**2.94**
PSPTO_1687	arsenate reductase	**-2.12**	/	**-1.99**
PSPTO_1901	bacteriophytochrome heme oxygenase BphO	**2.13**	/	/
PSPTO_1930	cyanate lyase	/	/	**-1.87**
PSPTO_2151	TonB-dependent siderophore receptor	**-1.85**	/	**-1.88**
PSPTO_2304	nitrate transporter	**-2.18**	/	/
PSPTO_2398	ribose ABC transporter ATP-binding protein	**-2.34**	/	/
PSPTO_2463	TonB-dependent siderophore receptor	/	/	**-2.70**
PSPTO_2517	sodium/hydrogen exchanger family protein	**2.14**	/	**3.39**
PSPTO_2557	phosphonates ABC transporter permease	/	/	**2.74**
PSPTO_2559	phosphonate metabolism protein PhnG	/	/	**2.60**
PSPTO_2560	PhnH protein	**2.47**	/	**2.66**
PSPTO_2561	phosphonate metabolism protein PhnI	/	/	**2.53**
PSPTO_2562	phosphonate metabolism protein PhnJ	**2.29**	/	**2.82**
PSPTO_2635	cation ABC transporter substrate-binding protein	**-2.46**	/	/
PSPTO_2691	membrane protein, TerC family	**-4.33**	/	**-2.46**

PSPTO_2746	OmpA family protein	**2.10**	/	/
PSPTO_2846	TonB-dependent siderophore receptor	**4.08**	/	**2.69**
PSPTO_2904	vanillate O-demethylase, oxygenase subunit	**-2.51**	**2.07**	**-5.20**
PSPTO_3011	TonB-dependent sideophore receptor	**2.43**	/	/
PSPTO_3242	TonB-dependent receptor	**2.34**	/	/
PSPTO_3472	potassium efflux system protein PhaC	/	**2.54**	/
PSPTO_3474	putative monovalent cation/H+ antiporter subunit E	/	**2.12**	/
PSPTO_3476	potassium efflux system protein PhaG	**1.87**	/	/
PSPTO_3489	sugar ABC transporter ATP-binding protein	**-3.42**	**-2.43**	/
PSPTO_3565	gluconate permease	/	/	**2.00**
PSPTO_3574	TonB-dependent siderophore receptor	/	/	**-2.05**
PSPTO_3597	hypothetical protein PSPTO_3597	**-2.27**	/	/
PSPTO_3598	dyp-type peroxidase family protein	**-2.66**	/	/
PSPTO_3599	hypothetical protein PSPTO_3599	**-2.54**	/	**-2.02**
PSPTO_3704	transporter	/	/	**2.27**
PSPTO_3718	ABC transporter permease	**-1.85**	/	**-1.93**
PSPTO_3719	ABC transporter permease	**-1.91**	/	/
PSPTO_3878	ABC transporter substrate-binding protein	**5.24**	/	**4.95**
PSPTO_3915	copper resistance protein B	**2.20**	/	**1.81**
PSPTO_3983	Dps family protein	/	/	**-2.16**
PSPTO_4128	TonB-dependent siderophore receptor	**1.76**	/	/
PSPTO_4193	iron utilization protein	**3.37**	/	**1.87**
PSPTO_4280	membrane protein	**-7.13**	**-2.52**	**-2.83**
PSPTO_4300	drug resistance transporter, EmrB/QacA family	**3.46**	/	**2.15**
PSPTO_4312	phosphate transporter family protein	**-2.14**	/	**-2.13**
PSPTO_4363	superoxide dismutase, Fe	/	/	**-2.04**
PSPTO_4366	iron-regulated protein A	**-2.04**	/	**-1.97**
PSPTO_4530	catalase/peroxidase HPI	**3.53**	/	**2.62**
PSPTO_4613	C4-dicarboxylate transporter/malic acid transport protein	**2.44**	/	/
PSPTO_4906	bacterioferritin	**4.60**	/	**4.98**
PSPTO_5079	malonate transporter subunit MadM	**-3.54**	/	/
PSPTO_5080	malonate transporter subunit MadL	**-4.11**	/	/
PSPTO_5115	gluconate transporter family protein	**-2.15**	/	/
PSPTO_5174	sodium-proton antiporter NhaA	**2.60**	/	**3.13**
PSPTO_5239	ChaC-related protein	**-1.89**	/	**-2.29**
PSPTO_5263	catalase	**18.24**	/	**20.35**
PSPTO_5301	putrescine ABC transporter permease	/	/	**1.98**
PSPTO_5483	phosphate transport system protein PhoU	**3.17**	/	**1.99**
PSPTO_5484	phosphate transporter ATP-binding protein	**3.67**	/	**2.17**
PSPTO_5519	sodium/hydrogen exchanger family protein	**2.94**	**2.13**	/
PSPTO_5542	glucan biosynthesis protein D	**-2.33**	/	/
PSPTO_5560	TonB-dependent receptor	/	/	**1.97**
PSPTO_5562	iron compound ABC transporter substrate-binding protein	**3.80**	/	**2.36**
PSPTO_4538	peptide ABC transporter permease	**1.96**	/	**2.64**
**Secondary metabolites biosynthesis, transport and catabolism**				
PSPTO_1009	isochorismatase family protein	**4.22**	/	**2.60**
PSPTO_1456	multicopper oxidase	/	**-1.88**	/

PSPTO_1677	dienelactone hydrolase family protein	**3.22**	/	**2.34**
PSPTO_1868	homospermidine synthase	**2.65**	/	**3.85**
PSPTO_2039	isochorismatase family protein	**-1.72**	/	/
PSPTO_2209	dienelactone hydrolase family protein	/	**-2.09**	**3.01**
PSPTO_2216	4-hydroxybenzoyl-CoA thioesterase	**2.34**	/	**2.29**
PSPTO_2614	dioxygenase, TauD/TfdA family	/	/	**-2.63**
PSPTO_2829	non-ribosomal peptide synthetase SyfA	**7.90**	**-7.37**	**58.20**
PSPTO_2830	non-ribosomal peptide synthetase SyfB	**4.56**	**-7.31**	**33.31**
PSPTO_2865	4-carboxymuconolactone decarboxylase	**2.61**	/	/
PSPTO_3193	metalloprotease	**-3.53**	**-1.94**	**-1.82**
PSPTO_3332	alkaline metalloendoprotease	**107.85**	**5.85**	**18.45**
PSPTO_3914	copper resistance protein A	**2.12**	/	**1.87**
PSPTO_4177	2-hydroxychromene-2-carboxylate isomerase	**-1.96**	/	/
PSPTO_4212	methyltransferase	**-1.91**	/	**-2.00**
PSPTO_4286	hypothetical protein PSPTO_4286	**-2.14**	/	/
PSPTO_4518	non-ribosomal peptide synthetase, initiating component	**1.99**	/	/
PSPTO_5083	malonate decarboxylase subunit gamma	**-4.37**	/	/
PSPTO_5087	malonate decarboxylase subunit alpha	**-4.25**	**-2.62**	/
PSPTO_5096	AMP-binding protein	**-3.35**	/	/
PSPTO_5117	N-acyl-D-amino acid deacylase family protein	**-2.36**	/	**-1.98**
PSPTO_5198	dioxygenase, TauD/TfdA family	**2.37**	/	/
PSPTO_5457	ornithine acetyl transferase inhibitor	**2.13**	/	/
PSPTO_0667	short-chain dehydrogenase/reductase family oxidoreductase	**3.54**	/	**2.97**
PSPTO_1056	short chain dehydrogenase/reductase family oxidoreductase	**3.76**	/	**4.38**
PSPTO_1119	short chain dehydrogenase/reductase family oxidoreductase	**3.41**	/	**2.41**
PSPTO_5094	acyl carrier protein	**-3.89**	/	/
PSPTO_5093	acyl carrier protein	**-3.51**	/	**-2.51**
**Function unknown**				
PSPTO_0013	hypothetical protein PSPTO_0013	**1.96**	/	/
PSPTO_0015	hypothetical protein PSPTO_0015	/	**-2.09**	/
PSPTO_0020	hypothetical protein PSPTO_0020	**2.08**	/	**2.76**
PSPTO_0021	hypothetical protein PSPTO_0021	/	/	**2.34**
PSPTO_0024	hypothetical protein PSPTO_0024	**4.46**	/	**2.77**
PSPTO_0025	haloacid dehalogenase-like family hydrolase	**1.84**	/	/
PSPTO_0038	hypothetical protein PSPTO_0038	**2.63**	/	**2.35**
PSPTO_0048	hypothetical protein PSPTO_0048	**2.87**	/	**2.88**
PSPTO_0049	hypothetical protein PSPTO_0049	**3.16**	/	/
PSPTO_0050	hypothetical protein PSPTO_0050	**4.43**	/	**2.74**
PSPTO_0059	hypothetical protein PSPTO_0059	**1.90**	/	/
PSPTO_0099	hypothetical protein PSPTO_0099	/	/	**-1.89**
PSPTO_0117	methyl-accepting chemotaxis protein	/	**-2.13**	/
PSPTO_0120	membrane protein	/	/	**2.02**
PSPTO_0136	alginate regulatory protein AlgR3	**-2.11**	/	**-2.00**
PSPTO_0151	hypothetical protein PSPTO_0151	**3.71**	/	**3.20**
PSPTO_0154	hypothetical protein PSPTO_0154	**3.81**	/	**5.48**
PSPTO_0155	hypothetical protein PSPTO_0155	**3.41**	/	**3.60**
PSPTO_0156	hypothetical protein PSPTO_0156	**5.21**	/	**3.19**

PSPTO_0167	hypothetical protein PSPTO_0167	**-3.18**	/	**-3.50**
PSPTO_0201	hypothetical protein PSPTO_0201	**10.03**	/	**8.77**
PSPTO_0206	hypothetical protein PSPTO_0206	**3.80**	**2.07**	**1.83**
PSPTO_0213	proteic killer protein	**2.02**	/	/
PSPTO_0216	hypothetical protein PSPTO_0216	**4.04**	/	**2.42**
PSPTO_0219	hypothetical protein PSPTO_0219	**2.99**	/	**3.67**
PSPTO_0220	hypothetical protein PSPTO_0220	**2.88**	/	**3.11**
PSPTO_0242	hypothetical protein PSPTO_0242	**2.59**	/	**3.79**
PSPTO_0243	hypothetical protein PSPTO_0243	**6.61**	/	**6.51**
PSPTO_0262	hypothetical protein PSPTO_0262	**10.54**	**2.97**	**3.55**
PSPTO_0263	methyl-accepting chemotaxis protein	/	**-2.26**	**1.92**
PSPTO_0274	hypothetical protein PSPTO_0274	**6.33**	/	**7.15**
PSPTO_0276	haloacid dehalogenase-like family hydrolase	**5.04**	/	**5.18**
PSPTO_0278	hypothetical protein PSPTO_0278	**3.60**	/	**3.46**
PSPTO_0279	hypothetical protein PSPTO_0279	**2.02**	/	**1.86**
PSPTO_0295	hypothetical protein PSPTO_0295	**3.31**	/	**2.48**
PSPTO_0312	hypothetical protein PSPTO_0312	**3.52**	/	**3.42**
PSPTO_0318	hypothetical protein PSPTO_0318	**2.85**	**2.31**	/
PSPTO_0322	adenylate cyclase	/	/	**-1.90**
PSPTO_0330	hypothetical protein PSPTO_0330	**4.09**	/	**5.04**
PSPTO_0332	hypothetical protein PSPTO_0332	**3.28**	/	**3.10**
PSPTO_0342	hypothetical protein PSPTO_0342	**5.57**	/	**5.02**
PSPTO_0363	sorbitol dehydrogenase	**2.18**	/	/
PSPTO_0371	indoleacetate-lysine ligase	**-11.27**	/	**-4.70**
PSPTO_0380	hypothetical protein PSPTO_0380	**2.08**	/	**1.95**
PSPTO_0381	hypothetical protein PSPTO_0381	**2.45**	/	/
PSPTO_0391	hypothetical protein PSPTO_0391	**1.93**	/	/
PSPTO_0395	hypothetical protein PSPTO_0395	**-4.28**	**-7.81**	/
PSPTO_0468	hypothetical protein PSPTO_0468	**2.14**	/	/
PSPTO_0493	competence protein ComF	**2.38**	/	/
PSPTO_0499	hypothetical protein PSPTO_0499	**-1.78**	/	/
PSPTO_0507	hypothetical protein PSPTO_0507	**2.22**	/	/
PSPTO_0509	coenzyme PQQ synthesis protein E	**3.23**	/	**2.64**
PSPTO_0513	coenzyme PQQ synthesis protein A	/	**3.17**	/
PSPTO_0533		**-141.77**	**-118.58**	/
PSPTO_0545	SpoVR like family protein	**3.54**	/	**2.30**
PSPTO_0546	hypothetical protein PSPTO_0546	**4.25**	/	**2.65**
PSPTO_0585	hypothetical protein PSPTO_0585	**3.12**	**2.12**	/
PSPTO_0666	hypothetical protein PSPTO_0666	**2.18**	/	/
PSPTO_0675	arylesterase	**7.58**	/	**10.69**
PSPTO_0684	hypothetical protein PSPTO_0684	**52.24**	**2.17**	**24.07**
PSPTO_0685	hypothetical protein PSPTO_0685	**14.03**	**2.34**	**5.99**
PSPTO_0714	autotransporter	**2.25**	/	**1.96**
PSPTO_0725	GNAT family acetyltransferase	**2.71**	/	**1.78**
PSPTO_0751	hypothetical protein PSPTO_0751	**2.22**	/	**1.77**
PSPTO_0758	2OG-Fe(II) oxygenase family oxidoreductase	**4.85**	/	**2.92**
PSPTO_0759	bmp family protein	**3.05**	/	/
PSPTO_0766	short chain dehydrogenase	**1.77**	/	/

PSPTO_0770	hypothetical protein PSPTO_0770	**-2.52**	/	/
PSPTO_0801	creA protein	**3.00**	/	**3.04**
PSPTO_0803	hypothetical protein PSPTO_0803	**2.56**	/	**2.29**
PSPTO_0804	membrane protein, MviN family	**1.85**	/	**1.88**
PSPTO_0837	hypothetical protein PSPTO_0837	**-2.65**	/	/
PSPTO_0846		**-2.38**	/	**-2.88**
PSPTO_0851	hypothetical protein PSPTO_0851	**-3.69**	/	**-3.05**
PSPTO_0856	hypothetical protein PSPTO_0856	**1.93**	/	**2.30**
PSPTO_0867	hypothetical protein PSPTO_0867	**-2.88**	/	/
PSPTO_0869	hypothetical protein PSPTO_0869	**-2.68**	/	**-2.61**
PSPTO_0871	macrolide efflux protein	**-1.86**	/	/
PSPTO_0874	nikkomycin biosynthesis domain protein	**-2.39**	/	/
PSPTO_0875	hypothetical protein PSPTO_0875	**-2.35**	/	/
PSPTO_0877	type III effector HopQ1-1	**-5.86**	/	**-4.24**
PSPTO_0891	hypothetical protein PSPTO_0891	**2.61**	/	**3.08**
PSPTO_0900	hypothetical protein PSPTO_0900	**2.32**	/	/
PSPTO_0914	STAS domain-containing protein	**146.42**	/	**116.99**
PSPTO_0916	methyl-accepting chemotaxis protein	**77.78**	/	**54.03**
PSPTO_0935	hypothetical protein PSPTO_0935	**3.01**	/	**2.56**
PSPTO_0936	hypothetical protein PSPTO_0936	**2.99**	/	**2.11**
PSPTO_0937	hypothetical protein PSPTO_0937	**2.81**	/	**2.44**
PSPTO_0975	pentapeptide repeat-containing protein	/	**-2.05**	/
PSPTO_0985	hypothetical protein PSPTO_0985	**3.96**	/	**3.18**
PSPTO_0993	hypothetical protein PSPTO_0993	**5.29**	/	**4.87**
PSPTO_0996	HD domain-containing protein	/	/	**-1.99**
PSPTO_1002		**-2.34**	/	**-2.26**
PSPTO_1006	hypothetical protein PSPTO_1006	**8.64**	/	**7.06**
PSPTO_1008	methyl-accepting chemotaxis protein	**16.67**	/	**11.48**
PSPTO_1044	hypothetical protein PSPTO_1044	**2.37**	/	**2.37**
PSPTO_1045	virulence-associated protein	**1.89**	/	/
PSPTO_1046	virulence-associated protein	**2.16**	/	/
PSPTO_1048	membrane protein	**2.22**	/	**2.77**
PSPTO_1061	methyl-accepting chemotaxis protein	/	/	**2.35**
PSPTO_1062	hypothetical protein PSPTO_1062	**50.89**	**2.40**	**21.24**
PSPTO_1068	membrane protein	**2.60**	/	**3.66**
PSPTO_1090	hypothetical protein PSPTO_1090	**18.52**	/	**8.62**
PSPTO_1091	hypothetical protein PSPTO_1091	**16.37**	/	**8.03**
PSPTO_1115	membrane protein	**2.14**	/	/
PSPTO_1117	hypothetical protein PSPTO_1117	**3.46**	/	**2.85**
PSPTO_1118	amine oxidase, flavin-containing protein	**3.81**	/	**2.99**
PSPTO_1127	hypothetical protein PSPTO_1127	**3.24**	/	**2.52**
PSPTO_1142	hypothetical protein PSPTO_1142	**2.33**	/	/
PSPTO_1149	hypothetical protein PSPTO_1149	/	**-2.79**	**3.01**
PSPTO_1150	hypothetical protein PSPTO_1150	/	**-3.17**	**2.74**
PSPTO_1159	bmp family protein	**-2.54**	/	/
PSPTO_1160	ABC transporter ATP-binding protein	**-2.46**	/	/
PSPTO_1176	hypothetical protein PSPTO_1176	**36.04**	**9.75**	**3.69**
PSPTO_1192	hypothetical protein PSPTO_1192	**7.50**	/	**5.83**

PSPTO_1193	hypothetical protein PSPTO_1193	**3.37**	/	**2.59**
PSPTO_1201	lipoprotein	/	**2.11**	**-2.09**
PSPTO_1202	hypothetical protein PSPTO_1202	**7.64**	/	**3.83**
PSPTO_1205	ribosomal subunit interface protein	/	/	**-2.83**
PSPTO_1252	lipoprotein	/	/	**2.26**
PSPTO_1282	hypothetical protein PSPTO_1282	/	/	**-1.81**
PSPTO_1304	hypothetical protein PSPTO_1304	**9.75**	/	**6.70**
PSPTO_1311	hypothetical protein PSPTO_1311	**9.19**	/	**5.70**
PSPTO_1312	fimbral biosynthesis protein	**3.92**	/	**2.96**
PSPTO_1320	hypothetical protein PSPTO_1320	**2.42**	/	**2.90**
PSPTO_1334	methyl-accepting chemotaxis protein	/	**-2.24**	/
PSPTO_1344	hypothetical protein PSPTO_1344	**3.11**	**1.89**	/
PSPTO_1368	lipoprotein	**2.27**	/	/
PSPTO_1371	effector locus protein	**-2.30**	/	**-2.49**
PSPTO_1406	type III effector HopB1	**-81.28**	**-6.58**	**-12.36**
PSPTO_1408	hypothetical protein PSPTO_1408	**-162.90**	**-11.86**	**-13.74**
PSPTO_1409	hypothetical protein PSPTO_1409	**-24.92**	**-3.05**	**-8.18**
PSPTO_1465	transferase, hexapeptide repeat protein	**-1.73**	/	**-1.79**
PSPTO_1466	hypothetical protein PSPTO_1466	**-1.78**	/	**-1.79**
PSPTO_1483	response regulator/EAL domain protein	**7.24**	/	**5.08**
PSPTO_1485	hypothetical protein PSPTO_1485	**2.74**	/	**2.53**
PSPTO_1491	hypothetical protein PSPTO_1491	**3.86**	**2.52**	/
PSPTO_1493	methyl-accepting chemotaxis protein	**3.22**	/	**3.15**
PSPTO_1507	hypothetical protein PSPTO_1507	**6.62**	/	**3.56**
PSPTO_1513	hypothetical protein PSPTO_1513	/	/	**2.21**
PSPTO_1514	hypothetical protein PSPTO_1514	/	**-2.57**	/
PSPTO_1518	hypothetical protein PSPTO_1518	**2.37**	/	**1.90**
PSPTO_1519	hypothetical protein PSPTO_1519	**31.50**	**4.18**	**7.53**
PSPTO_1521	hypothetical protein PSPTO_1521	**1.88**	/	/
PSPTO_1572	hypothetical protein PSPTO_1572	**2.08**	/	**2.11**
PSPTO_1588	hypothetical protein PSPTO_1588	**13.78**	/	**8.86**
PSPTO_1589	lipoprotein	**1.78**	/	/
PSPTO_1590	hypothetical protein PSPTO_1590	**2.03**	/	**1.81**
PSPTO_1594	hypothetical protein PSPTO_1594	**2.08**	/	**2.51**
PSPTO_1595	hypothetical protein PSPTO_1595	/	/	**2.57**
PSPTO_1596	hypothetical protein PSPTO_1596	**6.66**	/	**8.05**
PSPTO_1597	ribonuclease BN	**2.34**	/	**2.42**
PSPTO_1601	hypothetical protein PSPTO_1601	/	/	**1.89**
PSPTO_1603	hypothetical protein PSPTO_1603	**1.98**	/	/
PSPTO_1607	hypothetical protein PSPTO_1607	**1.85**	/	/
PSPTO_1619	hypothetical protein PSPTO_1619	/	/	**2.35**
PSPTO_1623	hypothetical protein PSPTO_1623	**3.86**	/	**4.09**
PSPTO_1624	sodium:solute symporter family protein	**3.08**	/	**3.42**
PSPTO_1625	hypothetical protein PSPTO_1625	**22.20**	/	**15.43**
PSPTO_1626	hypothetical protein PSPTO_1626	**2.92**	/	**3.78**
PSPTO_1628	hypothetical protein PSPTO_1628	**2.65**	/	/
PSPTO_1636	hypothetical protein PSPTO_1636	**3.00**	/	**1.96**
PSPTO_1641	hypothetical protein PSPTO_1641	**7.01**	/	**5.55**

PSPTO_1651	hypothetical protein PSPTO_1651	**2.90**	/	**2.23**
PSPTO_1657	hypothetical protein PSPTO_1657	**14.44**	/	**12.36**
PSPTO_1663	GNAT family acetyltransferase	**9.97**	/	**8.23**
PSPTO_1664	hypothetical protein PSPTO_1664	/	/	**2.34**
PSPTO_1686	trp repressor binding protein	**-1.91**	/	/
PSPTO_1697	hypothetical protein PSPTO_1697	**-2.10**	/	**-3.33**
PSPTO_1726	hypothetical protein PSPTO_1726	**10.48**	/	**5.96**
PSPTO_1730	NAD(P)H dehydrogenase, quinone family	**2.54**	/	**3.00**
PSPTO_1752	hypothetical protein PSPTO_1752	/	/	**-1.97**
PSPTO_1762	hypothetical protein PSPTO_1762	**2.87**	/	**2.13**
PSPTO_1763	hypothetical protein PSPTO_1763	**3.21**	/	**2.04**
PSPTO_1820	hypothetical protein PSPTO_1820	**-1.80**	/	**-2.10**
PSPTO_1824	hypothetical protein PSPTO_1824	/	/	**2.38**
PSPTO_1840	hypothetical protein PSPTO_1840	/	/	**-1.89**
PSPTO_1845	hypothetical protein PSPTO_1845	**18.39**	**2.96**	**6.22**
PSPTO_1848	hypothetical protein PSPTO_1848	**2.72**	/	**2.85**
PSPTO_1849	hypothetical protein PSPTO_1849	**2.44**	/	**2.83**
PSPTO_1860	hypothetical protein PSPTO_1860	**-2.27**	/	**-2.38**
PSPTO_1867		**-2.01**	/	/
PSPTO_1869	hypothetical protein PSPTO_1869	**2.88**	/	**4.17**
PSPTO_1913	hypothetical protein PSPTO_1913	**2.25**	/	/
PSPTO_1914	short chain dehydrogenase/reductase family oxidoreductase	**2.27**	/	**2.43**
PSPTO_1987	chemotaxis protein CheW	/	/	**1.81**
PSPTO_1992	hypothetical protein PSPTO_1992	/	**-2.83**	/
PSPTO_2020	hypothetical protein PSPTO_2020	**2.49**	/	**2.11**
PSPTO_2025	hypothetical protein PSPTO_2025	**2.75**	/	/
PSPTO_2027	hypothetical protein PSPTO_2027	**5.88**	/	**6.48**
PSPTO_2029	oxidoreductase zinc-binding protein	**1.98**	/	**2.85**
PSPTO_2041	hypothetical protein PSPTO_2041	/	**-3.04**	**2.51**
PSPTO_2046	ARD/ARD\' family protein	**-1.87**	/	/
PSPTO_2049	hypothetical protein PSPTO_2049	**5.70**	/	**3.55**
PSPTO_2051	ankyrin domain protein	**4.75**	/	**4.64**
PSPTO_2052	hypothetical protein PSPTO_2052	/	/	**2.05**
PSPTO_2054	hypothetical protein PSPTO_2054	**2.14**	/	/
PSPTO_2057	hypothetical protein PSPTO_2057	**2.50**	**2.07**	/
PSPTO_2058	hypothetical protein PSPTO_2058	**2.19**	**2.08**	/
PSPTO_2059	hypothetical protein PSPTO_2059	**3.22**	/	**2.12**
PSPTO_2060	hypothetical protein PSPTO_2060	**3.39**	/	**2.46**
PSPTO_2062	hypothetical protein PSPTO_2062	**6.74**	/	**5.06**
PSPTO_2069	hypothetical protein PSPTO_2069	**11.13**	/	**9.86**
PSPTO_2074	hypothetical protein PSPTO_2074	**2.44**	/	/
PSPTO_2104	major facilitator family transporter	**-5.60**	**-2.97**	**-1.89**
PSPTO_2114	hypothetical protein PSPTO_2114	**2.15**	/	/
PSPTO_2116	hypothetical protein PSPTO_2116	**-2.11**	/	**-3.51**
PSPTO_2124	hypothetical protein PSPTO_2124	**3.89**	/	**2.88**
PSPTO_2162	hypothetical protein PSPTO_2162	**4.82**	/	**5.51**
PSPTO_2207	lipoprotein	**2.90**	/	**2.02**
PSPTO_2217	hypothetical protein PSPTO_2217	**2.04**	/	**1.89**

PSPTO_2224	hypothetical protein PSPTO_2224	**3.77**	**2.01**	**1.88**
PSPTO_2226	hypothetical protein PSPTO_2226	**2.50**	/	/
PSPTO_2231	YD repeat protein, partial	**2.26**	/	/
PSPTO_2233	hypothetical protein PSPTO_2233	**4.85**	/	**2.71**
PSPTO_2236	hypothetical protein PSPTO_2236	**4.73**	**2.01**	**2.35**
PSPTO_2237	hypothetical protein PSPTO_2237	**1.80**	/	/
PSPTO_2238	lipoprotein	**4.94**	/	**2.77**
PSPTO_2239	YD repeat protein	**2.09**	/	**1.88**
PSPTO_2248	moxR protein	**2.02**	/	/
PSPTO_2253	hypothetical protein PSPTO_2253	**4.67**	/	/
PSPTO_2260	hypothetical protein PSPTO_2260	**2.73**	/	**3.16**
PSPTO_2262	hypothetical protein PSPTO_2262	**3.20**	/	**2.11**
PSPTO_2323	hypothetical protein PSPTO_2323	**2.73**	/	/
PSPTO_2329		**9.88**	**2.19**	**4.51**
PSPTO_2332	hypothetical protein PSPTO_2332	**3.18**	/	/
PSPTO_2333	hypothetical protein PSPTO_2333	**3.48**	/	**2.87**
PSPTO_2334	hypothetical protein PSPTO_2334	**2.57**	/	**1.95**
PSPTO_2356	hypothetical protein PSPTO_2356	**2.57**	/	**2.38**
PSPTO_2361	hypothetical protein PSPTO_2361	/	**2.00**	/
PSPTO_2363	hypothetical protein PSPTO_2363	**2.37**	/	**2.80**
PSPTO_2390	hypothetical protein PSPTO_2390	**6.87**	/	**4.43**
PSPTO_2394	radical SAM domain-containing protein	**2.03**	/	**2.12**
PSPTO_2396	short chain dehydrogenase/reductase family oxidoreductase	**2.06**	/	/
PSPTO_2397	short chain dehydrogenase	**-1.97**	/	/
PSPTO_2422	hypothetical protein PSPTO_2422	**3.32**	/	**3.71**
PSPTO_2441	methyl-accepting chemotaxis protein	/	**-2.26**	**2.12**
PSPTO_2442	chemotaxis protein CheW	/	**-2.53**	**2.09**
PSPTO_2443	hypothetical protein PSPTO_2443	**3.48**	/	**2.37**
PSPTO_2471	membrane protein	**2.56**	/	**3.31**
PSPTO_2472	methyl-accepting chemotaxis protein	/	**-3.18**	/
PSPTO_2476	hypothetical protein PSPTO_2476	**2.00**	/	**2.40**
PSPTO_2492	short-chain dehydrogenase/reductase family oxidoreductase	**2.27**	/	**2.88**
PSPTO_2501	hypothetical protein PSPTO_2501	**-1.86**	/	/
PSPTO_2506	lipoprotein	**3.94**	/	**2.81**
PSPTO_2511	methyl-accepting chemotaxis protein	/	/	**1.72**
PSPTO_2539	secreted protein Hcp	/	/	**-2.44**
PSPTO_2579	carbonic anhydrase-like protein	/	/	**-2.33**
PSPTO_2589	hypothetical protein PSPTO_2589	**111.37**	/	**85.04**
PSPTO_2616	methyl-accepting chemotaxis protein	/	**-2.09**	/
PSPTO_2648	hypothetical protein PSPTO_2648	**1.98**	/	**1.89**
PSPTO_2677	short chain dehydrogenase/reductase family oxidoreductase	**22.08**	/	**31.65**
PSPTO_2679	hypothetical protein PSPTO_2679	**-83.42**	**-6.72**	**-12.42**
PSPTO_2683	hypothetical protein PSPTO_2683	**3.88**	**3.75**	/
PSPTO_2692	hypothetical protein PSPTO_2692	**6.78**	/	**7.60**
PSPTO_2695	hypothetical protein PSPTO_2695	**3.17**	/	**2.61**
PSPTO_2696	mutT/nudix family protein	**5.10**	/	**4.02**
PSPTO_2748	hypothetical protein PSPTO_2748	**3.17**	/	**5.08**

PSPTO_2759	hypothetical protein PSPTO_2759	**3.43**	/	**3.29**
PSPTO_2768	hypothetical protein PSPTO_2768	**61.59**	**2.76**	**22.28**
PSPTO_2769	lipoprotein	**4.39**	/	**4.28**
PSPTO_2773	hypothetical protein PSPTO_2773	/	/	**3.01**
PSPTO_2774	hypothetical protein PSPTO_2774	/	/	**2.28**
PSPTO_2778	amino acid ABC transporter ATP-binding protein	**-4.16**	**-2.62**	/
PSPTO_2779	endoribonuclease L-PSP family protein	**-3.90**	**-2.54**	/
PSPTO_2784	hypothetical protein PSPTO_2784	**1.77**	/	/
PSPTO_2796	hypothetical protein PSPTO_2796	**-10.34**	**-2.85**	**-3.62**
PSPTO_2820	hypothetical protein PSPTO_2820	**-2.91**	/	/
PSPTO_2821	hypothetical protein PSPTO_2821	**-1.95**	/	/
PSPTO_2837		**7.00**	/	**4.59**
PSPTO_2847	ThiJ/PfpI family protein	**4.31**	/	**3.28**
PSPTO_2855	DNA-binding protein	**8.04**	**9.80**	/
PSPTO_2859	hypothetical protein PSPTO_2859	**5.58**	/	**5.70**
PSPTO_2866	cupin family protein	**3.61**	/	**2.79**
PSPTO_2868	hypothetical protein PSPTO_2868	**7.20**	/	**5.65**
PSPTO_2870	hypothetical protein PSPTO_2870	**2.01**	/	**2.62**
PSPTO_2871	hypothetical protein PSPTO_2871	**19.67**	/	**31.37**
PSPTO_2872	HopL1 protein	**11.81**	/	**19.61**
PSPTO_2873	hypothetical protein PSPTO_2873	**13.18**	/	**21.13**
PSPTO_2874	ppkA-related protein	**8.36**	/	**13.63**
PSPTO_2877	hypothetical protein PSPTO_2877	**13.82**	**-1.99**	**27.54**
PSPTO_2878	lipoprotein	**16.11**	/	**25.88**
PSPTO_2879	lipoprotein	**11.88**	/	**23.29**
PSPTO_2894	lectin repeat domain protein	**358.55**	**10.29**	**34.85**
PSPTO_2895	hypothetical protein PSPTO_2895	**6.80**	/	**10.14**
PSPTO_2908	hypothetical protein PSPTO_2908	**8.84**	/	**5.47**
PSPTO_2947	major facilitator family transporter	**-4.32**	/	**-4.68**
PSPTO_2948		**5.62**	/	**4.08**
PSPTO_2952	glycosidase	**2.11**	/	**2.46**
PSPTO_2982	hypothetical protein PSPTO_2982	**13.68**	**2.28**	**5.99**
PSPTO_3038	hypothetical protein PSPTO_3038	**2.98**	/	**2.19**
PSPTO_3042		**4.08**	/	**2.18**
PSPTO_3065	hypothetical protein PSPTO_3065	**2.58**	/	**2.40**
PSPTO_3066	membrane protein	**2.42**	/	**3.20**
PSPTO_3092	hypothetical protein PSPTO_3092	**8.23**	/	**5.25**
PSPTO_3123	hypothetical protein PSPTO_3123	**-3.60**	/	**-3.45**
PSPTO_3129	hypothetical protein PSPTO_3129	**4.72**	/	**3.25**
PSPTO_3131	hypothetical protein PSPTO_3131	**5.67**	/	**5.42**
PSPTO_3132	hypothetical protein PSPTO_3132	**4.62**	/	**5.32**
PSPTO_3133	methyltransferase	**4.32**	/	**5.50**
PSPTO_3135	hypothetical protein PSPTO_3135	**2.60**	/	/
PSPTO_3157	hypothetical protein PSPTO_3157	**2.78**	/	/
PSPTO_3165	lipoprotein	**2.99**	/	**2.77**
PSPTO_3166	hypothetical protein PSPTO_3166	**3.17**	/	**2.51**
PSPTO_3174	hypothetical protein PSPTO_3174	**5.00**	/	**2.44**
PSPTO_3177	hypothetical protein PSPTO_3177	**5.95**	/	**5.19**

PSPTO_3189	hypothetical protein PSPTO_3189	**-2.51**	/	/
PSPTO_3192	amidase family protein	**-2.60**	/	**-2.01**
PSPTO_3199	luciferase family protein	**2.92**	/	/
PSPTO_3200	hypothetical protein PSPTO_3200	**3.08**	/	**3.06**
PSPTO_3201	hypothetical protein PSPTO_3201	**4.33**	/	**3.37**
PSPTO_3202	oxidoreductase, 2-nitropropane dioxygenase family	**2.14**	/	**2.50**
PSPTO_3203	hypothetical protein PSPTO_3203	**2.84**	/	**2.74**
PSPTO_3264	alpha/beta fold family hydrolase	**2.44**	/	/
PSPTO_3270	hypothetical protein PSPTO_3270	**5.03**	/	**4.32**
PSPTO_3271	hypothetical protein PSPTO_3271	**3.97**	/	**4.48**
PSPTO_3286	hypothetical protein PSPTO_3286	**-2.26**	/	**-1.89**
PSPTO_3288	hypothetical protein PSPTO_3288	/	/	**-2.33**
PSPTO_3289	hypothetical protein PSPTO_3289	**-3.59**	/	**-6.02**
PSPTO_3299	3-hydroxyacyl-CoA-acyl carrier protein transferase	**-2.08**	/	/
PSPTO_3305	hypothetical protein PSPTO_3305	**2.54**	/	**2.52**
PSPTO_3333	membrane protein	**2.01**	**2.46**	/
PSPTO_3337	hypothetical protein PSPTO_3337	/	/	**-2.09**
PSPTO_3362	GNAT family acetyltransferase	**3.36**	/	**2.55**
PSPTO_3378	membrane protein	/	/	**2.41**
PSPTO_3386	hypothetical protein PSPTO_3386	**2.17**	/	/
PSPTO_3387	hypothetical protein PSPTO_3387	**2.30**	**2.45**	/
PSPTO_3407	hypothetical protein PSPTO_3407	**-2.48**	/	/
PSPTO_3410	portal protein	**-2.23**	/	/
PSPTO_3412	terminase large subunit	**-1.86**	/	/
PSPTO_3422	hypothetical protein PSPTO_3422	/	**2.13**	**-2.51**
PSPTO_3424	hypothetical protein PSPTO_3424	/	/	**-2.53**
PSPTO_3453	lipoprotein	**2.41**	**2.43**	/
PSPTO_3477	hypothetical protein PSPTO_3477	**-2.13**	/	/
PSPTO_3478	hypothetical protein PSPTO_3478	**7.11**	/	**7.41**
PSPTO_3481	hypothetical protein PSPTO_3481	/	/	**2.65**
PSPTO_3485	lipase family protein	**1.94**	/	/
PSPTO_3494	myo-inositol 2-dehydrogenase	**-1.76**	/	/
PSPTO_3504	endonuclease/exonuclease/phosphatase family protein	**2.07**	/	**2.97**
PSPTO_3538	transferase, hexapeptide repeat protein	**20.12**	/	**20.42**
PSPTO_3539	membrane protein PslK	**16.10**	/	**18.14**
PSPTO_3542	hypothetical protein PSPTO_3542	**5.54**	/	**8.34**
PSPTO_3543	hypothetical protein PSPTO_3543	**2.55**	/	**2.77**
PSPTO_3544	hypothetical protein PSPTO_3544	**-2.53**	/	**-1.84**
PSPTO_3546	hypothetical protein PSPTO_3546	**4.75**	/	**5.02**
PSPTO_3558	glcG protein	**2.05**	/	**1.97**
PSPTO_3567	hypothetical protein PSPTO_3567	/	/	**2.05**
PSPTO_3568	hypothetical protein PSPTO_3568	**7.43**	/	**17.10**
PSPTO_3569	hypothetical protein PSPTO_3569	**20.61**	/	**35.85**
PSPTO_3575	hypothetical protein PSPTO_3575	**2.50**	/	/
PSPTO_3580	methyl-accepting chemotaxis protein	/	**-2.54**	**3.26**
PSPTO_3583	ankyrin domain protein	**-2.91**	/	/
PSPTO_3585	hypothetical protein PSPTO_3585	**1.95**	/	**1.99**

PSPTO_3590	HAD-superfamily hydrolase	**8.17**	/	**8.99**
PSPTO_3591	rarD protein	**1.87**	/	**2.64**
PSPTO_3600	oxidoreductase, molybdopterin-binding protein	**16.02**	/	**11.57**
PSPTO_3602	hypothetical protein PSPTO_3602	**24.51**	/	**14.53**
PSPTO_3605	lyase	**-2.40**	/	/
PSPTO_3616	hypothetical protein PSPTO_3616	**2.56**	/	**4.14**
PSPTO_3650	hypothetical protein PSPTO_3650	**3.52**	/	**2.94**
PSPTO_3655	hypothetical protein PSPTO_3655	**2.94**	/	**5.62**
PSPTO_3677	hypothetical protein PSPTO_3677	/	**1.99**	/
PSPTO_3681	hypothetical protein PSPTO_3681	/	/	**2.36**
PSPTO_3682	hypothetical protein PSPTO_3682	/	/	**1.98**
PSPTO_3688	membrane protein	**14.88**	**2.37**	**6.28**
PSPTO_3689	hypothetical protein PSPTO_3689	**3.79**	/	**3.44**
PSPTO_3690	hypothetical protein PSPTO_3690	**11.91**	/	**9.51**
PSPTO_3691	ea59 protein	**13.44**	/	**10.49**
PSPTO_3693	hypothetical protein PSPTO_3693	**-2.33**	/	**-3.23**
PSPTO_3729	lipoprotein	**-2.10**	/	/
PSPTO_3730	membrane protein	**5.90**	/	**4.64**
PSPTO_3736	hypothetical protein PSPTO_3736	**2.29**	/	**2.66**
PSPTO_3738	ABC transporter permease	**-4.47**	**-3.31**	/
PSPTO_3745	UDP-2,3-diacylglucosamine hydrolase	**-2.05**	/	/
PSPTO_3754	hypothetical protein PSPTO_3754	**3.41**	/	**2.52**
PSPTO_3756	hypothetical protein PSPTO_3756	**2.33**	/	**2.38**
PSPTO_3760	hypothetical protein PSPTO_3760	**-2.08**	/	/
PSPTO_3761	hypothetical protein PSPTO_3761	**-2.39**	/	/
PSPTO_3762	von Willebrand factor type A domain-containing protein	**-2.34**	/	/
PSPTO_3776	hypothetical protein PSPTO_3776	**-2.29**	/	/
PSPTO_3777	membrane protein	**-1.86**	/	/
PSPTO_3779	hypothetical protein PSPTO_3779	/	**-2.04**	/
PSPTO_3781	hypothetical protein PSPTO_3781	**2.04**	/	**2.47**
PSPTO_3782	hypothetical protein PSPTO_3782	**1.94**	/	**1.98**
PSPTO_3783	hypothetical protein PSPTO_3783	**1.93**	/	**2.14**
PSPTO_3865	hypothetical protein PSPTO_3865	**2.67**	/	**2.18**
PSPTO_3879	hypothetical protein PSPTO_3879	**2.67**	/	**2.50**
PSPTO_3880	polyamine ABC transporter permease	**2.38**	/	**2.49**
PSPTO_3889	hypothetical protein PSPTO_3889	**2.34**	/	**2.53**
PSPTO_3903	hypothetical protein PSPTO_3903	**5.14**	/	**4.59**
PSPTO_3905	AAA ATPase	**1.81**	/	/
PSPTO_3907	hypothetical protein PSPTO_3907	**17.52**	/	**11.06**
PSPTO_3917	hypothetical protein PSPTO_3917	**4.26**	/	**6.72**
PSPTO_3923	membrane protein	**2.50**	/	**2.44**
PSPTO_3925	gas vesicle protein	**9.70**	/	**5.52**
PSPTO_3926		**3.64**	/	/
PSPTO_3927	hypothetical protein PSPTO_3927	**2.17**	/	/
PSPTO_3929	cold shock domain family protein	**6.04**	/	**9.02**
PSPTO_3973	tolA protein	**1.85**	/	/
PSPTO_3994	hypothetical protein PSPTO_3994	**3.21**	/	**5.86**
PSPTO_4016	hypothetical protein PSPTO_4016	**3.08**	/	**3.18**

PSPTO_4031	decarboxylase family protein	/	/	**-1.86**
PSPTO_4035	hypothetical protein PSPTO_4035	**2.56**	/	/
PSPTO_4047	hypothetical protein PSPTO_4047	**3.14**	/	**2.22**
PSPTO_4070	hypothetical protein PSPTO_4070	**5.12**	/	**3.64**
PSPTO_4087	hypothetical protein PSPTO_4087	**2.52**	/	**2.58**
PSPTO_4089	alpha/beta fold family hydrolase	**4.51**	/	**4.88**
PSPTO_4102	hypothetical protein PSPTO_4102	**-21.98**	/	**-5.49**
PSPTO_4126	hypothetical protein PSPTO_4126	**3.24**	/	**2.20**
PSPTO_4127	hypothetical protein PSPTO_4127	**2.56**	/	**1.80**
PSPTO_4150	hypothetical protein PSPTO_4150	**3.56**	/	**3.20**
PSPTO_4163	hypothetical protein PSPTO_4163	**2.75**	/	/
PSPTO_4185	membrane protein	**4.94**	/	**5.55**
PSPTO_4186	hypothetical protein PSPTO_4186	**3.20**	/	**3.04**
PSPTO_4207	hypothetical protein PSPTO_4207	/	/	**1.95**
PSPTO_4209	hypothetical protein PSPTO_4209	**2.35**	/	**2.24**
PSPTO_4218	hypothetical protein PSPTO_4218	**2.19**	/	/
PSPTO_4248	3-hydroxyacyl-CoA-acyl carrier protein transferase	**46.43**	**3.28**	**14.17**
PSPTO_4258	NAD(P)H dehydrogenase, quinone family	**-2.90**	/	/
PSPTO_4268		**3.44**	/	**2.19**
PSPTO_4271		**3.18**	/	**2.20**
PSPTO_4272	hypothetical protein PSPTO_4272	**-2.57**	**-2.82**	/
PSPTO_4275	short-chain dehydrogenase/reductase family oxidoreductase	/	/	**-2.03**
PSPTO_4279	hypothetical protein PSPTO_4279	**-4.82**	/	**-2.69**
PSPTO_4281	hypothetical protein PSPTO_4281	**-6.78**	**-2.35**	**-2.89**
PSPTO_4282	hypothetical protein PSPTO_4282	**-6.94**	/	**-3.36**
PSPTO_4297	hypothetical protein PSPTO_4297	**2.83**	**3.70**	/
PSPTO_4298	hypothetical protein PSPTO_4298	**-2.09**	/	**-3.23**
PSPTO_4299	hypothetical protein PSPTO_4299	**-2.21**	/	**-2.13**
PSPTO_4313	hypothetical protein PSPTO_4313	**1.80**	/	**1.75**
PSPTO_4315	transcriptional regulator	/	/	**-2.15**
PSPTO_4321	hypothetical protein PSPTO_4321	/	/	**-2.09**
PSPTO_4322	hypothetical protein PSPTO_4322	**-2.15**	/	**-2.17**
PSPTO_4323	hypothetical protein PSPTO_4323	**-1.81**	/	**-1.98**
PSPTO_4325	hypothetical protein PSPTO_4325	/	/	**1.85**
PSPTO_4332	hypothetical protein PSPTO_4332	**-18.11**	/	**-7.22**
PSPTO_4333	moxR protein	**-1.98**	/	**-1.94**
PSPTO_4334	hypothetical protein PSPTO_4334	**-2.83**	/	**-2.24**
PSPTO_4335	hypothetical protein PSPTO_4335	**10.40**	/	**6.12**
PSPTO_4346	hypothetical protein PSPTO_4346	**-3.49**	/	**-3.54**
PSPTO_4351	hypothetical protein PSPTO_4351	**-2.44**	/	**-2.53**
PSPTO_4355	hypothetical protein PSPTO_4355	**-2.89**	/	**-2.49**
PSPTO_4357	major facilitator family transporter	/	**2.02**	/
PSPTO_4359	hypothetical protein PSPTO_4359	**-2.18**	/	**-2.29**
PSPTO_4368	lipoprotein	**-2.34**	/	**-2.41**
PSPTO_4369	lipoprotein	**-2.50**	/	**-2.23**
PSPTO_4370	hypothetical protein PSPTO_4370	**3.41**	/	**1.93**
PSPTO_4372	hypothetical protein PSPTO_4372	**-2.37**	/	**-3.47**

PSPTO_4375	PAP2 superfamily protein	**1.98**	/	/
PSPTO_4379	hypothetical protein PSPTO_4379	/	/	**-2.14**
PSPTO_4381	hypothetical protein PSPTO_4381	**-2.49**	/	/
PSPTO_4386	hypothetical protein PSPTO_4386	**3.36**	/	**2.58**
PSPTO_4431	hypothetical protein PSPTO_4431	/	/	**-1.84**
PSPTO_4464	hypothetical protein PSPTO_4464	/	/	**-1.95**
PSPTO_4472	rod shape-determining protein MreB	**-1.75**	/	/
PSPTO_4484	hypothetical protein PSPTO_4484	**3.97**	/	/
PSPTO_4516	hypothetical protein PSPTO_4516	**6.66**	/	**6.88**
PSPTO_4525	hypothetical protein PSPTO_4525	**4.52**	/	/
PSPTO_4529	hypothetical protein PSPTO_4529	**1.94**	/	/
PSPTO_4539	LuxR family transcriptional regulator, autoinducer-regulated	**6.62**	**1.93**	**3.43**
PSPTO_4541	methyl-accepting chemotaxis protein	**2.31**	/	**2.62**
PSPTO_4544	membrane protein	/	**-4.70**	**3.27**
PSPTO_4546	hypothetical protein PSPTO_4546	**5.22**	/	**2.30**
PSPTO_4555	hypothetical protein PSPTO_4555	**2.57**	/	**2.59**
PSPTO_4580	hypothetical protein PSPTO_4580	**5.11**	/	**2.62**
PSPTO_4583	hypothetical protein PSPTO_4583	**2.96**	/	**2.01**
PSPTO_4584	hypothetical protein PSPTO_4584	**9.60**	/	**5.91**
PSPTO_4586	hypothetical protein PSPTO_4586	**3.87**	/	**2.24**
PSPTO_4587	hypothetical protein PSPTO_4587	**4.45**	/	**2.97**
PSPTO_4600	lipoprotein	**16.32**	/	**9.07**
PSPTO_4603	site-specific recombinase, phage integrase family	**3.74**	**2.31**	/
PSPTO_4605	hypothetical protein PSPTO_4605	**3.17**	**2.02**	/
PSPTO_4606	hypothetical protein PSPTO_4606	**2.15**	/	/
PSPTO_4607	hypothetical protein PSPTO_4607	**1.91**	/	/
PSPTO_4623	hypothetical protein PSPTO_4623	**3.05**	/	/
PSPTO_4624	methyl-accepting chemotaxis protein	/	/	**2.58**
PSPTO_4639	hypothetical protein PSPTO_4639	**3.01**	/	**2.34**
PSPTO_4646	hypothetical protein PSPTO_4646	**5.29**	/	**4.01**
PSPTO_4649	hypothetical protein PSPTO_4649	**3.78**	/	/
PSPTO_4653	xanthine/uracil permease family protein	**3.59**	/	**3.72**
PSPTO_4657	zinc metallopeptidase	**3.56**	/	**3.21**
PSPTO_4673	hypothetical protein PSPTO_4673	/	/	**2.32**
PSPTO_4684	coronafacic acid synthetase component	**2.66**	/	**2.79**
PSPTO_4706	response regulator CorP	**2.65**	/	/
PSPTO_4719	hypothetical protein PSPTO_4719	**8.27**	/	**5.68**
PSPTO_4733	hypothetical protein PSPTO_4733	**-8.97**	/	**-7.29**
PSPTO_4741	hypothetical protein PSPTO_4741	/	/	**-3.22**
PSPTO_4742	site-specific recombinase, phage integrase family	**-2.48**	/	**-2.45**
PSPTO_4743	hypothetical protein PSPTO_4743	**-3.14**	/	**-2.82**
PSPTO_4746	site-specific recombinase, phage integrase family domain protein	/	/	**-2.33**
PSPTO_4747	hypothetical protein PSPTO_4747	/	/	**-2.14**
PSPTO_4754	hypothetical protein PSPTO_4754	**1.96**	/	/
PSPTO_4770	hypothetical protein PSPTO_4770	**2.30**	/	**1.96**
PSPTO_4771	hypothetical protein PSPTO_4771	**2.91**	/	**2.26**
PSPTO_4772	hypothetical protein PSPTO_4772	**3.23**	/	**3.06**

PSPTO_4786	methyl-accepting chemotaxis protein	**2.54**	/	**2.12**
PSPTO_4791	hypothetical protein PSPTO_4791	**2.18**	/	/
PSPTO_4797	hypothetical protein PSPTO_4797	**2.17**	/	/
PSPTO_4801	hypothetical protein PSPTO_4801	**2.81**	/	/
PSPTO_4802	hypothetical protein PSPTO_4802	**2.03**	/	/
PSPTO_4809	hypothetical protein PSPTO_4809	**4.20**	/	**3.12**
PSPTO_4810	hypothetical protein PSPTO_4810	**17.96**	/	**13.81**
PSPTO_4832	hypothetical protein PSPTO_4832	**9.63**	/	**7.10**
PSPTO_4845	lipoprotein	**2.72**	/	**3.31**
PSPTO_4848	response regulator	**48.46**	**1.91**	**25.36**
PSPTO_4849	hypothetical protein PSPTO_4849	**141.69**	**2.13**	**66.62**
PSPTO_4857	hypothetical protein PSPTO_4857	**57.18**	/	**28.71**
PSPTO_4859	3-dehydroquinate dehydratase	**-1.86**	/	/
PSPTO_4870	hypothetical protein PSPTO_4870	**3.57**	/	**2.36**
PSPTO_4871	hypothetical protein PSPTO_4871	**3.37**	/	**2.56**
PSPTO_4873	hypothetical protein PSPTO_4873	**6.86**	**4.91**	/
PSPTO_4883	membrane protein	**4.59**	/	**7.43**
PSPTO_4899	membrane protein	**5.38**	/	**3.77**
PSPTO_4900	phosphate starvation-inducible protein PsiF	**9.91**	/	**6.82**
PSPTO_4902	hypothetical protein PSPTO_4902	**2.51**	/	**2.87**
PSPTO_4925	hypothetical protein PSPTO_4925	/	**-1.94**	/
PSPTO_4927	hypothetical protein PSPTO_4927	**2.19**	/	**1.96**
PSPTO_4936	methyl-accepting chemotaxis protein	**-2.15**	**-2.08**	/
PSPTO_4942	hfq protein	**2.33**	/	/
PSPTO_4956	hypothetical protein PSPTO_4956	**-1.98**	/	/
PSPTO_4966	hypothetical protein PSPTO_4966	**20.95**	**2.93**	**7.15**
PSPTO_4967	hypothetical protein PSPTO_4967	**175.63**	**2.71**	**64.81**
PSPTO_4970	YD repeat protein	**1.81**	/	/
PSPTO_4986	membrane protein	**-1.94**	/	/
PSPTO_4993	hypothetical protein PSPTO_4993, partial	**2.16**	/	/
PSPTO_5008	hypothetical protein PSPTO_5008	**-1.93**	/	/
PSPTO_5013	hypothetical protein PSPTO_5013	**2.92**	/	**2.18**
PSPTO_5022	hypothetical protein PSPTO_5022	**4.03**	/	**2.28**
PSPTO_5025	hypothetical protein PSPTO_5025	**3.40**	/	/
PSPTO_5048	hypothetical protein PSPTO_5048	**-2.12**	/	/
PSPTO_5053	hypothetical protein PSPTO_5053	**-7.25**	/	**-3.92**
PSPTO_5055	hypothetical protein PSPTO_5055	**2.46**	/	**2.69**
PSPTO_5071	hypothetical protein PSPTO_5071	**4.20**	/	**2.34**
PSPTO_5073	hypothetical protein PSPTO_5073	/	/	**-2.71**
PSPTO_5089	hypothetical protein PSPTO_5089	**5.03**	/	**4.55**
PSPTO_5095	membrane protein	**-3.81**	/	**-2.04**
PSPTO_5100	4-hydroxybenzoyl-CoA thioesterase	**-2.38**	/	**-2.12**
PSPTO_5102	membrane protein	**-2.16**	/	/
PSPTO_5119	hypothetical protein PSPTO_5119	**2.46**	/	/
PSPTO_5142	hypothetical protein PSPTO_5142	**3.48**	/	**2.64**
PSPTO_5143		**2.84**	/	/
PSPTO_5144	poly(3-hydroxyalkanoate) depolymerase	**8.23**	/	**6.71**
PSPTO_5147	polyhydroxyalkanoate granule-associated protein PhaF	**4.09**	/	**3.87**

PSPTO_5159	methyl-accepting chemotaxis protein	**3.27**	/	**2.92**
PSPTO_5166	membrane protein	**2.45**	/	**2.70**
PSPTO_5169	lipoprotein	**2.24**	/	**2.59**
PSPTO_5175	hypothetical protein PSPTO_5175	**49.52**	**1.94**	**25.50**
PSPTO_5201	hypothetical protein PSPTO_5201	**3.12**	/	**2.55**
PSPTO_5202	hypothetical protein PSPTO_5202	**1.95**	/	**1.87**
PSPTO_5203	hypothetical protein PSPTO_5203	**2.85**	/	**2.67**
PSPTO_5218	hypothetical protein PSPTO_5218	**5.51**	/	**3.21**
PSPTO_5228	hypothetical protein PSPTO_5228	**2.09**	/	**2.87**
PSPTO_5233	colicin/pyocin immunity family protein	**2.59**	/	/
PSPTO_5234	hypothetical protein PSPTO_5234	**3.66**	/	**2.78**
PSPTO_5237	tonB domain protein, partial	**2.78**	/	**2.93**
PSPTO_5257	hypothetical protein PSPTO_5257	**2.03**	/	**3.20**
PSPTO_5259	Sco1/SenC family protein	**3.60**	/	**3.17**
PSPTO_5264	lipoprotein	**2.61**	/	**1.85**
PSPTO_5281	hypothetical protein PSPTO_5281	**5.28**	/	**4.43**
PSPTO_5287	hypothetical protein PSPTO_5287	**2.70**	/	/
PSPTO_5290	hypothetical protein PSPTO_5290	**1.96**	/	/
PSPTO_5291	hypothetical protein PSPTO_5291	**5.13**	/	**2.93**
PSPTO_5318	outer membrane porin, OprD family	**2.45**	/	**2.94**
PSPTO_5332	trypsin domain-containing protein	**5.64**	**2.56**	**2.21**
PSPTO_5345	hypothetical protein PSPTO_5345	/	/	**-1.85**
PSPTO_5351	lipoprotein	**3.36**	/	**3.40**
PSPTO_5352	methyl-accepting chemotaxis protein	/	**-2.07**	**2.11**
PSPTO_5355	hypothetical protein PSPTO_5355	**-21.67**	**-2.67**	**-8.13**
PSPTO_5362	hypothetical protein PSPTO_5362	**2.82**	/	**3.47**
PSPTO_5365	hypothetical protein PSPTO_5365	**2.52**	/	**2.20**
PSPTO_5373	hypothetical protein PSPTO_5373	**-2.26**	/	/
PSPTO_5385	hypothetical protein PSPTO_5385	**14.31**	/	**11.97**
PSPTO_5386	hypothetical protein PSPTO_5386	**2.73**	/	/
PSPTO_5389	GNAT family acetyltransferase	**8.83**	/	**5.69**
PSPTO_5390	hypothetical protein PSPTO_5390	**3.58**	/	**4.17**
PSPTO_5392	hypothetical protein PSPTO_5392	**2.77**	/	**2.27**
PSPTO_5404	LysM domain/BON superfamily protein	**14.18**	/	**8.59**
PSPTO_5410	hypothetical protein PSPTO_5410	**1.96**	/	/
PSPTO_5419	hypothetical protein PSPTO_5419	/	/	**1.87**
PSPTO_5420	hypothetical protein PSPTO_5420	/	/	**1.83**
PSPTO_5423	hypothetical protein PSPTO_5423	**2.05**	/	**2.25**
PSPTO_5426	hypothetical protein PSPTO_5426	**2.12**	/	**2.24**
PSPTO_5427	hypothetical protein PSPTO_5427	/	/	**1.90**
PSPTO_5430	hypothetical protein PSPTO_5430	**3.09**	/	**3.56**
PSPTO_5431	hypothetical protein PSPTO_5431	**3.68**	/	**3.60**
PSPTO_5432	hypothetical protein PSPTO_5432	**3.84**	/	**3.33**
PSPTO_5433	hypothetical protein PSPTO_5433	**3.36**	/	**3.06**
PSPTO_5435	secreted protein Hcp	**13.72**	/	**11.47**
PSPTO_5437	hypothetical protein PSPTO_5437	**7.22**	/	**7.56**
PSPTO_5447	hypothetical protein PSPTO_5447	**6.35**	/	**8.26**
PSPTO_5452	HAD-superfamily hydrolase	/	/	**-1.71**

PSPTO_5455	hypothetical protein PSPTO_5455	**2.72**	**/**	**/**
PSPTO_5456	hypothetical protein PSPTO_5456	**2.70**	**/**	**/**
PSPTO_5458	hypothetical protein PSPTO_5458	**2.25**	**/**	**/**
PSPTO_5470	hypothetical protein PSPTO_5470	**4.89**	**/**	**3.01**
PSPTO_5491	hypothetical protein PSPTO_5491	**2.64**	**/**	**3.15**
PSPTO_5492	hypothetical protein PSPTO_5492	**4.23**	**/**	**4.62**
PSPTO_5495	hypothetical protein PSPTO_5495	**48.76**	**15.74**	**3.10**
PSPTO_5496	hypothetical protein PSPTO_5496	**4.16**	**/**	**4.14**
PSPTO_5497	hypothetical protein PSPTO_5497	**12.03**	**5.16**	**/**
PSPTO_5513	hypothetical protein PSPTO_5513	**5.28**	**/**	**3.34**
PSPTO_5514	hypothetical protein PSPTO_5514	**16.00**	**3.43**	**4.67**
PSPTO_5517	hypothetical protein PSPTO_5517	**2.40**	**1.94**	**/**
PSPTO_5526	cobalamin synthesis protein/P47K family protein	**-1.89**	**/**	**-2.25**
PSPTO_5554	methyl-accepting chemotaxis protein	**/**	**-2.79**	**2.24**
PSPTO_5558	hypothetical protein PSPTO_5558	**19.95**	**/**	**28.62**
PSPTO_5565	hypothetical protein PSPTO_5565	**2.64**	**/**	**/**
PSPTO_5572		**2.28**	**/**	**1.90**
PSPTO_5574		**/**	**2.30**	**/**
PSPTO_5577		**/**	**/**	**2.62**
PSPTO_5592	hypothetical protein PSPTO_5592	**2.39**	**/**	**2.32**
PSPTO_5604	F0F1 ATP synthase subunit C	**2.02**	**/**	**1.89**
PSPTO_5616	hypothetical protein PSPTO_5616	**-176.49**	**-7.99**	**-22.08**
PSPTO_5619	hypothetical protein PSPTO_5619	**-10.05**	**/**	**-14.12**
PSPTO_5621	hypothetical protein PSPTO_5621	**2.28**	**/**	**/**
PSPTO_5622	hypothetical protein PSPTO_5622	**-1390.71**	**-81.07**	**-17.15**
PSPTO_5625	binary cytotoxin component	**8.38**	**2.77**	**3.02**
PSPTO_5630	hypothetical protein PSPTO_5630	**5.54**	**/**	**4.39**
PSPTO_5631	hypothetical protein PSPTO_5631	**7.20**	**2.55**	**2.82**
PSPTO_5632	hypothetical protein PSPTO_5632	**/**	**2.09**	**-1.94**
PSPTO_5636	hypothetical protein PSPTO_5636	**2.09**	**/**	**2.30**
PSPTO_5642	hypothetical protein PSPTO_5642	**6.09**	**/**	**6.14**
PSPTO_5643	hypothetical protein PSPTO_5643	**9.48**	**/**	**8.67**
PSPTO_5645	hypothetical protein PSPTO_5645	**2.58**	**/**	**2.62**
PSPTO_5646	hypothetical protein PSPTO_5646	**/**	**/**	**1.97**
PSPTO_5652	hypothetical protein	**-22.19**	**-6.25**	**-3.55**
PSPTO_5660	hypothetical protein	**5.04**	**/**	**5.62**
PSPTO_5664	hypothetical protein	**2.30**	**/**	**/**
PSPTO_5669	hypothetical protein	**-2.29**	**/**	**-2.56**
PSPTO_5670	hypothetical protein	**1.90**	**2.19**	**/**
PSPTO_5671	hypothetical protein	**-9.83**	**/**	**/**
PSPTO_5672	hypothetical protein	**-4.26**	**/**	**/**
PSPTO_5674	hypothetical protein	**-14.47**	**/**	**-10.20**
PSPTO_5675	hypothetical protein	**-7.59**	**-4.53**	**/**
PSPTO_B0005	hypothetical protein	**-21.57**	**-2.05**	**-10.54**
PSPTO_B0009	hypothetical protein	**2.79**	**/**	**2.89**
PSPTO_B0012	hypothetical protein	**1.92**	**/**	**/**
PSPTO_B0015	hypothetical protein	**/**	**/**	**2.19**
PSPTO_B0023	hypothetical protein	**4.16**	**2.09**	**1.99**

PSPTO_B0031	hypothetical protein	**2.26**	**/**	**/**
PSPTO_B0032	hypothetical protein	**/**	**/**	**-2.40**
PSPTO_B0033	hypothetical protein	**/**	**/**	**-2.06**
PSPTO_B0043	hypothetical protein	**2.21**	**/**	**/**
PSPTO_B0071	hypothetical protein	**3.23**	**2.04**	**/**
PSPTOA0001	hypothetical protein	**-2.80**	**/**	**-2.45**
PSPTOA0006		**2.07**	**/**	**2.20**
PSPTOA0007	hypothetical protein	**-8568.22**	**-7111.02**	**/**
PSPTOA0008	hypothetical protein	**-3823.01**	**-6763.33**	**/**
PSPTOA0009	hypothetical protein	**-3142.96**	**-7395.60**	**2.35**
PSPTOA0010	hypothetical protein	**-2.29**	**/**	**-2.38**
PSPTOA0012	hypothetical protein	**-8.45**	**/**	**-5.99**
PSPTOA0017	hypothetical protein	**-2.70**	**/**	**-1.95**
PSPTOA0018	hypothetical protein	**-3.14**	**/**	**-3.04**
PSPTOA0019	hypothetical protein	**-2.73**	**/**	**-2.45**
PSPTOA0027	hypothetical protein	**-2.14**	**/**	**/**
PSPTOA0030	hypothetical protein	**-2.09**	**/**	**-2.07**
PSPTOA0032	hypothetical protein	**/**	**/**	**-1.85**
PSPTOA0033	hypothetical protein	**3.53**	**/**	**3.03**
PSPTOA0035	hypothetical protein	**3.66**	**/**	**2.13**
PSPTOA0036	hypothetical protein	**2.89**	**2.23**	**/**
PSPTOA0037	hypothetical protein	**3.15**	**2.57**	**/**
PSPTOA0038	hypothetical protein	**3.46**	**2.28**	**/**
PSPTOA0039	hypothetical protein	**/**	**1.95**	**-1.98**
PSPTOA0065	hypothetical protein	**2.11**	**/**	**/**

Table S3. List of differentially expressed genes (DEGs) in *Pst*DC3000, the *rsmA3* mutant, and the *rsmA23* double mutant in King's medium B with |FC| value ≥1.5 and an adjusted p-value <0.05.

Locus tag	Description	Δ*rsmA23* /*Pst*DC3000	Δ*rsmA23* /Δ*rsmA3*	Δ*rsmA3* /*Pst*DC3000
**Type III secretion system**				
PSPTO_0044	type III effector HopK1	/	**2.84**	**-2.42**
PSPTO_0852	type III helper protein HopAJ1	**3.22**	/	**2.68**
PSPTO_0905	type III effector HopAH1	**2.25**	/	/
PSPTO_1381	type III helper protein HrpA1	**-2.17**	/	/
PSPTO_1391	negative regulator of hrp expression HrpV	**6.34**	**3.07**	/
PSPTO_1404	RNA polymerase sigma factor HrpL	**-11.25**	**-5.69**	/
PSPTO_1568	type III effector HopAF1	/	/	**-3.08**
PSPTO_3292	type III effector HopAH2-1	**-2.67**	**-6.20**	**2.32**
PSPTO_3293	type III effector HopAH2-2	/	**-1.88**	**1.81**
PSPTO_4718	type III effector HopAA1-2	/	/	**2.45**
PSPTO_4722	type III effector HopAO1	/	/	**-2.24**
**Translation, ribosomal structure and biogenesis**				
PSPTO_0077	ribonuclease PH	/	/	**-1.69**
PSPTO_0089	50S ribosomal protein L28	**-1.96**	/	**-3.11**
PSPTO_0090	50S ribosomal protein L33	/	/	**-3.09**
PSPTO_0102	endoribonuclease L-PSP family protein	/	**-3.44**	**3.65**
PSPTO_0178	methionyl-tRNA formyltransferase	/	/	**-2.31**
PSPTO_0179	sun protein	/	/	**-2.41**
PSPTO_0184	glycyl-tRNA synthetase subunit alpha	/	**1.95**	**-1.77**
PSPTO_0185	glycyl-tRNA synthetase subunit beta	/	**1.97**	**-1.79**
PSPTO_0539	30S ribosomal protein S21	**-1.89**	/	**-2.92**
PSPTO_0551	dimethyladenosine transferase	/	**2.26**	**-3.98**
PSPTO_0615	50S ribosomal protein L11	**-1.90**	**1.79**	**-3.40**
PSPTO_0616	50S ribosomal protein L1	**-1.94**	**1.87**	**-3.62**
PSPTO_0617	50S ribosomal protein L10	**-2.34**	**2.38**	**-5.58**
PSPTO_0618	50S ribosomal protein L7/L12	**-2.29**	**2.88**	**-6.58**
PSPTO_0621	30S ribosomal protein S12	/	**1.74**	**-2.79**
PSPTO_0622	30S ribosomal protein S7	/	**1.91**	**-3.18**
PSPTO_0623	translation elongation factor G	/	**1.95**	**-3.12**
PSPTO_0624	translation elongation factor Tu	/	**2.08**	**-3.49**
PSPTO_0625	30S ribosomal protein S10	/	**1.83**	**-3.07**
PSPTO_0626	50S ribosomal protein L3	/	**1.98**	**-3.44**
PSPTO_0627	50S ribosomal protein L4	**-1.77**	**2.15**	**-3.81**
PSPTO_0628	50S ribosomal protein L23	**-1.89**	**2.01**	**-3.78**
PSPTO_0629	50S ribosomal protein L2	/	**2.11**	**-3.34**
PSPTO_0630	30S ribosomal protein S19	/	**2.08**	**-3.63**
PSPTO_0631	50S ribosomal protein L22	/	**2.14**	**-3.65**
PSPTO_0632	30S ribosomal protein S3	/	**2.43**	**-3.67**
PSPTO_0633	50S ribosomal protein L16	/	**2.73**	**-4.10**
PSPTO_0634	50S ribosomal protein L29	/	**3.13**	**-4.63**
PSPTO_0635	30S ribosomal protein S17	/	**3.21**	**-4.94**
PSPTO_0636	50S ribosomal protein L14	/	**3.23**	**-5.04**
PSPTO_0637	50S ribosomal protein L24	/	**3.18**	**-4.98**
PSPTO_0638	50S ribosomal protein L5	/	**3.28**	**-5.92**

PSPTO_0639	30S ribosomal protein S14	/	**2.93**	**-5.13**
PSPTO_0640	30S ribosomal protein S8	/	**2.11**	**-3.52**
PSPTO_0641	50S ribosomal protein L6	/	**2.32**	**-3.73**
PSPTO_0642	50S ribosomal protein L18	/	**2.44**	**-3.75**
PSPTO_0643	30S ribosomal protein S5	/	**2.54**	**-3.98**
PSPTO_0644	50S ribosomal protein L30	**-1.81**	**2.47**	**-4.46**
PSPTO_0645	50S ribosomal protein L15	/	**2.52**	**-4.34**
PSPTO_0648	30S ribosomal protein S13	**-1.76**	**2.33**	**-4.10**
PSPTO_0649	30S ribosomal protein S11	/	**2.34**	**-4.21**
PSPTO_0650	30S ribosomal protein S4	**-1.79**	**2.29**	**-4.10**
PSPTO_0652	50S ribosomal protein L17	**-1.91**	**2.69**	**-5.13**
PSPTO_0797	50S ribosomal protein L21	**-1.85**	**1.86**	**-3.43**
PSPTO_0802	30S ribosomal protein S20	/	/	**-2.40**
PSPTO_0806	isoleucyl-tRNA synthetase	/	/	**-1.92**
PSPTO_0963	poly(A) polymerase	**-2.08**	/	**-2.23**
PSPTO_1101	GTP-binding protein YchF	**-2.10**	**2.13**	**-4.47**
PSPTO_1102	peptidyl-tRNA hydrolase	**-2.08**	**1.87**	**-3.88**
PSPTO_1103	ribosomal 5S rRNA E-loop binding protein Ctc/L25/TL5	**-1.98**	**2.19**	**-4.34**
PSPTO_1268	valyl-tRNA synthetase	/	/	**-1.77**
PSPTO_1361	allophanate hydrolase	**3.12**	/	**3.44**
PSPTO_1412	S-adenosylmethionine--tRNA ribosyltransferase-isomerase	**-2.03**	/	**-2.23**
PSPTO_1413	queuine tRNA-ribosyltransferase	**-1.86**	/	**-2.56**
PSPTO_1420	RNA methyltransferase, TrmH family, group 1	/	/	**-1.98**
PSPTO_1435	histidyl-tRNA synthetase	/	/	**-1.86**
PSPTO_1457	cytidine/deoxycytidylate deaminase family protein	/	**-2.41**	/
PSPTO_1473	30S ribosomal protein S16	/	**1.82**	**-2.99**
PSPTO_1474	16S rRNA-processing protein RimM	/	/	**-2.99**
PSPTO_1475	tRNA (guanine-N1)-methyltransferase	**-1.76**	**2.07**	**-3.65**
PSPTO_1476	50S ribosomal protein L19	/	**2.22**	**-3.76**
PSPTO_1500	hypothetical protein PSPTO_1500	/	/	**-2.07**
PSPTO_1501	lysyl-tRNA synthetase	/	**2.20**	**-3.31**
PSPTO_1534	30S ribosomal protein S2	/	/	**-1.79**
PSPTO_1535	translation elongation factor Ts	/	/	**-2.10**
PSPTO_1537	ribosome recycling factor	/	**1.99**	**-2.82**
PSPTO_1598	polypeptide deformylase	**1.99**	/	/
PSPTO_1693	23S rRNA m(5)U1939 methyltransferase	**-1.99**	/	/
PSPTO_1750	30S ribosomal protein S1	**-1.91**	/	**-3.28**
PSPTO_1765	translation elongation factor P	/	/	**-1.90**
PSPTO_1815	ribosomal large subunit pseudouridine synthase B	/	/	**-2.10**
PSPTO_1818	hypothetical protein PSPTO_1818	/	/	**-2.11**
PSPTO_2169	dihydrouridine synthase family protein	/	/	**-1.96**
PSPTO_2310	ribosome modulation factor-related protein	/	**-4.30**	**4.55**
PSPTO_2311	23S rRNA m(2)G2445 methyltransferase	/	/	**-1.96**
PSPTO_2378	threonyl-tRNA synthetase	**-2.21**	/	**-2.84**
PSPTO_2379	translation initiation factor IF-3	**-3.14**	/	**-3.04**
PSPTO_2380	50S ribosomal protein L35	**-2.95**	/	**-3.10**
PSPTO_2381	50S ribosomal protein L20	**-2.71**	/	**-3.11**

PSPTO_2382	phenylalanyl-tRNA synthetase subunit alpha	**-1.91**	/	**-3.03**
PSPTO_2383	phenylalanyl-tRNA synthetase subunit beta	/	**2.18**	**-3.47**
PSPTO_3345	seryl-tRNA synthetase	/	**2.02**	**-2.40**
PSPTO_3352	translation initiation factor IF-1	**-2.11**	/	**-3.76**
PSPTO_3358	tRNA (5-methyl aminomethyl-2-thiouridylate)-methyltransferase	**-1.88**	/	**-2.53**
PSPTO_3750	hypothetical protein PSPTO_3750	**-1.83**	/	**-2.10**
PSPTO_3817	tRNA pseudouridine synthase A	/	/	**-2.89**
PSPTO_3835	50S ribosomal protein L32	**-1.88**	/	**-2.69**
PSPTO_3840	ribosomal large subunit pseudouridine synthase C	/	/	**-1.92**
PSPTO_3841	ribonuclease, Rne/Rng family protein	/	/	**-2.00**
PSPTO_3875	ribosomal large subunit pseudouridine synthase A	/	/	**-2.19**
PSPTO_3916	hypothetical protein PSPTO_3916	/	/	**-2.00**
PSPTO_4019	ribosomal protein S12 methylthiotransferase	/	**1.88**	**-1.93**
PSPTO_4134	hypothetical protein PSPTO_4134	**2.21**	/	/
PSPTO_4147	methionyl-tRNA synthetase	/	/	**-2.14**
PSPTO_4158	ribonuclease T	/	/	**-2.46**
PSPTO_4183	50S ribosomal protein L31 type B	/	/	**2.51**
PSPTO_4204	hypothetical protein PSPTO_4204	**-2.56**	**-3.23**	/
PSPTO_4425	30S ribosomal protein S9	/	/	**-3.04**
PSPTO_4426	50S ribosomal protein L13	/	/	**-2.84**
PSPTO_4468	ribonuclease G	/	**2.38**	**-2.57**
PSPTO_4473	aspartyl/glutamyl-tRNA amidotransferase subunit C	/	/	**-1.95**
PSPTO_4474	aspartyl/glutamyl-tRNA amidotransferase subunit A	/	/	**-1.81**
PSPTO_4475	aspartyl/glutamyl-tRNA amidotransferase subunit B	/	/	**-2.00**
PSPTO_4486	polyribonucleotide nucleotidyltransferase	/	**2.04**	**-3.22**
PSPTO_4487	30S ribosomal protein S15	/	/	**-2.22**
PSPTO_4488	tRNA pseudouridine synthase B	**-2.06**	**2.79**	**-5.76**
PSPTO_4489	ribosome-binding factor A	**-1.87**	**3.66**	**-6.83**
PSPTO_4490	translation initiation factor IF-2	/	**3.01**	**-3.88**
PSPTO_4498	FtsJ cell division protein	**-2.04**	**1.84**	**-3.75**
PSPTO_4579	peptide chain release factor 3	/	**1.84**	**-2.54**
PSPTO_4654	tRNA (uracil-5-)-methyltransferase	/	**1.93**	**-3.12**
PSPTO_4862	ribosomal protein L11 methyltransferase	/	/	**-1.83**
PSPTO_4930	50S ribosomal protein L9	/	**2.64**	**-4.40**
PSPTO_4932	30S ribosomal protein S18	/	**2.29**	**-3.98**
PSPTO_4933	30S ribosomal protein S6	/	**2.16**	**-3.75**
PSPTO_5120	amidase family protein	/	/	**-2.24**
PSPTO_5136	50S ribosomal protein L31	**-1.87**	**1.80**	**-3.36**
PSPTO_5138	arginyl-tRNA synthetase	/	/	**-2.25**
PSPTO_5163	D-tyrosyl-tRNA(Tyr) deacylase	/	**-1.94**	/
PSPTO_5559	hypothetical protein PSPTO_5559	**9.96**	/	**16.69**
PSPTO_5615	50S ribosomal protein L34	/	/	**-2.10**
**Transcription**				
PSPTO_0032	hypothetical protein PSPTO_0032	/	**-2.07**	/
PSPTO_0033	ParB family protein	/	**-2.62**	/
PSPTO_0074	DNA-directed RNA polymerase subunit omega	**-1.78**	/	**-2.56**
PSPTO_0105	DNA-binding protein	/	**-2.31**	**2.36**

PSPTO_0214	virulence-associated protein	**2.46**	/	**2.23**
PSPTO_0261	AsnC family transcriptional regulator	/	/	**2.23**
PSPTO_0281	hypothetical protein PSPTO_0281	**2.80**	/	/
PSPTO_0327	S1 RNA binding domain-containing protein	/	/	**-1.94**
PSPTO_0440	regulatory protein BetI	/	**2.65**	/
PSPTO_0465	AraC family transcriptional regulator	/	/	**5.25**
PSPTO_0537	RNA polymerase sigma-70 factor	/	/	**-2.19**
PSPTO_0611	pantothenate kinase	/	/	**-2.42**
PSPTO_0614	transcription antitermination protein NusG	/	/	**-2.69**
PSPTO_0619	DNA-directed RNA polymerase subunit beta	/	**3.25**	**-5.83**
PSPTO_0620	DNA-directed RNA polymerase subunit beta\'	**-2.11**	/	**-3.81**
PSPTO_0694	N utilization substance protein B	/	**1.77**	**-2.49**
PSPTO_0749	heavy metal-dependent transcriptional regulator	**-2.22**	/	**-2.11**
PSPTO_0853	dnaK suppressor protein	/	**-2.74**	**4.03**
PSPTO_1120	hypothetical protein PSPTO_1120	/	**-2.41**	**5.96**
PSPTO_1151	LuxR family transcriptional regulator	/	/	**2.45**
PSPTO_1216	LysR family transcriptional regulator	**-2.63**	**-1.96**	/
PSPTO_1286	RNA polymerase sigma-70 family protein	**-4.38**	**-3.32**	/
PSPTO_1322	GNAT family acetyltransferase	**-3.46**	**-2.29**	/
PSPTO_1422	rrf2 family protein	/	/	**-1.83**
PSPTO_1565	RNA polymerase sigma-38 factor	**9.55**	/	**6.35**
PSPTO_1613	LuxR family transcriptional regulator	/	/	**-2.21**
PSPTO_1618	LysR family transcriptional regulator	/	/	**2.03**
PSPTO_1704	NAD-dependent deacetylase	**-2.01**	/	**-2.17**
PSPTO_1758	TetR family transcriptional regulator	**-4.66**	**-2.27**	**-2.05**
PSPTO_1768	MarR family transcriptional regulator	**-3.91**	**-4.00**	/
PSPTO_1831	transcriptional regulator ArgR	/	**2.01**	**-2.97**
PSPTO_1873	GntR family transcriptional regulator	/	**-2.09**	**2.85**
PSPTO_1925	negative regulator of flagellin synthesis FlgM	**-2.64**	/	**-1.84**
PSPTO_1954	transcriptional regulator FleQ	**-2.81**	**-2.09**	/
PSPTO_1991	transcriptional activator Anr	**4.92**	/	**4.00**
PSPTO_2121	RulA protein	**2.11**	/	/
PSPTO_2133	RNA polymerase sigma-70 family protein	/	**-5.56**	**7.15**
PSPTO_2190	GntR family transcriptional regulator	/	**-1.80**	/
PSPTO_2286	GntR family transcriptional regulator	**4.56**	/	**4.93**
PSPTO_2370	ribose operon repressor	**-3.01**	/	**-3.02**
PSPTO_2444	GNAT family acetyltransferase	/	/	**-2.22**
PSPTO_2505	AraC family transcriptional regulator	**2.77**	/	**2.08**
PSPTO_2508	hypothetical protein PSPTO_2508	/	/	**2.37**
PSPTO_2606	AraC family transcriptional regulator	/	**-1.95**	/
PSPTO_2708	transcriptional activator MltR	/	**-1.86**	/
PSPTO_2743	MerR family transcriptional regulator	/	**-2.93**	**2.97**
PSPTO_2751	LysR family transcriptional regulator	/	/	**2.40**
PSPTO_2780	IclR family transcriptional regulator	/	**1.90**	/
PSPTO_2828	transcriptional regulator SyrR	**5.02**	/	**9.82**
PSPTO_2833	LuxR family transcriptional regulator	**3.85**	/	**3.54**
PSPTO_2906	transcriptional regulator VanR	**-2.04**	**-1.97**	/
PSPTO_2923	nitrogen assimilation transcriptional regulator	/	/	**3.08**

PSPTO_3017	LacI family transcriptional regulator	/	/	**2.26**
PSPTO_3025	DNA-binding protein	/	**-1.99**	**2.93**
PSPTO_3074	AraC family transcriptional regulator	/	**-2.25**	/
PSPTO_3191	DeoR family transcriptional regulator	/	/	**-2.03**
PSPTO_3196	LysR family transcriptional regulator	**2.31**	/	**2.76**
PSPTO_3265	LysR family transcriptional regulator	/	**-1.96**	**2.16**
PSPTO_3355	cold shock domain family protein	**3.05**	/	**4.71**
PSPTO_3563	gluconate utilization system GNT-I transcriptional repressor	/	/	**1.92**
PSPTO_3617	MarR family transcriptional regulator	/	/	**3.09**
PSPTO_3877	GntR family transcriptional regulator	**4.28**	**-3.01**	**12.86**
PSPTO_4020	GNAT family acetyltransferase	**2.37**	/	/
PSPTO_4104	ATP-dependent helicase HepA	/	**1.91**	**-2.99**
PSPTO_4192	hypothetical protein PSPTO_4192	/	/	**2.24**
PSPTO_4217	ribonuclease III	**-1.98**	**1.99**	**-3.95**
PSPTO_4250	DNA-binding protein	/	**-2.25**	**3.48**
PSPTO_4267	TetR family transcriptional regulator	**-2.18**	/	/
PSPTO_4302	TetR family transcriptional regulator	/	/	**-2.51**
PSPTO_4442	toluene tolerance protein	/	/	**-2.43**
PSPTO_4491	N utilization substance protein A	/	**2.11**	**-3.22**
PSPTO_4500	transcription elongation factor GreA	/	**2.66**	**-4.22**
PSPTO_4601	transcription elongation factor GreB	**2.35**	/	/
PSPTO_4644	LuxR family transcriptional regulator	**5.02**	/	**3.76**
PSPTO_4997	hypothetical protein PSPTO_4997	/	/	**-2.50**
PSPTO_5146	transcriptional regulator PhaD	**7.36**	/	**4.28**
PSPTO_5176	RNA polymerase sigma-70 family protein	**3.74**	/	**2.46**
PSPTO_5177	hypothetical protein PSPTO_5177	**4.45**	**2.01**	**2.21**
PSPTO_5242	transcription termination factor Rho	**-1.78**	/	**-2.95**
**Replication, recombination and repair**				
PSPTO_0046	hypothetical protein PSPTO_0046	/	/	**3.02**
PSPTO_0047	UvrD/REP helicase family protein	**2.64**	**-2.11**	**5.56**
PSPTO_0086	DNA repair protein RadC	**19.64**	**2.33**	**8.42**
PSPTO_0175	DNA processing protein DprA	**3.04**	/	**2.39**
PSPTO_0284	hypothetical protein PSPTO_0284	/	/	**3.46**
PSPTO_0470	exonuclease	**-6.17**	**-4.97**	/
PSPTO_0538	DNA primase	**-2.38**	/	**-3.56**
PSPTO_0656	single-stranded DNA-binding protein	/	/	**-1.89**
PSPTO_0776	exodeoxyribonuclease V subunit gamma	**-1.93**	/	**-2.36**
PSPTO_0777	exodeoxyribonuclease V subunit beta	/	/	**-2.18**
PSPTO_0778	exodeoxyribonuclease V subunit alpha	/	/	**-2.15**
PSPTO_1084	integrase/recombinase XerD	/	/	**2.43**
PSPTO_1121	deoxyribodipyrimidine photolyase	/	**-2.29**	**4.05**
PSPTO_1455	hypothetical protein PSPTO_1455	**9.22**	**1.86**	**4.97**
PSPTO_1464	mutT/nudix family protein	/	**-1.88**	**2.76**
PSPTO_1488	single-stranded-DNA-specific exonuclease RecJ	/	/	**-1.96**
PSPTO_1548	ribonuclease HII	**-2.74**	/	**-4.00**
PSPTO_1549	DNA polymerase III subunit alpha	**-2.48**	/	**-2.79**
PSPTO_1587	ATP-dependent RNA helicase SrmB	/	/	**-1.80**
PSPTO_1775	ATP-dependent RNA helicase, DEAD box family	/	/	**-3.13**

PSPTO_2023	exonuclease	**2.96**	/	/
PSPTO_2099	helicase/SNF2 family domain protein	/	**1.79**	**-2.96**
PSPTO_2164	excinuclease ABC subunit B	/	/	**2.44**
PSPTO_2364	endonuclease I	/	/	**-1.87**
PSPTO_2860	helicase domain-containing protein	**5.88**	/	**9.87**
PSPTO_2976	DNA topoisomerase, type I	**2.95**	/	/
PSPTO_3357	mutT/nudix family protein	**-2.54**	/	**-2.73**
PSPTO_3385	site-specific recombinase, phage integrase family	/	/	**2.21**
PSPTO_3388	DNA adenine methylase	**-18.27**	**-2.50**	**-7.32**
PSPTO_3427	C-5 cytosine-specific DNA methylase family protein	**-3.16**	/	**-3.07**
PSPTO_3464	ATP-dependent DNA ligase	**3.42**	/	**5.15**
PSPTO_3465	KU domain protein	**4.75**	/	**4.97**
PSPTO_3514	DNA topoisomerase I	/	/	**-1.93**
PSPTO_3765	exonuclease SbcD	/	/	**-2.70**
PSPTO_3766	exonuclease SbcC	**-2.06**	/	**-2.04**
PSPTO_3930	retron reverse transcriptase	/	/	**3.20**
PSPTO_3977	Holliday junction DNA helicase RuvB	/	/	**-2.07**
PSPTO_3979	crossover junction endodeoxyribonuclease RuvC	/	/	**-2.47**
PSPTO_4058	DNA mismatch repair protein MutS	/	/	**-2.27**
PSPTO_4095	ATP-dependent helicase HrpA	/	/	**-1.94**
PSPTO_4120	DinG family ATP-dependent helicase	/	/	**-1.86**
PSPTO_4135	ATP-dependent DNA ligase	/	/	**2.32**
PSPTO_4181	mutT/nudix family protein	/	/	**-2.30**
PSPTO_4215	DNA repair protein RecO	**-2.18**	**2.63**	**-5.74**
PSPTO_4316	exodeoxyribonuclease I	**-2.13**	/	**-2.04**
PSPTO_4604	site-specific recombinase, phage integrase family	/	**-2.68**	**4.08**
PSPTO_4665	exonuclease	**-1.89**	/	/
PSPTO_4745	ATP-dependent helicase HrpB	**3.77**	**2.54**	/
PSPTO_4751	UvrD/REP helicase family protein	/	**-1.91**	**2.27**
PSPTO_4814	DNA polymerase III subunit delta	/	/	**-2.49**
PSPTO_4929	replicative DNA helicase	**-1.78**	**1.99**	**-3.54**
PSPTO_4960	DNA topoisomerase IV subunit A	/	**1.94**	**-2.25**
PSPTO_4997	hypothetical protein PSPTO_4997	/	/	**-2.50**
PSPTO_5007	ATP-independent RNA helicase DbpA	/	/	**-1.77**
PSPTO_5070	ATP-dependent RNA helicase rhlE	/	/	**-2.02**
PSPTO_5135	staphylococcal nuclease-like protein	/	/	**-2.94**
**Chromatin structure and dynamics**				
PSPTO_4662	histone deacetylase family protein	/	/	**2.11**
**Cell cycle control, cell division, chromosome partitioning**				
PSPTO_0855	ParA family protein	**2.79**	/	**3.13**
PSPTO_1026	cell morphology protein	/	/	**-2.55**
PSPTO_1986	ParA family protein	/	**-2.68**	**2.03**
PSPTO_3349	cell division protein FtsK	/	/	**-1.97**
PSPTO_4409	cell division protein FtsW	**-1.95**	/	**-2.60**
PSPTO_4469	maf protein	/	/	**-2.23**
PSPTO_5090	ParA family protein	/	/	**2.14**
PSPTO_5139	hypothetical protein PSPTO_5139	/	/	**-2.95**
**Defense mechanisms**				

PSPTO_0008	HsdR family type I site-specific deoxyribonuclease	**2.45**	/	**1.81**
PSPTO_0115	multidrug efflux protein NorA	**5.04**	**-1.94**	**9.78**
PSPTO_1076	O-antigen ABC transporter, permease protein	/	/	**-1.95**
PSPTO_1089	type I restriction-modification enzyme, R subunit	**1.79**	/	/
PSPTO_1653	hypothetical protein PSPTO_1653	/	/	**2.26**
PSPTO_2428	multidrug resistance protein NorM	/	/	**1.95**
PSPTO_2593	multidrug resistance protein, AcrA/AcrE family	**3.41**	/	**3.27**
PSPTO_2875	ABC transporter ATP-binding protein	/	/	**5.00**
PSPTO_2876	ABC transporter permease	**3.58**	**-2.19**	**7.86**
PSPTO_3100	aliphatic isothiocyanate resistance protein SaxF; AcrB/AcrD/AcrF family	**2.09**	/	/
PSPTO_3141	undecaprenyl pyrophosphate phosphatase	/	/	**-1.93**
PSPTO_3330	ABC transporter ATP-binding protein	**1.93**	/	/
PSPTO_3620	HlyD family secretion protein	/	**-2.32**	**3.15**
PSPTO_4303	RND family efflux transporter MFP subunit	/	**2.11**	**-2.02**
PSPTO_4304	isothiocyanate resistance protein SaxB; isochorismatase family	/	**2.86**	**-2.39**
PSPTO_4984	lipid A ABC transporter, ATP-binding/permease protein	**-1.77**	/	**-1.93**
**Signal transduction mechanisms**				
PSPTO_0127	alginate biosynthesis regulatory protein AlgR	**1.96**	/	/
PSPTO_0236	EAL domain-containing protein	**2.17**	/	/
PSPTO_0304	sensory box/GGDEF domain protein	**3.37**	**2.01**	/
PSPTO_0306	sensory box/GGDEF domain/EAL domain-containing protein	/	/	**-1.96**
PSPTO_0313	cyclic nucleotide-binding protein	/	**-1.94**	**2.23**
PSPTO_0352	nitrogen regulation protein NR(I)	/	/	**3.40**
PSPTO_0353	nitrogen regulation protein NtrB	/	/	**2.86**
PSPTO_0361	GTP-binding protein TypA	/	**2.57**	**-3.86**
PSPTO_0378	DNA-binding heavy metal response regulator	/	/	**1.97**
PSPTO_0406	sensory box/GGDEF domain/EAL domain-containing protein	/	**-2.31**	/
PSPTO_0445	regulatory protein	/	/	**2.98**
PSPTO_0466	methyl-accepting chemotaxis protein	**-4.07**	/	**-3.41**
PSPTO_0471	nucleotidyltransferase	**-15.07**	**-10.94**	/
PSPTO_0505	diguanylate cyclase	**-2.47**	/	/
PSPTO_0536	sensory box/GGDEF domain/EAL domain-containing protein	**-3.12**	**-6.34**	**2.03**
PSPTO_0547	hypothetical protein PSPTO_0547	**2.72**	/	**4.38**
PSPTO_0786	DNA-binding response regulator	/	**-3.58**	/
PSPTO_0908	protein-glutamate methylesterase CheB	**5.17**	/	**4.82**
PSPTO_0909	chemoreceptor glutamine deamidase CheD	**5.32**	/	**6.28**
PSPTO_0910	chemotaxis protein methyltransferase CheR	**5.44**	/	**8.89**
PSPTO_0911	chemotaxis protein CheW	**7.67**	/	**11.76**
PSPTO_0912	methyl-accepting chemotaxis protein	**5.50**	**-2.19**	**12.03**
PSPTO_0913	chemotaxis sensor histidine kinase CheA	**2.49**	**-2.84**	**7.08**
PSPTO_0915	chemotaxis protein CheY	**2.26**	**-2.16**	**4.88**
PSPTO_0946	tellurium resistance protein	**1.75**	/	/
PSPTO_1152	HDIG domain protein	/	/	**2.35**
PSPTO_1246	PhoH-like protein	**5.59**	/	**9.40**
PSPTO_1278	sensory box protein/response regulator	**-2.10**	/	/
PSPTO_1290	DNA-binding response regulator	**-2.48**	/	/

PSPTO_1291	sensor histidine kinase	**-3.10**	/	**-2.50**
PSPTO_1323	chemotaxis protein CheV	**-2.92**	**-2.15**	/
PSPTO_1490	sensory box histidine kinase/response regulator	**2.96**	/	/
PSPTO_1494	chemotaxis protein CheW	**-2.55**	/	/
PSPTO_1495	chemotaxis protein methyltransferase CheR	**-2.62**	**-1.95**	/
PSPTO_1496	chemotaxis protein CheW	**-2.72**	**-2.22**	/
PSPTO_1497	sensor histidine kinase/response regulator	**-2.32**	/	/
PSPTO_1667	universal stress protein family	/	**-4.29**	**8.19**
PSPTO_1672	DNA-binding response regulator	/	**-2.03**	**2.26**
PSPTO_1739	diguanylate cyclase	**1.89**	**2.50**	/
PSPTO_1803	sensor histidine kinase	/	/	**-1.83**
PSPTO_1844	carbon storage regulator	**-11.39**	**-6.16**	/
PSPTO_1870	sensory box histidine kinase/response regulator	**2.52**	/	/
PSPTO_1910	hypothetical protein PSPTO_1910	/	**1.96**	/
PSPTO_1911	response regulator/TPR domain protein	**-2.94**	/	**-1.95**
PSPTO_1912	sensor histidine kinase	**-2.88**	/	/
PSPTO_1927	chemotaxis protein CheV	**-3.15**	/	**-1.80**
PSPTO_1928	chemotaxis protein methyltransferase CheR	**-3.01**	/	**-1.89**
PSPTO_1955	sensor histidine kinase FleS	**2.91**	**-2.97**	**8.65**
PSPTO_1956	sigma-54 dependent transcriptional regulator/response regulator FleR	**2.93**	/	**6.81**
PSPTO_1963	STAS domain-containing protein	/	/	**-2.55**
PSPTO_1964	response regulator	/	/	**-3.31**
PSPTO_1980	chemotaxis protein CheY	/	/	**-1.91**
PSPTO_1981	chemotaxis protein CheZ	/	/	**-2.35**
PSPTO_1982	chemotaxis sensor histidine kinase CheA	/	/	**-2.60**
PSPTO_1983	protein-glutamate methylesterase CheB	**-1.87**	/	**-2.71**
PSPTO_2117	response regulator	**-1.77**	**-2.17**	/
PSPTO_2118	anti-anti-sigma factor	/	**-1.85**	/
PSPTO_2127	universal stress protein family	/	/	**2.19**
PSPTO_2171	sensory box/GGDEF domain/EAL domain-containing protein	/	**-2.11**	/
PSPTO_2222	sensor histidine kinase	/	**-1.89**	/
PSPTO_2259	sigma-54 dependent transcriptional regulator	/	/	**1.96**
PSPTO_2303	serine/threonine protein kinase	/	/	**3.73**
PSPTO_2479	hypothetical protein PSPTO_2479	**6.17**	/	**10.10**
PSPTO_2711	response regulator	**2.04**	/	/
PSPTO_2715	sensor histidine kinase/response regulator	/	/	**-1.96**
PSPTO_2716	response regulator	/	**2.20**	/
PSPTO_2717	sensory box histidine kinase/response regulator	/	/	**2.22**
PSPTO_2907	GGDEF domain/EAL domain protein	**-3.76**	**-3.59**	/
PSPTO_3098	methyl-accepting chemotaxis protein	/	**-14.47**	**9.59**
PSPTO_3111	sensory box histidine kinase	**5.01**	**2.08**	**2.41**
PSPTO_3237	methyl-accepting chemotaxis protein	**-3.65**	**-2.27**	/
PSPTO_3379	methyl-accepting chemotaxis protein	**-1.96**	**-1.85**	/
PSPTO_3487	dnaK suppressor protein	**-1.83**	/	**-3.07**
PSPTO_3524	chemotaxis protein CheV	**-3.03**	**-2.04**	/
PSPTO_3526	LuxR family DNA-binding response regulator	/	**-2.77**	/
PSPTO_3566	carbon storage regulator	**-47.55**	**52.10**	**-2477.20**

PSPTO_3577	methyl-accepting chemotaxis protein	**-2.67**	**-2.13**	/
PSPTO_3680	methyl-accepting chemotaxis protein	**-5.07**	**-2.41**	**-2.11**
PSPTO_3696	sensory box histidine kinase/response regulator	**-2.85**	/	**-1.78**
PSPTO_3699	methyl-accepting chemotaxis protein	**-3.03**	**-2.30**	/
PSPTO_3796	diguanylate cyclase	/	**-1.95**	**2.98**
PSPTO_3864	autoinducer synthesis protein PsyI	**12.81**	/	**15.37**
PSPTO_3886	EAL domain/GGDEF domain protein	**2.51**	**2.29**	/
PSPTO_3900	sensory box histidine kinase/response regulator	/	**-2.42**	/
PSPTO_4079	sensor histidine kinase/response regulator	/	**-3.64**	**4.40**
PSPTO_4106	GGDEF domain/EAL domain protein	**4.48**	/	**7.37**
PSPTO_4175	sensor histidine kinase	**-3.11**	/	**-2.68**
PSPTO_4176	sigma-54 dependent transcriptional regulator/response regulator	**-2.90**	/	**-1.96**
PSPTO_4208	HAMP domain/GGDEF domain/EAL domain protein	**-3.72**	**-2.26**	/
PSPTO_4291	sensor histidine kinase	**-2.33**	/	**-2.82**
PSPTO_4292	sigma-54 dependent transcriptional regulator/response regulator	/	/	**-2.79**
PSPTO_4371	inaA protein	/	/	**-2.26**
PSPTO_4373	sensor histidine kinase ColS	**-1.89**	/	**-2.24**
PSPTO_4531	methyl-accepting chemotaxis protein	**-13.83**	/	**-7.17**
PSPTO_4543	GAF domain/GGDEF domain/EAL domain protein	/	**-2.47**	/
PSPTO_4631	sensory box/GGDEF domain/EAL domain-containing protein	**-3.14**	/	**-2.26**
PSPTO_4638	carbon starvation protein CstA	**-5.06**	**-3.08**	/
PSPTO_4796	sensor histidine kinase	**-7.20**	**-5.12**	/
PSPTO_4833	sensory box histidine kinase	**2.86**	**-2.31**	**6.62**
PSPTO_4836	DNA binding response regulator, LuxR family	**2.78**	**-3.27**	**9.07**
PSPTO_4837	response regulator	/	**-4.63**	**2.97**
PSPTO_4868	sensor histidine kinase/response regulator RetS	/	**2.19**	**-1.97**
PSPTO_4997	hypothetical protein PSPTO_4997	/	/	**-2.50**
PSPTO_4998	lipopolysaccharide biosynthesis protein	/	/	**-2.64**
PSPTO_4999	lipopolysaccharide core biosynthesis protein	/	/	**-2.26**
PSPTO_5014	response regulator/sensory box/GGDEF domain/EAL domain protein	**7.66**	/	**7.23**
PSPTO_5284	phosphoenolpyruvate-protein phosphotransferase PtsP	/	**-2.94**	**1.77**
PSPTO_5422	FHA domain-containing protein	/	**1.92**	/
PSPTO_5477	phosphate regulon transcriptional regulatory protein PhoB	**6.19**	**6.65**	/
PSPTO_5478	phosphate regulon sensor protein phoR	**4.24**	**5.60**	/
PSPTO_5482	response regulator	**-3.98**	**-2.11**	**-1.88**
PSPTO_5573	sensor histidine kinase	/	/	**2.34**
**Cell wall/membrane/envelope biogenesis**				
PSPTO_0067	tonB protein	/	/	**3.88**
PSPTO_0103	alanine racemase	/	**-3.69**	**3.97**
PSPTO_0373	Rhs family protein	**3.08**	/	**3.63**
PSPTO_0679	outer membrane protein OmpW	/	**-2.23**	**3.20**
PSPTO_0732	hypothetical protein PSPTO_0732	**5.13**	**2.79**	**1.84**
PSPTO_0807	lipoprotein signal peptidase	/	**1.95**	**-2.73**
PSPTO_0977	penicillin-binding protein	/	/	**-2.06**
PSPTO_1005	GDP-mannose 4,6-dehydratase	**2.34**	/	**2.65**
PSPTO_1027	cellulose synthase, catalytic subunit	**-2.04**	**2.20**	**-4.49**

PSPTO_1028	cellulose synthase regulator protein	/	**2.24**	**-3.85**
PSPTO_1074	glycosyl transferase family protein	/	**1.88**	**-2.68**
PSPTO_1075	O-antigen ABC transporter, ATP-binding protein	/	/	**-2.04**
PSPTO_1077	dTDP-4-dehydrorhamnose 3,5-epimerase	/	/	**-2.06**
PSPTO_1116	cyclopropane-fatty-acyl-phospholipid synthase	**7.64**	/	**5.92**
PSPTO_1217	outer membrane efflux protein	**-2.60**	**-2.10**	/
PSPTO_1232	alginate biosynthesis protein AlgA	**3.11**	**2.38**	/
PSPTO_1233	alginate biosynthesis protein AlgF	**3.27**	/	/
PSPTO_1234	alginate biosynthesis protein AlgJ	**5.13**	/	**3.83**
PSPTO_1235	alginate biosynthesis protein AlgI	**4.80**	/	**3.04**
PSPTO_1237	alginate biosynthesis protein AlgX	**2.24**	**3.21**	/
PSPTO_1238	alginate biosynthesis protein AlgG	**2.80**	**5.14**	/
PSPTO_1239	alginate biosynthesis protein AlgE	**5.73**	**8.14**	/
PSPTO_1240	alginate biosynthesis protein AlgK	**8.21**	**8.08**	/
PSPTO_1241	alginate biosynthesis protein Alg44	**8.14**	**3.98**	/
PSPTO_1242	alginate biosynthesis protein Alg8	**6.51**	/	**3.71**
PSPTO_1243	GDP-mannose 6-dehydrogenase AlgD	**6.43**	**2.20**	**2.92**
PSPTO_1437	PQQ enzyme repeat domain-containing protein	/	**1.78**	**-2.23**
PSPTO_1542	outer membrane protein	/	/	**-1.70**
PSPTO_1543	outer membrane protein OmpH	/	/	**-2.13**
PSPTO_1544	UDP-3-O	/	**1.85**	**-2.95**
PSPTO_1546	UDP-N-acetylglucosamine acyltransferase	/	**2.02**	**-3.54**
PSPTO_1547	lipid A disaccharide synthase	**-1.98**	**2.02**	**-3.99**
PSPTO_1553	2-dehydro-3-deoxyphosphooctonate aldolase	/	/	**-1.87**
PSPTO_1564	lipoprotein NlpD	**2.12**	/	**2.45**
PSPTO_1706	lipoprotein	/	/	**-1.91**
PSPTO_1918	glycosyl transferase family protein	/	/	**2.38**
PSPTO_1920	DegT/DnrJ/EryC1/StrS family aminotransferase	**2.88**	**-4.11**	**11.83**
PSPTO_1923	hypothetical protein PSPTO_1923	**-1.74**	/	/
PSPTO_1946	glycosyl transferase family protein	**-3.72**	/	**-4.22**
PSPTO_1947	glycosyl transferase family protein	**-2.46**	/	/
PSPTO_2056	membrane protein	/	**2.54**	**-3.54**
PSPTO_2109	lipoprotein releasing system transmembrane protein LolC	/	/	**-2.07**
PSPTO_2189	ompA family protein	/	/	**-2.13**
PSPTO_2228	outer membrane usher protein fimD	/	**2.14**	**-2.18**
PSPTO_2256	transglycosylase	/	**1.97**	/
PSPTO_2481	tonB protein	**-4.35**	/	/
PSPTO_2681	penicillin-binding protein 7	**-1.88**	/	**-1.84**
PSPTO_2733	esterified fatty acid cis/trans isomerase	/	**-2.70**	/
PSPTO_2767	lipopolysaccharide core biosynthesis domain protein	/	/	**2.01**
PSPTO_2899	hypothetical protein PSPTO_2899	**5.83**	/	**3.26**
PSPTO_2900	UDP-glucose 4-epimerase	/	/	**2.95**
PSPTO_3101	outer membrane efflux protein	/	**2.46**	/
PSPTO_3134	glycosyl transferase family protein	**2.81**	/	**2.93**
PSPTO_3188	choline/ethanolamine kinase	/	**2.89**	**-2.68**
PSPTO_3439	DegT/DnrJ/EryC1/StrS family aminotransferase	**2.92**	/	**2.56**
PSPTO_3447	glycoside hydrolase family protein	**6.78**	**2.21**	**3.07**

PSPTO_3448	glycoside hydrolase family protein	**6.17**	/	/
PSPTO_3449	capsular polysaccharide biosynthesis protein	**5.63**	/	/
PSPTO_3450	capsular polysaccharide biosynthesis protein	**6.32**	**2.67**	/
PSPTO_3482	Rhs element Vgr protein	**46.38**	**3.44**	**13.47**
PSPTO_3529	capsular polysaccharide biosynthesis protein PslA	**-3.03**	**-2.27**	/
PSPTO_3530	mannose-1-phosphate guanylyltransferase/mannose-6-phosphate isomerase PslB	**-2.50**	**-2.37**	/
PSPTO_3531	lipoprotein PslD	**-2.54**	**-2.94**	/
PSPTO_3532	exopolysaccharide biosynthesis protein PslE	/	**-2.10**	/
PSPTO_3536	glycosyl transferase, group 1 family protein PslI	**3.08**	/	**2.56**
PSPTO_3537	membrane protein PslJ	**3.29**	/	**2.64**
PSPTO_3621	outer membrane efflux protein	/	/	**3.27**
PSPTO_3648	acid phosphatase	**2.64**	**2.28**	/
PSPTO_3671	hypothetical protein PSPTO_3671	**-2.29**	/	/
PSPTO_3842	UDP-N-acetylenolpyruvoylglucosamine reductase	/	/	**-2.04**
PSPTO_3871	lipid A biosynthesis lauroyl acyltransferase	**-1.91**	/	/
PSPTO_3971	peptidoglycan-associated lipoprotein	/	**1.79**	**-1.90**
PSPTO_4081	Rhs family protein	/	/	**2.19**
PSPTO_4117	ompA family protein	/	**1.98**	/
PSPTO_4122	nucleoside-specific channel-forming protein	**2.59**	/	**4.18**
PSPTO_4191	hypothetical protein PSPTO_4191	/	/	**4.10**
PSPTO_4220	GTP-binding protein LepA	/	/	**-2.32**
PSPTO_4305	outer membrane efflux protein	/	**3.09**	**-3.48**
PSPTO_4385	Rhs element Vgr protein	**2.42**	/	**3.04**
PSPTO_4392	mechanosensitive ion channel family protein	/	**2.19**	**-2.10**
PSPTO_4406	D-alanine--D-alanine ligase	/	/	**-2.18**
PSPTO_4407	UDP-N-acetylmuramate--L-alanine ligase	**-2.07**	/	**-2.59**
PSPTO_4408	UDP-N-acetylglucosamine-N-acetylmuramyl-(pentapeptide) pyrophosphoryl-undecaprenol N-acetylglucosamine transferase	**-2.32**	/	**-2.72**
PSPTO_4410	UDP-N-acetylmuramoylalanine--D-glutamate ligase	/	/	**-2.66**
PSPTO_4411	phospho-N-acetylmuramoyl-pentapeptide-transferase	/	**1.90**	**-2.35**
PSPTO_4441	UDP-N-acetylglucosamine 1-carboxyvinyltransferase	/	/	**-2.39**
PSPTO_4448	sugar isomerase	/	/	**-1.73**
PSPTO_4470	rod shape-determining protein MreD	/	/	**-2.93**
PSPTO_4471	rod shape-determining protein MreC	**-1.89**	/	**-3.03**
PSPTO_4476	rplA family protein	/	/	**-1.93**
PSPTO_4560	outer membrane porin, OprD family	/	**2.61**	**-3.02**
PSPTO_4641	large conductance mechanosensitive channel protein	**2.68**	/	**2.22**
PSPTO_4808	apolipoprotein N-acyltransferase	/	/	**-1.83**
PSPTO_4813	lipoprotein	/	/	**-1.99**
PSPTO_4821	D-alanyl-D-alanine carboxypeptidase	/	/	**-2.15**
PSPTO_4823	membrane-bound lytic murein transglycosylase B	/	**2.15**	**-2.36**
PSPTO_4824	rod-shape-determining protein RodA	/	**1.92**	**-2.55**
PSPTO_4825	penicillin-binding protein	/	/	**-2.47**
PSPTO_4907	hypothetical protein PSPTO_4907	**2.67**	/	/
PSPTO_4987	hypothetical protein PSPTO_4987	**-1.85**	/	**-2.71**
PSPTO_4991	glycoside hydrolase family protein	/	/	**-2.01**

PSPTO_5000	lipopolysaccharide core biosynthesis protein WaaP	/	/	**-2.28**
PSPTO_5001	lipopolysaccharide core biosynthesis protein WaaG	/	/	**-2.60**
PSPTO_5002	lipopolysaccharide heptosyltransferase	**-1.92**	/	**-2.28**
PSPTO_5003	ADP-heptose--LPS heptosyltransferase II	/	/	**-1.81**
PSPTO_5050	metW protein	/	/	**-2.01**
PSPTO_5097	glycosyl transferase family protein	**-3.22**	/	**-4.01**
PSPTO_5098	hypothetical protein PSPTO_5098	**-2.27**	/	**-2.56**
PSPTO_5101	hypothetical protein PSPTO_5101	**-3.09**	/	**-5.01**
PSPTO_5133	penicillin-binding protein	**-2.21**	/	**-2.45**
PSPTO_5193	RND family efflux transporter MFP subunit	**2.02**	**1.94**	/
PSPTO_5391	outer membrane porin, OprD family	/	**1.80**	**-2.87**
PSPTO_5415	Rhs element Vgr protein	**1.85**	/	/
PSPTO_5436	Rhs element Vgr protein	**5.38**	/	**4.10**
PSPTO_5438	Rhs family protein	**2.11**	/	/
PSPTO_5585	UDP-glucose 6-dehydrogenase	**5.41**	**4.54**	/
PSPTO_5586	capsular polysaccharide biosynthesis protein	**7.77**	**3.49**	/
PSPTO_5587	glycosyl transferase family protein	/	**2.44**	/
PSPTO_5595	glucosamine--fructose-6-phosphate aminotransferase	/	**1.94**	**-2.04**
**Cell motility**				
PSPTO_0466	methyl-accepting chemotaxis protein	**-4.07**	/	**-3.41**
PSPTO_0815	type IV pilus-associated protein	**-2.17**	/	/
PSPTO_0909	chemoreceptor glutamine deamidase CheD	**5.32**	/	**6.28**
PSPTO_0910	chemotaxis protein methyltransferase CheR	**5.44**	/	**8.89**
PSPTO_0911	chemotaxis protein CheW	**7.67**	/	**11.76**
PSPTO_0912	methyl-accepting chemotaxis protein	**5.50**	**-2.19**	**12.03**
PSPTO_1039	response regulator	**-1.83**	**-2.07**	/
PSPTO_1323	chemotaxis protein CheV	**-2.92**	**-2.15**	/
PSPTO_1494	chemotaxis protein CheW	**-2.55**	/	/
PSPTO_1495	chemotaxis protein methyltransferase CheR	**-2.62**	**-1.95**	/
PSPTO_1496	chemotaxis protein CheW	**-2.72**	**-2.22**	/
PSPTO_1924	hypothetical protein PSPTO_1924	**-1.79**	/	/
PSPTO_1926	flagellar basal body P-ring biosynthesis protein FlgA	/	**-2.53**	**5.52**
PSPTO_1928	chemotaxis protein methyltransferase CheR	**-3.01**	/	**-1.89**
PSPTO_1933	flagellar basal-body rod protein FlgB	/	**-2.83**	**5.80**
PSPTO_1934	flagellar basal body rod protein FlgC	**2.42**	**-2.28**	**5.50**
PSPTO_1935	basal-body rod modification protein FlgD	**2.55**	/	**4.85**
PSPTO_1936	flagellar hook protein FlgE	/	/	**2.20**
PSPTO_1939	flagellar basal body rod protein FlgF	**3.05**	/	**5.46**
PSPTO_1940	flagellar basal-body rod protein FlgG	**2.63**	/	**4.20**
PSPTO_1941	flagellar L-ring protein FlgH	/	/	**3.33**
PSPTO_1942	flagellar P-ring protein FlgI	/	/	**2.54**
PSPTO_1945	flagellar hook-associated protein FlgL	/	**1.98**	**-2.55**
PSPTO_1949	flagellin	**-5.49**	**-3.70**	/
PSPTO_1950	flagellin FlaG	**-4.75**	**-2.31**	**-2.06**
PSPTO_1951	flagellar hook-associated protein FliD	**-4.39**	**-2.26**	**-1.94**
PSPTO_1952	flagellar protein FliS	**-2.52**	/	/
PSPTO_1957	flagellar hook-basal body complex protein FliE	/	/	**4.70**

PSPTO_1958	flagellar M-ring protein FliF	/	/	**3.32**
PSPTO_1966	flagellar hook-length control protein FliK	/	/	**2.97**
PSPTO_1968	flagellar basal body-associated protein FliL	/	**-2.38**	**4.96**
PSPTO_1969	flagellar motor switch protein FliM	**2.58**	/	**5.44**
PSPTO_1970	flagellar motor switch protein FliN	/	/	**2.53**
PSPTO_1971	flagellar protein FliO	/	/	**2.35**
PSPTO_1976	flagellar biosynthesis protein FlhA	/	**-2.48**	**5.53**
PSPTO_1977	flagellar biosynthesis protein FlhF	/	/	**3.97**
PSPTO_1978	flagellar synthesis regulator FleN	/	/	**2.88**
PSPTO_1984	flagellar motor protein	**-2.92**	/	**-3.47**
PSPTO_1985	motB protein	**-3.49**	/	**-3.23**
PSPTO_2229	chaperone protein PapD	/	/	**-2.88**
PSPTO_2230	type I pilus biogensis protein FimA	/	/	**-2.22**
PSPTO_2526	methyl-accepting chemotaxis protein	/	**-3.96**	**3.01**
PSPTO_3237	methyl-accepting chemotaxis protein	**-3.65**	**-2.27**	/
PSPTO_3524	chemotaxis protein CheV	**-3.03**	**-2.04**	/
PSPTO_3818	hypothetical protein PSPTO_3818	/	/	**1.87**
PSPTO_4156	sodium-type flagellar protein MotY	**-2.89**	**-2.44**	/
PSPTO_4952	flagellar motor protein MotB	**-4.20**	**-2.08**	**-2.03**
PSPTO_4953	flagellar motor protein MotA	**-2.96**	**-1.90**	/
PSPTO_5129	type IV pilus biogenesis protein PilP	/	/	**2.13**
PSPTO_5132	type IV pilus assembly protein PilM	/	**-2.60**	**4.37**
**Intracellular trafficking, secretion, and vesicular transport**				
PSPTO_0068	TonB system transport protein ExbD	/	**-5.30**	**3.69**
PSPTO_0069	TonB system transport protein ExbB	/	**-6.67**	**3.44**
PSPTO_0613	preprotein translocase subunit SecE	/	/	**-2.08**
PSPTO_0672	TonB system transport protein ExbD	/	/	**4.55**
PSPTO_0807	lipoprotein signal peptidase	/	**1.95**	**-2.73**
PSPTO_0810	type IV pilus biogenesis protein	**4.88**	/	**3.08**
PSPTO_0811	pillin	**-2.07**	/	/
PSPTO_0813	hypothetical protein PSPTO_0813	**-2.37**	/	**-2.12**
PSPTO_0815	type IV pilus-associated protein	**-2.17**	/	/
PSPTO_0881	hypothetical protein PSPTO_0881	/	/	**1.83**
PSPTO_0925	type IV pilus biogenesis protein PilC	**2.70**	/	**3.39**
PSPTO_0926	type IV pilus biogenesis protein PilB	**1.84**	**-1.77**	**3.27**
PSPTO_0927	type IV pilus biogenesis protein	/	/	**2.26**
PSPTO_1415	protein-export membrane protein SecD	/	/	**-2.77**
PSPTO_1416	protein-export membrane protein SecF	/	**2.11**	**-3.34**
PSPTO_1924	hypothetical protein PSPTO_1924	**-1.79**	/	/
PSPTO_2229	chaperone protein PapD	/	/	**-2.88**
PSPTO_2230	type I pilus biogensis protein FimA	/	/	**-2.22**
PSPTO_3229	filamentous hemagglutinin, intein-containing	**-3.87**	**-3.19**	/
PSPTO_3230	hemolysin activator protein, HlyB family	**-18.18**	**-9.99**	/
PSPTO_3307	general secretion pathway protein D	/	**2.79**	**-4.48**
PSPTO_3309	general secretion pathway protein M	/	**3.09**	**-2.78**
PSPTO_3310	general secretion pathway protein L	/	**2.57**	/
PSPTO_3363	type II and III secretion system family protein	**-2.43**	/	**-1.94**
PSPTO_3818	hypothetical protein PSPTO_3818	/	/	**1.87**

PSPTO_3972	tolB protein	/	/	**-1.79**
PSPTO_3975	tolQ protein	/	/	**-1.88**
PSPTO_3992	hypothetical protein PSPTO_3992	**-2.09**	/	**-2.11**
PSPTO_4219	signal peptidase I	**-1.72**	/	**-2.96**
PSPTO_4834	hypothetical protein PSPTO_4834	/	/	**3.79**
PSPTO_4850	hypothetical protein PSPTO_4850	/	**-3.60**	**2.23**
PSPTO_4851	type II/III secretion system protein	/	**-3.41**	**5.81**
PSPTO_4852	hypothetical protein PSPTO_4852	/	**-4.93**	**6.42**
PSPTO_4853	type II/IV secretion system protein	/	**-4.69**	**6.15**
PSPTO_4854	hypothetical protein PSPTO_4854	/	**-2.65**	**4.25**
PSPTO_4855	membrane protein	/	/	**4.57**
PSPTO_4856	TPR domain-containing protein	**4.74**	/	**5.28**
PSPTO_5125	hypothetical protein PSPTO_5125	/	/	**-2.68**
PSPTO_5129	type IV pilus biogenesis protein PilP	/	/	**2.13**
PSPTO_5130	type IV pilus biogenesis protein PilO	/	/	**2.87**
PSPTO_5131	type IV pilus biogenesis protein PilN	/	/	**3.08**
PSPTO_5132	type IV pilus assembly protein PilM	/	**-2.60**	**4.37**
PSPTO_5157	sec-independent protein translocase TatC	/	/	**-1.81**
PSPTO_5324	protein-export protein SecB	/	/	**-1.74**
PSPTO_5612	inner membrane protein, 60 kDa	/	/	**-2.42**
**Posttranslational modification, protein turnover, chaperones**				
PSPTO_0426	peptidase, M16 family	/	/	**-1.78**
PSPTO_0540	O-sialoglycoprotein endopeptidase	/	**1.78**	**-1.99**
PSPTO_0553	peptidyl-prolyl cis-trans isomerase SurA	/	/	**-2.16**
PSPTO_0808	FKBP-type peptidyl-prolyl cis-trans isomerase	/	**2.10**	**-2.90**
PSPTO_0829	clpB protein	/	**-2.87**	**2.84**
PSPTO_1178	thioredoxin reductase	/	**-4.80**	**5.37**
PSPTO_1324	disulfide oxidoreductase	/	/	**2.14**
PSPTO_1329	protoheme IX farnesyltransferase	/	**2.19**	**-2.87**
PSPTO_1426	co-chaperone Hsc20	/	**2.17**	**-2.52**
PSPTO_1471	membrane protein	**-1.99**	/	/
PSPTO_1510	hypothetical protein PSPTO_1510	**-2.61**	/	**-2.53**
PSPTO_1649	autotransporter	**12.87**	/	**11.75**
PSPTO_1650	autotransporter	**7.65**	**2.02**	**3.78**
PSPTO_1719	glutathione peroxidase	/	**-1.85**	**2.90**
PSPTO_1767	organic hydroperoxide resistance protein	**-6.10**	/	**-4.53**
PSPTO_1924	hypothetical protein PSPTO_1924	**-1.79**	/	/
PSPTO_2034	hypothetical protein PSPTO_2034	/	/	**-2.13**
PSPTO_2170	heat shock protein, Hsp20 family	/	**-3.37**	/
PSPTO_2336	hypothetical protein PSPTO_2336	/	**-1.95**	**1.97**
PSPTO_2657	xanthine dehydrogenase accessory factor XdhC	/	/	**2.42**
PSPTO_3107	alkyl hydroperoxide reductase subunit F	**2.08**	/	**2.70**
PSPTO_3409	ATP-dependent Clp protease, proteolytic subunit ClpP	**-6.56**	/	**-4.73**
PSPTO_3560	GDA1/CD39 family protein	**2.42**	/	**2.10**
PSPTO_3629	thiol:disulfide interchange protein DsbE	/	/	**-2.23**
PSPTO_3662	xanthine dehydrogenase accessory factor XdhC	**2.49**	**-2.73**	**6.79**
PSPTO_3727	trigger factor	/	/	**-2.33**
PSPTO_3744	peptidyl-prolyl cis-trans isomerase B	/	/	**-1.78**

PSPTO_3856	glutathione S-transferase family protein	**3.12**	/	**2.69**
PSPTO_3890	FKBP-type peptidyl-prolyl cis-trans isomerase	/	**2.08**	/
PSPTO_4376	chaperonin, 60 kDa	**-2.53**	/	/
PSPTO_4377	chaperonin, 10 kDa	**-2.83**	/	/
PSPTO_4424	stringent starvation protein A	**-1.90**	/	**-2.99**
PSPTO_4497	cell division protein FtsH	/	**-2.24**	**1.92**
PSPTO_4581	FKBP-type peptidyl-prolyl cis-trans isomerase	/	/	**-2.41**
PSPTO_4898	heat shock protein YegD	/	**2.06**	**-3.45**
PSPTO_4910	urease accessory protein UreF	/	/	**3.17**
PSPTO_4992	carbamoyltransferase family protein	/	/	**-1.92**
PSPTO_5140	heat shock protein HslV	**-2.27**	/	**-2.44**
PSPTO_5243	thioredoxin	/	/	**2.15**
PSPTO_5317	antioxidant, AhpC/Tsa family	**2.67**	/	**4.48**
PSPTO_5363	DnaJ domain-containing protein	**-2.16**	**-1.99**	/
PSPTO_5425	clpB protein	/	**2.01**	/
**Energy production and conversion**				
PSPTO_0062	citrate transporter	**-4.68**	**-2.55**	/
PSPTO_0092	aldehyde dehydrogenase family protein	/	/	**1.80**
PSPTO_0106	cytochrome c5	/	**-2.26**	/
PSPTO_0107	hypothetical protein PSPTO_0107	**2.34**	/	**2.67**
PSPTO_0233	proton/glutamate symporter	**-4.56**	/	**-4.77**
PSPTO_0248	oxidoreductase, FAD-binding protein	/	/	**2.68**
PSPTO_0402	iron-sulfur cluster-binding protein	/	/	**2.40**
PSPTO_0441	betaine aldehyde dehydrogenase BADH	**1.87**	/	/
PSPTO_0455	glutathione-independent formaldehyde dehydrogenase	/	/	**2.14**
PSPTO_0722	inorganic pyrophosphatase	/	/	**-1.80**
PSPTO_0728	aldehyde dehydrogenase family protein	**-4.13**	**-4.45**	/
PSPTO_0986	membrane protein	/	**-3.17**	**5.78**
PSPTO_1136	malate:quinone oxidoreductase	**-2.05**	**-2.20**	/
PSPTO_1326	cytochrome o ubiquinol oxidase subunit I	/	/	**-1.78**
PSPTO_1327	cytochrome o ubiquinol oxidase subunit III	/	**1.95**	**-2.46**
PSPTO_1328	cytochrome o ubiquinol oxidase subunit IV	/	**2.13**	**-2.67**
PSPTO_1424	iron-binding protein IscU	/	**2.19**	**-2.17**
PSPTO_1428	ferredoxin, 2Fe-2S	/	**2.58**	**-3.27**
PSPTO_1669	propionate kinase	/	**-2.54**	**4.30**
PSPTO_1682	C4-dicarboxylate transport protein	**-5.08**	**-3.53**	/
PSPTO_1731	class II fumarate hydratase	**4.65**	/	**2.64**
PSPTO_1795	alkanesulfonate monooxygenase	/	/	**3.56**
PSPTO_1875	glutathione-dependent formaldehyde dehydrogenase	**8.13**	/	**8.71**
PSPTO_2016	aconitate hydratase 1	**2.06**	/	/
PSPTO_2047	HAD-superfamily hydrolase	/	**2.02**	**-3.17**
PSPTO_2106	soluble pyridine nucleotide transhydrogenase	**-3.38**	/	**-2.04**
PSPTO_2194	citrate synthase I	**-2.17**	/	/
PSPTO_2195	succinate dehydrogenase, cytochrome b556 subunit	/	/	**-1.78**
PSPTO_2196	succinate dehydrogenase, hydrophobic membrane anchor protein	/	/	**-2.26**
PSPTO_2197	succinate dehydrogenase, flavoprotein subunit	/	/	**-2.53**
PSPTO_2198	succinate dehydrogenase, iron-sulfur protein	/	/	**-2.28**

PSPTO_2199	2-oxoglutarate dehydrogenase E1 component	/	**1.90**	**-2.12**
PSPTO_2200	dihydrolipoamide succinyltransferase	/	**2.34**	**-2.91**
PSPTO_2201	dihydrolipoamide dehydrogenase	/	/	**-2.68**
PSPTO_2202	succinyl-CoA synthetase subunit beta	/	/	**-2.08**
PSPTO_2288	2-methylcitrate synthase	**3.32**	/	**2.09**
PSPTO_2405	xenobiotic reductase A	**2.20**	/	/
PSPTO_2655	oxidoreductase, molybdopterin-binding subunit	/	/	**2.55**
PSPTO_2656	aldehyde oxidase and xanthine dehydrogenase family protein	/	/	**2.68**
PSPTO_2676	glutathione-independent formaldehyde dehydrogenase	**15.86**	/	**20.02**
PSPTO_2680	succinate-semialdehyde dehydrogenase	/	**1.79**	/
PSPTO_2697	oxidoreductase zinc-binding protein	/	**-2.08**	**1.98**
PSPTO_2730	sulfite reductase	/	/	**1.94**
PSPTO_2791	oxidoreductase, aldo/keto reductase family	/	**-1.88**	/
PSPTO_2805	oxidoreductase, FAD-binding	**-3.84**	/	**-3.04**
PSPTO_2932	oxidoreductase, aldo/keto reductase family	/	/	**-2.88**
PSPTO_2957	methanol dehydrogenase, NAD-dependent	/	**2.21**	/
PSPTO_3080	NADH:flavin oxidoreductase / NADH oxidase family	/	/	**2.66**
PSPTO_3106	lactoylglutathione lyase	/	**2.30**	/
PSPTO_3155	indolepyruvate ferredoxin oxidoreductase	/	**-2.75**	**2.69**
PSPTO_3198	luciferase family protein	**2.64**	/	/
PSPTO_3205	isocitrate/isopropylmalate family dehydrogenase	/	/	**2.42**
PSPTO_3262	nitrite reductase	/	/	**3.15**
PSPTO_3356	isocitrate dehydrogenase	**-3.47**	/	**-2.38**
PSPTO_3365	NADH:ubiquinone oxidoreductase subunit A	**-1.96**	/	**-2.25**
PSPTO_3366	NADH dehydrogenase subunit B	/	/	**-2.24**
PSPTO_3368	NADH dehydrogenase subunit E	**-2.17**	/	/
PSPTO_3369	NADH dehydrogenase I subunit F	**-2.04**	/	/
PSPTO_3371	NADH:ubiquinone oxidoreductase subunit H	/	**2.97**	/
PSPTO_3372	NADH dehydrogenase subunit I	/	**3.14**	**-2.92**
PSPTO_3373	NADH:ubiquinone oxidoreductase subunit J	/	**3.06**	**-3.57**
PSPTO_3374	NADH:ubiquinone oxidoreductase subunit K	/	**2.91**	**-3.89**
PSPTO_3375	NADH:ubiquinone oxidoreductase subunit L	/	**2.75**	**-3.55**
PSPTO_3376	NADH:ubiquinone oxidoreductase subunit M	/	**2.65**	**-2.97**
PSPTO_3377	NADH:ubiquinone oxidoreductase subunit N	/	**2.29**	**-2.49**
PSPTO_3471	putative monovalent cation/H+ antiporter subunit A	**1.82**	/	**1.77**
PSPTO_3601	hypothetical protein PSPTO_3601	**3.17**	/	**4.87**
PSPTO_3752	aconitate hydratase 2	**-2.04**	/	**-1.87**
PSPTO_3860	pyruvate dehydrogenase, E1 component	**30.75**	**3.37**	**9.12**
PSPTO_3862	hypothetical protein PSPTO_3862	**12.43**	/	**11.33**
PSPTO_3920	D-isomer specific 2-hydroxyacid dehydrogenase family protein	/	/	**-1.93**
PSPTO_4024	ferredoxin--NADP reductase	/	/	**2.46**
PSPTO_4059	ferredoxin	/	/	**-2.40**
PSPTO_4162	oxidoreductase, molybdopterin-binding protein	**3.04**	/	**2.44**
PSPTO_4168	glycerol kinase	/	**-1.92**	/
PSPTO_4170	glycerol-3-phosphate dehydrogenase	/	/	**3.72**
PSPTO_4285	alcohol dehydrogenase II	/	**-3.17**	**2.83**
PSPTO_4339	fumarate hydratase, class I	**-2.37**	/	**-3.72**

PSPTO_4358	oxidoreductase FAD/FMN-binding protein	/	**2.86**	**-3.11**
PSPTO_4367	lipoprotein	/	**-2.60**	/
PSPTO_4652	iron-sulfur cluster-binding protein	**3.53**	/	/
PSPTO_4928	hypothetical protein PSPTO_4928	/	/	**-1.94**
PSPTO_5006	pyruvate dehydrogenase complex, E2 component, dihydrolipoamide acetyltransferase	/	/	**-2.37**
PSPTO_5072	cytochrome b561	**-4.20**	/	**-2.92**
PSPTO_5134	malic enzyme family protein	/	**2.37**	**-2.21**
PSPTO_5406	formate dehydrogenase accessory protein FdhD	/	**-2.42**	**1.89**
PSPTO_5407	oxidoreductase, molybdopterin-binding protein	/	**-2.35**	/
PSPTO_5453	oxidoreductase, aldo/keto reductase family	**2.21**	/	**2.10**
PSPTO_5460	CAIB/BAIF family protein	/	**-3.29**	**3.96**
PSPTO_5465	acetyl-CoA hydrolase/transferase family protein	**-3.84**	**-2.30**	/
PSPTO_5510	pyruvate carboxylase subunit B	/	**12.25**	**-8.44**
PSPTO_5598	F0F1 ATP synthase subunit epsilon	/	**3.15**	**-4.15**
PSPTO_5599	F0F1 ATP synthase subunit beta	/	**3.61**	**-3.99**
PSPTO_5600	F0F1 ATP synthase subunit gamma	/	**3.69**	**-3.79**
PSPTO_5601	F0F1 ATP synthase subunit alpha	/	**2.95**	**-2.91**
PSPTO_5602	F0F1 ATP synthase subunit delta	/	**2.53**	**-2.66**
PSPTO_5603	F0F1 ATP synthase subunit B	/	**2.53**	**-2.65**
PSPTO_5605	F0F1 ATP synthase subunit A	/	/	**-1.95**
**Carbohydrate transport and metabolism**				
PSPTO_0202	membrane protein	**2.98**	/	/
PSPTO_0364	sugar ABC transporter periplasmic sugar-binding protein	/	/	**-2.84**
PSPTO_0386	D-erythrose 4-phosphate dehydrogenase	/	/	**-1.83**
PSPTO_0390	fructose-bisphosphate aldolase	/	/	**-1.71**
PSPTO_0400	chitinase	**2.72**	/	/
PSPTO_0563	polyamine ABC transporter periplasmic polyamine-binding protein	**-2.78**	/	**-4.58**
PSPTO_0732	hypothetical protein PSPTO_0732	**5.13**	**2.79**	**1.84**
PSPTO_0999	major facilitator family transporter	**-1.85**	/	**-2.39**
PSPTO_1029	endoglucanase	**-2.34**	/	**-4.47**
PSPTO_1052	senescence marker protein-30 family protein	/	/	**2.61**
PSPTO_1075	O-antigen ABC transporter, ATP-binding protein	/	/	**-2.04**
PSPTO_1236	alginate lyase	**2.83**	**2.30**	/
PSPTO_1287	glyceraldehyde 3-phosphate dehydrogenase, type I	**-4.34**	**-3.29**	/
PSPTO_1288	6-phosphogluconate dehydratase	**-3.98**	**-2.44**	/
PSPTO_1289	glucokinase	**-2.56**	/	/
PSPTO_1292	glucose ABC transporter, periplasmic glucose-binding protein	**-3.49**	/	/
PSPTO_1293	glucose ABC transporter permease	**-4.48**	/	**-2.87**
PSPTO_1294	glucose ABC transporter permease	**-3.96**	/	**-2.72**
PSPTO_1295	glucose ABC transporter ATP-binding protein	**-2.70**	/	/
PSPTO_1302	keto-hydroxyglutarate-aldolase/keto-deoxy-phosphogluconate aldolase	**-2.37**	/	**-2.97**
PSPTO_1345	gluconolactonase	**2.11**	/	/
PSPTO_1363	sugar transporter family protein	/	**3.21**	/
PSPTO_1419	inositol-1-monophosphatase	/	**1.95**	**-3.06**
PSPTO_1554	enolase	/	**1.93**	**-2.26**
PSPTO_1608	hypothetical protein PSPTO_1608	**1.94**	/	/

PSPTO_1670	xylulose-5-phosphate/fructose-6-phosphate phosphoketolase	/	**-2.46**	**5.03**
PSPTO_1707	L-sorbosone dehydrogenase	**3.49**	/	**2.99**
PSPTO_1716	alpha-ribazole-5\'-phosphate phosphatase	**-2.41**	/	**-4.25**
PSPTO_1922	major facilitator family transporter	/	**2.43**	**-2.21**
PSPTO_2044	transporter	/	/	**-2.24**
PSPTO_2102	glyceraldehyde 3-phosphate dehydrogenase, type I	/	**2.17**	**-1.92**
PSPTO_2103	membrane protein	/	/	**2.43**
PSPTO_2177	2-dehydro-3-deoxygalactonokinase	/	**-3.99**	**6.04**
PSPTO_2178	2-dehydro-3-deoxy-6-phosphogalactonate aldolase	/	**-4.26**	**4.97**
PSPTO_2179	galactonate dehydratase	/	**-5.64**	**4.26**
PSPTO_2180	MFS transporter, phthalate permease family	/	**-4.36**	**2.82**
PSPTO_2287	methylisocitrate lyase	**4.72**	/	**3.86**
PSPTO_2340	4-hydroxybenzoate transporter	**-2.80**	/	**-2.08**
PSPTO_2367	ribose ABC transporter periplasmic ribose-binding protein	**-1.98**	/	**-2.28**
PSPTO_2368	ribose ABC transporter ATP-binding protein	**-2.45**	/	**-3.49**
PSPTO_2369	ribose ABC transporter permease	**-2.70**	/	**-3.14**
PSPTO_2371	ribokinase	**-2.10**	/	/
PSPTO_2473	periplasmic substrate-binding protein	**-4.17**	**-2.84**	/
PSPTO_2487	aldolase II superfamily protein	/	**-5.02**	**2.61**
PSPTO_2500	major facilitator family transporter	**2.14**	/	/
PSPTO_2688	major facilitator family transporter	**-1.93**	**1.95**	**-3.76**
PSPTO_2702	xylulokinase	**-2.36**	**-2.18**	/
PSPTO_2703	D-mannonate oxidoreductase	**-3.76**	**-3.84**	/
PSPTO_2707	mannitol ABC transporter, periplasmic mannitol-binding protein	**-2.83**	**-2.53**	/
PSPTO_2760	alpha-amylase family protein	**3.07**	/	**3.09**
PSPTO_2761	alpha-amylase family protein	/	/	**2.38**
PSPTO_2785	putrescine ABC transporter periplasmic putrescine-binding protein	/	**-5.60**	**5.58**
PSPTO_2996	ribose ABC transporter, periplasmic ribose-binding protein	/	**-2.27**	/
PSPTO_3052	major facilitator family transporter	/	**-3.81**	/
PSPTO_3122	6-phosphogluconate dehydrogenase	**2.31**	/	/
PSPTO_3125	glycogen synthase	**6.10**	/	**3.69**
PSPTO_3126	alpha-amylase family protein	**4.19**	**-2.02**	**8.46**
PSPTO_3127	4-alpha-glucanotransferase	**4.39**	/	**6.79**
PSPTO_3128	glycosyl hydrolase family protein	**3.46**	/	**4.08**
PSPTO_3130	glycogen operon protein GlgX	**2.63**	/	**2.27**
PSPTO_3278	hypothetical protein PSPTO_3278	/	**-1.90**	/
PSPTO_3318	beta-glucosidase	/	/	**-1.90**
PSPTO_3470	membrane protein	/	/	**2.25**
PSPTO_3560	GDA1/CD39 family protein	**2.42**	/	**2.10**
PSPTO_3589	transporter	**2.25**	/	/
PSPTO_3666	polysaccharide deacetylase family protein	/	**-2.51**	**3.81**
PSPTO_3687	L-sorbosone dehydrogenase	**3.98**	/	**4.96**
PSPTO_3694	glycosyl transferase family protein	**-2.04**	/	**-2.21**
PSPTO_3740	ABC transporter ATP-binding protein	/	**-3.51**	**3.67**
PSPTO_4083	membrane protein	/	/	**-2.37**
PSPTO_4283	pectin lyase	/	**-2.39**	**2.51**
PSPTO_4290	beta-glucosidase	**-2.01**	/	**-2.19**

PSPTO_4337	pyruvate kinase	**-2.48**	/	**-2.63**
PSPTO_4383	AmpG protein	**-2.74**	/	**-3.18**
PSPTO_4494	triosephosphate isomerase	/	/	**-1.77**
PSPTO_4522	membrane protein	**2.40**	/	**2.31**
PSPTO_5062	putative aldolase	/	/	**2.97**
PSPTO_5289	ribose 5-phosphate isomerase	/	**2.05**	**-3.04**
PSPTO_5340	MFS permease-like protein	**-2.90**	/	**-2.19**
**Amino acid transport and metabolism**				
PSPTO_0101	D-amino acid dehydrogenase small subunit	/	/	**2.20**
PSPTO_0144	oligopeptidase A	/	**-3.63**	**5.62**
PSPTO_0203	cysteine synthase	**5.45**	/	**3.37**
PSPTO_0251	peptide ABC transporter permease	/	**-5.16**	**8.28**
PSPTO_0259	4-aminobutyrate aminotransferase	/	/	**3.90**
PSPTO_0272	cysteine desulfurase	/	**-11.73**	**12.55**
PSPTO_0280	methionine aminopeptidase	**2.09**	/	/
PSPTO_0359	glutamine synthetase, type I	**3.56**	/	**2.11**
PSPTO_0462	glycine/betaine/L-proline ABC transporter, ATP-binding subunit	/	**1.90**	/
PSPTO_0463	glycine/betaine/L-proline ABC transporter, permease protein	/	**2.12**	/
PSPTO_0464	glycine betaine/L-proline ABC transporter, periplasmic substrate-binding protein	/	**2.73**	**-2.25**
PSPTO_0481	ACT domain-containing protein	/	/	**2.38**
PSPTO_0518	tryptophan 2-monooxygenase	**10.63**	/	**10.56**
PSPTO_0562	polyamine ABC transporter ATP-binding protein	**-4.68**	**2.41**	**-11.28**
PSPTO_0598	S-adenosylmethionine decarboxylase	**-2.47**	/	**-3.60**
PSPTO_0727	ethanolamine ammonia-lyase, heavy subunit	/	**-2.65**	**2.43**
PSPTO_0774	beta-alanine--pyruvate aminotransferase	/	/	**2.21**
PSPTO_0981	acetolactate synthase large subunit	/	/	**-2.02**
PSPTO_0982	acetolactate synthase small subunit	/	/	**-2.53**
PSPTO_0983	ketol-acid reductoisomerase	/	**1.96**	**-2.95**
PSPTO_1133	amino acid ABC transporter permease	/	**2.29**	**-2.24**
PSPTO_1134	amino acid ABC transporter substrate-binding protein	/	**1.97**	/
PSPTO_1221	LysE family transporter	**15.41**	/	**14.90**
PSPTO_1258	amino acid ABC transporter ATP-binding protein	/	**2.89**	/
PSPTO_1275	glycine cleavage system T protein	/	**-4.38**	**5.32**
PSPTO_1276	glycine dehydrogenase	/	**-6.29**	**5.55**
PSPTO_1277	glycine cleavage system H protein	/	**-4.35**	/
PSPTO_1335	cyclohexadienyl dehydratase	/	**-1.83**	**1.92**
PSPTO_1343	hypothetical protein PSPTO_1343	**3.87**	/	**3.62**
PSPTO_1421	serine O-acetyltransferase	/	/	**-2.61**
PSPTO_1423	cysteine desulfurase	/	**2.24**	**-2.32**
PSPTO_1444	2-isopropylmalate synthase	/	/	**2.12**
PSPTO_1480	homoserine dehydrogenase	/	/	**-1.91**
PSPTO_1481	threonine synthase	/	**2.04**	**-2.22**
PSPTO_1527	cysteine sulfinate desulfinase	/	/	**-2.03**
PSPTO_1528	tetrahydrodipicolinate succinylase	/	/	**-2.32**
PSPTO_1531	class I and II aminotransferase	/	**2.38**	/
PSPTO_1746	phosphoserine aminotransferase	/	/	**-1.87**
PSPTO_1747	chorismate mutase/prephenate dehydratase	/	**2.12**	**-2.19**

PSPTO_1748	prephenate dehydrogenase/3-phosphoshikimate 1-carboxyvinyltransferase family protein	/	**2.16**	**-2.60**
PSPTO_1760	threonine/serine transporter	/	/	**-2.40**
PSPTO_1817	aromatic amino acid permease	**-1.80**	/	**-2.64**
PSPTO_1822	phenylalanine-4-hydroxylase	/	**-3.81**	**2.75**
PSPTO_1826	arginine/ornithine ABC transporter, periplasmic arginine/ornithine-binding protein	/	/	**-2.15**
PSPTO_1827	arginine/ornithine ABC transporter, permease protein	/	/	**-2.18**
PSPTO_1828	arginine/ornithine ABC transporter, permease protein	/	/	**-2.94**
PSPTO_1830	histidine ABC transporter ATP-binding protein	/	**2.30**	**-3.53**
PSPTO_1836	succinylarginine dihydrolase	/	/	**-2.40**
PSPTO_1838	succinylglutamate desuccinylase	/	/	**-2.21**
PSPTO_1885	efflux protein, LysE family	/	**-2.09**	/
PSPTO_2026	ethanolamine permease family protein	**2.29**	/	**3.04**
PSPTO_2045	methylthioribulose-1-phosphate dehydratase	/	/	**-2.11**
PSPTO_2055	spermidine synthase	/	**2.09**	**-2.61**
PSPTO_2061	transglutaminase-like domain protein	**2.64**	/	**1.87**
PSPTO_2120	peptidase, M20/M25/M40 family	**3.80**	/	**4.52**
PSPTO_2206	branched-chain amino acid transport system II carrier protein	/	/	**-2.28**
PSPTO_2261	racemase	**3.16**	/	**1.89**
PSPTO_2451	sarcosine oxidase subunit delta	/	**-6.90**	**4.96**
PSPTO_2775	amino acid ABC transporter substrate-binding protein	/	**-1.94**	**2.77**
PSPTO_2776	amino acid ABC transporter permease	/	/	**4.95**
PSPTO_2777	amino acid ABC transporter permease	/	/	**2.54**
PSPTO_2781	hypothetical protein PSPTO_2781	/	**2.03**	/
PSPTO_2812	peptide ABC transporter ATP-binding protein	/	/	**3.48**
PSPTO_2814	peptide ABC transporter periplasmic peptide-binding protein	/	/	**-2.61**
PSPTO_2915	glutamine ABC transporter, permease protein	/	/	**-4.37**
PSPTO_2934	urea amidolyase-related protein	/	**-2.19**	**2.67**
PSPTO_2935	hypothetical protein PSPTO_2935	/	**-2.44**	**3.02**
PSPTO_3060	glycine betaine/L-proline ABC transporter ATP-binding protein	/	/	**3.09**
PSPTO_3176	thermolysin metallopeptidase	**5.00**	/	**6.50**
PSPTO_3452	LysE family transporter	/	/	**1.99**
PSPTO_3470	membrane protein	/	/	**2.25**
PSPTO_3810	O-succinylhomoserine sulfhydrylase	/	/	**-1.79**
PSPTO_3867	dihydrodipicolinate synthetase family protein	**11.33**	/	**7.05**
PSPTO_3882	polyamine ABC transporter, ATP-binding protein	/	**2.12**	/
PSPTO_3883	HAD-superfamily hydrolase	/	**2.18**	/
PSPTO_4083	membrane protein	/	/	**-2.37**
PSPTO_4112	high-affinity amino acid ABC transporter, ATP-binding protein	/	**2.14**	/
PSPTO_4136	amino acid ABC transporter substrate-binding protein	**2.38**	/	**2.05**
PSPTO_4140	amino acid ABC transporter ATP-binding protein	/	**2.53**	/
PSPTO_4155	argininosuccinate synthase	/	/	**-2.20**
PSPTO_4172	amino acid ABC transporter permease	**-4.32**	/	**-2.87**
PSPTO_4173	amino acid ABC transporter permease	**-5.47**	/	**-4.36**
PSPTO_4174	amino acid ABC transporter ATP-binding protein	**-5.05**	/	**-3.86**
PSPTO_4362	LysE family transporter	**4.17**	**-3.23**	**13.47**

PSPTO_4439	ATP phosphoribosyltransferase	/	/	**-2.24**
PSPTO_4534	peptide ABC transporter substrate-binding protein	**3.45**	**3.46**	/
PSPTO_4535	peptide ABC transporter ATP-binding protein	**7.26**	**3.27**	/
PSPTO_4538	peptide ABC transporter permease	**9.95**	**3.87**	**2.57**
PSPTO_4540	proline iminopeptidase	**42.04**	**3.85**	**10.93**
PSPTO_4558	dipeptide ABC transporter, periplasmic dipeptide-binding protein	/	**-2.26**	/
PSPTO_4561	dipeptide ABC transporter substrate-binding protein	/	**2.09**	**-2.27**
PSPTO_4632	serine hydroxymethyltransferase	/	/	**-2.13**
PSPTO_4885	branched-chain amino acid ABC transporter substrate-binding protein	/	**-2.98**	**5.31**
PSPTO_4886	branched-chain amino acid ABC transporter permease	/	/	**3.14**
PSPTO_4889	branched-chain amino acid ABC transporter ATP-binding protein	/	**2.27**	/
PSPTO_4918	high-affinity branched-chain amino acid ABC transporter, permease protein BraD	/	/	**2.66**
PSPTO_4919	high affinity branched-chain amino acid ABC transporter periplasmic amino acid-binding protein	/	**-2.20**	**2.66**
PSPTO_4924	transglutaminase-like domain protein	**-3.07**	/	**-2.88**
PSPTO_4938	ATP phosphoribosyltransferase regulatory subunit	/	/	**-2.09**
PSPTO_5099	histidine ammonia-lyase	**-3.14**	/	**-5.19**
PSPTO_5121	glutamate synthase, small subunit	/	**2.16**	**-3.31**
PSPTO_5123	glutamate synthase, large subunit	/	/	**-3.07**
PSPTO_5126	3-dehydroquinate synthase	/	/	**-2.31**
PSPTO_5127	shikimate kinase	/	**1.95**	**-2.83**
PSPTO_5178	serine O-acetyltransferase	/	/	**2.32**
PSPTO_5245	amino acid ABC transporter substrate-binding protein	/	/	**4.47**
PSPTO_5246	amino acid ABC transporter permease	**3.63**	/	**6.27**
PSPTO_5247	amino acid ABC transporter permease	/	/	**5.10**
PSPTO_5248	amino acid ABC transporter ATP-binding protein	/	/	**2.53**
PSPTO_5276	proline-specific permease proY	/	/	**-2.43**
PSPTO_5294	D-3-phosphoglycerate dehydrogenase	/	/	**-2.10**
PSPTO_5310	glutamine synthetase	/	**-1.75**	/
PSPTO_5339	aromatic-amino-acid aminotransferase	/	/	**2.13**
PSPTO_5357	amino acid ABC transporter ATP-binding protein	/	/	**-1.82**
PSPTO_5359	amino acid ABC transporter permease	/	/	**-2.75**
PSPTO_5360	amino acid ABC transporter permease	/	/	**-2.68**
PSPTO_5393	hypothetical protein PSPTO_5393	/	**1.98**	**-1.81**
PSPTO_5394	carbon-nitrogen hydrolase family protein	/	**2.12**	/
PSPTO_5500	sodium/alanine transporter	**-2.60**	/	**-2.32**
**Nucleotide transport and metabolism**				
PSPTO_0043	cytidine/deoxycytidylate deaminase family protein	/	/	**2.65**
PSPTO_0073	guanosine-3\',5\'-bis(diphosphate) 3\'-pyrophosphohydrolase	**-2.09**	/	**-2.12**
PSPTO_0230	adenylate cyclase	**-4.40**	**-2.10**	**-2.09**
PSPTO_0549	bis(5\'-nucleosyl)-tetraphosphatase, symmetrical	**-2.33**	/	**-3.72**
PSPTO_0772	xanthine/uracil permease family protein	/	**-2.88**	**2.77**
PSPTO_1129	uracil transporter	/	/	**-2.13**
PSPTO_1131	hypoxanthine phosphoribosyltransferase	/	**1.90**	**-2.78**
PSPTO_1153	NAD(P)H-flavin oxidoreductase	**5.46**	/	**3.78**
PSPTO_1157	luciferase family protein	**4.23**	/	**4.69**

PSPTO_1427	chaperone protein HscA	/	**2.41**	**-2.92**
PSPTO_1449	inosine 5\'-monophosphate dehydrogenase	/	/	**-1.72**
PSPTO_1450	GMP synthase	/	/	**-2.22**
PSPTO_1457	cytidine/deoxycytidylate deaminase family protein	/	**-2.41**	/
PSPTO_1459	phosphoribosylformylglycinamidine synthase	/	/	**-2.20**
PSPTO_1468	phosphoribosylglycinamide formyltransferase 2	/	/	**-1.81**
PSPTO_1509	adenylate kinase	**-2.95**	/	/
PSPTO_1552	CTP synthase	/	/	**-2.02**
PSPTO_1699	phosphoribosylglycinamide formyltransferase	/	/	**-2.17**
PSPTO_1700	phosphoribosylformylglycinamidine cyclo-ligase	/	/	**-1.78**
PSPTO_1749	cytidylate kinase	**-1.96**	/	**-2.81**
PSPTO_1871	cytosine/purines uracil thiamine allantoin permease	/	**-3.33**	**6.06**
PSPTO_2331	deoxyguanosinetriphosphate triphosphohydrolase-like protein	/	/	**1.81**
PSPTO_2341	3-carboxy-cis,cis-muconate cycloisomerase	**-3.42**	/	**-3.17**
PSPTO_2474	DNA/RNA non-specific endonuclease	**2.16**	/	/
PSPTO_3360	adenylosuccinate lyase	/	**2.06**	**-2.97**
PSPTO_3660	xanthine dehydrogenase, N-terminal subunit, partial	**2.71**	/	**3.44**
PSPTO_3661	xanthine dehydrogenase, C-terminal subunit, partial	**2.64**	/	**5.11**
PSPTO_3663	guanine aminohydrolase	/	**-2.35**	**4.36**
PSPTO_3668	allantoicase	/	/	**2.33**
PSPTO_3750	hypothetical protein PSPTO_3750	**-1.83**	/	**-2.10**
PSPTO_3811	amidophosphoribosyltransferase	/	/	**-2.00**
PSPTO_3950	phosphoribosylaminoimidazolesuccinocarboxamide synthase	/	**1.91**	**-2.48**
PSPTO_4157	dihydroorotase, homodimeric type	/	/	**-2.08**
PSPTO_4314	formyltetrahydrofolate deformylase	**-1.78**	/	**-2.46**
PSPTO_4501	carbamoyl-phosphate synthase large subunit	/	**1.98**	**-2.78**
PSPTO_4502	carbamoyl-phosphate synthase small subunit	/	/	**-2.03**
PSPTO_4650	aldehyde oxidase and xanthine dehydrogenase family protein	**2.87**	/	**3.30**
PSPTO_4651	oxidoreductase, molybdopterin-binding subunit	**3.42**	/	**3.04**
PSPTO_4866	phosphoribosylaminoimidazolecarboxamide formyltransferase/IMP cyclohydrolase	/	**1.95**	**-2.96**
PSPTO_4867	phosphoribosylamine--glycine ligase	/	**2.48**	**-3.94**
PSPTO_4911	urease accessory protein UreG	/	/	**2.45**
PSPTO_4937	adenylosuccinate synthetase	/	**2.08**	**-2.97**
PSPTO_4975	cytosine/purines uracil thiamine allantoin permease	/	/	**-2.61**
PSPTO_5493	phosphoribosylaminoimidazole carboxylase ATPase subunit	/	/	**-2.62**
PSPTO_5494	phosphoribosylaminoimidazole carboxylase catalytic subunit	/	/	**-2.18**
**Coenzyme transport and metabolism**				
PSPTO_0360	thiamin biosynthesis protein ThiI	/	**1.76**	**-2.50**
PSPTO_0383	S-adenosylmethionine synthetase	/	/	**-1.81**
PSPTO_0494	biotin synthetase	**2.59**	/	/
PSPTO_0542	dihydroneopterin aldolase	/	/	**-2.05**
PSPTO_0552	pyridoxal phosphate biosynthetic protein PdxA	/	**1.94**	**-3.12**
PSPTO_0610	birA bifunctional protein	/	/	**-2.49**
PSPTO_0695	thiamine monophosphate kinase	/	**2.02**	**-3.61**
PSPTO_0696	GTP cyclohydrolase II	**-1.82**	/	**-2.45**
PSPTO_0960	pantoate--beta-alanine ligase	/	/	**-1.80**

PSPTO_0962	2-amino-4-hydroxy-6-hydroxymethyldihydropteridine pyrophosphokinase	/	/	**-2.19**
PSPTO_1128	ferrochelatase	/	/	**2.86**
PSPTO_1710	nitroreductase family protein	/	/	**-2.41**
PSPTO_1711	cobalamin biosynthesis protein CobD	/	/	**-2.40**
PSPTO_1712	cobalamin biosynthesis protein CobC	/	**2.33**	**-3.15**
PSPTO_1713	cobyric acid synthase	/	**3.15**	**-4.13**
PSPTO_1714	cobinamide kinase/cobinamide phosphate guanylyltransferase	/	**2.77**	**-3.96**
PSPTO_1715	nicotinate-nucleotide--dimethylbenzimidazole phosphoribosyltransferase	/	**2.38**	**-4.81**
PSPTO_1717	cobalamin (5\'-phosphate) synthase	**-2.66**	/	**-3.37**
PSPTO_1738	hypothetical protein PSPTO_1738	**4.28**	/	**5.68**
PSPTO_1821	pterin-4-alpha-carbinolamine dehydratase	/	**-2.66**	/
PSPTO_1871	cytosine/purines uracil thiamine allantoin permease	/	**-3.33**	**6.06**
PSPTO_1993	oxygen-independent coproporphyrinogen III oxidase	/	**-3.44**	**5.97**
PSPTO_2035	GTP cyclohydrolase I	/	/	**-1.85**
PSPTO_2346	4-hydroxyphenylpyruvate dioxygenase	/	/	**2.05**
PSPTO_2351	molybdopterin-guanine dinucleotide biosynthesis protein MobA	/	/	**-2.00**
PSPTO_3147	hypothetical protein PSPTO_3147	/	**2.11**	**-3.10**
PSPTO_3148	magnesium chelatase, subunit ChII	/	**2.29**	**-3.24**
PSPTO_3204	hypothetical protein PSPTO_3204	/	/	**3.06**
PSPTO_3344	siroheme synthase	/	**2.08**	**-2.65**
PSPTO_3451	FMN reductase, NADH-dependent	/	**-5.09**	**10.06**
PSPTO_3733	methylenetetrahydrofolate dehydrogenase/methenyltetrahydrofolate cyclohydrolase	/	/	**-1.77**
PSPTO_3814	bifunctional folylpolyglutamate synthase/dihydrofolate synthase	/	/	**-2.23**
PSPTO_3959	quinolinate synthetase	/	/	**-1.85**
PSPTO_3969	radical SAM domain-containing protein	/	/	**-2.22**
PSPTO_4034	cinA domain protein	**2.27**	/	/
PSPTO_4116	pyridoxamine 5\'-phosphate oxidase	/	**2.48**	**-1.86**
PSPTO_4214	pyridoxal phosphate biosynthetic protein PdxJ	**-1.80**	/	**-2.79**
PSPTO_4819	lipoate-protein ligase B	/	/	**-1.81**
PSPTO_4976	thiamin biosynthesis protein ThiC	/	**2.03**	**-2.08**
PSPTO_5052	HemN family oxidoreductase	/	/	**-2.38**
PSPTO_5086	2-(5\'\'-triphosphoribosyl)-3\'-dephosphocoenzyme-A synthase	/	**-5.51**	/
PSPTO_5118	uroporphyrinogen decarboxylase	/	**-2.42**	**2.65**
**Lipid transport and metabolism**				
PSPTO_0187	hdtS protein	/	**2.36**	**-2.92**
PSPTO_0200	hypothetical protein PSPTO_0200	**2.04**	/	/
PSPTO_0452	putative acyltransferase	**-2.07**	/	**-2.36**
PSPTO_0500	acyl-CoA dehydrogenase family protein	**2.65**	/	**1.99**
PSPTO_0506	acyl-CoA dehydrogenase family protein	/	/	**2.29**
PSPTO_0734	MaoC-like domain protein	**9.36**	/	**9.90**
PSPTO_0742	MaoC-like domain protein	**2.77**	/	**3.32**
PSPTO_0743	3-ketoacyl-(acyl-carrier-protein) reductase	**2.15**	/	/
PSPTO_0744	acetyl-CoA acetyltransferase	**2.92**	/	**2.04**
PSPTO_0783	3-hydroxyisobutyrate dehydrogenase	/	/	**2.64**
PSPTO_0809	hydroxymethylbutenyl pyrophosphate reductase	/	**2.16**	**-3.15**

PSPTO_0957	acetyl-CoA acetyltransferase	/	**-1.92**	/
PSPTO_1056	short chain dehydrogenase/reductase family oxidoreductase	**3.85**	/	**3.77**
PSPTO_1119	short chain dehydrogenase/reductase family oxidoreductase	**3.47**	**-2.99**	**10.38**
PSPTO_1538	undecaprenyl diphosphate synthase	/	**1.90**	**-2.86**
PSPTO_1539	phosphatidate cytidylyltransferase	/	**2.01**	**-3.49**
PSPTO_1540	1-deoxy-D-xylulose 5-phosphate reductoisomerase	**-1.76**	/	**-3.05**
PSPTO_1545	(3R)-hydroxymyristoyl-ACP dehydratase	/	**2.18**	**-3.64**
PSPTO_1550	acetyl-CoA carboxylase carboxyltransferase subunit alpha	/	/	**-1.78**
PSPTO_1556	4-diphosphocytidyl-2C-methyl-D-erythritol synthase	/	**1.89**	**-2.61**
PSPTO_1720	outer membrane protein	/	**-2.18**	**2.01**
PSPTO_1766	lipase	**-4.52**	/	**-3.83**
PSPTO_1825	acetyl-CoA synthetase	**-11.36**	**-11.13**	/
PSPTO_1916	3-oxoacyl-(acyl-carrier-protein) synthase III	**2.55**	**-2.31**	**5.88**
PSPTO_2335	exopolysaccharide production protein ExoZ	/	/	**2.26**
PSPTO_2736	biotin carboxylase	/	**-5.09**	**3.12**
PSPTO_2737	gamma-carboxygeranoyl-CoA hydratase	/	**-3.90**	**2.78**
PSPTO_2738	carboxyl transferase domain protein	/	**-4.77**	**3.39**
PSPTO_2739	acyl-CoA dehydrogenase	/	**-4.38**	**3.30**
PSPTO_2742	hydroxymethylglutaryl-CoA lyase	/	**-2.48**	**2.77**
PSPTO_3282	lipoprotein	/	**-2.45**	**2.68**
PSPTO_3455	3-oxoacid CoA-transferase, subunit A family	/	**-2.17**	**2.17**
PSPTO_3456	3-oxoacid CoA-transferase, subunit B family	/	**-2.02**	/
PSPTO_3486	hypothetical protein PSPTO_3486	**4.83**	**3.09**	/
PSPTO_3527	hypothetical protein PSPTO_3527	**2.04**	/	/
PSPTO_3705	enoly-CoA hydratase/isomerase family protein	**2.97**	/	**3.67**
PSPTO_3706	acyl-CoA dehydrogenase	**4.23**	/	**4.80**
PSPTO_3721	enoyl-(acyl-carrier-protein) reductase	/	/	**-2.38**
PSPTO_3815	acetyl-CoA carboxylase subunit beta	/	/	**-2.04**
PSPTO_3831	acyl carrier protein	/	/	**-2.60**
PSPTO_3832	3-ketoacyl-(acyl-carrier-protein) reductase	**-1.94**	/	**-3.42**
PSPTO_3833	malonyl CoA-acyl carrier protein transacylase	**-2.06**	/	**-3.14**
PSPTO_3834	fatty acid/phospholipid synthesis protein PlsX	**-2.38**	/	**-2.96**
PSPTO_3857	acyl-CoA dehydrogenase	**3.93**	/	**3.10**
PSPTO_4094	3-oxoacyl-(acyl carrier protein) synthase III	/	**-4.59**	**3.46**
PSPTO_4098	long-chain-fatty-acid--CoA ligase	/	/	**2.16**
PSPTO_4201	short chain dehydrogenase/reductase family oxidoreductase	/	/	**-2.16**
PSPTO_4307	3-oxoadipyl-CoA thiolase	**-2.89**	/	/
PSPTO_4354	acyltransferase	/	/	**-3.63**
PSPTO_4380	3-ketoacyl-(acyl-carrier-protein) reductase	/	**-2.15**	/
PSPTO_4781	hypothetical protein PSPTO_4781	**2.05**	/	/
PSPTO_4843	esterase/lipase/thioesterase family protein	/	**1.86**	/
PSPTO_4860	acetyl-CoA carboxylase, biotin carboxyl carrier protein	**-1.74**	/	**-2.26**
PSPTO_4861	acetyl-CoA carboxylase, biotin carboxylase	/	/	**-2.34**
PSPTO_5067	4-hydroxybenzoyl-CoA thioesterase	/	/	**2.87**
PSPTO_5084	malonate decarboxylase subunit beta	/	**-10.47**	**10.41**
PSPTO_5085	malonate decarboxylase subunit delta	/	**-7.27**	**17.53**

PSPTO_5092	acyltransferase family protein	**-2.75**	/	**-2.28**
PSPTO_5093	acyl carrier protein	**-4.48**	/	**-4.34**
PSPTO_5094	acyl carrier protein	**-3.39**	/	**-3.48**
PSPTO_5108	hypothetical protein PSPTO_5108	/	/	**-4.09**
PSPTO_5145	poly(3-hydroxyalkanoate) polymerase	**19.85**	**2.12**	**9.36**
PSPTO_5299	hypothetical protein PSPTO_5299	/	/	**2.01**
PSPTO_5381	biotin carboxylase	/	**1.92**	**-2.25**
PSPTO_5461	acyl-CoA dehydrogenase	/	**-4.01**	**6.66**
PSPTO_5489	cytosolic long-chain acyl-CoA thioester hydrolase family protein	/	**-2.58**	**2.14**
PSPTO_5511	pyruvate carboxylase subunit A	/	**7.95**	**-4.64**
PSPTO_5530	cardiolipin synthetase	**2.50**	/	/
PSPTO_5536	CDP-alcohol phosphatidyltransferase	/	/	**2.30**
PSPTO_5537	hypothetical protein PSPTO_5537	/	/	**2.42**
**Inorganic ion transport and metabolism**				
PSPTO_0110	metallo-beta-lactamase superfamily protein	/	**-2.31**	**2.29**
PSPTO_0118	Na/Pi cotransporter family protein	**-3.17**	/	/
PSPTO_0148	oligopeptide transporter	**2.61**	/	**3.37**
PSPTO_0180	Trk system potassium uptake protein TrkA	/	/	**-2.04**
PSPTO_0250	peptide ABC transporter permease	/	**-4.41**	**6.70**
PSPTO_0251	peptide ABC transporter permease	/	**-5.16**	**8.28**
PSPTO_0314	iron ABC transporter periplasmic iron-binding protein	/	**-2.13**	**3.04**
PSPTO_0366	Na+/H+ antiporter NhaP	/	**4.05**	**-3.74**
PSPTO_0445	regulatory protein	/	/	**2.98**
PSPTO_0550	apaG protein	/	/	**-2.69**
PSPTO_0564	polyamine ABC transporter permease	**-2.27**	/	**-3.66**
PSPTO_0750	copper-translocating P-type ATPase	**-2.16**	/	**-2.16**
PSPTO_0752	copZ protein	**-2.92**	/	**-2.98**
PSPTO_0753	Bcr/CflA family multidrug resistance transporter	**-2.59**	/	**-3.07**
PSPTO_0763	iron(III) dicitrate transport system, periplasmic iron-binding protein FecB	/	**-3.42**	/
PSPTO_0820	AcrB/AcrD/AcrF family protein	**2.72**	/	/
PSPTO_0998	alkylphosphonate utilization operon protein PhnA	/	/	**-2.61**
PSPTO_1260	cyanate MFS transporter	/	**2.52**	**-2.76**
PSPTO_1338	superoxide dismutase, Cu-Zn	**2.45**	/	**2.29**
PSPTO_1340	carbonic anhydrase	**-2.13**	/	/
PSPTO_1529	arsC family protein	/	/	**-2.57**
PSPTO_2304	nitrate transporter	/	**-2.80**	**3.64**
PSPTO_2484	TonB-dependent siderophore receptor	**2.48**	/	**2.59**
PSPTO_2592	aliphatic isothiocyanate resistance protein SaxG; AcrB/AcrD/AcrF family	**4.70**	/	**3.42**
PSPTO_2705	mannitol ABC transporter permease	**-3.94**	**-2.47**	/
PSPTO_2706	mannitol ABC transporter permease	**-4.73**	**-3.02**	/
PSPTO_2746	OmpA family protein	**4.35**	**6.02**	/
PSPTO_2809	hypothetical protein PSPTO_2809	/	**-2.80**	**3.78**
PSPTO_2812	peptide ABC transporter ATP-binding protein	/	/	**3.48**
PSPTO_2846	TonB-dependent siderophore receptor	/	**1.97**	**-3.07**
PSPTO_3062	iron-sulfur cluster-binding protein, rieske family	/	**-2.49**	**2.26**
PSPTO_3234	sulfate permease family protein	**-2.68**	/	**-3.02**
PSPTO_3249	dipeptide ABC transporter, permease protein DppB	/	**4.54**	/

PSPTO_3256	iron ABC transporter, periplasmic iron-binding protein	**-2.05**	/	/
PSPTO_3266	phosphate transporter ATP-binding protein	**2.48**	**12.08**	**-4.88**
PSPTO_3267	phosphate ABC transporter permease	**3.52**	**11.64**	**-3.31**
PSPTO_3268	phosphate ABC transporter permease	**4.49**	**12.32**	**-2.74**
PSPTO_3269	phosphate ABC transporter substrate-binding protein	**4.50**	**11.17**	**-2.48**
PSPTO_3474	putative monovalent cation/H+ antiporter subunit E	/	**2.26**	/
PSPTO_3475	potassium efflux system protein PhaF	/	**2.28**	/
PSPTO_3565	gluconate permease	**-2.24**	**-2.05**	/
PSPTO_3574	TonB-dependent siderophore receptor	/	**-2.33**	/
PSPTO_3582	catalase	**4.31**	/	**4.38**
PSPTO_3593	sulfate permease family protein	/	/	**-2.21**
PSPTO_3597	hypothetical protein PSPTO_3597	/	**-2.94**	**3.39**
PSPTO_3599	hypothetical protein PSPTO_3599	/	/	**-2.18**
PSPTO_3701	cation transporter	/	**3.30**	**-2.44**
PSPTO_3718	ABC transporter permease	/	**1.86**	**-2.09**
PSPTO_3719	ABC transporter permease	/	**2.05**	**-2.69**
PSPTO_3784	potassium channel protein	**1.93**	/	/
PSPTO_3878	ABC transporter substrate-binding protein	**3.36**	/	**3.03**
PSPTO_4171	amino acid ABC transporter substrate-binding protein	**-3.76**	**-2.06**	/
PSPTO_4193	iron utilization protein	/	/	**2.05**
PSPTO_4238	ABC transporter substrate-binding protein	/	**-2.52**	**2.89**
PSPTO_4300	drug resistance transporter, EmrB/QacA family	**-2.01**	/	/
PSPTO_4312	phosphate transporter family protein	**-4.32**	/	**-5.13**
PSPTO_4366	iron-regulated protein A	/	**-2.44**	/
PSPTO_4432	bifunctional sulfate adenylyltransferase subunit 1/adenylylsulfate kinase protein	/	/	**-1.76**
PSPTO_4477	K+-dependent Na+/Ca+ exchanger-like protein	/	/	**-2.24**
PSPTO_4480	Tat (twin-arginine translocation) pathway signal sequence domain-containing protein	**3.03**	/	/
PSPTO_4533	sodium-proton antiporter NhaA	/	**3.00**	**-2.15**
PSPTO_4535	peptide ABC transporter ATP-binding protein	**7.26**	**3.27**	/
PSPTO_4537	peptide ABC transporter permease	**14.80**	**4.31**	**3.43**
PSPTO_4538	peptide ABC transporter permease	**9.95**	**3.87**	**2.57**
PSPTO_4906	bacterioferritin	**2.64**	/	**2.67**
PSPTO_5191	AcrB/AcrD/AcrF family protein	/	**1.96**	/
PSPTO_5261	D-methionine ABC transporter permease	/	/	**-2.80**
PSPTO_5262	DL-methionine transporter ATP-binding subunit	/	/	**-2.72**
PSPTO_5263	catalase	**8.28**	/	**8.56**
PSPTO_5265	zinc ABC transporter permease	/	/	**-2.34**
PSPTO_5319	taurine ABC transporter, periplasmic taurine-binding protein	/	/	**-2.95**
PSPTO_5483	phosphate transport system protein PhoU	/	**2.42**	**-2.07**
PSPTO_5484	phosphate transporter ATP-binding protein	/	**3.14**	**-2.03**
PSPTO_5485	phosphate ABC transporter permease	**2.20**	**4.51**	**-2.05**
PSPTO_5486	phosphate ABC transporter permease	**4.09**	**5.00**	/
PSPTO_5487	phosphate ABC transporter substrate-binding protein	**9.75**	**7.63**	/
PSPTO_5519	sodium/hydrogen exchanger family protein	**3.23**	/	**3.24**
PSPTO_5542	glucan biosynthesis protein D	**-4.10**	**-3.34**	/
PSPTO_5550	ABC transporter ATP-binding protein	**-3.20**	/	**-2.50**

PSPTO_5551	ABC transporter permease	**-5.31**	/	**-3.57**
PSPTO_5560	TonB-dependent receptor	/	/	**2.99**
**Secondary metabolites biosynthesis, transport and catabolism**				
PSPTO_1056	short chain dehydrogenase/reductase family oxidoreductase	**3.85**	/	**3.77**
PSPTO_1119	short chain dehydrogenase/reductase family oxidoreductase	**3.47**	**-2.99**	**10.38**
PSPTO_1356	isochorismatase family protein	/	**2.14**	/
PSPTO_1456	multicopper oxidase	/	**-2.29**	/
PSPTO_1677	dienelactone hydrolase family protein	**2.16**	/	**2.44**
PSPTO_1868	homospermidine synthase	/	/	**1.99**
PSPTO_2216	4-hydroxybenzoyl-CoA thioesterase	**2.24**	/	**2.12**
PSPTO_2338	protocatechuate 3,4-dioxygenase subunit beta	**-2.48**	**-1.96**	/
PSPTO_2339	protocatechuate 3,4-dioxygenase subunit alpha	**-2.70**	/	/
PSPTO_2342	4-carboxymuconolactone decarboxylase	**-4.57**	/	**-3.84**
PSPTO_2829	non-ribosomal peptide synthetase SyfA	**4.70**	/	**7.98**
PSPTO_3051	2,4\'-dihydroxyacetophenone dioxygenase	**-8.52**	**-8.09**	/
PSPTO_3193	metalloprotease	**-1.85**	/	/
PSPTO_3332	alkaline metalloendoprotease	**29.51**	**2.58**	**11.42**
PSPTO_3389	lysozyme	**-19.85**	/	**-8.79**
PSPTO_3550	fumarylacetoacetase	/	**-2.37**	**1.90**
PSPTO_3551	homogentisate 1,2-dioxygenase	/	**-2.28**	**2.33**
PSPTO_3864	autoinducer synthesis protein PsyI	**12.81**	/	**15.37**
PSPTO_4084	mannuronan C-5-epimerase	**2.08**	**2.28**	/
PSPTO_4201	short chain dehydrogenase/reductase family oxidoreductase	/	/	**-2.16**
PSPTO_4213	tRNA mo(5)U34 methyltransferase	/	/	**-1.93**
PSPTO_4286	hypothetical protein PSPTO_4286	**-2.61**	/	**-2.08**
PSPTO_4519	non-ribosomal peptide synthetase, terminal component	/	**1.82**	/
PSPTO_4662	histone deacetylase family protein	/	/	**2.11**
PSPTO_5083	malonate decarboxylase subunit gamma	/	**-7.20**	**8.28**
PSPTO_5093	acyl carrier protein	**-4.48**	/	**-4.34**
PSPTO_5094	acyl carrier protein	**-3.39**	/	**-3.48**
PSPTO_5096	AMP-binding protein	**-3.66**	/	**-3.98**
**Function unknown**				
PSPTO_0012	hypothetical protein PSPTO_0012	**2.05**	/	**2.40**
PSPTO_0013	hypothetical protein PSPTO_0013	/	/	**2.02**
PSPTO_0015	hypothetical protein PSPTO_0015	/	**-2.09**	/
PSPTO_0020	hypothetical protein PSPTO_0020	/	/	**5.20**
PSPTO_0021	hypothetical protein PSPTO_0021	/	**-2.44**	**4.91**
PSPTO_0025	haloacid dehalogenase-like family hydrolase	/	/	**2.11**
PSPTO_0038	hypothetical protein PSPTO_0038	**2.24**	/	**1.94**
PSPTO_0050	hypothetical protein PSPTO_0050	**3.46**	/	**2.22**
PSPTO_0108	membrane protein	/	**-2.66**	**2.06**
PSPTO_0109	hypothetical protein PSPTO_0109	/	**-2.94**	**2.71**
PSPTO_0117	methyl-accepting chemotaxis protein	**-5.45**	**-2.65**	**-2.05**
PSPTO_0143	hypothetical protein PSPTO_0143	/	**-2.68**	**4.96**
PSPTO_0147	hypothetical protein PSPTO_0147	**2.74**	/	**3.79**
PSPTO_0161	hypothetical protein PSPTO_0161	**2.21**	**1.90**	/
PSPTO_0188		/	**-5.72**	/

PSPTO_0188		/	/	**7.20**
PSPTO_0201	hypothetical protein PSPTO_0201	**2.47**	/	/
PSPTO_0207		/	/	**2.38**
PSPTO_0208	hypothetical protein PSPTO_0208	/	/	**2.73**
PSPTO_0232	hypothetical protein PSPTO_0232	**-2.33**	/	/
PSPTO_0242	hypothetical protein PSPTO_0242	/	**-2.18**	/
PSPTO_0243	hypothetical protein PSPTO_0243	/	/	**3.03**
PSPTO_0247	haloacid dehalogenase	/	/	**2.53**
PSPTO_0249	peptide ABC transporter periplasmic peptide-binding protein	/	**-3.31**	**5.20**
PSPTO_0252	peptide ABC transporter ATP-binding protein	/	/	**4.37**
PSPTO_0260	hypothetical protein PSPTO_0260	/	**-2.99**	**5.89**
PSPTO_0262	hypothetical protein PSPTO_0262	/	/	**4.07**
PSPTO_0263	methyl-accepting chemotaxis protein	/	/	**2.55**
PSPTO_0274	hypothetical protein PSPTO_0274	**4.41**	/	**4.12**
PSPTO_0276	haloacid dehalogenase-like family hydrolase	**2.64**	/	/
PSPTO_0278	hypothetical protein PSPTO_0278	**3.19**	/	**2.63**
PSPTO_0283	hypothetical protein PSPTO_0283	**-1.90**	**2.23**	**-4.24**
PSPTO_0286		/	/	**-1.82**
PSPTO_0295	hypothetical protein PSPTO_0295	**3.44**	/	/
PSPTO_0312	hypothetical protein PSPTO_0312	**2.63**	/	**3.61**
PSPTO_0326	hypothetical protein PSPTO_0326	/	**2.17**	**-2.55**
PSPTO_0330	hypothetical protein PSPTO_0330	**2.20**	**5.51**	**-2.50**
PSPTO_0332	hypothetical protein PSPTO_0332	**3.59**	**3.71**	/
PSPTO_0393	hypothetical protein PSPTO_0393	/	/	**2.32**
PSPTO_0412	hypothetical protein PSPTO_0412	/	/	**-2.63**
PSPTO_0435	tRNA (guanine-N(7)-)-methyltransferase	/	/	**-1.81**
PSPTO_0438	hypothetical protein PSPTO_0438	/	**2.12**	/
PSPTO_0468	hypothetical protein PSPTO_0468	/	**2.60**	/
PSPTO_0469	hypothetical protein PSPTO_0469	**1.95**	**2.54**	/
PSPTO_0472	response regulator	**3.32**	/	**2.38**
PSPTO_0491	Tat (twin-arginine translocation) pathway signal sequence domain-containing protein	**9.39**	**9.19**	/
PSPTO_0493	competence protein ComF	**6.16**	/	**4.76**
PSPTO_0499	hypothetical protein PSPTO_0499	/	**-2.59**	**2.66**
PSPTO_0517	hydrolase	**11.52**	/	**11.14**
PSPTO_0525	lipoprotein	**2.31**	/	**2.61**
PSPTO_0530	hypothetical protein PSPTO_0530	/	/	**3.87**
PSPTO_0533		/	/	**3.53**
PSPTO_0545	SpoVR like family protein	**2.32**	**-2.81**	**6.52**
PSPTO_0546	hypothetical protein PSPTO_0546	**2.82**	**-2.40**	**6.78**
PSPTO_0570	transcriptional regulator PrtN	/	**2.09**	**-1.98**
PSPTO_0573	hypothetical protein PSPTO_0573	/	**2.59**	**-3.50**
PSPTO_0575	hypothetical protein PSPTO_0575	/	**2.96**	**-5.00**
PSPTO_0576	hypothetical protein PSPTO_0576	/	**3.63**	**-4.03**
PSPTO_0577	tail sheath protein	/	**3.43**	**-3.40**
PSPTO_0578	hypothetical protein PSPTO_0578	/	**4.86**	**-4.01**
PSPTO_0579	sigma factor domain protein	/	**4.04**	/
PSPTO_0580	tail tape measure protein	/	**2.80**	**-2.93**

PSPTO_0601	short chain dehydrogenase/reductase family oxidoreductase	/	/	**2.10**
PSPTO_0602	hypothetical protein PSPTO_0602	**2.31**	/	**3.12**
PSPTO_0612	hypothetical protein PSPTO_0612	**-2.01**	/	**-2.34**
PSPTO_0646	preprotein translocase subunit SecY	/	**2.46**	**-4.27**
PSPTO_0647	50S ribosomal protein L36	/	**2.65**	**-4.68**
PSPTO_0651	DNA-directed RNA polymerase subunit alpha	/	**2.40**	**-4.18**
PSPTO_0658	hypothetical protein PSPTO_0658	/	**-1.96**	**2.22**
PSPTO_0666	hypothetical protein PSPTO_0666	**3.08**	/	**3.13**
PSPTO_0675	arylesterase	**4.04**	/	**5.11**
PSPTO_0684	hypothetical protein PSPTO_0684	**9.36**	**2.16**	**4.34**
PSPTO_0693	6,7-dimethyl-8-ribityllumazine synthase	/	/	**-2.56**
PSPTO_0697	hypothetical protein PSPTO_0697	/	/	**-2.43**
PSPTO_0725	GNAT family acetyltransferase	**3.82**	/	**3.43**
PSPTO_0738	lipoprotein	/	/	**2.37**
PSPTO_0751	hypothetical protein PSPTO_0751	/	/	**2.29**
PSPTO_0758	2OG-Fe(II) oxygenase family oxidoreductase	**2.77**	/	**5.42**
PSPTO_0759	bmp family protein	/	**-2.28**	**3.52**
PSPTO_0766	short chain dehydrogenase	/	/	**1.92**
PSPTO_0767	ABC transporter permease	/	/	**4.00**
PSPTO_0768	ABC transporter permease	/	**-2.29**	**3.13**
PSPTO_0769	ABC transporter ATP-binding protein	/	**-3.50**	**9.32**
PSPTO_0798	50S ribosomal protein L27	**-1.78**	**2.01**	**-3.58**
PSPTO_0799	GTP-binding protein, GTP1/Obg family	**-1.77**	**1.87**	**-3.31**
PSPTO_0803	hypothetical protein PSPTO_0803	/	/	**3.24**
PSPTO_0814	hypothetical protein PSPTO_0814	**-2.21**	/	/
PSPTO_0828	hypothetical protein PSPTO_0828	/	/	**-2.01**
PSPTO_0840	hypothetical protein PSPTO_0840	/	**-1.98**	/
PSPTO_0854	hypothetical protein PSPTO_0854	/	**-2.18**	**4.77**
PSPTO_0856	hypothetical protein PSPTO_0856	**2.41**	/	**2.67**
PSPTO_0914	STAS domain-containing protein	/	/	**3.95**
PSPTO_0916	methyl-accepting chemotaxis protein	**2.82**	/	**4.00**
PSPTO_0985	hypothetical protein PSPTO_0985	**2.01**	**-2.06**	**4.15**
PSPTO_0987	hypothetical protein PSPTO_0987	**-2.45**	/	**-3.66**
PSPTO_0988	hypothetical protein PSPTO_0988	/	**2.07**	**-2.67**
PSPTO_0989	hypothetical protein PSPTO_0989	/	/	**-2.06**
PSPTO_0990	hypothetical protein PSPTO_0990	/	/	**-1.89**
PSPTO_0993	hypothetical protein PSPTO_0993	**3.13**	/	**2.42**
PSPTO_1002		/	**-2.28**	/
PSPTO_1003	GNAT family acetyltransferase	**2.22**	/	**2.68**
PSPTO_1008	methyl-accepting chemotaxis protein	**3.48**	/	**4.20**
PSPTO_1030	cellulose synthase operon protein C	/	/	**-2.34**
PSPTO_1037	hypothetical protein PSPTO_1037	/	**-2.12**	**2.91**
PSPTO_1038	hypothetical protein PSPTO_1038	/	/	**3.65**
PSPTO_1040	sensory box protein	**-2.31**	**-2.13**	/
PSPTO_1062	hypothetical protein PSPTO_1062	**6.05**	/	**5.85**
PSPTO_1066	methyl-accepting chemotaxis protein	**2.61**	**-2.98**	**7.79**
PSPTO_1073	membrane protein	**-2.27**	/	**-2.97**
PSPTO_1085	hypothetical protein PSPTO_1085	/	/	**2.61**

PSPTO_1090	hypothetical protein PSPTO_1090	**5.72**	**-2.33**	**13.34**
PSPTO_1091	hypothetical protein PSPTO_1091	/	/	**4.36**
PSPTO_1104	ribose-phosphate pyrophosphokinase	**-2.07**	/	**-2.94**
PSPTO_1114	hypothetical protein PSPTO_1114	**2.74**	/	/
PSPTO_1115	membrane protein	**4.14**	/	/
PSPTO_1117	hypothetical protein PSPTO_1117	**5.74**	/	**9.42**
PSPTO_1118	amine oxidase, flavin-containing protein	**4.63**	**-2.60**	**12.01**
PSPTO_1139	hypothetical protein PSPTO_1139	**2.62**	**5.73**	**-2.19**
PSPTO_1145	hypothetical protein PSPTO_1145	/	/	**-2.06**
PSPTO_1150	hypothetical protein PSPTO_1150	/	/	**2.03**
PSPTO_1154	alpha/beta fold family hydrolase	**3.61**	/	/
PSPTO_1159	bmp family protein	/	**-3.20**	**5.18**
PSPTO_1160	ABC transporter ATP-binding protein	/	**-2.57**	**5.64**
PSPTO_1167	hypothetical protein PSPTO_1167	/	**3.96**	**-4.88**
PSPTO_1176	hypothetical protein PSPTO_1176	**46.44**	**6.92**	**6.71**
PSPTO_1179	HopJ1 protein	/	/	**-1.95**
PSPTO_1180	hypothetical protein PSPTO_1180	/	/	**-1.93**
PSPTO_1184	hypothetical protein PSPTO_1184	/	**-2.17**	**3.44**
PSPTO_1191	hypothetical protein PSPTO_1191	/	/	**2.91**
PSPTO_1197	hypothetical protein PSPTO_1197	/	/	**-1.87**
PSPTO_1200	hypothetical protein PSPTO_1200	/	**-2.40**	/
PSPTO_1201	lipoprotein	**4.19**	/	/
PSPTO_1202	hypothetical protein PSPTO_1202	**4.64**	/	**3.70**
PSPTO_1208	regulatory protein	/	**-4.47**	**5.09**
PSPTO_1244	hypothetical protein PSPTO_1244	/	**-2.90**	**2.26**
PSPTO_1279		**-2.25**	**-1.91**	/
PSPTO_1297	hypothetical protein PSPTO_1297	**-8.16**	/	/
PSPTO_1304	hypothetical protein PSPTO_1304	**5.41**	**10.46**	/
PSPTO_1320	hypothetical protein PSPTO_1320	**2.68**	**-3.30**	**8.85**
PSPTO_1333	hypothetical protein PSPTO_1333	**2.03**	/	**2.59**
PSPTO_1334	methyl-accepting chemotaxis protein	**-4.62**	**-3.05**	/
PSPTO_1341	hypothetical protein PSPTO_1341	**2.03**	/	**2.09**
PSPTO_1342	hypothetical protein PSPTO_1342	**3.68**	/	**3.50**
PSPTO_1344	hypothetical protein PSPTO_1344	**2.70**	/	**2.20**
PSPTO_1357	aliphatic amidase	/	**2.57**	/
PSPTO_1358	ABC transporter permease	/	**3.37**	/
PSPTO_1360	bmp family protein	**2.80**	/	/
PSPTO_1362	hypothetical protein PSPTO_1362	**2.62**	/	/
PSPTO_1368	lipoprotein	/	/	**3.49**
PSPTO_1410	exchangeable effector locus protein	**2.30**	/	**2.25**
PSPTO_1411	hypothetical protein PSPTO_1411	**-1.98**	**2.18**	**-4.31**
PSPTO_1414	preprotein translocase subunit YajC	/	/	**-2.08**
PSPTO_1425	iron-binding protein IscA	/	**2.31**	**-2.23**
PSPTO_1429	hypothetical protein PSPTO_1429	/	**2.13**	**-2.87**
PSPTO_1430	nucleoside diphosphate kinase	/	/	**-2.06**
PSPTO_1433	hypothetical protein PSPTO_1433	/	/	**-1.78**
PSPTO_1436	hypothetical protein PSPTO_1436	/	/	**-1.89**
PSPTO_1438	GTP-binding protein EngA	/	**1.86**	**-3.37**

PSPTO_1460	hypothetical protein PSPTO_1460	/	**1.90**	**-2.34**
PSPTO_1466	hypothetical protein PSPTO_1466	/	/	**2.17**
PSPTO_1491	hypothetical protein PSPTO_1491	**2.06**	/	**2.08**
PSPTO_1493	methyl-accepting chemotaxis protein	**-2.32**	/	/
PSPTO_1514	hypothetical protein PSPTO_1514	**-2.74**	**-2.53**	/
PSPTO_1519	hypothetical protein PSPTO_1519	**3.23**	/	/
PSPTO_1536	uridylate kinase	/	/	**-2.48**
PSPTO_1559	esterase	/	**1.89**	/
PSPTO_1571	hypothetical protein PSPTO_1571	/	**-2.00**	**2.08**
PSPTO_1588	hypothetical protein PSPTO_1588	**4.08**	/	**3.46**
PSPTO_1590	hypothetical protein PSPTO_1590	/	/	**2.56**
PSPTO_1593	hypothetical protein PSPTO_1593	**-1.77**	/	/
PSPTO_1596	hypothetical protein PSPTO_1596	**3.10**	/	/
PSPTO_1597	ribonuclease BN	**2.78**	/	/
PSPTO_1609	hypothetical protein PSPTO_1609	**2.81**	/	/
PSPTO_1615	hypothetical protein PSPTO_1615	**-2.38**	/	/
PSPTO_1619	hypothetical protein PSPTO_1619	**2.02**	/	/
PSPTO_1623	hypothetical protein PSPTO_1623	**-22.15**	**-18.71**	/
PSPTO_1624	sodium:solute symporter family protein	**-11.02**	**-10.63**	/
PSPTO_1625	hypothetical protein PSPTO_1625	**6.87**	/	**3.09**
PSPTO_1627	short chain dehydrogenase	**2.90**	/	/
PSPTO_1651	hypothetical protein PSPTO_1651	**10.15**	/	**13.06**
PSPTO_1656	hypothetical protein PSPTO_1656	/	/	**1.84**
PSPTO_1657	hypothetical protein PSPTO_1657	**3.45**	/	**4.77**
PSPTO_1663	GNAT family acetyltransferase	/	/	**3.99**
PSPTO_1697	hypothetical protein PSPTO_1697	/	/	**-2.06**
PSPTO_1721	hypothetical protein PSPTO_1721	**-2.51**	/	**-4.30**
PSPTO_1724	hypothetical protein PSPTO_1724	/	/	**-2.73**
PSPTO_1725	hypothetical protein PSPTO_1725	/	/	**2.38**
PSPTO_1730	NAD(P)H dehydrogenase, quinone family	**3.50**	/	**2.63**
PSPTO_1761	hypothetical protein PSPTO_1761	/	/	**2.05**
PSPTO_1774	hypothetical protein PSPTO_1774	/	/	**-2.00**
PSPTO_1777	thiopurine s-methyltransferase	**-2.10**	/	**-3.73**
PSPTO_1785	hypothetical protein PSPTO_1785	**-1.89**	/	/
PSPTO_1816	hypothetical protein PSPTO_1816	/	/	**-2.53**
PSPTO_1820	hypothetical protein PSPTO_1820	/	**-1.90**	/
PSPTO_1824	hypothetical protein PSPTO_1824	**-2.19**	**-2.72**	/
PSPTO_1829	hypothetical protein PSPTO_1829	/	**1.94**	**-2.59**
PSPTO_1845	hypothetical protein PSPTO_1845	**9.56**	/	**6.56**
PSPTO_1848	hypothetical protein PSPTO_1848	**5.26**	**2.41**	/
PSPTO_1849	hypothetical protein PSPTO_1849	**4.36**	**2.24**	/
PSPTO_1869	hypothetical protein PSPTO_1869	**2.85**	/	/
PSPTO_1884	hypothetical protein PSPTO_1884	/	**-2.55**	**2.66**
PSPTO_1913	hypothetical protein PSPTO_1913	**2.90**	**-2.26**	**6.56**
PSPTO_1914	short chain dehydrogenase/reductase family oxidoreductase	**3.20**	/	**7.01**
PSPTO_1915	transferase, hexapeptide repeat protein	**2.80**	/	**6.16**
PSPTO_1919	hypothetical protein PSPTO_1919	**2.62**	**-3.08**	**8.06**
PSPTO_1953	hypothetical protein PSPTO_1953	**-3.13**	**-2.29**	/

PSPTO_1987	chemotaxis protein CheW	/	**-2.21**	/
PSPTO_1992	hypothetical protein PSPTO_1992	**-2.82**	/	/
PSPTO_2013	hypothetical protein PSPTO_2013	**-4.12**	/	**-2.81**
PSPTO_2017	hypothetical protein PSPTO_2017	/	/	**-1.99**
PSPTO_2038	hypothetical protein PSPTO_2038	**2.12**	**1.94**	/
PSPTO_2041	hypothetical protein PSPTO_2041	**-2.76**	/	/
PSPTO_2042	hypothetical protein PSPTO_2042	/	**1.98**	/
PSPTO_2046	ARD/ARD\' family protein	/	**1.79**	**-2.50**
PSPTO_2049	hypothetical protein PSPTO_2049	**3.97**	**5.16**	/
PSPTO_2051	ankyrin domain protein	**2.58**	/	/
PSPTO_2052	hypothetical protein PSPTO_2052	/	/	**-2.06**
PSPTO_2054	hypothetical protein PSPTO_2054	**2.26**	**2.22**	/
PSPTO_2059	hypothetical protein PSPTO_2059	**3.03**	/	**1.97**
PSPTO_2060	hypothetical protein PSPTO_2060	**2.83**	/	**1.81**
PSPTO_2062	hypothetical protein PSPTO_2062	**3.86**	/	**3.39**
PSPTO_2069	hypothetical protein PSPTO_2069	**6.18**	/	**5.46**
PSPTO_2073	hypothetical protein PSPTO_2073	/	/	**2.27**
PSPTO_2075	hypothetical protein PSPTO_2075	/	**-2.37**	**4.46**
PSPTO_2077	hypothetical protein PSPTO_2077	/	/	**4.13**
PSPTO_2080	hypothetical protein PSPTO_2080	/	/	**4.48**
PSPTO_2083	hypothetical protein PSPTO_2083	**3.05**	**-1.92**	**5.87**
PSPTO_2100	hypothetical protein PSPTO_2100	/	/	**-2.05**
PSPTO_2104	major facilitator family transporter	**-3.82**	**-2.59**	/
PSPTO_2108	hypothetical protein PSPTO_2108	/	/	**1.99**
PSPTO_2114	hypothetical protein PSPTO_2114	**5.89**	**4.81**	/
PSPTO_2124	hypothetical protein PSPTO_2124	**2.28**	/	/
PSPTO_2125	hypothetical protein PSPTO_2125	/	/	**2.67**
PSPTO_2217	hypothetical protein PSPTO_2217	/	/	**1.98**
PSPTO_2224	hypothetical protein PSPTO_2224	**4.34**	/	**2.68**
PSPTO_2231	YD repeat protein, partial	/	/	**1.99**
PSPTO_2233	hypothetical protein PSPTO_2233	**5.97**	**1.81**	**3.30**
PSPTO_2236	hypothetical protein PSPTO_2236	**7.56**	**3.30**	/
PSPTO_2237	hypothetical protein PSPTO_2237	**2.88**	/	**2.43**
PSPTO_2238	lipoprotein	**6.51**	/	**4.04**
PSPTO_2239	YD repeat protein	**2.17**	/	**1.80**
PSPTO_2247	lipoprotein	/	**2.08**	/
PSPTO_2251	hypothetical protein PSPTO_2251	**1.98**	/	/
PSPTO_2253	hypothetical protein PSPTO_2253	/	**-15.88**	**28.26**
PSPTO_2254	methyl-accepting chemotaxis protein	/	**-3.61**	**2.63**
PSPTO_2260	hypothetical protein PSPTO_2260	**3.70**	**1.79**	**2.07**
PSPTO_2318	hypothetical protein PSPTO_2318	/	/	**2.77**
PSPTO_2325	hypothetical protein PSPTO_2325	/	**3.21**	**-3.42**
PSPTO_2350	lipoprotein	/	**1.82**	/
PSPTO_2374	hypothetical protein PSPTO_2374	/	**-6.98**	**6.84**
PSPTO_2375	lipoprotein	/	**1.99**	/
PSPTO_2390	hypothetical protein PSPTO_2390	**3.99**	/	**2.85**
PSPTO_2422	hypothetical protein PSPTO_2422	/	/	**2.24**
PSPTO_2441	methyl-accepting chemotaxis protein	**-2.59**	**-3.88**	/

PSPTO_2442	chemotaxis protein CheW	**-2.26**	**-6.50**	**2.87**
PSPTO_2443	hypothetical protein PSPTO_2443	/	/	**2.53**
PSPTO_2471	membrane protein	**2.68**	/	/
PSPTO_2472	methyl-accepting chemotaxis protein	**-2.70**	**-2.28**	/
PSPTO_2475	methyl-accepting chemotaxis protein	**-1.99**	**-2.20**	/
PSPTO_2480	methyl-accepting chemotaxis protein	**-4.84**	**-2.24**	**-2.16**
PSPTO_2489	hypothetical protein PSPTO_2489	**2.04**	/	/
PSPTO_2501	hypothetical protein PSPTO_2501	**-1.95**	**-1.86**	/
PSPTO_2506	lipoprotein	**2.38**	/	/
PSPTO_2511	methyl-accepting chemotaxis protein	**-5.29**	**-2.48**	**-2.13**
PSPTO_2514	hypothetical protein PSPTO_2514	/	/	**1.93**
PSPTO_2515	lipoprotein	/	/	**2.21**
PSPTO_2531	hypothetical protein PSPTO_2531	/	/	**2.10**
PSPTO_2539	secreted protein Hcp	/	**-2.37**	/
PSPTO_2579	carbonic anhydrase-like protein	/	**-3.07**	/
PSPTO_2589	hypothetical protein PSPTO_2589	**4.71**	/	**5.88**
PSPTO_2615	GAF domain-containing protein	/	**-1.87**	/
PSPTO_2616	methyl-accepting chemotaxis protein	**-5.20**	**-2.78**	**-1.87**
PSPTO_2620	hypothetical protein PSPTO_2620	/	**-10.23**	**14.87**
PSPTO_2623	phenazine biosynthesis protein, PhzF family	**3.13**	/	**3.66**
PSPTO_2647	hypothetical protein PSPTO_2647	**1.88**	/	**2.17**
PSPTO_2648	hypothetical protein PSPTO_2648	**2.54**	/	**2.42**
PSPTO_2650	membrane protein	/	**2.17**	/
PSPTO_2653		**-2.06**	/	/
PSPTO_2659	NAD(P)H-flavin oxidoreductase	/	**2.15**	/
PSPTO_2677	short chain dehydrogenase/reductase family oxidoreductase	**7.92**	/	**11.25**
PSPTO_2683	hypothetical protein PSPTO_2683	/	/	**4.18**
PSPTO_2684	hypothetical protein PSPTO_2684	/	/	**1.83**
PSPTO_2692	hypothetical protein PSPTO_2692	**5.53**	/	**4.53**
PSPTO_2695	hypothetical protein PSPTO_2695	**2.13**	/	/
PSPTO_2696	mutT/nudix family protein	**2.30**	/	/
PSPTO_2698	hypothetical protein PSPTO_2698	/	/	**2.57**
PSPTO_2704		**-3.86**	**-3.06**	/
PSPTO_2722	oxidoreductase, Gfo/Idh/MocA family	/	/	**2.67**
PSPTO_2723	short chain dehydrogenase	/	/	**3.23**
PSPTO_2724	hypothetical protein PSPTO_2724	**3.42**	/	**5.18**
PSPTO_2748	hypothetical protein PSPTO_2748	**2.23**	/	/
PSPTO_2758	short-chain dehydrogenase/reductase family oxidoreductase	/	/	**1.89**
PSPTO_2759	hypothetical protein PSPTO_2759	**3.26**	/	**3.84**
PSPTO_2766	ABC transporter ATP-binding protein	/	/	**-2.19**
PSPTO_2769	lipoprotein	**2.99**	/	**1.82**
PSPTO_2784	hypothetical protein PSPTO_2784	/	/	**1.93**
PSPTO_2790	hypothetical protein PSPTO_2790	/	**2.07**	/
PSPTO_2796	hypothetical protein PSPTO_2796	/	**-2.01**	**2.32**
PSPTO_2815	hypothetical protein PSPTO_2815	/	/	**-2.92**
PSPTO_2816	alkylhydroperoxidase AhpD domain-containing protein	/	/	**-4.48**
PSPTO_2843	hypothetical protein PSPTO_2843	/	**-2.14**	/

PSPTO_2855	DNA-binding protein	**6.39**	**10.13**	/
PSPTO_2858	hypothetical protein PSPTO_2858	/	/	**3.42**
PSPTO_2859	hypothetical protein PSPTO_2859	**5.68**	/	**8.04**
PSPTO_2866	cupin family protein	/	/	**3.66**
PSPTO_2870	hypothetical protein PSPTO_2870	/	**-2.43**	**3.55**
PSPTO_2871	hypothetical protein PSPTO_2871	**3.15**	/	**4.60**
PSPTO_2872	HopL1 protein	**3.37**	/	**2.72**
PSPTO_2873	hypothetical protein PSPTO_2873	/	/	**2.47**
PSPTO_2874	ppkA-related protein	**2.08**	/	**2.96**
PSPTO_2877	hypothetical protein PSPTO_2877	**4.24**	/	**6.94**
PSPTO_2878	lipoprotein	**3.79**	/	**4.47**
PSPTO_2881	hypothetical protein PSPTO_2881	**-8.19**	/	/
PSPTO_2882		**-3.95**	/	/
PSPTO_2883	methyl-accepting chemotaxis protein	**-3.44**	**-2.31**	/
PSPTO_2893	PAP2 superfamily protein	/	/	**2.17**
PSPTO_2894	lectin repeat domain protein	**16.70**	**6.34**	**2.63**
PSPTO_2895	hypothetical protein PSPTO_2895	**3.42**	/	**4.29**
PSPTO_2897	hypothetical protein PSPTO_2897	/	/	**3.29**
PSPTO_2930	hypothetical protein PSPTO_2930	/	/	**-1.95**
PSPTO_2936	LamB/YcsF family protein	/	**-2.30**	**2.94**
PSPTO_2939	hypothetical protein PSPTO_2939	/	/	**2.10**
PSPTO_2980	2OG-Fe(II) oxygenase family oxidoreductase	/	**-2.25**	**1.93**
PSPTO_2997	methyl-accepting chemotaxis protein	/	**-2.38**	/
PSPTO_3016	hypothetical protein PSPTO_3016	**3.21**	/	/
PSPTO_3020	hypothetical protein PSPTO_3020	/	**1.91**	**-2.02**
PSPTO_3038	hypothetical protein PSPTO_3038	**4.00**	**1.94**	/
PSPTO_3050	AraC family transcriptional regulator	/	/	**2.03**
PSPTO_3061	LysR family transcriptional regulator	/	/	**3.45**
PSPTO_3067	hypothetical protein PSPTO_3067	**-2.96**	**2.96**	**-8.76**
PSPTO_3092	hypothetical protein PSPTO_3092	**3.07**	/	**3.76**
PSPTO_3094	lipoprotein	/	**-3.08**	**2.28**
PSPTO_3105	hypothetical protein PSPTO_3105	/	/	**1.86**
PSPTO_3115	hypothetical protein PSPTO_3115	/	**1.96**	/
PSPTO_3120	Cof-like hydrolase family protein	/	/	**2.06**
PSPTO_3123	hypothetical protein PSPTO_3123	**-9.43**	**-2.71**	**-3.48**
PSPTO_3124	GNAT family acetyltransferase	**2.30**	/	**1.99**
PSPTO_3129	hypothetical protein PSPTO_3129	**2.91**	/	**2.92**
PSPTO_3131	hypothetical protein PSPTO_3131	/	/	**2.70**
PSPTO_3132	hypothetical protein PSPTO_3132	/	/	**3.18**
PSPTO_3133	methyltransferase	/	/	**2.64**
PSPTO_3135	hypothetical protein PSPTO_3135	**3.83**	/	**2.44**
PSPTO_3157	hypothetical protein PSPTO_3157	/	**-3.14**	**4.76**
PSPTO_3165	lipoprotein	**2.57**	**2.76**	/
PSPTO_3166	hypothetical protein PSPTO_3166	**8.60**	/	**14.74**
PSPTO_3168	hypothetical protein PSPTO_3168	**4.72**	/	**5.44**
PSPTO_3177	hypothetical protein PSPTO_3177	**3.75**	/	**5.84**
PSPTO_3189	hypothetical protein PSPTO_3189	/	**2.32**	**-2.29**
PSPTO_3192	amidase family protein	**-2.03**	/	**-2.47**

PSPTO_3199	luciferase family protein	**3.54**	/	**2.32**
PSPTO_3200	hypothetical protein PSPTO_3200	**9.46**	/	**4.64**
PSPTO_3201	hypothetical protein PSPTO_3201	**4.53**	/	**2.85**
PSPTO_3202	oxidoreductase, 2-nitropropane dioxygenase family	**2.75**	/	**2.13**
PSPTO_3203	hypothetical protein PSPTO_3203	**5.87**	/	**3.88**
PSPTO_3206		/	/	**2.19**
PSPTO_3226	hypothetical protein PSPTO_3226	/	**2.00**	**-3.29**
PSPTO_3228	hypothetical protein PSPTO_3228	/	/	**-2.24**
PSPTO_3236	hypothetical protein PSPTO_3236	/	**2.23**	**-2.33**
PSPTO_3264	alpha/beta fold family hydrolase	**2.52**	/	/
PSPTO_3270	hypothetical protein PSPTO_3270	**2.55**	/	**2.74**
PSPTO_3271	hypothetical protein PSPTO_3271	**3.41**	/	**2.79**
PSPTO_3291	methyl-accepting chemotaxis protein	**-2.48**	/	/
PSPTO_3299	3-hydroxyacyl-CoA-acyl carrier protein transferase	/	**-2.61**	**2.17**
PSPTO_3305	hypothetical protein PSPTO_3305	**2.08**	/	/
PSPTO_3306	phospholipase/carboxylesterase family protein	/	/	**-2.38**
PSPTO_3308	general secretion pathway protein N	/	**2.88**	**-2.96**
PSPTO_3331	protease inhibitor Inh	**3.29**	/	/
PSPTO_3359	hypothetical protein PSPTO_3359	/	/	**-2.44**
PSPTO_3361	hypothetical protein PSPTO_3361	/	**1.86**	**-3.25**
PSPTO_3362	GNAT family acetyltransferase	/	/	**-2.27**
PSPTO_3386	hypothetical protein PSPTO_3386	**2.21**	/	/
PSPTO_3390	tail protein D	**-7.44**	/	**-6.72**
PSPTO_3391	tail protein X	**-3.19**	/	**-3.21**
PSPTO_3392	hypothetical protein PSPTO_3392	**-3.50**	/	**-4.17**
PSPTO_3393	tail tape measure protein	**-6.37**	/	**-4.63**
PSPTO_3394	hypothetical protein PSPTO_3394	**-14.33**	/	**-9.99**
PSPTO_3395	major tail tube protein	**-18.55**	/	**-8.81**
PSPTO_3396	major tail sheath protein	**-16.58**	/	**-8.26**
PSPTO_3397	tail fiber assembly domain protein	**-4.83**	/	**-4.30**
PSPTO_3398	tail fiber protein H	**-5.70**	/	**-4.09**
PSPTO_3399	tail protein I	**-7.78**	/	**-5.79**
PSPTO_3400	baseplate assembly protein J	**-6.20**	/	**-5.32**
PSPTO_3401	baseplate assembly protein W	**-5.05**	/	**-4.57**
PSPTO_3402	hypothetical protein PSPTO_3402	**-18.18**	/	**-6.65**
PSPTO_3403	baseplate assembly protein V	**-10.36**	/	**-7.40**
PSPTO_3404	hypothetical protein PSPTO_3404	**-9.91**	/	**-5.71**
PSPTO_3405	hypothetical protein PSPTO_3405	**-12.10**	/	**-6.84**
PSPTO_3406	hypothetical protein PSPTO_3406	**-15.58**	/	**-9.69**
PSPTO_3407	hypothetical protein PSPTO_3407	**-16.15**	**-2.05**	**-7.89**
PSPTO_3408	hypothetical protein PSPTO_3408	**-8.25**	/	**-7.02**
PSPTO_3410	portal protein	**-6.58**	/	**-5.67**
PSPTO_3412	terminase large subunit	**-8.12**	/	**-5.58**
PSPTO_3413	hypothetical protein PSPTO_3413	**-16.29**	**-2.81**	**-5.79**
PSPTO_3414	hypothetical protein PSPTO_3414	**-9.01**	/	**-6.64**
PSPTO_3415	hypothetical protein PSPTO_3415	**-12.85**	/	**-8.46**
PSPTO_3416	holin	**-9.99**	/	**-4.56**
PSPTO_3418	DNA primase domain protein	**-2.12**	/	**-2.86**

PSPTO_3420	hypothetical protein PSPTO_3420	**-2.28**	/	**-2.06**
PSPTO_3422	hypothetical protein PSPTO_3422	**-3.14**	/	**-2.88**
PSPTO_3423	hypothetical protein PSPTO_3423	**-3.19**	/	**-3.03**
PSPTO_3424	hypothetical protein PSPTO_3424	**-2.81**	/	**-2.68**
PSPTO_3425	transcriptional regulator	**-2.42**	/	**-2.59**
PSPTO_3426	hypothetical protein PSPTO_3426	**-4.18**	/	**-4.09**
PSPTO_3428	hypothetical protein PSPTO_3428	**-3.82**	/	**-4.60**
PSPTO_3435		/	/	**3.16**
PSPTO_3477	hypothetical protein PSPTO_3477	**-2.39**	/	**-2.37**
PSPTO_3478	hypothetical protein PSPTO_3478	**3.17**	/	**2.75**
PSPTO_3480	methyl-accepting chemotaxis protein	**-2.30**	**-2.46**	/
PSPTO_3481	hypothetical protein PSPTO_3481	**20.08**	**4.22**	**4.76**
PSPTO_3483	hypothetical protein PSPTO_3483	**24.00**	**3.74**	**6.42**
PSPTO_3484	lipoprotein	**12.95**	**4.81**	**2.69**
PSPTO_3485	lipase family protein	**10.43**	**4.25**	**2.46**
PSPTO_3504	endonuclease/exonuclease/phosphatase family protein	**2.07**	/	/
PSPTO_3515	hypothetical protein PSPTO_3515	/	/	**-2.04**
PSPTO_3519	hypothetical protein PSPTO_3519	/	/	**-2.95**
PSPTO_3521	ABC transporter ATP-binding protein	/	**1.95**	**-2.82**
PSPTO_3525	hypothetical protein PSPTO_3525	/	**-3.86**	**2.23**
PSPTO_3542	hypothetical protein PSPTO_3542	**3.68**	/	**3.42**
PSPTO_3543	hypothetical protein PSPTO_3543	/	/	**3.09**
PSPTO_3544	hypothetical protein PSPTO_3544	**-2.45**	**-2.17**	/
PSPTO_3546	hypothetical protein PSPTO_3546	**4.26**	/	/
PSPTO_3558	glcG protein	**2.17**	/	/
PSPTO_3567	hypothetical protein PSPTO_3567	**3.62**	/	**5.57**
PSPTO_3569	hypothetical protein PSPTO_3569	**10.28**	**-2.04**	**21.01**
PSPTO_3572	hypothetical protein PSPTO_3572	/	**-2.61**	**5.35**
PSPTO_3580	methyl-accepting chemotaxis protein	/	**-1.90**	**2.80**
PSPTO_3581	GNAT family acetyltransferase	/	/	**2.60**
PSPTO_3583	ankyrin domain protein	**6.77**	**-3.13**	**21.19**
PSPTO_3600	oxidoreductase, molybdopterin-binding protein	/	/	**3.09**
PSPTO_3602	hypothetical protein PSPTO_3602	**3.09**	/	**3.69**
PSPTO_3605	lyase	/	**-5.73**	**3.93**
PSPTO_3616	hypothetical protein PSPTO_3616	/	/	**2.38**
PSPTO_3618	membrane protein	/	/	**2.78**
PSPTO_3636	hypothetical protein PSPTO_3636, partial	**-2.51**	/	/
PSPTO_3637	FlhB domain-containing protein	**-2.69**	/	/
PSPTO_3645	hypothetical protein PSPTO_3645	/	/	**-1.76**
PSPTO_3650	hypothetical protein PSPTO_3650	/	/	**2.75**
PSPTO_3655	hypothetical protein PSPTO_3655	**2.61**	/	**2.41**
PSPTO_3667	hypothetical protein PSPTO_3667	/	/	**2.98**
PSPTO_3672	membrane protein	/	**2.68**	/
PSPTO_3685	methyl-accepting chemotaxis protein	/	**-9.22**	**5.87**
PSPTO_3688	membrane protein	**6.29**	/	**5.31**
PSPTO_3689	hypothetical protein PSPTO_3689	**3.05**	/	**5.30**
PSPTO_3690	hypothetical protein PSPTO_3690	**5.41**	/	**8.83**
PSPTO_3691	ea59 protein	**8.71**	/	**11.79**

PSPTO_3693	hypothetical protein PSPTO_3693	**-1.95**	/	**-2.31**
PSPTO_3700	oxidoreductase, aldo/keto reductase family	/	**2.57**	/
PSPTO_3709	hypothetical protein PSPTO_3709	/	**2.36**	/
PSPTO_3720	ABC transporter ATP-binding protein	/	/	**-2.26**
PSPTO_3723	DNA-binding protein HU-beta	**-1.82**	/	**-1.94**
PSPTO_3730	membrane protein	**17.16**	/	**22.46**
PSPTO_3756	hypothetical protein PSPTO_3756	**2.14**	/	/
PSPTO_3763	TPR domain-containing protein	/	/	**-1.97**
PSPTO_3764	hypothetical protein PSPTO_3764	/	/	**-2.63**
PSPTO_3781	hypothetical protein PSPTO_3781	/	**1.76**	/
PSPTO_3782	hypothetical protein PSPTO_3782	/	**1.80**	/
PSPTO_3783	hypothetical protein PSPTO_3783	**1.84**	/	/
PSPTO_3795	hypothetical protein PSPTO_3795	/	/	**2.48**
PSPTO_3809	oxidoreductase	/	**1.94**	**-2.12**
PSPTO_3820	hypothetical protein PSPTO_3820	/	/	**3.09**
PSPTO_3821	lipoprotein	**2.58**	/	**4.80**
PSPTO_3836	hypothetical protein PSPTO_3836	/	/	**-1.75**
PSPTO_3865	hypothetical protein PSPTO_3865	**20.97**	**1.96**	**10.72**
PSPTO_3866	hypothetical protein PSPTO_3866	**11.31**	/	**5.65**
PSPTO_3868	hypothetical protein PSPTO_3868	**6.72**	/	**5.31**
PSPTO_3869	hypothetical protein PSPTO_3869	**4.96**	/	**4.91**
PSPTO_3870	patatin family protein	**-2.01**	/	/
PSPTO_3879	hypothetical protein PSPTO_3879	**5.26**	/	**4.75**
PSPTO_3880	polyamine ABC transporter permease	**3.25**	/	**2.63**
PSPTO_3895	hypothetical protein PSPTO_3895	**-1.95**	/	**-3.12**
PSPTO_3903	hypothetical protein PSPTO_3903	**2.21**	/	/
PSPTO_3917	hypothetical protein PSPTO_3917	**2.36**	/	**2.14**
PSPTO_3925	gas vesicle protein	**3.04**	/	**5.18**
PSPTO_3926		/	/	**2.48**
PSPTO_3927	hypothetical protein PSPTO_3927	/	**-2.14**	**2.98**
PSPTO_3929	cold shock domain family protein	/	/	**3.14**
PSPTO_3951	metallo-beta-lactamase superfamily protein	/	/	**-1.99**
PSPTO_3968	exsB protein	/	**1.92**	**-2.49**
PSPTO_3974	tolR protein	/	/	**-1.88**
PSPTO_3989	lipoprotein	/	/	**-2.20**
PSPTO_3991	membrane protein	**-1.92**	/	/
PSPTO_3994	hypothetical protein PSPTO_3994	**3.62**	/	**3.98**
PSPTO_4009	regulatory protein Cro	/	**3.83**	/
PSPTO_4012	hypothetical protein PSPTO_4012	/	/	**2.29**
PSPTO_4032	recX protein	/	/	**-1.76**
PSPTO_4047	hypothetical protein PSPTO_4047	**2.95**	/	**4.00**
PSPTO_4070	hypothetical protein PSPTO_4070	**14.11**	**2.69**	**5.25**
PSPTO_4082	short chain dehydrogenase/reductase family oxidoreductase	/	/	**2.56**
PSPTO_4093	leucine rich repeat domain protein	/	/	**2.07**
PSPTO_4114	hypothetical protein PSPTO_4114	/	/	**-2.23**
PSPTO_4145	cold shock protein CapB	**-1.94**	/	**-2.44**
PSPTO_4159	bacterioferritin-associated ferredoxin	/	**-6.42**	/
PSPTO_4160	bacterioferritin	/	**1.64**	**-2.47**

PSPTO_4163	hypothetical protein PSPTO_4163	**4.54**	/	**4.16**
PSPTO_4185	membrane protein	**3.38**	/	/
PSPTO_4198	cobalamin synthesis protein/P47K family protein	/	/	**-2.18**
PSPTO_4206	globin family protein	**-2.61**	**-2.36**	/
PSPTO_4207	hypothetical protein PSPTO_4207	**-3.68**	**-3.17**	/
PSPTO_4209	hypothetical protein PSPTO_4209	**-2.61**	**-2.51**	/
PSPTO_4216	GTP-binding protein Era	**-2.01**	**2.04**	**-4.10**
PSPTO_4218	hypothetical protein PSPTO_4218	/	/	**-3.55**
PSPTO_4246	hypothetical protein PSPTO_4246	/	/	**3.56**
PSPTO_4248	3-hydroxyacyl-CoA-acyl carrier protein transferase	**19.14**	/	**27.52**
PSPTO_4268		**-4.86**	/	**-5.82**
PSPTO_4272	hypothetical protein PSPTO_4272	/	**-3.52**	**2.72**
PSPTO_4284	GNAT family acetyltransferase	/	**-3.23**	**3.37**
PSPTO_4287	binary cytotoxin component	**2.31**	/	/
PSPTO_4289	hypothetical protein PSPTO_4289	/	/	**-2.23**
PSPTO_4297	hypothetical protein PSPTO_4297	**-2.57**	/	**-1.92**
PSPTO_4298	hypothetical protein PSPTO_4298	**-1.87**	/	/
PSPTO_4299	hypothetical protein PSPTO_4299	/	/	**-1.95**
PSPTO_4301	hypothetical protein PSPTO_4301	**-1.95**	/	**-3.22**
PSPTO_4313	hypothetical protein PSPTO_4313	/	/	**-2.07**
PSPTO_4315	transcriptional regulator	/	/	**-1.90**
PSPTO_4324	hypothetical protein PSPTO_4324	**-7.43**	**-5.54**	/
PSPTO_4325	hypothetical protein PSPTO_4325	**-4.97**	**-3.59**	/
PSPTO_4326	hypothetical protein PSPTO_4326	**-4.55**	**-2.02**	**-2.25**
PSPTO_4327	hypothetical protein PSPTO_4327	**-4.43**	/	**-3.60**
PSPTO_4333	moxR protein	**-1.92**	/	/
PSPTO_4334	hypothetical protein PSPTO_4334	**-2.25**	/	/
PSPTO_4336	lipoprotein	**-3.72**	**-2.14**	/
PSPTO_4350	hypothetical protein PSPTO_4350	/	**2.35**	**-1.94**
PSPTO_4357	major facilitator family transporter	/	**2.06**	**-2.19**
PSPTO_4359	hypothetical protein PSPTO_4359	**-2.54**	/	/
PSPTO_4368	lipoprotein	**-1.97**	/	/
PSPTO_4372	hypothetical protein PSPTO_4372	/	/	**-2.31**
PSPTO_4378	hypothetical protein PSPTO_4378	/	/	**-4.03**
PSPTO_4379	hypothetical protein PSPTO_4379	/	/	**-1.97**
PSPTO_4381	hypothetical protein PSPTO_4381	**-2.80**	/	/
PSPTO_4384	hypothetical protein PSPTO_4384	**-2.22**	/	**-2.31**
PSPTO_4386	hypothetical protein PSPTO_4386	**2.56**	/	**2.91**
PSPTO_4393	hypothetical protein PSPTO_4393	/	/	**-1.97**
PSPTO_4428	ATPase	/	/	**-2.22**
PSPTO_4467	hypothetical protein PSPTO_4467	/	**1.95**	**-3.01**
PSPTO_4472	rod shape-determining protein MreB	/	/	**-2.14**
PSPTO_4482	hypothetical protein PSPTO_4482	/	**2.43**	**-2.43**
PSPTO_4484	hypothetical protein PSPTO_4484	/	**3.95**	**-2.36**
PSPTO_4492	hypothetical protein PSPTO_4492	/	/	**-2.32**
PSPTO_4516	hypothetical protein PSPTO_4516	**2.79**	/	**3.03**
PSPTO_4536	peptide ABC transporter ATP-binding protein	**14.58**	**3.84**	**3.80**
PSPTO_4539	LuxR family transcriptional regulator, autoinducer-regulated	**15.34**	**1.74**	**8.81**

PSPTO_4545	hypothetical protein PSPTO_4545	/	/	**2.78**
PSPTO_4546	hypothetical protein PSPTO_4546	**2.45**	/	/
PSPTO_4555	hypothetical protein PSPTO_4555	**2.25**	/	/
PSPTO_4574	hypothetical protein PSPTO_4574	**2.24**	/	**2.36**
PSPTO_4584	hypothetical protein PSPTO_4584	/	/	**2.78**
PSPTO_4586	hypothetical protein PSPTO_4586	**2.32**	/	**2.31**
PSPTO_4603	site-specific recombinase, phage integrase family	/	/	**2.67**
PSPTO_4605	hypothetical protein PSPTO_4605	/	**-1.98**	**3.77**
PSPTO_4606	hypothetical protein PSPTO_4606	/	/	**2.46**
PSPTO_4607	hypothetical protein PSPTO_4607	**5.13**	/	**7.59**
PSPTO_4611	hypothetical protein PSPTO_4611	**2.03**	/	**2.11**
PSPTO_4624	methyl-accepting chemotaxis protein	/	**-2.67**	/
PSPTO_4627	hypothetical protein PSPTO_4627	/	**-2.09**	**4.09**
PSPTO_4628	hypothetical protein PSPTO_4628	**3.78**	/	**5.01**
PSPTO_4633	hypothetical protein PSPTO_4633	/	/	**2.02**
PSPTO_4634	N-acetylmuramoyl-L-alanine amidase	/	/	**2.11**
PSPTO_4636	cobalamin synthesis protein/P47K family protein	**-2.92**	/	**-2.25**
PSPTO_4637	hypothetical protein PSPTO_4637	**-4.86**	**-2.77**	/
PSPTO_4639	hypothetical protein PSPTO_4639	/	**-2.05**	**3.20**
PSPTO_4653	xanthine/uracil permease family protein	/	**1.91**	**-3.18**
PSPTO_4657	zinc metallopeptidase	**2.11**	/	/
PSPTO_4658	hypothetical protein PSPTO_4658	/	/	**1.87**
PSPTO_4676	hypothetical protein PSPTO_4676	/	**-2.03**	**2.35**
PSPTO_4719	hypothetical protein PSPTO_4719	/	/	**5.63**
PSPTO_4741	hypothetical protein PSPTO_4741	/	**2.94**	**-4.53**
PSPTO_4742	site-specific recombinase, phage integrase family	**-2.06**	/	**-3.65**
PSPTO_4743	hypothetical protein PSPTO_4743	**-2.91**	/	**-3.12**
PSPTO_4752	hypothetical protein PSPTO_4752	/	/	**1.83**
PSPTO_4754	hypothetical protein PSPTO_4754	/	/	**3.43**
PSPTO_4761	hypothetical protein PSPTO_4761	/	**-2.06**	**2.36**
PSPTO_4762	von Willebrand factor type A domain-containing protein	/	**-1.81**	**2.67**
PSPTO_4763	hypothetical protein PSPTO_4763	/	**-1.97**	/
PSPTO_4770	hypothetical protein PSPTO_4770	/	**-2.12**	**4.14**
PSPTO_4772	hypothetical protein PSPTO_4772	**3.68**	/	**2.92**
PSPTO_4791	hypothetical protein PSPTO_4791	**2.12**	/	/
PSPTO_4797	hypothetical protein PSPTO_4797	/	**-3.29**	/
PSPTO_4803	hypothetical protein PSPTO_4803	/	/	**-3.13**
PSPTO_4810	hypothetical protein PSPTO_4810	/	/	**4.22**
PSPTO_4820	hypothetical protein PSPTO_4820	/	/	**-2.10**
PSPTO_4826	hypothetical protein PSPTO_4826	/	/	**-1.96**
PSPTO_4839	hypothetical protein PSPTO_4839	**2.33**	/	/
PSPTO_4845	lipoprotein	**2.65**	/	**1.97**
PSPTO_4848	response regulator	**11.43**	/	**16.91**
PSPTO_4849	hypothetical protein PSPTO_4849	**71.98**	/	**107.53**
PSPTO_4857	hypothetical protein PSPTO_4857	**5.51**	/	**5.98**
PSPTO_4859	3-dehydroquinate dehydratase	**-2.12**	/	/
PSPTO_4863	hypothetical protein PSPTO_4863	/	/	**-1.85**

PSPTO_4865	global DNA-binding transcriptional dual regulator Fis	**-1.94**	/	**-2.85**
PSPTO_4870	hypothetical protein PSPTO_4870	**2.79**	/	/
PSPTO_4871	hypothetical protein PSPTO_4871	**2.95**	**2.38**	/
PSPTO_4872	hypothetical protein PSPTO_4872	/	**2.79**	/
PSPTO_4873	hypothetical protein PSPTO_4873	/	**2.32**	/
PSPTO_4883	membrane protein	/	**2.29**	**-2.70**
PSPTO_4900	phosphate starvation-inducible protein PsiF	**3.13**	**-2.04**	**6.38**
PSPTO_4925	hypothetical protein PSPTO_4925	**-3.17**	/	**-1.97**
PSPTO_4931	membrane protein	/	**2.48**	**-4.19**
PSPTO_4936	methyl-accepting chemotaxis protein	/	**-2.11**	**2.07**
PSPTO_4949	membrane protein	/	/	**-2.18**
PSPTO_4965	Ser/Thr protein phosphatase	/	/	**1.85**
PSPTO_4967	hypothetical protein PSPTO_4967	**14.24**	/	**11.72**
PSPTO_4986	membrane protein	/	/	**-2.25**
PSPTO_4988	hypothetical protein PSPTO_4988	/	**1.98**	**-3.49**
PSPTO_4989	hypothetical protein PSPTO_4989	/	/	**-3.48**
PSPTO_4990	hypothetical protein PSPTO_4990	/	/	**-2.49**
PSPTO_5022	hypothetical protein PSPTO_5022	**4.35**	/	/
PSPTO_5071	hypothetical protein PSPTO_5071	**-2.37**	/	**-1.96**
PSPTO_5073	hypothetical protein PSPTO_5073	**-9.11**	/	**-4.98**
PSPTO_5095	membrane protein	**-5.04**	/	**-4.55**
PSPTO_5100	4-hydroxybenzoyl-CoA thioesterase	**-3.54**	/	**-4.31**
PSPTO_5102	membrane protein	**-3.81**	/	**-3.57**
PSPTO_5119	hypothetical protein PSPTO_5119	**4.57**	**2.10**	**2.17**
PSPTO_5124	hypothetical protein PSPTO_5124	**-2.56**	**2.23**	**-5.71**
PSPTO_5143		**11.37**	/	**8.38**
PSPTO_5144	poly(3-hydroxyalkanoate) depolymerase	**7.02**	/	**4.61**
PSPTO_5147	polyhydroxyalkanoate granule-associated protein PhaF	/	/	**2.14**
PSPTO_5160	methyl-accepting chemotaxis protein	**-2.67**	/	/
PSPTO_5166	membrane protein	/	/	**2.16**
PSPTO_5169	lipoprotein	/	**2.01**	/
PSPTO_5175	hypothetical protein PSPTO_5175	**3.20**	/	**2.19**
PSPTO_5201	hypothetical protein PSPTO_5201	**2.57**	/	**3.69**
PSPTO_5202	hypothetical protein PSPTO_5202	**2.91**	/	**3.15**
PSPTO_5203	hypothetical protein PSPTO_5203	**4.06**	/	**3.63**
PSPTO_5204	EF hand domain-containing protein	**4.07**	/	**3.10**
PSPTO_5218	hypothetical protein PSPTO_5218	**6.58**	/	**6.01**
PSPTO_5220	AraC family transcriptional regulator	/	/	**2.27**
PSPTO_5234	hypothetical protein PSPTO_5234	**4.08**	/	**3.29**
PSPTO_5332	trypsin domain-containing protein	**3.00**	**2.16**	/
PSPTO_5385	hypothetical protein PSPTO_5385	**4.07**	/	**4.95**
PSPTO_5390	hypothetical protein PSPTO_5390	**4.77**	**2.41**	/
PSPTO_5404	LysM domain/BON superfamily protein	**3.33**	/	**3.54**
PSPTO_5408	hypothetical protein PSPTO_5408	/	/	**1.92**
PSPTO_5420	hypothetical protein PSPTO_5420	/	**1.94**	/
PSPTO_5427	hypothetical protein PSPTO_5427	/	/	**-2.17**
PSPTO_5430	hypothetical protein PSPTO_5430	/	**2.10**	/

PSPTO_5431	hypothetical protein PSPTO_5431	/	**2.30**	/
PSPTO_5432	hypothetical protein PSPTO_5432	/	**2.12**	/
PSPTO_5435	secreted protein Hcp	**3.83**	/	**4.08**
PSPTO_5437	hypothetical protein PSPTO_5437	**6.46**	/	**4.52**
PSPTO_5447	hypothetical protein PSPTO_5447	**4.33**	/	**3.44**
PSPTO_5455	hypothetical protein PSPTO_5455	**2.81**	/	/
PSPTO_5481	hypothetical protein PSPTO_5481	**-2.32**	/	**-2.05**
PSPTO_5491	hypothetical protein PSPTO_5491	**2.05**	/	/
PSPTO_5492	hypothetical protein PSPTO_5492	**2.40**	/	/
PSPTO_5495	hypothetical protein PSPTO_5495	**20.23**	**3.08**	**6.58**
PSPTO_5497	hypothetical protein PSPTO_5497	**12.09**	**5.03**	/
PSPTO_5514	hypothetical protein PSPTO_5514	**8.64**	/	**4.91**
PSPTO_5522	hypothetical protein PSPTO_5522	/	/	**2.13**
PSPTO_5553	methyl-accepting chemotaxis protein	**-5.37**	/	**-2.70**
PSPTO_5555	hypothetical protein PSPTO_5555	/	/	**3.82**
PSPTO_5558	hypothetical protein PSPTO_5558	**10.99**	/	**9.68**
PSPTO_5572		**5.89**	/	**3.83**
PSPTO_5574		/	/	**2.57**
PSPTO_5604	F0F1 ATP synthase subunit C	/	**2.04**	**-2.32**
PSPTO_5611	tRNA modification GTPase TrmE	/	/	**-2.15**
PSPTO_5613	hypothetical protein PSPTO_5613	/	/	**-2.43**
PSPTO_5621	hypothetical protein PSPTO_5621	/	**-2.93**	**4.82**
PSPTO_5622	hypothetical protein PSPTO_5622	**-2.79**	**-2.19**	/
PSPTO_5625	binary cytotoxin component	**9.04**	**2.69**	**3.36**
PSPTO_5630	hypothetical protein PSPTO_5630	**4.23**	/	**4.04**
PSPTO_5635	hypothetical protein PSPTO_5635	**-4.17**	/	**-2.51**
PSPTO_5636	hypothetical protein PSPTO_5636	**2.31**	/	/
PSPTO_5637	hypothetical protein PSPTO_5637	/	/	**-2.54**
PSPTO_5645	hypothetical protein PSPTO_5645	/	**2.29**	**-1.92**
PSPTO_5646	hypothetical protein PSPTO_5646	/	**2.39**	**-2.18**
PSPTO_5647	hypothetical protein	**-2.43**	/	/
PSPTO_5648	hypothetical protein	**-1.99**	**3.40**	**-6.76**
PSPTO_5650	hypothetical protein	**-2.25**	/	/
PSPTO_5652	hypothetical protein	**-42.31**	**-23.20**	/
PSPTO_5660	hypothetical protein	/	**-1.99**	**3.47**
PSPTO_5662	hypothetical protein	/	/	**-2.52**
PSPTO_5663	hypothetical protein	**-1.82**	/	/
PSPTO_5666	hypothetical protein	**-2.09**	/	**-2.23**
PSPTO_5668	hypothetical protein	**-2.56**	/	/
PSPTO_5669	hypothetical protein	**-6.84**	**-2.59**	**-2.64**
PSPTO_5671	hypothetical protein	**-91.63**	**-8.94**	**-10.25**
PSPTO_5672	hypothetical protein	**-16.63**	/	**-9.90**
PSPTO_5674	hypothetical protein	**-60.09**	/	**-38.81**
PSPTO_5675	hypothetical protein	**-18.70**	/	/
PSPTO_B0001	hypothetical protein	/	**2.47**	**-3.31**
PSPTO_B0009	hypothetical protein	**3.96**	/	**3.53**
PSPTO_B0012	hypothetical protein	**-3.32**	/	**-3.16**
PSPTO_B0013	hypothetical protein	**-1.99**	/	/

PSPTO_B0021	hypothetical protein	**/**	**/**	**2.05**
PSPTO_t07	hypothetical protein	**-2.23**	**-1.95**	**/**
PSPTO_t18	hypothetical protein	**/**	**/**	**-1.75**
PSPTO_t51	hypothetical protein	**/**	**2.22**	**-2.55**
PSPTOA0001	hypothetical protein	**/**	**2.30**	**-4.21**
PSPTOA0002	hypothetical protein	**/**	**/**	**-2.40**
PSPTOA0006		**-75.42**	**-52.72**	**/**
PSPTOA0007	hypothetical protein	**-2452.76**	**-2550.53**	**/**
PSPTOA0008	hypothetical protein	**-5205.27**	**-5311.96**	**/**
PSPTOA0009	hypothetical protein	**-3330.08**	**-3870.06**	**/**
PSPTOA0010	hypothetical protein	**/**	**-1.83**	**/**
PSPTOA0022	hypothetical protein	**/**	**-1.97**	**/**
PSPTOA0030	hypothetical protein	**-2.15**	**2.46**	**-5.30**
PSPTOA0031	hypothetical protein	**/**	**/**	**-2.19**
PSPTOA0032	hypothetical protein	**-1.96**	**/**	**-3.16**
PSPTOA0034	hypothetical protein	**-4.35**	**-2.36**	**-1.85**
PSPTOA0059	hypothetical protein	**/**	**/**	**-4.59**
PSPTOA0071	hypothetical protein	**/**	**3.04**	**-4.18**
