# Supplementary material for: The RsmA RNA-Binding Proteins in Pseudomonas syringae Exhibit Distinct and Overlapping Roles in Modulating Virulence and Survival Under Different Nutritional Conditions
Source: Front Plant Sci. 2021 Feb 26;12:637595. doi: 10.3389/fpls.2021.637595 (PMC7952654; doi:10.3389/fpls.2021.637595)
Supplement: Supplementary Table 4 — Fold changes of the type III secretion system (T3SS) and alginate biosynthesis genes in HMM. [file Data_Sheet_3.pdf]

Table S4. Fold changes of the type III secretion system (T3SS) and alginate biosynthesis genes in HMM

Locus tag	Description	**Δ*rsmA23*/ *Pst*DC3000**	**Δ*rsmA23*/ Δ*rsmA3***	**Δ*rsmA3*/ *Pst*DC3000**
**Type III secretion system**				
PSPTO_1404	*hrpL*, RNA polymerase sigma factor HrpL	**-504.9**	**-24.79**	**-20.37**
PSPTO_1379	*hrpR*, type III transcriptional regulator HrpR	**-6.82**	**-2.11**	**-3.24**
PSPTO_1380	*hrpS*, type III transcriptional regulator HrpS	**-7.05**	**-2.22**	**-3.18**
PSPTO_0061	*hopY1*, type III effector HopY1	**-80.53**	**-10.03**	**-8.03**
PSPTO_0588	*hopH1*, type III effector HopH1	**-117.78**	**-7.83**	**-15.05**
PSPTO_0589	*hopC1*, type III effector HopC1	**-75.79**	**-5.28**	**-14.35**
PSPTO_1370	*hopN1*, type III effector HopN1	**-43.08**	**-4.51**	**-9.56**
PSPTO_1372	*hopAA1-1*, type III effector HopAA1-1	**-62.9**	**-5.83**	**-10.79**
PSPTO_1375	*hopM1*, type III effector HopM1	**-29.03**	**-2.51**	**-11.54**
PSPTO_1377	*avrE1*, type III effector protein AvrE1	**-16.45**	**-2.79**	**-5.89**
PSPTO_1406	*hopB1*, type III effector HopB1	**-81.28**	**-6.58**	**-12.36**
PSPTO_2678	*hopP1*, type III helper protein HopP1	**-182.2**	**-10.69**	**-17.04**
PSPTO_3087	*hopAB2*, type III effector HopAB2	**-58.72**	**-5.41**	**-10.86**
PSPTO_4001	*avrPto1*, type III effector protein AvrPto1	**-84.14**	**-7.38**	**-11.4**
PSPTO_4331	*hopE1*, type III effector HopE1	**-213.12**	**-9.08**	**-23.46**
PSPTO_4588	*hopS2*, type III effector HopS2	**-47**	**-6.88**	**-6.83**
PSPTO_4597	*hopS1*, type III effector HopS1	**-140.87**	**-8.24**	**-17.09**
PSPTO_4776	*hopI1*, type III effector HopI1	**-75.24**	**-5.8**	**-12.98**
PSPTO_5354	*hopA1*, type III effector HopA1	**-69.62**	**-5.15**	**-13.53**
PSPTO_0502	*hopF2*, type III effector HopF2	**-15.08**	/	**-11.61**
PSPTO_0877	*hopQ1-1*, type III effector HopQ1-1	**-5.86**	/	**-4.24**
PSPTO_0901	*hopAG1*, type III effector HopAG1	**-2.37**	/	**-2.74**
PSPTO_1568	*hopAF1*, type III effector HopAF1	**-5.79**	/	**-3.33**
PSPTO_4691	*hopAD1*, type III effector HopAD1	**-15.83**	/	**-10.72**
PSPTO_4718	*hopAA1-2*, type III effector HopAA1-2	**-2.98**	/	**-1.83**
PSPTO_4720	*hopV1*, type III effector HopV1	**-8.73**	/	**-5.7**
PSPTO_4722	*hopAO1*, type III effector HopAO1	**-11.73**	/	**-8.71**
PSPTO_4727	*hopG1*, type III effector HopG1	**-18.6**	/	**-10.18**
PSPTOA0012	*hopX1*, type III effector HopX1	**-8.45**	/	**-5.99**
PSPTOA0018	*hopO1-1*, type III effector HopO1-1	**-3.14**	/	**-3.04**
PSPTOA0019	*hopT1-1*, type III effector HopT1-1	**-2.73**	/	**-2.45**
PSPTO_1391	*hrpV*, negative regulator of *hrp* expression	**-3.25**	/	**-3.37**
PSPTOA0017	*shc01*, type III chaperone ShcO1	**-2.7**	/	**-1.95**
PSPTO_1376	*shcE*, type III chaperone ShcE	**-4.67**	/	**-4.36**
PSPTO_0503	*shcF*, type III chaperone protein ShcF	**-54.35**	**-3.06**	**-17.77**
PSPTO_1369	*shcN*, type III chaperone protein ShcN	**-38.18**	**-3.67**	**-10.39**
PSPTO_1374	*shcM*, type III chaperone ShcM	**-142.67**	**-7.78**	**-18.33**
PSPTO_4589	*shcS2*, type III chaperone ShcS2	**-14.13**	**-2.35**	**-6.02**
PSPTO_4599	*shcS1*, type III chaperone ShcS1	**-54.99**	**-3.33**	**-16.52**

PSPTO_4721	*shcV*, type III chaperone ShcV	**-32.76**	**-2.85**	**-11.51**
PSPTO_5353	*shcA*, type III chaperone protein ShcA	**-73.98**	**-6.06**	**-12.2**
PSPTO_1390	*hrpT*, type III secretion protein HrpT	**-10.4**	/	**-7.46**
PSPTO_1384	*hrcJ*, type III secretion protein HrcJ	**-176.38**	**-12.42**	**-14.2**
PSPTO_1400	*hrcN*, type III secretion cytoplasmic ATPase	**-192.15**	**-12.53**	**-15.33**
PSPTO_1397	*hrcQa*, type III secretion protein HrcQa	**-997.41**	**-70.41**	**-14.17**
PSPTO_1396	*hrcQb*, type III secretion protein HrcQb	**-263.65**	**-14.84**	**-17.76**
PSPTO_1395	*hrcR*, type III secretion protein HrcR	**-126.58**	**-7.75**	**-16.34**
PSPTO_1394	*hrcS*, type III secretion protein HrcS	**-276.6**	**-21.81**	**-12.68**
PSPTO_1393	*hrcT*, type III secretion protein HrcT	**-161.66**	**-13.21**	**-12.24**
PSPTO_1392	*hrcU*, type III secretion protein HrcU	**-58.37**	**-4.89**	**-11.94**
PSPTO_1402	*hrcV*, type III secretion protein HrcV	**-222.59**	**-13.68**	**-16.27**
PSPTO_1383	*hrpB*, type III secretion protein HrpB	**-464.83**	**-28.76**	**-16.16**
PSPTO_1385	*hrpD*, type III secretion protein HrpD	**-181.24**	**-12.29**	**-14.75**
PSPTO_1386	*hrpE*, type III secretion protein HrpE	**-215.99**	**-13.02**	**-16.59**
PSPTO_1387	*hrpF*, type III secretion protein HrpF	**-402.02**	**-26.78**	**-15.01**
PSPTO_1388	*hrpG*, type III secretion protein HrpG	**-959.23**	**-57.35**	**-16.73**
PSPTO_1403	*hrpJ*, type III secretion protein HrpJ	**-204.17**	**-12.87**	**-15.86**
PSPTO_1399	*hrpO*, type III secretion protein HrpO	**-239.83**	**-16.13**	**-14.87**
PSPTO_1398	*hrpP*, type III secretion protein HrpP	**-384.41**	**-24.48**	**-15.7**
PSPTO_1401	*hrpQ*, type III secretion protein HrpQ	**-327**	**-19.28**	**-16.96**
PSPTO_1378	*hrpH*, lytic murein transglycosylase	**-142.88**	**-11.74**	**-12.17**
PSPTO_1389	*hrcC*, type III secretion protein HrcC	**-226.35**	**-15.82**	**-14.31**
PSPTO_0852	*hopAJ1*, type III helper protein HopAJ1	**-24.47**	**-3.26**	**-7.51**
PSPTO_4101	*hopAK1*, type III helper protein HopAK1	**-55.64**	**-4.78**	**-11.65**
PSPTO_1381	*hrpA1*, type III helper protein HrpA1	**-1084.47**	**-53.6**	**-20.23**
PSPTO_1405	*hrpK1*, type III helper protein HrpK1	**-281.56**	**-17.75**	**-15.86**
PSPTO_1373	*hrpW1*, type III helper protein HrpW1	**-248.19**	**-18.86**	**-13.16**
PSPTO_1382	*hrpZ1*, type III restriction system endonuclease	**-883.37**	**-45.44**	**-19.44**
**Alginate biosynthesis**				
PSPTO_1235	*algI*, alginate biosynthesis protein AlgI	**20.27**	**2.03**	**10**
PSPTO_1236	*algL*, alginate lyase AlgL	**14.18**	**2.13**	**6.65**
PSPTO_1237	*algX*, alginate biosynthesis protein AlgX	**19.92**	**2.07**	**9.62**
PSPTO_1238	*algG*, alginate biosynthesis protein AlgG	**22.1**	**2.08**	**10.62**
PSPTO_1239	*algE*, alginate biosynthesis protein AlgE	**20.37**	**2.03**	**10.04**
PSPTO_1240	*algK*, alginate biosynthesis protein AlgK	**20.41**	**1.91**	**10.69**
PSPTO_1241	*alg44*, alginate biosynthesis protein Alg44	**22.14**	**2.11**	**10.47**
PSPTO_1232	*algA*, alginate biosynthesis protein AlgA	**27.6**	**2.51**	**11**
PSPTO_1233	*algF*, alginate biosynthesis protein AlgF	**24.71**	**3.12**	**7.91**
PSPTO_1234	*algJ*, alginate biosynthesis protein AlgJ	**22.26**	**2.13**	**10.47**
PSPTO_1242	*alg8*, alginate biosynthesis protein Alg8	**22.67**	**2.04**	**11.12**
PSPTO_1243	*algD*, GDP-mannose 6-dehydrogenase AlgD	**26.46**	**3.18**	**8.33**

Differential expression in Δ*rsmA23* versus *Pst*DC3000, Δ*rsmA23* versus ΔrsmA3, and Δ*rsmA3* versus *Pst*DC3000 with fold change (FC)≥1.5 or FC≤-1.5 and p-value <0.05. /: non-differentially expressed genes (DEGs); HMM: *hrp*-inducing minimum medium; *Pst*DC3000: *Pseudomonas syringae* pv. *tomato* DC3000; Δ*rsmA3*: the *rsmA3* mutant; Δ*rsmA23*: the *rsmA2/A3* double mutant.

Table S5. Fold changes of the syringafactin biosynthesis genes in HMM

Locus tag	Description	**Δ*rsmA23* /*Pst*DC3000**	**Δ*rsmA23* /Δ*rsmA3***	**Δ*rsmA3* /*Pst*DC3000**
**Syringafactin biosynthesis**				
PSPTO_2828	syrR, transcriptional regulator SyrR	**12.93**	**-2.38**	**30.74**
PSPTO_2829	*syfA*, non-ribosomal peptide synthetase SyfA	**7.9**	**-7.37**	**58.2**
PSPTO_2830	*syfB*, non-ribosomal peptide synthetase SyfB	**4.56**	**-7.31**	**33.31**
PSPTO_2831	*syfC*, syringafactin efflux protein SyfC	**3.3**	**-5.39**	**17.77**
PSPTO_2832	*syfD*, syringafactin efflux protein SyfD	**2.6**	**-5.36**	**13.97**

Differential expression in Δ*rsmA23* versus *Pst*DC3000, Δ*rsmA23* versus ΔrsmA3, and Δ*rsmA3* versus *Pst*DC3000 with fold change (FC)≥1.5 or FC≤-1.5 and p-value <0.05. HMM: *hrp*-inducing minimum medium; *Pst*DC3000: *Pseudomonas syringae* pv. *tomato* DC3000; Δ*rsmA3*: the *rsmA3* mutant; Δ*rsmA23*: the *rsmA2/A3* double mutant.

Table S6. Selected genes regulated mainly by RsmA2 in HMM

Locus tag	Description	**Δ*rsmA23* /*Pst*DC3000**	**Δ*rsmA23* /Δ*rsmA3***	**Δ*rsmA3* /*Pst*DC3000**
**Positively regulated by RsmA2**				
**Sarcosine metabolism**				
PSPTO_0457	*soxG-1*, sarcosine oxidase, gamma subunit	**-2.9**	**-3.91**	/
PSPTO_0458	*soxA-1*, sarcosine oxidase, alpha subunit	**-2.94**	**-4.21**	/
PSPTO_0459	*soxD-1*, sarcosine oxidase subunit delta	**-3.52**	**-4.21**	/
PSPTO_0460	*soxB-1*, sarcosine oxidase, beta subunit	**-3.13**	**-3.86**	/
**Thiamine biosynthesis**				
PSPTO_4798	*thiD*, hydroxy-methylpyrimidine kinase	**-2.05**	**-2.06**	/
PSPTO_4799	*thiE*, thiamine-phosphate pyrophosphorylase	**-2.1**	**-2.01**	/
**Negatively regulated by RsmA2**				
**Oxidative stress response**				
PSPTO_0066	*oxyR*, oxidative stress regulatory OxyR	**2.2**	**2.16**	/
**Efflux pump**				
PSPTO_3099	*mexE*, multidrug efflux RND membrane fusion protein MexE	**3.21**	**2.95**	/
PSPTO_3100	*saxF*, aliphatic isothiocyanate resistance protein SaxF; AcrB/AcrD/AcrF family	**3.28**	**3**	/
PSPTO_3101	*oprN*, outer membrane efflux protein	**3.89**	**3.3**	/

Differential expression in Δ*rsmA23* versus *Pst*DC3000, Δ*rsmA23* versus ΔrsmA3, and Δ*rsmA3* versus *Pst*DC3000 with fold change (FC)≥1.5 or FC≤-1.5 and p-value <0.05. /: non-differentially expressed genes (DEGs); HMM: *hrp*-inducing minimum medium; *Pst*DC3000: *Pseudomonas syringae* pv. *tomato* DC3000; Δ*rsmA3*: the *rsmA3* mutant; Δ*rsmA23*: the *rsmA2/A3* double mutant.

Table S7. Selected genes regulated mainly by RsmA3 in HMM

Locus tag	Description	Δ*rsmA23* /*Pst*DC3000	Δ*rsmA23* /Δ*rsmA3*	Δ*rsmA3* /*Pst*DC3000
**Positively regulated by RsmA3**				
**Fatty acid biosynthesis/degradation**				
PSPTO_5093	acyl carrier protein	**-3.51**	/	**-2.51**
PSPTO_4860	*accB*, acetyl-CoA carboxylase, biotin carboxyl carrier protein	**-1.96**	/	**-2.11**
PSPTO_3706	acyl-CoA dehydrogenase	**2.74**	/	**2.95**
PSPTO_3857	acyl-CoA dehydrogenase	**5.63**	/	**4.27**
PSPTO_5020	acyl-CoA dehydrogenase family protein	**2.15**	/	**2.09**
PSPTO_0667	short-chain dehydrogenase	**3.54**	/	**2.97**
PSPTO_1056	short chain dehydrogenase	**3.76**	/	**4.38**
PSPTO_1119	short chain dehydrogenase	**3.41**	/	**2.41**
**Cellulose biosynthesis**				
PSPTO_1026	*wssA*, cell morphology protein	**-3.27**	/	**-3.05**
PSPTO_1027	*wssB*, cellulose synthase, catalytic subunit	**-1.79**	/	**-1.96**
PSPTO_1029	*wssD*, endoglucanase	**-2.21**	/	**-1.64**
**Two-component system**				
PSPTO_4373	*colS*, sensor histidine kinase ColS	**-1.99**	/	**-1.64**
PSPTO_4374	*colR*, response regulator ColR	**-1.91**	/	**-1.86**
**Negatively regulated by RsmA3**				
**Signal transduction**				
PSPTO_0073	*spoT*, guanosine-3',5'-bis(diphosphate,3'-pyrophosphohydrolase	**-1.38**	/	**-1.75**
PSPTO_0856	*fpRel*, hypothetical protein	**1.93**	/	**2.3**
PSPTO_0969	dksA, dnaK suppressor protein	**2.13**	/	**2.11**
PSPTO_3863	*psyR*, transcriptional regulator PsyR	**3.45**	/	**2.1**
PSPTO_3864	*psyI*, autoinducer synthesis protein PsyI	**1.62**	/	**1.51**
PSPTO_1499	*wspR*, GGDEF domain-containing protein	**2.42**	/	**2.32**
PSPTO_5477	*phoB*, phosphate regulon protein PhoB	**3.02**	/	**2.43**
PSPTO_5478	*phoR*, phosphate regulon sensor phoR	**2.05**	/	**1.81**
PSPTO_5483	*phoU*, phosphate transport system PhoU	**3.17**	/	**1.99**
PSPTO_5484	*pstB-2*, phosphate transporter ATP-binding protein	**3.67**	/	**2.17**
**Chemotaxis**				
PSPTO_0908	*cheB-1,* glutamate methylesterase CheB	**13.42**	/	**17.54**
PSPTO_0909	glutamine deamidase CheD	**32.07**	/	**30.81**
PSPTO_0910	*cheR-1,* chemotaxis protein CheR	**123.62**	/	**97.38**
PSPTO_0911	*cheW-1,* chemotaxis protein CheW	**204.42**	/	**175.37**
PSPTO_0912	methyl-accepting chemotaxis protein	**144.44**	/	**101.24**
PSPTO_0913	*cheA-1,* chemotaxis histidine kinase CheA	**132.04**	/	**107.78**
PSPTO_0915	*cheY-1,* chemotaxis protein CheY	**176.81**	/	**119.59**
PSPTO_0916	*methyl-accepting chemotaxis protein*	**77.78**	/	**54.03**

PSPTO_1008	methyl-accepting chemotaxis protein	**16.67**	/	**11.48**
PSPTO_1493	methyl-accepting chemotaxis protein	**3.22**	/	**3.15**
PSPTO_1494	chemotaxis protein CheW	**2.93**	/	**2.9**
PSPTO_1495	chemotaxis methyltransferase CheR	**2.76**	/	**2.94**
PSPTO_1496	chemotaxis protein CheW	**2.84**	/	**3.25**
PSPTO_1928	*cheR-2*, chemotaxis protein methyltransferase CheR	**2.59**	/	**2.81**
PSPTO_2526	methyl-accepting chemotaxis protein	**2.88**	/	**5.74**
PSPTO_4541	methyl-accepting chemotaxis protein	**2.31**	/	**2.62**
PSPTO_4786	methyl-accepting chemotaxis protein	**2.54**	/	**2.12**
PSPTO_5159	methyl-accepting chemotaxis protein	**3.27**	/	**2.92**
**Type VI secretion system**				
PSPTO_3482	Rhs element Vgr protein	**2.1**	/	**1.92**
PSPTO_4385	Rhs element Vgr protein	**2.42**	/	**2.17**
PSPTO_5415	Rhs element Vgr protein	**1.75**	/	**1.99**
PSPTO_5418	hypothetical protein PSPTO_5418	**1.79**	/	**2.19**
PSPTO_5425	clpB protein	**1.95**	/	**2.36**
PSPTO_5435	*hcp-2*, secreted protein Hcp	**13.72**	/	**11.47**
PSPTO_5436	Rhs element Vgr protein	**6.38**	/	**7**
PSPTO_5438	Rhs family protein	**3.75**	/	**4.11**
**Capsular polysaccharide**				
PSPTO_3529	*pslA*, capsular polysaccharide biosynthesis protein PslA	**44.49**	/	**35.66**
PSPTO_3530	*pslB*, mannose-6-phosphate isomerase PslB	**34.38**	/	**29.57**
PSPTO_3531	*pslD*, lipoprotein PslD	**49.77**	/	**38.29**
PSPTO_3532	*pslE*, exopolysaccharide biosynthesis PslE	**55.35**	/	**43.74**
PSPTO_3533	*pslF*, glycosyl transferase PslF	**36.21**	/	**34.33**
PSPTO_3534	*pslG*, glycosyl hydrolase, family 5 PslG	**20.75**	/	**17.78**
PSPTO_3535	*pslH*, glycosyl transferase PslH	**25.99**	/	**23.63**
PSPTO_3536	*pslI*, glycosyl transferase PslI	**31.85**	/	**26.82**
PSPTO_3537	*pslJ*, membrane protein PslJ	**22.25**	/	**20.75**
PSPTO_3539	*pslK*, membrane protein PslK	**16.1**	/	**18.14**
**Osmotic/Oxidative stress response**				
PSPTO_0152	*osmC*, osmotically inducible protein	**4.81**	/	**3.77**
PSPTO_1338	*sodC*, superoxide dismutase Cu-Zn	**7.65**	/	**8.15**
PSPTO_4530	*katG*, catalase/peroxidase HPI	**3.53**	/	**2.62**
PSPTO_5263	*katE*, catalase	**18.24**	/	**20.35**

Differential expression in Δ*rsmA23* versus *Pst*DC3000, Δ*rsmA23* versus ΔrsmA3, and Δ*rsmA3* versus *Pst*DC3000 with fold change (FC)≥1.5 or FC≤-1.5 and p-value <0.05. /: non-differentially expressed genes (DEGs); HMM: *hrp*-inducing minimum medium; *Pst*DC3000: *Pseudomonas syringae* pv. *tomato* DC3000; Δ*rsmA3*: the *rsmA3* mutant; Δ*rsmA23*: the *rsmA2/A3* double mutant.

Table S8. Fold changes of the chemotaxis genes in KB

Locus tag	Description	**Δ*rsmA23* /*Pst*DC3000**	**Δ*rsmA23* /Δ*rsmA3***	**Δ*rsmA3* /*Pst*DC3000**
**Chemotaxis**				
PSPTO_0117	methyl-accepting chemotaxis protein	**-5.45**	**-2.65**	**-2.05**
PSPTO_2480	methyl-accepting chemotaxis protein	**-4.84**	**-2.24**	**-2.16**
PSPTO_2511	methyl-accepting chemotaxis protein	**-5.29**	**-2.48**	**-2.13**
PSPTO_2616	methyl-accepting chemotaxis protein	**-5.2**	**-2.78**	**-1.87**
PSPTO_3680	methyl-accepting chemotaxis protein	**-5.07**	**-2.41**	**-2.11**
PSPTO_1323	chemotaxis protein CheV	**-2.92**	**-2.15**	/
PSPTO_1334	methyl-accepting chemotaxis protein	**-4.62**	**-3.05**	/
PSPTO_1495	chemotaxis protein methyltransferase CheR	**-2.62**	**-1.95**	/
PSPTO_1496	chemotaxis protein CheW	**-2.72**	**-2.22**	/
PSPTO_2441	methyl-accepting chemotaxis protein	**-2.59**	**-3.88**	/
PSPTO_2472	methyl-accepting chemotaxis protein	**-2.70**	**-2.28**	/
PSPTO_2475	methyl-accepting chemotaxis protein	**-1.99**	**-2.20**	/
PSPTO_2883	methyl-accepting chemotaxis protein	**-3.44**	**-2.31**	/
PSPTO_3237	methyl-accepting chemotaxis protein	**-3.65**	**-2.27**	/
PSPTO_3379	methyl-accepting chemotaxis protein	**-1.96**	**-1.85**	/
PSPTO_3480	methyl-accepting chemotaxis protein	**-2.30**	**-2.46**	/
PSPTO_3524	chemotaxis protein CheV	**-3.03**	**-2.04**	/
PSPTO_3577	methyl-accepting chemotaxis protein	**-2.67**	**-2.13**	/
PSPTO_3699	methyl-accepting chemotaxis protein	**-3.03**	**-2.30**	/
PSPTO_0466	methyl-accepting chemotaxis protein	**-4.07**	/	**-3.41**
PSPTO_1927	chemotaxis protein CheV	**-3.15**	/	**-1.80**
PSPTO_1928	chemotaxis protein methyltransferase CheR	**-3.01**	/	**-1.89**
PSPTO_4531	methyl-accepting chemotaxis protein	**-13.83**	/	**-7.17**
PSPTO_5553	methyl-accepting chemotaxis protein	**-5.37**	/	**-2.70**

Differential expression in Δ*rsmA23* versus *Pst*DC3000, Δ*rsmA23* versus ΔrsmA3, and Δ*rsmA3* versus *Pst*DC3000 with fold change (FC)≥1.5 or FC≤-1.5 and p-value <0.05. /: non-differentially expressed genes (DEGs); KB: King's B medium; *Pst*DC3000: *Pseudomonas syringae* pv. *tomato* DC3000; Δ*rsmA3*: the *rsmA3* mutant; Δ*rsmA23*: the *rsmA2/A3* double mutant.

Table S9. Fold changes of the ribosome genes in KB

Locus tag	Description	**Δ*rsmA23* /*Pst*DC3000**	**Δ*rsmA23* /Δ*rsmA3***	**Δ*rsmA3* /*Pst*DC3000**
**Ribosome proteins**				
PSPTO_0615	*rplK*, 50S ribosomal protein L11	**-1.9**	**1.79**	**-3.4**
PSPTO_0616	*rplA*, 50S ribosomal protein L1	**-1.94**	**1.87**	**-3.62**
PSPTO_0617	*rplJ*, 50S ribosomal protein L10	**-2.34**	**2.38**	**-5.58**
PSPTO_0618	*rplL*, 50S ribosomal protein L7/L12	**-2.29**	**2.88**	**-6.58**
PSPTO_0627	*rplD*, 50S ribosomal protein L4	**-1.77**	**2.15**	**-3.81**

PSPTO_0628	*rplW*, 50S ribosomal protein L23	**-1.89**	**2.01**	**-3.78**
PSPTO_0644	*rpmD*, 50S ribosomal protein L30	**-1.81**	**2.47**	**-4.46**
PSPTO_0648	*rpsM*, 30S ribosomal protein S13	**-1.76**	**2.33**	**-4.1**
PSPTO_0650	*rpsD*, 30S ribosomal protein S4	**-1.79**	**2.29**	**-4.1**
PSPTO_0652	*rplQ*, 50S ribosomal protein L17	**-1.91**	**2.69**	**-5.13**
PSPTO_0797	*rplU*, 50S ribosomal protein L21	**-1.85**	**1.86**	**-3.43**
PSPTO_0798	*rpmA*, 50S ribosomal protein L27	**-1.78**	**2.01**	**-3.58**
PSPTO_0619	*rpoB*, RNA polymerase subunit beta	/	**3.25**	**-5.83**
PSPTO_0621	*rpsL*, 30S ribosomal protein S12	/	**1.74**	**-2.79**
PSPTO_0622	*rpsG*, 30S ribosomal protein S7	/	**1.91**	**-3.18**
PSPTO_0625	*rpsJ*, 30S ribosomal protein S10	/	**1.83**	**-3.07**
PSPTO_0626	*rplC*, 50S ribosomal protein L3	/	**1.98**	**-3.44**
PSPTO_0629	*rplB*, 50S ribosomal protein L2	/	**2.11**	**-3.34**
PSPTO_0630	*rpsS*, 30S ribosomal protein S19	/	**2.08**	**-3.63**
PSPTO_0631	*rplV*, 50S ribosomal protein L22	/	**2.14**	**-3.65**
PSPTO_0632	*rpsC*, 30S ribosomal protein S3	/	**2.43**	**-3.67**
PSPTO_0633	*rplP*, 50S ribosomal protein L16	/	**2.73**	**-4.1**
PSPTO_0634	*rpmC*, 50S ribosomal protein L29	/	**3.13**	**-4.63**
PSPTO_0635	*rpsQ*, 30S ribosomal protein S17	/	**3.21**	**-4.94**
PSPTO_0636	*rplN*, 50S ribosomal protein L14	/	**3.23**	**-5.04**
PSPTO_0637	*rplX*, 50S ribosomal protein L24	/	**3.18**	**-4.98**
PSPTO_0638	*rplE*, 50S ribosomal protein L5	/	**3.28**	**-5.92**
PSPTO_0639	*rpsN*, 30S ribosomal protein S14	/	**2.93**	**-5.13**
PSPTO_0640	*rpsH*, 30S ribosomal protein S8	/	**2.11**	**-3.52**
PSPTO_0641	*rplF*, 50S ribosomal protein L6	/	**2.32**	**-3.73**
PSPTO_0642	*rplR*, 50S ribosomal protein L18	/	**2.44**	**-3.75**
PSPTO_0643	*rpsE*, 30S ribosomal protein S5	/	**2.54**	**-3.98**
PSPTO_0645	*rplO*, 50S ribosomal protein L15	/	**2.52**	**-4.34**
PSPTO_0647	*rpmJ*, 50S ribosomal protein L36	/	**2.65**	**-4.68**
PSPTO_0649	*rpsK*, 30S ribosomal protein S11	/	**2.34**	**-4.21**
PSPTO_1473	*rpsP*, 30S ribosomal protein S16	/	**1.82**	**-2.99**
PSPTO_1476	*rplS*, 50S ribosomal protein L19	/	**2.22**	**-3.76**
PSPTO_4930	*rplI*, 50S ribosomal protein L9	/	**2.64**	**-4.4**
PSPTO_4932	*rpsR*, 30S ribosomal protein S18	/	**2.29**	**-3.98**
PSPTO_4933	*rpsF*, 30S ribosomal protein S6	/	**2.16**	**-3.75**

Differential expression in Δ*rsmA23* versus *Pst*DC3000, Δ*rsmA23* versus ΔrsmA3, and Δ*rsmA3* versus *Pst*DC3000 with fold change (FC)≥1.5 or FC≤-1.5 and p-value <0.05. /: non-differentially expressed genes (DEGs); KB: King's B medium; *Pst*DC3000: *Pseudomonas syringae* pv. *tomato* DC3000; Δ*rsmA3*: the *rsmA3* mutant; Δ*rsmA23*: the *rsmA2/A3* double mutant.

Table S10. Selected genes regulated mainly by RsmA2 in KB

Locus tag	Description	**Δ*rsmA23* /*Pst*DC3000**	**Δ*rsmA23* /Δ*rsmA3***	**Δ*rsmA3* /*Pst*DC3000**
**Positive regulated by RsmA2**				
**Capsular polysaccharide**				
PSPTO_3529	*pslA*, capsular polysaccharide biosynthesis protein PslA	**-3.03**	**-2.27**	/
PSPTO_3530	*pslB*, mannose-6-phosphate isomerase PslB	**-2.5**	**-2.37**	/
PSPTO_3531	*pslD*, lipoprotein *PslD*	**-2.54**	**-2.94**	/
**Mannitol metabolism**				
PSPTO_2703	*uxuB*, D-mannonate oxidoreductase	**-3.76**	**-3.84**	/
PSPTO_2705	mannitol ABC transporter permease	**-3.94**	**-2.47**	/
PSPTO_2706	mannitol ABC transporter permease	**-4.73**	**-3.02**	/
PSPTO_2707	mannitol ABC transporter	**-2.83**	**-2.53**	/
**Negatively regulated by RsmA2**				
**Alginate biosynthesis**				
PSPTO_1232	*algA*, alginate biosynthesis protein AlgA	**3.11**	**2.38**	/
PSPTO_1236	*algL*, alginate lyase	**2.83**	**2.3**	/
PSPTO_1237	*algX*, alginate biosynthesis protein AlgX	**2.24**	**3.21**	/
PSPTO_1238	*algG*, alginate biosynthesis protein AlgG	**2.8**	**5.14**	/
PSPTO_1239	*algE*, alginate biosynthesis protein AlgE	**5.73**	**8.14**	/
PSPTO_1240	*algK*, alginate biosynthesis protein AlgK	**8.21**	**8.08**	/
PSPTO_1241	*alg44*, alginate biosynthesis protein Alg44	**8.14**	**3.98**	/
**Phosphate regulation**				
PSPTO_5477	*phoB*, phosphate regulon transcriptional regulatory protein PhoB	**6.19**	**6.65**	/
PSPTO_5478	*phoR*, phosphate regulon sensor protein phoR	**4.24**	**5.6**	/
PSPTO_5486	phosphate ABC transporter permease	**4.09**	**5**	/
PSPTO_5487	phosphate ABC transporter substrate-binding protein	**9.75**	**7.63**	/

Differential expression in Δ*rsmA23* versus *Pst*DC3000, Δ*rsmA23* versus ΔrsmA3, and Δ*rsmA3* versus *Pst*DC3000 with fold change (FC)≥1.5 or FC≤-1.5 and p-value <0.05. /: non-differentially expressed genes (DEGs); KB: King's B medium; *Pst*DC3000: *Pseudomonas syringae* pv. *tomato* DC3000; Δ*rsmA3*: the *rsmA3* mutant; Δ*rsmA23*: the *rsmA2/A3* double mutant.

Table S11. Selected genes regulated mainly by RsmA3 in KB

Locus tag	Gene description	Δ*rsmA23* /*Pst*DC3000	Δ*rsmA23* /Δ*rsmA3*	Δ*rsmA3* /*Pst*DC3000
**Positive regulated by RsmA3**				
**Fatty acid Biosynthesis /Degradation**				
PSPTO_3832	*fabG*, 3-ketoacyl-(acyl-carrier-protein) reductase	**-1.94**	/	**-3.42**
PSPTO_3833	*fabD*, malonyl CoA-acyl carrier protein transacylase	**-2.06**	/	**-3.14**
PSPTO_5093	acyl carrier protein	**-4.48**	/	**-4.34**
PSPTO_5094	acyl carrier protein	**-3.39**	/	**-3.48**
PSPTO_3834	fatty acid/phospholipid synthesis protein	**-2.38**	/	**-2.96**
PSPTO_4860	*accB*, acetyl-CoA carboxylase, biotin carboxyl carrier protein	**-1.74**	/	**-2.26**
PSPTO_0500	acyl-CoA dehydrogenase family protein	**2.65**	/	**1.99**
PSPTO_1056	short chain dehydrogenase	**3.85**	/	**3.77**
PSPTO_3706	acyl-CoA dehydrogenase	**4.23**	/	**4.80**
PSPTO_3857	acyl-CoA dehydrogenase	**3.93**	/	**3.10**
**Cellulose biosynthesis**				
PSPTO_1026	*wssA*, cell morphology protein	**-2.52**	/	**-2.55**
PSPTO_1027	*wssB*, cellulose synthase, catalytic subunit	**-2.04**	/	**-4.49**
PSPTO_1029	*wssD*, endoglucanase	**-2.34**	/	**-4.47**
**Two-component system**				
PSPTO_4373	*colS*, sensor histidine kinase ColS	**-1.89**	/	**-2.24**
PSPTO_4374	*colR*, DNA-binding response regulator ColR	**-1.78**	/	**-1.48**
**Negatively regulated by RsmA3**				
**Signal transduction**				
PSPTO_0073	*spoT*, guanosine-3',5'-bis(diphosphate),3'-pyrophosphohydrolase	**-2.09**	/	**-2.12**
PSPTO_0856	*fpRel*, hypothetical protein	**2.41**	/	**2.67**
PSPTO_3863	*psyR*, transcriptional regulator PsyR	**1.81**	/	**2.18**
PSPTO_3864	*psyI*, autoinducer synthesis protein PsyI	**12.81**	/	**15.37**
**Type VI secretion system**				
PSPTO_0373	Rhs family protein	**3.08**	/	**3.63**
PSPTO_4385	Rhs element Vgr protein	**2.42**	/	**3.04**
PSPTO_5435	*hcp-2*, secreted protein Hcp	**3.83**	/	**4.08**
PSPTO_5436	Rhs element Vgr protein	**5.38**	/	**4.1**
PSPTO_5438	Rhs family protein	**2.11**	/	**1.71**
**Syringafactin biosynthesis**				
PSPTO_2828	*syrR*, transcriptional regulator SyrR	**5.02**	**-1.96**	**9.82**
PSPTO_2829	*syfA*, non-ribosomal peptide synthetase	**4.70**	/	**7.98**
**Alginate biosynthesis**				
PSPTO_1234	*algJ*, alginate biosynthesis protein AlgJ	**5.13**	/	**3.83**

PSPTO_1235	*algI*, alginate biosynthesis protein AlgI	**4.8**	/	**3.04**
PSPTO_1242	*alg8*, alginate biosynthesis protein Alg8	**6.51**	/	**3.71**
**Capsular polysaccharide**				
PSPTO_3536	*pslI*, glycosyl transferase PslI	**3.08**	/	**2.56**
PSPTO_3537	*pslJ*, membrane protein PslJ	**3.29**	/	**2.64**
**Osmotic and oxidative stress response**				
PSPTO_0152	*osmC*, osmotically inducible protein	**1.89**	/	**2**
PSPTO_1338	*sodC*, superoxide dismutase, Cu-Zn	**2.45**	/	**2.29**
PSPTO_3582	*katB*, catalase	**4.31**	/	**4.38**
PSPTO_5263	*katE*, catalase	**8.28**	/	**8.56**

Differential expression in Δ*rsmA23* versus *Pst*DC3000, Δ*rsmA23* versus ΔrsmA3, and Δ*rsmA3* versus *Pst*DC3000 with fold change (FC)≥1.5 or FC≤-1.5 and p-value <0.05. /: non-differentially expressed genes (DEGs); KB: King's B medium; *Pst*DC3000: *Pseudomonas syringae* pv. *tomato* DC3000; Δ*rsmA3*: the *rsmA3* mutant; Δ*rsmA23*: the *rsmA2/A3* double mutant.
